# Ruthenium-catalyzed formal sp^3^ C–H activation of allylsilanes/esters with olefins: efficient access to functionalized 1,3-dienes†

**DOI:** 10.1039/d0sc06845d

**Published:** 2021-02-02

**Authors:** Dattatraya H. Dethe, Nagabhushana C. Beeralingappa, Saikat Das, Appasaheb K. Nirpal

**Affiliations:** Department of Chemistry, Indian Institute of Technology Kanpur Kanpur 208016 India ddethe@iitk.ac.in

## Abstract

Ru-catalysed oxidative coupling of allylsilanes and allyl esters with activated olefins has been developed *via* isomerization followed by C(allyl)–H activation providing efficient access to stereodefined 1,3-dienes in excellent yields. Mild reaction conditions, less expensive catalysts, and excellent regio- and diastereoselectivity ensure universality of the reaction. In addition, the unique power of this reaction was illustrated by performing the Diels–Alder reaction, and enantioselective synthesis of highly functionalized cyclohexenone and piperidine and finally synthetic utility was further demonstrated by the efficient synthesis of norpyrenophorin, an antifungal agent.

## Abstract 2

Ru-catalysed oxidative coupling of allylsilanes and allyl esters with activated olefins has been developed *via* isomerization followed by C(allyl)–H activation providing efficient access to stereodefined 1,3-dienes in excellent yields.

## Graphical abstract

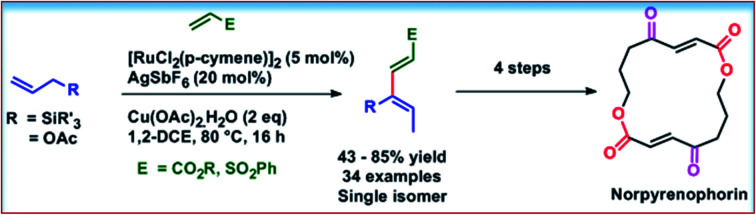

1,3-Dienes not only are widespread structural motifs in biologically pertinent molecules but also feature as a foundation for a broad range of chemical transformations.^1–14^ Indeed, these conjugated dienes serve as substrates in many fundamental synthetic methodologies such as cycloaddition, metathesis, ene reactions, oxidoreduction, or reductive aldolization. It is well-understood that the geometry of olefins often influences the stereochemical outcome and the reactivity of reactions involving 1,3-dienes.^15^ Hence, a plethora of synthetic methods have been developed for the stereoselective construction of substituted 1,3-dienes.^16–24^ The past decade has witnessed a huge advancement in the field of metal-catalyzed C–H activation/functionalization.^25–27^ Although, a significant amount of work in the field of C(alkyl)–H and C(aryl)–H activation has been reported; C(alkenyl)–H activation has not been explored conspicuously, probably due to the complications caused by competitive reactivity of the alkene moiety, which can make chemoselectivity a significant challenge. Over the past few years, several different palladium-based protocols have been developed for C(alkenyl)–H functionalization, but the reactions are generally limited to employing conjugated alkenes, such as styrenes,^28–31^ acrylates/acrylamides,^32–36^ enamides,^37^ and enol esters/ethers.^38,39^ To date, only a few reports have appeared in the literature for expanding this reactivity towards non-conjugated olefins, which can be exemplified by camphene dimerization,^40^ and carboxylate-directed C(alkenyl)–H alkenylation of 1,4-cyclohexadienes.^41^ In 2009, Trost *et al.* reported a ruthenium-catalyzed stereoselective alkene–alkyne coupling method for the synthesis of 1,3-dienes.^42^ The same group also reported alkene–alkyne coupling for the stereoselective synthesis of trisubstituted ene carbamates.^43^ A palladium catalyzed chelation control method for the synthesis of dienes *via* alkenyl sp^2^ C–H bond functionalization was described by Loh *et al.*^44^ Recently, Engle and coworkers reported an elegant approach for synthesis of highly substituted 1,3-dienes from two different alkenes using an 8-aminoquinoline directed, palladium(ii)-mediated C(alkenyl)–H activation strategy.^45^ Allyl and vinyl silanes are known as indispensable nucleophiles in synthetic chemistry.^46^ Alder ene reactions of allyl silanes with alkynes are reported for the synthesis of 1,4-dienes.^47^ Innumerable methods are known for the preparation of both allyl and vinyl silanes^48–52^ but limitations are associated with many of the current protocols, which impedes the synthesis of unsaturated organosilanes in an efficient manner. Silicon-functionalized building blocks are used as coupling partners in the Hiyama reaction^53^ and are easily converted into iodo-functionalized derivatives (precursor for the Suzuki cross-coupling reaction), but there is little attention given for the synthesis of functionalized vinyl silanes. Herein, we report a general approach for the stereoselective synthesis of trisubstituted 1,3-dienes by the Ru-catalyzed C(sp^3^)–H functionalization reaction of allylsilanes (Scheme 1).

**Scheme 1:**
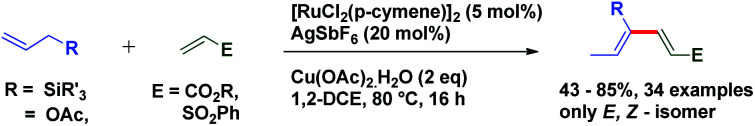
Highly stereoselective construction of 1,3-dienes.

In 1993, Trost and coworkers reported an elegant method for highly chemoselective ruthenium-catalyzed redox isomerization of allyl alcohols without affecting the primary and secondary alcohols and isolated double bonds.^54,55^ Inspired by the potential of ruthenium for such isomerization of double bonds in allyl alcohols, we sought to identify a ruthenium-based catalytic system that can promote isomerization of olefins in allylsilanes followed by *in situ* oxidative coupling with an activated olefin to form substituted 1,3-dienes. We initiated our studies by choosing trimethylallylsilane **1a** and acrylate **2a** by using a commercially available [RuCl_2_(*p*-cymene)]_2_ catalyst in the presence of AgSbF_6_ as an additive and co-oxidant Cu(OAc)_2_ in 1,2-DCE at 100 °C. Interestingly, it resulted into direct formation of (2*E*,4*Z*)-1,3-diene **3aa** as a single isomer in 55% yield. It is likely that this reaction occurs by C(allyl)–H activation of the π-allyl ruthenium complex followed by oxidative coupling with the acrylate and leaving the silyl group intact (Table 1). π-Allyl ruthenium complex formation may be highly favorable due to the α-silyl effect which stabilizes the carbanion forming *in situ* in the reaction.^56^ Next, the regioselective C–H insertion of vinyl silanes could be controlled by stabilization of the carbon–metal (C–M) bond in the α-position to silicon. This stability arises due to the overlapping of the filled carbon–metal orbital with the d orbitals on silicon or the antibonding orbitals of the methyl–silicon (Me–Si) bond.^57^ The stereochemistry of the diene was established by **1D** and **2D** spectroscopic analysis of the compound **3aa**. To quantify the C–H activation mediated coupling efficiency, an extensive optimization study was conducted (allylsilanes followed by *in situ* oxidative coupling with an activated olefin to form substituted 1,3-dienes). The change of solvents from 1,2-DCE to t-AmOH, DMF, dioxane, THF or MeCN did not give any satisfactory result, rather a very sluggish reaction rate or decomposition of starting materials was observed in each case (entry 2–6).

**Table 1:** Optimization of reaction conditions^a^

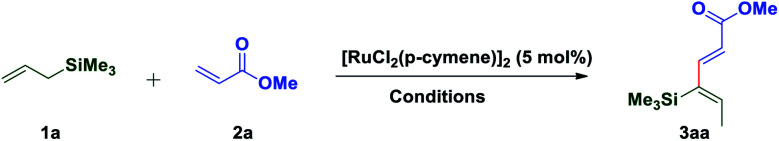				
Entry	Additive (20 mol%)	Oxidant (2 equiv.)	Solvent	Yield^b^ (%)
1	AgSbF_6_	Cu(OAc)_2_	DCE	55
2	AgSbF_6_	Cu(OAc)_2_	t-AmOH	10
3	AgSbF_6_	Cu(OAc)_2_	DMF	0
4	AgSbF_6_	Cu(OAc)_2_	Dioxane	8
5	AgSbF_6_	Cu(OAc)_2_	THF	21
6	AgSbF_6_	Cu(OAc)_2_	MeCN	0
7^c^	AgSbF_6_	Cu(OAc)_2_	DCE	35
8^d^	AgSbF_6_	Cu(OAc)_2_	DCE	82
9^e^	AgSbF_6_	Cu(OAc)_2_	DCE	45
10^d^	Ag_2_CO_3_	Cu(OAc)_2_	DCE	0
11^d^	AgOAc	Cu(OAc)_2_	DCE	20
12^d^	AgSbF_6_	—	DCE	0

a Reaction conditions: **1a** (0.24 mmol), **2a** (0.2 mmol), [Ru(*p*-cymene)Cl_2_]_2_ (5 mol%), additive (20 mol%) and oxidant (2 equiv.) at 100 °C in a specific solvent (2.0 mL), under argon, for 16 h.

b Isolated yields are of product **3aa**.

c The reaction was performed at 120 °C.

d The reaction was performed at 80 °C.

e The reaction was performed at 60 °C. t-AmOH – tertiary amyl alcohol, DMF – *N*,*N*-dimethylformamide, DCE – 1,2-dichloroethane.

The increase of temperature from 100 °C to 120 °C resulted in the formation of diene in lower yield (entry 7). To our delight, it was found that a substantial enhancement in the yield (82%) was observed when the reaction was performed at 80 °C (entry 8). In particular, this was found to be the best reaction condition since further lowering of the temperature led to noteworthy attenuation of the reaction rate and yield (entry 9). Interestingly, the reaction was not efficient, when AgSbF_6_ was replaced with other additives, such as Ag_2_CO_3_ and AgOAc. It was also observed that, co-oxidant Cu(OAc)_2_ is necessary for the success of this reaction (entry 12).

With these optimized conditions in hand, various allyl sources and acrylates have been tested (Table 2). It was found that a variety of acrylates **2** bearing alkyl and sterically crowded cyclic substituents successfully underwent the coupling reaction with allyl silane **1a** to afford corresponding silyl substituted (2*E*,4*Z*)-1,3-dienes in good yields (**3aa–3af**). Similarly, dimethyl benzylallylsilane **1b** reacted smoothly with acrylates such as methyl, isobutyl and *n*-butyl to generate desired dienes **3ba**, **3bb** and **3bc** in 83%, 85% and 82% yield respectively. Interestingly, sterically crowded, *tert*-butyldimethyl allylsilane **1c** showed its reactivity towards the coupling reaction with *n*-butyl acrylate to provide required diene **3cb** in 80% yield. It is worth mentioning that allylsilanes **1a** and **1b** also exhibited their coupling reactivity with phenyl vinyl sulfone and successfully generated corresponding 1,3-dienes **3ag** and **3bg** in 78% and 76% yield respectively. When *tert*-butyldiphenylallylsilane **1d** was subjected to the coupling reaction with methyl acrylate **2a**, end–end coupling product **3da** was isolated in 68% yield. This may be attributed to the steric crowding offered by bulky groups on silicon which prevents allyl to vinyl isomerization.

**Table 2:** Substrate scope for oxidative coupling of allylsilanes with acrylates and vinyl sulfones^a^

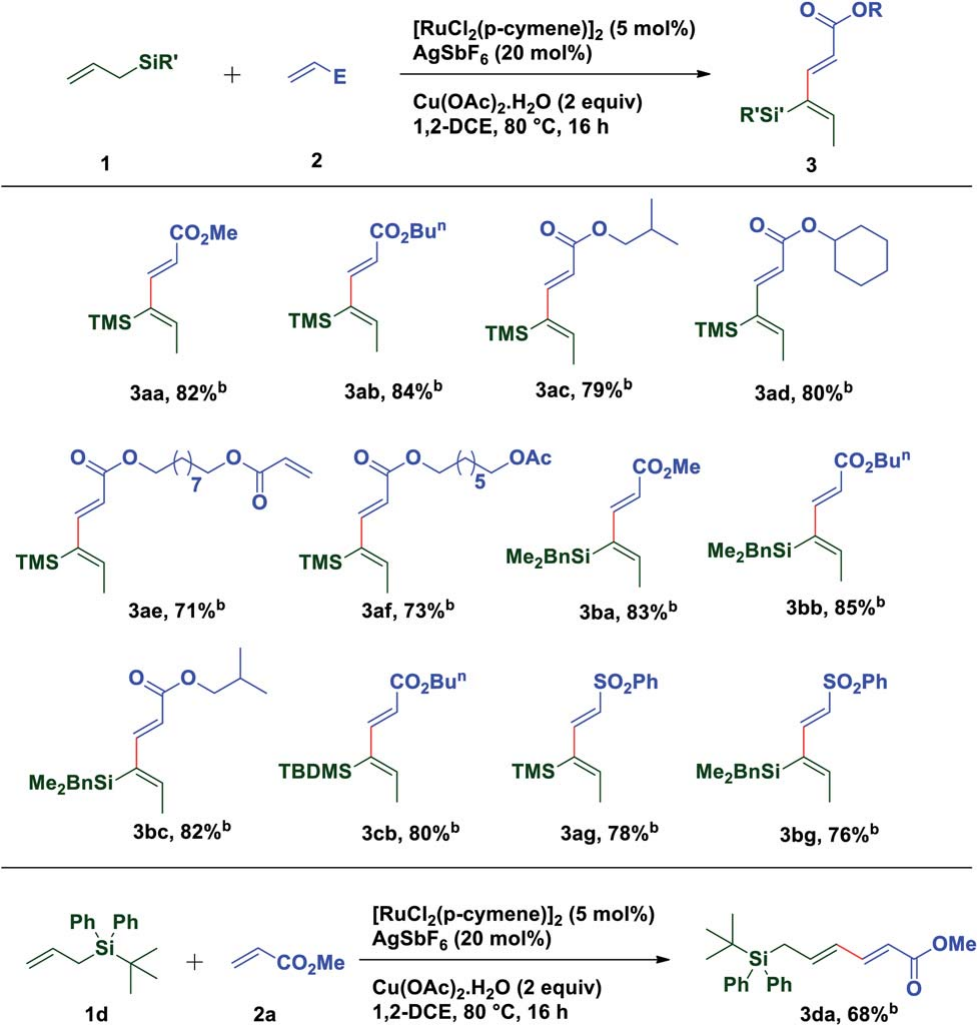

a Reaction conditions: **1** (0.24 mmol), **2** (0.2 mmol), [Ru(*p*-cymene)Cl_2_]_2_ (5 mol%), AgSbF_6_ (20 mol%) and Cu(OAc)_2_·H_2_O (2 equiv.) at 80 °C in 1,2-dichloroethane (2.0 mL), under argon, 16 h.

b Isolated yields are of product **3**. TMS – trimethylsilyl, TBDMS – tertiarybutyldimethyl silyl.

To extend the substrate scope of the reaction, we next examined the scope of allylesters by employing **2a** as the coupling partner. First, we carried out the coupling reaction between allyl ester derivative **4a** and methyl acrylate **2a** under standard conditions. To our delight, a single isomer of acetate substituted (2*E*,4*Z*)-1,3-diene **5aa** was isolated with a good yield (75%) (Table 3). This result may be extremely unusual due to the weak thermodynamic driving force for the double bond migration of allyl esters and tendency of many metal catalysts to insert themselves into the C(allyl)–O bond to form a stable carboxylate complex.^58^ Even for unsubstituted allyl esters very few reports of double bond migrations exist.^59–62^ It is worth mentioning that unlike the Tsuji–Trost reaction,^63–65^ the C(allyl)–O bond doesn't break to form the π-allyl palladium complex as an electrophile, instead it forms a nucleophilic π-allylruthenium complex (umpolung reactivity) keeping the acetate group intact, which further reacts with an electrophile. The stereochemistry of the diene was established by **1D** and **2D** spectroscopic analysis of the compound **5ga** and also by comparison of spectroscopic data with those of an authentic compound.^66^ Next we turned our attention to expand the scope of the coupling reaction between various acrylates and allyl esters. It was found that a variety of allyl esters bearing alkyl substituents on the carbonyl carbon could provide moderate to good yields of the corresponding stereodefined (2*E*,4*Z*)-1,3,4-trisubstituted 1,3-dienes successfully. As can be seen from Table 2, alkyl substituents (**4b–4d**) had little influence on the yields (65–75%). Gratifyingly, we noticed that the presence of a bulky substituent in **4** also showed its viability towards the coupling reaction, albeit with modest yields (**5ea** & **5fa**). Also, various acrylate derivatives reacted smoothly to generate the 1,3-dienes in excellent yield. A simple allyl acetate **4g** reacted with a series of different acrylates **2** to afford the desired products in good yields.

**Table 3:** Substrate scope for oxidative coupling of various allyl esters with different acrylates and vinyl sulfones^a^

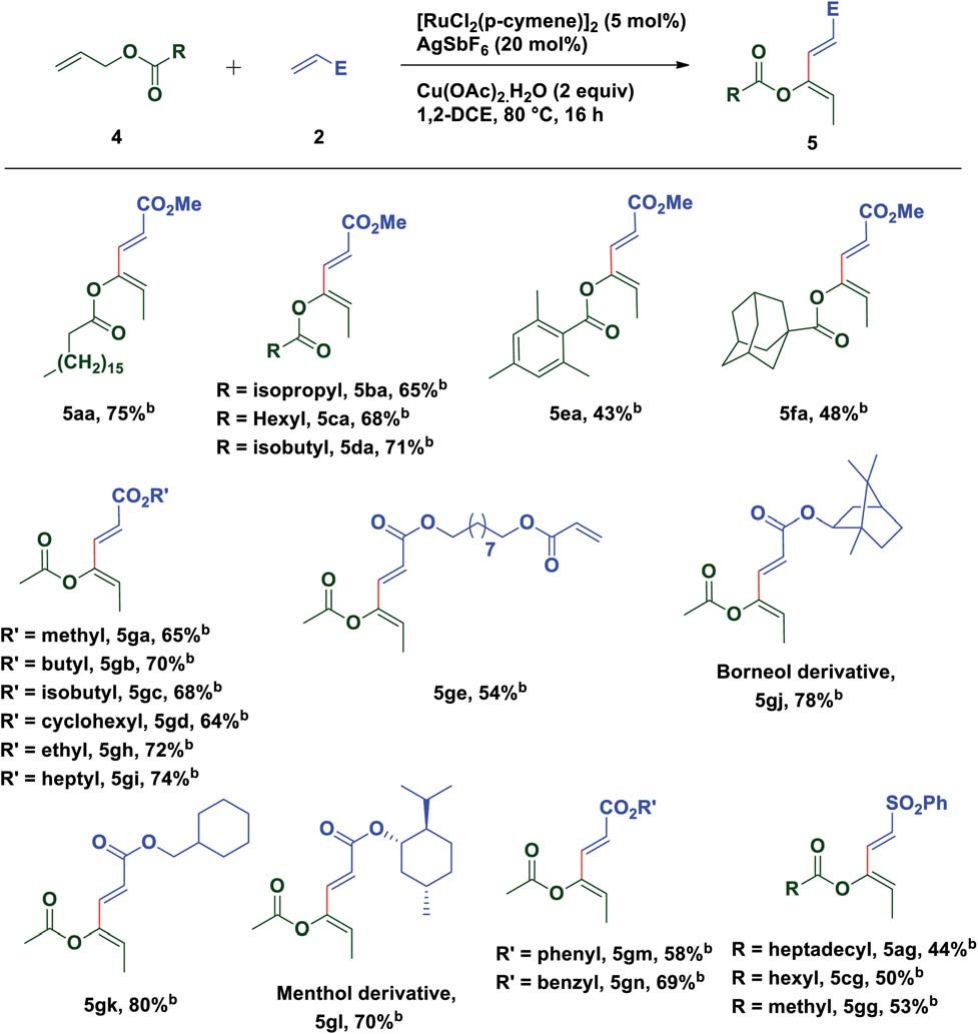

a Reaction conditions: **4** (0.24 mmol), **2** (0.2 mmol), [Ru(*p*-cymene)Cl_2_]_2_ (5 mol%), AgSbF_6_ (20 mol%) and Cu(OAc)_2_·H_2_O (2 equiv.) at 80 °C in 1,2-dichloroethane (2.0 mL), under argon, 16 h.

b Isolated yields are of product **5**.

Several acrylates such as methyl-, ethyl-, *n*-butyl-, isobutyl-, *n*-heptyl-, cyclohexylmethyl-, benzyl-, *etc.* were tested and good to very good yields of the products were obtained. Also, gram scale synthesis of **5gh** (1.35 g) by the reaction of acetate **4g** with **2h** gave identical results in terms of yield (69%) and diastereoselectivity, indicating the robustness and practicality of this method. Markedly, a C2-symmetric diacrylate (**2e**) also reacted with allyl acetate to form a mono-coupled product **5ge**, though in a somewhat lower yield. In contrast to the allyl esters, the coupling was not affected by the steric bulk of the acrylate substituents as depicted in Table 3. Even the borneol derivative **2j** and menthol derivative **2l**, which can offer considerable steric hindrance, were found to be equally effective in the formation of **5gj** and **5gl** in very good yields. A somewhat reduced yield of the product **5gm** was observed while using phenyl acrylate (**2m**) perhaps due to competitive reactive sites. Interestingly, the versatility of this methodology was not restricted only to acrylates, since phenyl vinyl sulfone was also found to be equally efficient for oxidative C–H functionalization with different allyl esters and a successful C–C coupling reaction was observed in each case with moderate yield and excellent diastereoselectivity.

Interestingly treatment of allylsilanes under standard reaction conditions in the absence of an acrylate coupling partner led to isomerization of various allylsilanes to afford corresponding vinylsilanes **6b–6e** in excellent yields (Scheme 2a). When allylsilane **1d** was subjected to isomerization in the presence of CD_3_CO_2_D, a significant amount of deuterium scrambling at the α-position (>20%) as well as at the methyl group (>45%) was observed in corresponding vinylsilane, indicating that the isomerization step is reversible and the rate determining step (Scheme 2b). It is also observed that when vinylsilane **6b** was made to react with methyl acrylate **2a** under standard conditions, it successfully underwent highly regioselective C–H activation and afforded coupling product **3b′a** in 80% yield (Scheme 2c). This result confirms that the coupling reaction proceeds *via* vinyl silane intermediate **6**.

**Scheme 2:**
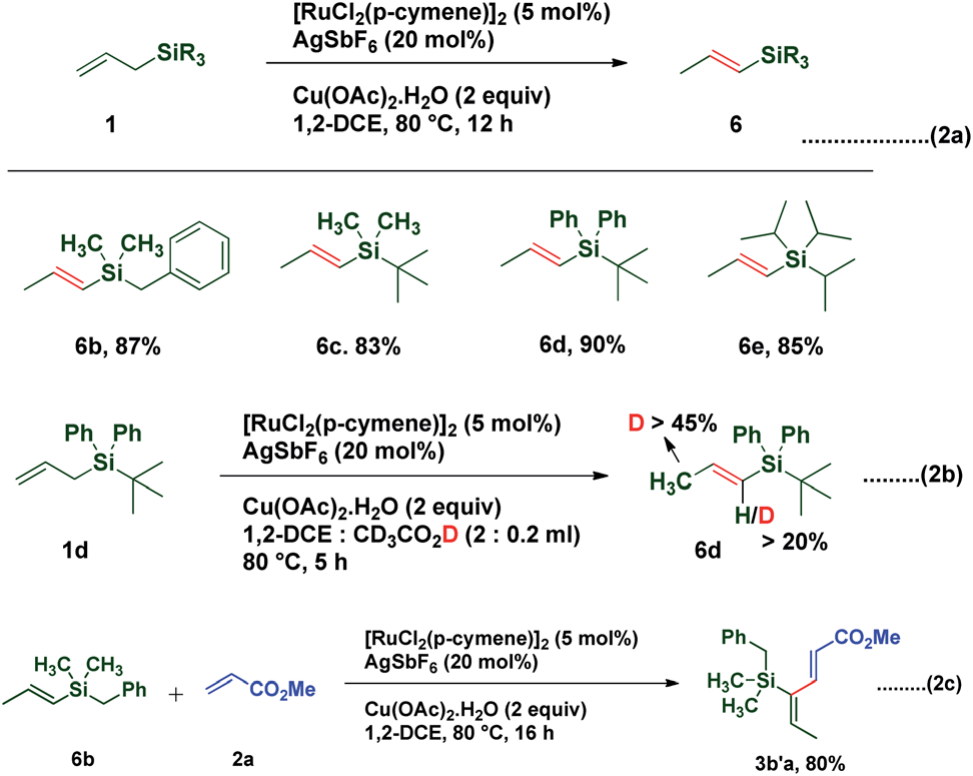
Isomerization of allylsilanes and deuterium study.

It is delightful to mention that diene **3aa** successfully underwent the Diels–Alder reaction with *N*-phenyl maleimide **7** in toluene at 80 °C, to afford single isomer **8** in 70% yield which ensures the pragmatism of the method (Scheme 3). The unique power of this ruthenium-catalyzed C–H functionalization strategy is illustrated by the late-stage diversification of the diene **5gh**, to a very reactive Michael acceptor **9** (conventional route for preparation of **9** requires *in situ* oxidation of α-hydroxyketones using 10 equiv. MnO_2_ followed by the Wittig reaction, which generates a superstoichiometric amount of phosphine waste)^67,68^*via* selective hydrolysis of the acetate group, which is useful in the synthesis of ester-thiol **10**,^69^ cyclohexenone **11** and polysubstituted piperidine **12** (ref. 70) (Scheme 4). Thus the Micheal acceptor **9** on reaction with thiophenol generated compound **10** in excellent yield and high regioselectivity. On the other hand compound **9** on reaction with heptanal in the presence of Hayashi–Jørgensen's catalyst afforded the Michael adduct **13** in 72% yield and excellent diastereoselectivity. Keto-aldehyde **13** was converted to highly substituted cyclohexenone **11** and piperidine **12**.

**Scheme 3:**
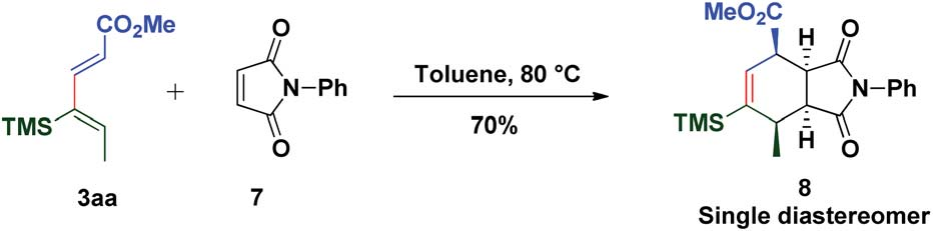
Application to the Diels–Alder reaction.

**Scheme 4:**
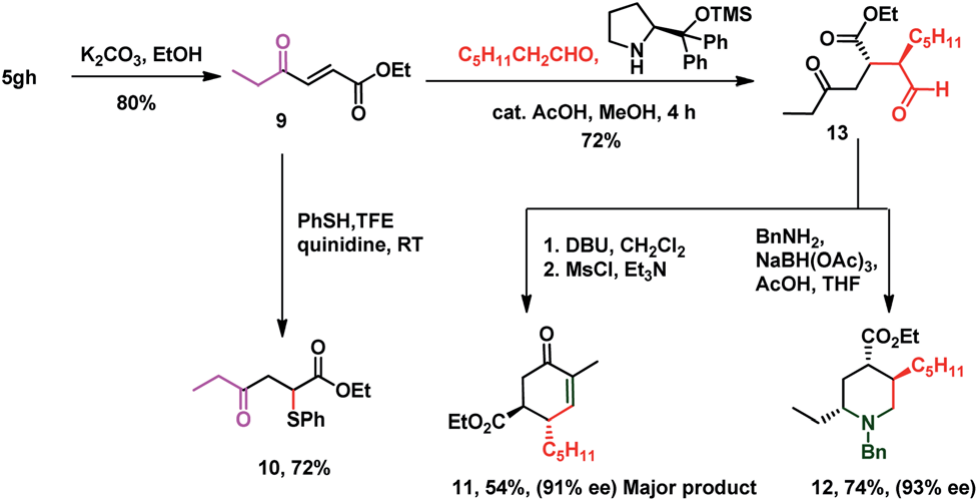
Application to the organocatalytic Michael addition reaction.

The potential of this Ru-catalysed reaction was further demonstrated by norpyrenophorin synthesis.^71–74^ Norpyrenophorin **14** is a synthetic 16-membered lactone which has essentially the same physiological activity as the natural fungicide pyrenophorin **15** and the antibiotic vermiculin **16**.^73^ A brief retrosynthetic analysis revealed that the dimeric macrocycle **14** could be dissected into monomer **17** which could be easily accessed from oxidative coupling of **2a** with **18** using the C–H activation reaction (Scheme 5). Ruthenium catalysed oxidative coupling of symmetric allylester **18** with **2a** generated the key intermediate **19** in 32% yield. Selective hydrolysis of acetyl enolate **19** was accomplished by the treatment with K_2_CO_3_ in methanol to provide **20** in 70% yield. In accordance with some previously reported studies, the active ketone functionality of **20** was protected as ketal by treatment with ethylene glycol in refluxing benzene to afford substrate **21**. Selective hydrolysis of acetate was achieved using Bu_2_SnO to generate alcohol **22** and finally, aluminium–selenium adduct mediated^72^ ring closing lactonization followed by deketalization ensured the completion of synthesis of **14** in 23% yield (two steps) (Scheme 6). A similar type of dimerization reaction could be envisioned to synthesize the natural products pyrenophorin **15** and vermiculin **16**.

**Scheme 5:**
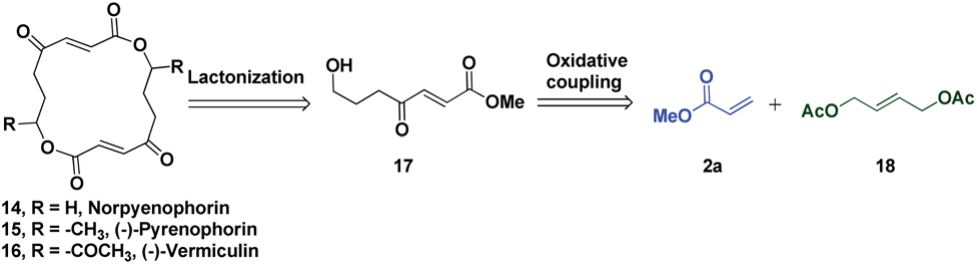
Retrosynthetic analysis of norpyrenophorin.

**Scheme 6:**
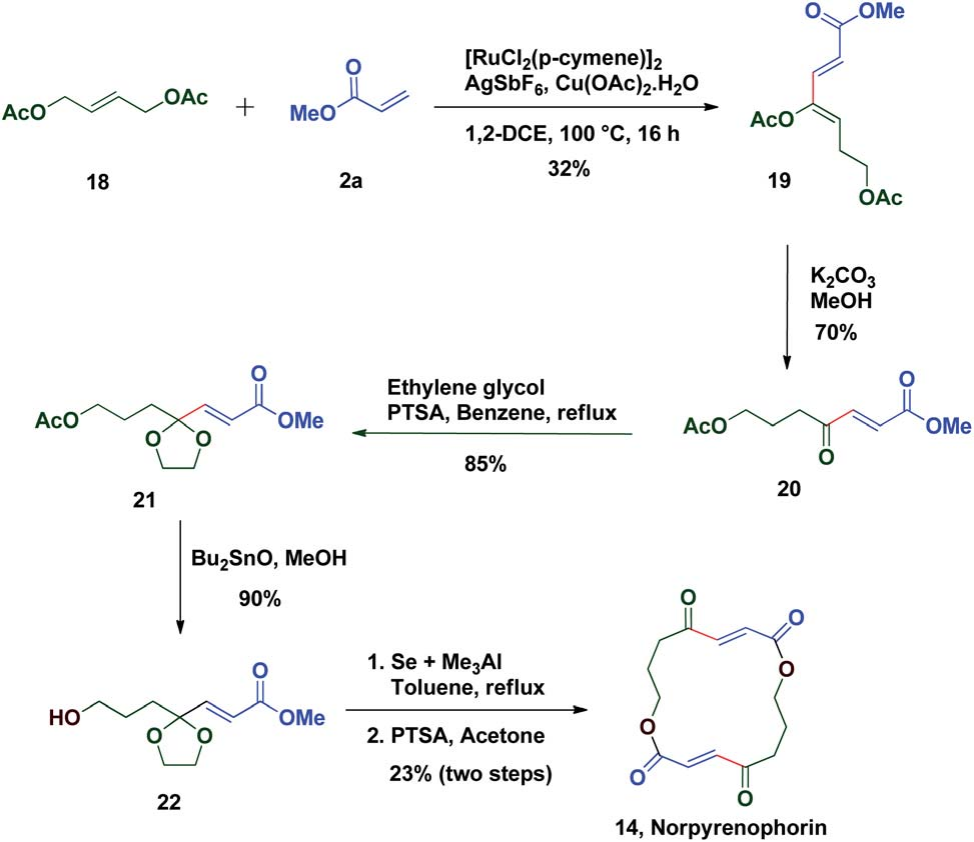
Synthesis of norpyrenophorin.

Based on the above result and previous report, a plausible mechanism for this oxidative coupling reaction is depicted in Scheme 7. The catalytic cycle is initiated by substrate **4g** coordination to *in situ* generated reactive cationic ruthenium complex **[Ru(OAc)L]+ A**, followed by weakly coordinating ester group directed C–H activation of allyl ester to give a π-allyl ruthenium intermediate **C**, which again would undergo isomerization to produce intermediate **D**. In the case of allyl silanes, an α-silyl effect might play an important role for the isomerisation of allylsilanes to vinylsilanes *via* the silylated allyl anion.^56^ Regioselective C–H activation of *in situ* generated vinyl acetate would give intermediate **E**. Induction of stability to the carbon–metal bond by the silyl group favours regioselective C–H insertion in the case of vinyl silanes.^57^ Coordination followed by 1,4-addition of vinyl ruthenium species to the activated olefins (acrylate, **2a**) would generate intermediate **G**, which would further undergo β-hydride elimination to provide a single isomer of 1,3-diene **H** and intermediate **I** could undergo reductive elimination followed by reoxidation of *in situ* forming Ru(0) species in the presence of Cu(OAc)_2_ to regenerate the reactive ruthenium(ii) complex **A** for the next catalytic cycle.

**Scheme 7:**
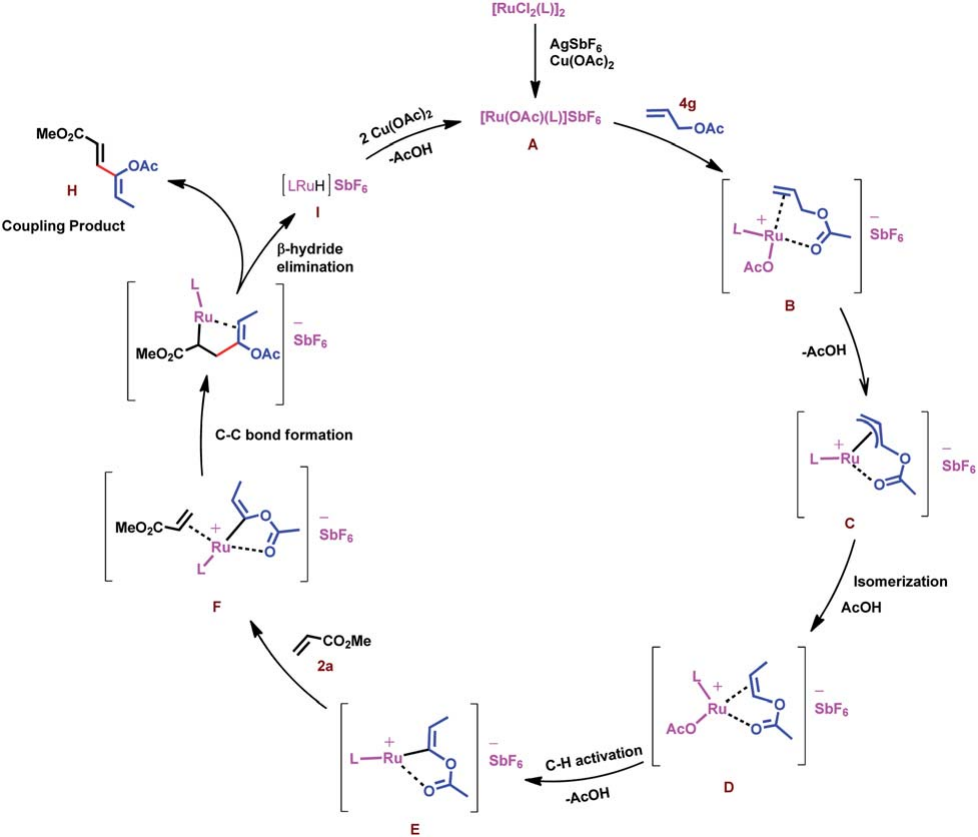
Plausible reaction mechanism.

## Conclusions

In summary, we have developed a ruthenium catalyzed efficient and straightforward method for the synthesis of highly stereodefined 1,3-dienes. Synthetic utility of this reaction towards the Diels–Alder reaction and diverse functional group transformations has been demonstrated. Finally, the scope of this reaction was further explored by the synthesis of norpyrenophorin in five steps.

## Author contributions

D. H. D. directed the project and wrote the manuscript. N. C. B. conducted most of the synthetic experiments and wrote the manuscript. S. D. and A. K. N. synthesized some of the silyl and acetate substituted dienes.

## Conflicts of interest

The authors declare no competing financial interest.

## Supplementary Material

SC-012-D0SC06845D-s001
