# Supplementary material for: Ruthenium-catalyzed formal sp3 C–H activation of allylsilanes/esters with olefins: efficient access to functionalized 1,3-dienes
Source: Chem Sci. 2021 Feb 2;12(12):4367–72. doi: 10.1039/d0sc06845d (PMC8179449; doi:10.1039/d0sc06845d)
Supplement: SC-012-D0SC06845D-s001 [file SC-012-D0SC06845D-s001.pdf]

Electronic Supplementary Material (ESI) for Chemical Science.
This journal is © The Royal Society of Chemistry 2021

Supplementary Information for

Ruthenium-Catalyzed Formal sp^3-H Activation of Allylsilanes/esters with Olefins: Efficient Access to functionalized 1,3-Dienes

Dattatraya H. Dethe*[a], Nagabhushana C B[a], Saikat Das[a,†], Appasaheb Nirpal[a†]

[a]Department of Chemistry, Indian Institute of Technology-Kanpur, Kanpur - 208016, India.
Tel: + 91-512-2596537, fax: + 91-512-2597436.

Table of Contents

1. General Information:…………………………………………………………………………………….3

2. Experimental procedures

2.1. General procedure for the oxidative coupling reaction of allyl esters with acrylates

2.1a. Examples with data ………..4 - 8

2.1b. Procedure and data for isomerization of Allylsilanes and Deuterated study………………………………………9

2.1c. Coupling reaction between vinylsilane **6b** and methyl acrylate **2a**………………………………….............9-10

2.1d. Procedure and data for Diels-Alder reaction, **8** ……………………………………………………………………10

2.2.Gram scale synthesis and its applications…………………………………………………………….10 - 12

2.2a. Synthesis of compound **5gh**

2.2b. Synthesis of compound **9**

2.2c. Synthesis of compound **13**

2.2d. Synthesis of compound **11**

2.2e. Synthesis of compound **12**

2.2f. Synthesis of compound **10**

2.3.Total synthesis of norpyrenophorin……………………………………………………………..12-14

2.3a. Synthesis of compound **19**

2.3b. Synthesis of compound **20**

2.3c. Synthesis of compound **21**

2.3d. Synthesis of compound **22**

2.3e. Synthesis of norpyenophorin **14**

3. NMR spectra……………………………………………………………………………………...15 - 68

4. References……………………………………………………………………………………………..69

1. General Information:

General Aspects: Experiments involving moisture and/or air sensitive components were performed in oven-dried glassware. Commercial solvents and reagents were used without further purification unless otherwise noted. All the chemicals were purchased commercially, and used without further purification. Yields refer to chromatographically pure compounds, unless otherwise stated. Reactions were monitored by thin-layer chromatography (TLC) carried out on 0.25 mm Merck silica gel plates (60F-254) using UV light as a visualizing agent and an p-anisaldehyde or ninhydrine stain, and heat as developing agents. Merck silica gel (particle size 100-200 and 230-400 mesh) was used for flash column chromatography. Neat compounds were used for record IR spectra. NMR spectra were recorded on either a Bruker Avance 400 (1H, 400 MHz; 13C, 100 MHz), Bruker Avance 500 (1H, 500 MHz; 13C, 125 MHz), or JEOL DELTA (ECX) 500 (1H, 500 MHz; 13C, 125 MHz). Mass spectrometric data were obtained using WATERS-Q-Tof-Premier-HAB213 and WATERS-QTof-Premier-ESI-MS instruments. Optical rotations were measured using a polarimeter (AUTOPOL II) at 30 °C. The following abbreviations were used to explain the multiplicities: s = singlet, d = doublet, t = triplet, q = quartet, spt= septet, dd = doublet of doublet, ddd = doublet of a doublet of a doublet, dt = doublet of a triplet, td = triplet of a doublet, m = multiplet, br = broad.

2. Experimental Procedures:

2.1. General procedure for the oxidative coupling reaction of allylsilanes/esters with activated olefins

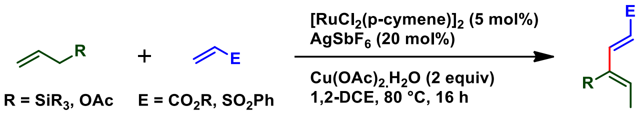

A 8 mL screw-cap vial was charged with $[RuCl_2(p\text{-cymene})]_2$ (6.1 mg, 0.01 mmol, 5.0 mol%), $Cu(OAc)_2{\cdot}H_2O$ (80 mg, 0.4 mmol, 2.0 equiv), $AgSbF_6$ (14 mg, 0.04 mmol, 20 mol%) and 1,2-dichloroethane (2.0 mL). Then acrylate/vinyl sulfone (0.2 mmol, 1.0 equiv) and allylsilane or allyl ester (0.24 mmol, 1.2 equiv) were added into the solution in sequence. The vial was sealed under N_2 and heated to 80°C with stirring for 16 h. After cooling down, the mixture was diluted with ethyl acetate, filtrated and concentrated to give the crude compound which was directly purified by column chromatography.

2.1a. Examples with data

Compound 3aa Following general procedure, **3aa** was obtained as a pale yellow oil (30:1::pet ether:EtOAc) (33 mg, 0.16 mmol, 82%).^{1}H NMR (400MHz, $CDCl_3$) δ 7.38 (d, *J* = 15.9 Hz, 1H), 6.56 (q, *J* = 7.2 Hz, 1H), 5.79 (d, *J* = 15.7 Hz, 1H), 3.70 (s, 3H), 1.88 (d, *J* = 7.2 Hz, 3H), 0.21 (s, 9H). ^{13}C NMR (125MHz, $CDCl_3$) δ 167.9, 152.2, 146.2, 138.3, 116.7, 51.4, 18.3, 0.5 (3C).HRMS (ESI) m/z calcd. for $C_{10}H_{18}O_2Si$ $[M+H]^+$199.1154; found 199.1158. IR (neat): v_{max}/cm^{-1}2970, 2950, 1743, 1712, 1643, 1618, 1432, 1300, 1115, 910, 840.

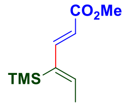

NOESY shows that H_a (0.21) and H_b (1.88) are correlated and also H_c (6.56) and H_d (7.38) are weakly correlated.

Compound 3ab Following general procedure, **3ab** was obtained as a pale yellow oil (30:1::pet ether:EtOAc) (40 mg, 0.168 mmol, 84%).^{1}H NMR (400MHz, $CDCl_3$) δ 7.38 (d, *J* = 15.9 Hz, 1H), 6.57 (q, *J* = 7.2 Hz, 1H), 5.79 (d, *J* = 15.5 Hz, 1H), 4.12 (t, *J* = 6.4 Hz, 2H), 1.89 (d, *J* = 7.2 Hz, 3H), 1.67 - 1.61 (m, 2H), 1.42 - 1.35 (m, 2H), 0.93 (t, *J* = 7.3 Hz, 3H), 0.22 (s, 9H). ^{13}C NMR (100MHz,$CDCl_3$) δ167.6, 151.8, 146.1, 138.3, 117.2, 64.1, 30.9, 19.3, 18.4, 13.8, 0.5(3C).HRMS (ESI) m/z calcd. for $C_{13}H_{24}O_2Si$ $[M+H]^+$241.1624; found 241.1626. IR (neat): v_{max}/cm^{-1}2975, 2948, 1749, 1716, 1640, 1619, 1430, 1298, 1120, 908, 838.

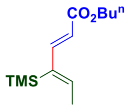

Compound 3ac Following general procedure, **3ac** was obtained as a pale yellow oil (30:1::pet ether:EtOAc) (38 mg, 0.158 mmol, 79%).^{1}H NMR (400MHz, $CDCl_3$) δ7.39 (d, *J* = 15.9 Hz, 1H), 6.58 (q, *J* = 7.2 Hz, 1H), 5.81 (d, *J* = 15.3 Hz, 1H), 3.91 (d, *J* = 6.4 Hz, 2H), 2.00 - 1.94 (m, 1H), 1.89 (d, *J* = 7.0 Hz, 3H), 0.94 (d, *J* = 6.5 Hz, 6H), 0.23 (s, 9H).^{13}C NMR (125MHz,$CDCl_3$) δ167.5, 151.8, 145.8, 138.4, 117.2, 70.4, 27.9, 19.2 (2C), 18.3, 0.5 (3C).HRMS (ESI) m/z calcd. for $C_{13}H_{24}O_2Si$ $[M+H]^+$241.1624; found 241.1626. IR (neat): v_{max}/cm^{-1}2978, 2949, 1748, 1716, 1642, 1622, 1431, 1297, 1122, 906, 839.

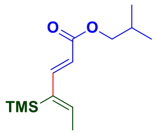

Compound 3ad Following general procedure, **3ad** was obtainedas a pale yellow oil (30:1::pet ether:EtOAc) (42 mg, 0.16 mmol, 80%).^{1}H NMR (400MHz, $CDCl_3$) δ 7.37 (d, *J* = 15.9 Hz, 1H), 6.57 (q, *J* = 7.2 Hz, 1H), 5.78 (d, *J* = 15.3 Hz, 1H), 4.83 - 4.76 (m, 1H), 1.88 (d, *J* = 7.3 Hz, 3H), 1.76 - 1.71 (m, 2H), 1.43 (d, *J* = 9.8 Hz, 2H), 1.38 - 1.34 (m, 2H), 0.22 (s, 9H). ^{13}C NMR (100MHz,$CDCl_3$) δ 166.9, 151.5, 145.9, 138.3, 117.7, 72.3, 31.8 (2C), 25.5, 23.9 (2C), 18.4, 0.5 (3C).HRMS (ESI) m/z calcd. for $C_{15}H_{26}O_2Si$ $[M+H]^+$267.1780; found 267.1783. IR (neat): v_{max}/cm^{-1}2983, 2953, 1743, 1708, 1636, 1620, 1439, 1293, 1115, 902, 832.

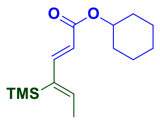

Compound 3ae Following general procedure, **3ae** was obtained as a pale yellow oil (30:1::pet ether:EtOAc) (54 mg, 0.150 mmol, 71%).^{1}H NMR (500MHz, $CDCl_3$) δ7.38 (d, *J* = 15.9 Hz, 1H), 6.57 (q, *J* = 7.2 Hz, 1H), 6.37 (d, *J* = 6.9 Hz, 1H), 6.11 (dd, *J* = 6.9, 5.2, Hz, 1H), 5.81 - 5.78 (m, 2H), 4.15 - 4.12 (m, 4H), 1.89 (d, *J* = 7.4 Hz, 3H), 1.65 (m, 6H), 1.37 - 1.33 (m, 8H), 0.23 (s, 9H). ^{13}C NMR (125MHz,$CDCl_3$) δ167.6, 166.4, 151.8, 146.0, 130.5, 128.7, 117.2, 64.8, 64.4, 29.5, 29.4, 29.3, 29.2, 28.8, 28.7, 26.0 (2C), 18.4, 0.5 (3C).HRMS (ESI) m/z calcd. for $C_{21}H_{36}O_4Si$ $[M+H]^+$381.2461; found 381.2460. IR (neat): v_{max}/cm^{-1}2945, 2860, 1769, 1720, 1658, 1629, 1432, 1370, 1259, 1120, 920, 850.

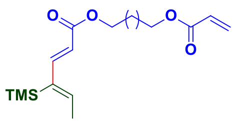

Compound 3af Following general procedure, **3af** was obtained as a pale yellow oil (30:1::pet ether:EtOAc) (50 mg, 0.146 mmol, 73%). ^{1}H NMR (400 MHz, $CDCl_3$) δ 7.38 (d, *J* = 15.9 Hz, 1H), 6.51 (q, *J* = 7.2 Hz, 1H), 5.79 (d, *J* = 15.7 Hz, 1H), 4.09 (t, *J* = 6.4 Hz, 2H), 4.03 (t, *J* = 6.4 Hz, 2H), 2.03 (s, 3H), 1.89 (d, *J* = 7.2 Hz, 3H), 1.65 - 1.59 (m, 6H), 1.34 (m, 4H), 0.22 (s, 9H). ^{13}C NMR (125 MHz, $CDCl_3$) δ

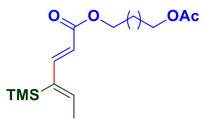

171.3, 167.5, 151.8, 146.0, 138.4, 117.2, 64.6, 64.2, 29.0, 28.7, 28.6, 25.9, 25.9, 21.1, 18.3, 0.5 (3C).HRMS (ESI) m/z calcd. for $C_{18}H_{32}O_4Si[M+H]^+$341.2148; found 341.2149. IR (neat): ν_{max}/cm^{-1}2935, 2850, 1723, 1638, 1463, 1415, 1370, 1293, 1125, 935. 860

Compound 3ba Following general procedure, **3ba** was obtained as a pale yellow oil (30:1::pet ether:EtOAc) (45 mg, 0.166 mmol, 83%).^{1}H NMR (500 MHz, $CDCl_3$) δ7.37 (d, *J* = 16.0 Hz, 1H), 7.19 (t, *J* = 7.4 Hz, 2H), 7.07 (t, *J* = 7.2 Hz, 1H), 6.98 (d, *J* = 6.9 Hz, 2H), 6.57 (q, *J* = 7.2 Hz, 1H), 5.76 (d, *J* = 16.0 Hz, 1H), 3.73 (s, 3H), 2.26 (s, 2H), 1.76 (d, *J* = 6.9 Hz, 3H), 0.20 (s, 6H).^{13}C NMR (100MHz, $CDCl_3$) δ167.8, 152.0, 147.0, 139.3, 136.7, 128.3 (2C), 128.2 (2C), 124.4, 117.0, 51.5, 26.1, 18.4, -1.3 (2C).HRMS (ESI) m/z calcd. for $C_{16}H_{22}O_2Si$ $[M+H]^+$275.1467; found 275.1469. IR (neat): ν_{max}/cm^{-1}3033, 2940, 1820, 1765, 1730, 1663, 1615, 1450, 1370, 1260, 1123, 960, 835.

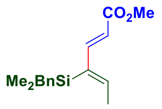

Compound 3bb Following general procedure, **3bb** was obtained as a pale yellow oil (30:1::pet ether:EtOAc) (54 mg, 0.17 mmol, 85%).^{1}H NMR (500 MHz, $CDCl_3$) δ7.35 (d, *J* = 15.5 Hz, 1H), 7.18 (t, *J* = 7.4 Hz, 2H), 7.06 (t, *J* = 7.2 Hz, 1H), 6.98 (d, *J* = 7.4 Hz, 2H), 6.59 (q, *J* = 6.9 Hz, 1H), 5.75 (d, *J* = 16.0 Hz, 1H), 4.12 (t, *J* = 6.4 Hz, 2H), 2.26 (s, 2H), 1.76 (d, *J* = 6.9 Hz, 3H), 1.65 (m, 2H), 1.40 (m, 2H), 0.93 (t, *J* = 7.2 Hz, 3H), 0.20 (s, 6H). ^{13}C NMR (125MHz, $CDCl_3$) δ167.4, 151.5, 146.5, 139.3, 136.8, 128.3 (2C), 128.2 (2C), 124.4, 117.6, 64.1, 30.9, 26.1, 19.3, 18.3, 13.8, -1.3 (2C).HRMS (ESI) m/z calcd. for $C_{19}H_{28}O_2Si$ $[M+H]^+$317.1937; found 317.1938. IR (neat): ν_{max}/cm^{-1}3025, 2930, 1835, 1740, 1715, 1670, 1630, 1425, 1360, 1225, 1115, 930, 840.

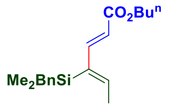

Compound 3bc Following general procedure, **3bc** was obtained as a pale yellow oil (30:1::pet ether:EtOAc) (54 mg, 0.164 mmol, 82%).^{1}H NMR (500 MHz, $CDCl_3$) δ7.36 (d, *J* = 16.0 Hz, 1H), 7.16 (t, *J* = 7.4 Hz 2H), 7.09 (t, *J* = 7.2 Hz, 1H), 6.98 (d, *J* = 7.4 Hz, 2H), 6.59 (q, *J* = 7.1 Hz, 1H), 5.75 (d, *J* = 16.0 Hz, 1H), 3.91 (d, *J* = 6.9 Hz, 2H), 2.26 (s, 2H), 1.96 (m, 1H), 1.77 (d, *J* = 6.9 Hz, 3H), 0.95 (d, *J* = 6.9 Hz, 6H), 0.20 (s, 6H). ^{13}C NMR (125MHz, $CDCl_3$) δ167.4, 151.5, 146.3, 139.3, 136.8, 128.3 (2C), 128.2(2C), 124.4, 117.6, 70.4, 27.9, 26.1, 19.2 (2C), 18.4, -1.3 (2C).HRMS (ESI) m/z calcd. for $C_{19}H_{28}O_2Si$ $[M+H]^+$317.1937; found 317.1938. IR (neat): ν_{max}/cm^{-1}3026, 2932, 1839, 1742, 1717, 1672, 1632, 1430, 1365, 1229, 1116, 938, 838.

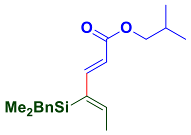

Compound 3cb Following general procedure, **3cb** was obtained as a pale yellow oil (30:1::pet ether:EtOAc) (45 mg, 0.16 mmol, 80%).^{1}H NMR (500 MHz, $CDCl_3$) δ7.43 (d, *J* = 15.9 Hz, 1H), 6.59 (q, *J* = 6.9 Hz, 1H), 5.79 (d, *J* = 15.4 Hz, 1H), 4.12 (t, *J* = 6.7 Hz, 2H), 1.88 (d, *J* = 7.2 Hz, 3H), 1.67 - 1.62 (m, 2H), 1.42 - 1.37 (m, 2H), 0.92 (t, *J* = 6.7 Hz, 3H), 0.91 (s, 9H), 0.20 (s, 6H). ^{13}C NMR (125MHz, $CDCl_3$) δ167.5, 152.8, 145.1, 136.8, 117.3, 64.1, 30.9, 27.0 (3C), 19.3, 19.1, 18.5, 13.8, -3.2 (2C).HRMS (ESI) m/z calcd. for $C_{16}H_{30}O_2Si$ $[M+H]^+$283.2093; found 283.2097. IR (neat): ν_{max}/cm^{-1}2965, 2939, 2872, 1773, 1720, 1643, 1630, 1315, 1173, 1050, 960, 855.

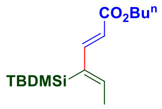

Compound 3ag Following general procedure, **3ag** was obtained as a pale yellow solid (15:1::pet ether:EtOAc) (43 mg, 0.154 mmol, 78%).^{1}H NMR (500 MHz, $CDCl_3$) δ7.87 (d, *J* = 7.4 Hz, 2H), 7.58 (t, *J* = 7.4 Hz, 1H), 7.51 (t, *J* = 7.4 Hz, 2H), 7.37 (d, *J* = 14.9 Hz, 1H), 6.60 - 6.54 (m, 1H), 6.24 (d, *J* = 14.9 Hz, 1H), 1.89 (d, *J* = 7.4 Hz, 3H), 0.19 (s, 9H).^{13}C NMR (125MHz, $CDCl_3$) δ 149.6, 147.6, 141.5, 136.7, 133.1, 129.3 (2C), 127.5 (2C), 125.9, 18.5, 0.3(3C).HRMS (ESI) m/z calcd. for $C_{14}H_{20}O_2SSi$ $[M+H]^+$281.1032; found 281.1035. IR (neat): ν_{max}/cm^{-1}3039, 2945, 1820, 1778, 1715, 1660, 1615, 1472, 1343, 1235, 1132, 1055, 960, 853.

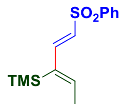

Compound 3bg Following general procedure, **3bg** was obtained as a pale yellow solid (15:1::pet ether:EtOAc) (54 mg, 0.152 mmol, 76%).^{1}H NMR (500 MHz, $CDCl_3$) δ7.82 (d, *J* = 7.4 Hz, 2H), 7.59 (t, *J* = 7.2 Hz, 1H), 7.52 (t, *J* = 7.4 Hz, 2H), 7.30 (d, *J* = 15.5 Hz, 1H), 7.10 (t, *J* = 7.4 Hz, 2H), 6.99 (t, *J* = 7.4 Hz, 1H), 6.90 (d, *J* = 6.9 Hz, 2H), 6.58 (q, *J* = 7.4 Hz, 1H), 6.05 (d, *J* = 14.9 Hz, 1H), 2.21 (s, 2H), 1.80 (d, *J* = 7.4 Hz, 3H), 0.18 (s, 6H). ^{13}C NMR (125 MHz, $CDCl_3$) δ149.3, 148.0, 141.3, 138.8, 135.3, 133.1, 129.3 (2C), 128.3, 128.2 (3C), 127.5(2C), 126.3, 124.5, 25.9, 18.5, -1.5 (2C).HRMS (ESI) m/z calcd. for $C_{20}H_{24}O_2SSi[M+H]^+$357.1345; found 357.1349. IR (neat): ν_{max}/cm^{-1}3050, 2958, 2936, 2915, 1816, 1780, 1740, 1615, 1602, 1520, 1459, 1368, 1232, 1128, 1059, 960, 856.

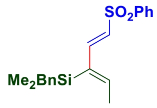

Compound 3da Following general procedure, **3da** was obtained as a pale yellow oil (30:1::pet ether:EtOAc)(49 mg, 0.136 mmol, 68%).^{1}H NMR (500 MHz, $CDCl_3$) δ7.55 (d, *J* = 8.0 Hz, 4H), 7.42 - 7.38 (m, 2H), 7.37 - 7.33 (m, 4H), 7.10 (dd, *J* = 10.9, 15.5 Hz, 1H), 6.18 - 6.08 (m, 1H), 5.95 (dd, *J* = 10.9, 14.9 Hz, 1H), 5.59 (d, *J* = 14.9 Hz, 1H), 3.68 (s, 3H), 2.30 (d, *J* = 8.0 Hz, 2H), 1.07 (s, 9H). ^{13}C NMR

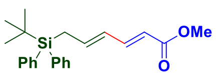

(125MHz, $CDCl_3$) δ168.0, 145.6, 141.9, 136.0 (4C), 133.7, 129.5 (2C), 128.5, 127.8 (5C), 117.1, 51.4, 27.9 (3C), 19.4, 18.7. HRMS (ESI) m/z calcd. for $C_{23}H_{28}O_2Si$ $[M+H]^+$ 365.1937; found 365.1938. IR (neat): $ν_{max}/cm^{-1}$3080, 3032, 2980, 2936, 2915, 1826, 1790, 1745, 1630, 1580, 1523, 1480, 1353, 1238, 1130, 1030, 953, 842.

Compoumd 5aa Following general procedure, **5aa** was obtained as a white solid (8.5:1.5::pet ether:EtOAc) (54 mg, 0.132 mmol, 66%). ^1H NMR (500 MHz, $CDCl_3$) δ 7.18 (d, J = 15.6 Hz, 1 H), 5.83 (q, J = 7.2 Hz, 1 H), 5.75 (d, J = 15.6 Hz, 1 H), 3.75 (s, 3 H), 2.54 (t, J = 7.6 Hz, 2 H), 1.77 - 1.72 (m, 2 H), 1.69 (d, J = 7.2 Hz, 3 H), 1.42 - 1.38 (m, 2 H), 1.27 (s, 26 H), 0.88 (t, J = 6.7 Hz, 3 H). ^{13}C NMR (125 MHz, $CDCl_3$): δ 170.9, 167.3, 145.7, 139.7, 126.3, 116.7, 52.0, 34.2, 32.3, 30.0(8C), 29.9, 29.8, 29.7, 29.5, 25.4, 23.0, 14.5, 12.6. HRMS (ESI) m/z calcd. for $C_{25}H_{44}O_4$ $[M+NH_4]^+$ 426.3583; found 426.3583. IR (neat): $ν_{max}/cm^{-1}$ 2925, 2854, 1764, 1725, 1652, 1628, 1435, 1306, 1167.

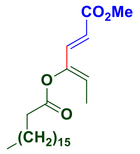

Compound 5ba Following general procedure, **5ba** was obtained as a pale yellow oil (8:2::pet ether:EtOAc) (28 mg, 0.13mmol, 65%). ^1H NMR (500 MHz, $CDCl_3$): δ 7.19 (d, J = 15.5 Hz, 1 H), 5.85 - 5.80 (m, 1 H), 5.73 (d, J = 15.5 Hz, 1 H), 3.75 (s, 3 H), 2.81 (spt, J = 7.0 Hz, 1 H), 1.68 (d, J = 7.2 Hz, 3 H), 1.33 (s, 3 H), 1.32 (s, 3 H). ^{13}C NMR (125 MHz, $CDCl_3$): δ 174.2, 167.3, 145.6, 139.7, 126.2, 116.5, 77.1, 52.0, 34.3, 19.5(2C), 12.4. HRMS (ESI) m/z calcd. for $C_{11}H_{16}O_4$ $[M+H]^+$213.1127; found 213.1134. IR (neat): $ν_{max}/cm^{-1}$ 2975, 2950, 1757, 1721, 1652, 1627, 1436, 1306, 1168.

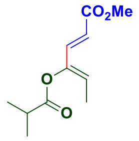

Compound 5ca Following general procedure, **5ca** was obtained as a white solid (8:2::pet ether:EtOAc) (35 mg, 0.136 mmol, 68%). ^1H NMR (400 MHz, $CDCl_3$): δ 7.17 (d, J = 15.3 Hz, 1 H), 5.82 (q, J = 7.3 Hz, 1 H), 5.74 (d, J = 15.9 Hz, 1 H), 3.74 (s, 3 H), 2.54 (t, J = 7.3 Hz, 2 H), 1.77 - 1.72 (m, 2 H), 1.68 (d, J = 7.2 Hz, 3 H), 1.43 - 1.37 (m, 2 H), 1.35 - 1.31 (m, 4 H), 0.89 (t, J = 6.7 Hz, 3 H). ^{13}C NMR (100 MHz, $CDCl_3$): δ 170.9, 167.3, 145.6, 139.7, 126.3, 116.6, 52.0, 34.2, 31.7, 29.2, 25.3, 22.8, 14.3, 12.6. HRMS (ESI) m/z calcd. for $C_{14}H_{23}O_4$ $[M+H]^+$ 255.1596; found 255.1591. IR (neat): $ν_{max}/cm^{-1}$ 2931, 2859, 1761, 1723, 1652, 1628, 1435, 1308, 1168.

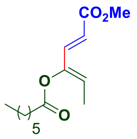

Compound 5da Following general procedure, **5da** was obtained as a pale yellow oil (8:2::pet ether:EtOAc) (32 mg, 0.142mmol, 71%). ^1H NMR (500 MHz, $CDCl_3$):δ 7.17 (d, J = 15.5 Hz, 1 H), 5.83 (q, J = 7.3 Hz, 1 H), 5.74 (d, J = 15.5 Hz, 1 H), 3.74 (s, 3 H), 2.42 (d, J = 7.2 Hz, 2 H), 2.27 - 2.18 (m, 1 H), 1.69 (d, J = 7.2 Hz, 3 H), 1.05 (s, 3 H), 1.04 (s, 3 H). ^{13}C NMR (125 MHz, $CDCl_3$): δ 169.9, 167.1, 145.4, 139.5, 126.0, 116.4, 51.8, 42.8, 25.7, 22.5(2C), 12.4. HRMS (ESI) m/z calcd. for $C_{12}H_{18}O_4$ $[M+H]^+$ 227.1283; found 227.1280. IR (neat): $ν_{max}/cm^{-1}$ 2959, 2874, 1760, 1723, 1653, 1628, 1466, 1307, 1169.

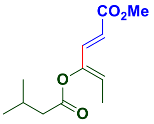

Compound 5ea Following general procedure, **5ea** was obtained as a white solid (8:2::pet ether:EtOAc) (25mg, 0.086mmol, 43%). ^1H NMR (400 MHz, $CDCl_3$): δ 7.27 (d, J = 15.9 Hz, 1 H), 6.94 (s, 2 H), 5.98 - 5.88 (m, 2 H), 3.75 (s, 3 H), 2.47 (s, 6 H), 2.33 (s, 3 H), 1.85 (d, J = 6.7 Hz, 3 H). ^{13}C NMR (125 MHz, $CDCl_3$): δ167.4, 166.8, 145.9, 140.7, 140.1, 136.9(2C), 129.5(2C), 129.1, 126.6, 117.0, 52.1, 21.5, 21.4(2C), 13.3. HRMS (ESI) m/z calcd. for $C_{17}H_{24}NO_4$ $[M+NH_4]^+$ 306.1705; found 306.1705. IR (neat): $ν_{max}/cm^{-1}$ 2923, 2852, 1725, 1612, 1651, 1435, 1307, 1234, 1165, 1052.

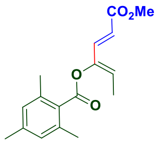

Compoumd 5fa Following general procedure, **5fa** was obtained as a white solid (8:2::pet ether:EtOAc) (29 mg, 0.096 mmol, 48%). ^1H NMR (400 MHz, $CDCl_3$): δ 7.18 (d, J = 15.9 Hz, 1 H), 5.81 (q, J = 7.3 Hz, 1 H), 5.70 (d, J = 15.9 Hz, 1 H), 3.75 (s, 3 H), 2.09 (br. s., 3 H), 2.07 (br, s, 6 H), 1.77 (br. s, 6 H), 1.66 (d, J = 6.7 Hz, 3 H). ^{13}C NMR (125 MHz, $CDCl_3$): δ 174.7, 167.4, 145.6, 139.9, 126.2, 116.4, 52.0, 41.6, 39.3(3C), 36.7(3C), 28.2(3C), 12. HRMS (ESI) m/z calcd. for $C_{18}H_{24}O_4$ $[M+NH_4]^+$ 322.2018; found 322.2019. IR (neat): $ν_{max}/cm^{-1}$ 2909, 2853, 1750, 1722, 1651, 1627, 1453, 1435, 1307, 1201, 1180, 1167.

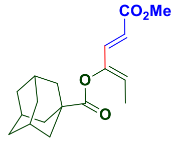

Compound 5ga Following general procedure, **5ga** was obtained as a pale yellow oil (8:2::pet ether:EtOAc) (24 mg, 0.13 mmol, 65%). ^1H NMR (500 MHz, $CDCl_3$): δ 7.17 (d, J = 15.6 Hz, 1 H), 5.82 (q, J = 7.2 Hz, 1 H), 5.77 (d, J = 15.5 Hz, 1 H), 3.75 (s, 3 H), 2.28 (s, 3 H), 1.70 (d, J = 7.2 Hz, 3 H). ^{13}C NMR (125 MHz, $CDCl_3$): δ 167.8, 167.0, 145.4, 139.2, 126.1, 116.5, 51.8, 20.4, 12.3. HRMS (ESI) m/z calcd. for $C_9H_{12}O_4$ $[M+H]^+$ 185.0814; found 185.0818. IR (neat): $ν_{max}/cm^{-1}$ 3058, 2953, 1762, 1723, 1653, 1628, 1436, 1310, 1200, 1171.

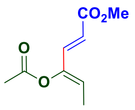

NOESY shows that H_a (5.80 - 5.86) and H_b (7.19 - 7.15) are correlated. H_c (5.76 - 5.78) and H_d (1.70) are weakly correlated.

Compound 5gb Following general procedure, **5gb** was obtained as a pale yellow oil (8:2::pet ether:EtOAc) (32 mg, 0.140 mmol, 70%). ^{1}H NMR (500 MHz, $CDCl_3$): δ 7.15 (d, *J* = 15.5 Hz, 1 H), 5.82 (q, *J* = 7.3 Hz, 1 H), 5.76 (d, *J* = 15.5 Hz, 1 H), 4.15 (t, *J* = 7.1 Hz, 3 H), 2.28 (s, 3 H), 1.69 (d, *J* = 7.3 Hz, 3 H), 1.66 - 1.63 (m, 2 H), 1.42 - 1.38 (m, 2 H), 0.94 (t, *J* = 7.1 Hz, 3 H).^{13}C NMR (125 MHz, $CDCl_3$): δ 168.1, 166.9, 145.8, 139.1, 126.0, 117.3, 64.8, 31.1, 20.6, 19.5, 14.0, 12.5. HRMS (ESI) m/z calcd. for $C_{12}H_{18}O_4$ $[M+H]^+$ 227.1283; found 227.1281. IR (neat): v_{max}/cm^{-1} 2960, 2934, 2874, 1765, 1715, 1653, 1627, 1304, 1196, 1170.

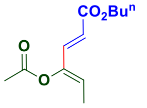

Compound 5gc Following general procedure, **5gc** was obtained as a pale yellow oil (8:2::pet ether:EtOAc) (31 mg, 0.136 mmol, 68%). ^{1}H NMR (500 MHz, $CDCl_3$): δ 7.16 (d, *J* = 15.5 Hz, 1 H), 5.83 (q, *J* = 7.3 Hz, 1 H), 5.77 (d, *J* = 15.6 Hz, 1 H), 3.93 (d, *J* = 6.6 Hz, 2 H), 2.29 (s, 3 H), 2.00 - 1.92 (m, 1 H), 1.70 (d, *J* = 7.2 Hz, 3 H), 0.95 (d, *J* = 6.7 Hz, 6 H). ^{13}C NMR (125 MHz, $CDCl_3$): δ 167.8, 166.6, 145.4, 138.8, 125.8, 116.9, 70.7, 27.8, 20.4, 19.1(2C), 12.2. HRMS (ESI) m/z calcd. for $C_{12}H_{18}O_4$ $[M+H]^+$ 227.1283; found 227.1282. IR (neat): v_{max}/cm^{-1} 2962, 2928, 2875, 2854, 1765, 1718, 1627, 1653, 1470, 1371, 1211, 1197, 1168.

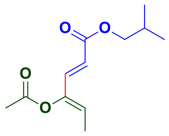

Compound 5gd Following general procedure, **5gd** was obtained as a white solid (8:2::pet ether:EtOAc) (32 mg, 0.128 mmol, 64%). ^{1}H NMR (500 MHz, $CDCl_3$): δ 7.15 (d, *J* = 15.6 Hz, 1 H), 5.82 (q, *J* = 7.3 Hz, 1 H), 5.74 (d, *J* = 15.5 Hz, 1 H), 4.85 - 4.78 (m, 1 H), 2.28 (s, 3 H), 1.89 - 1.84 (m, 2 H), 1.74 (m, 2 H), 1.69 (d, *J* = 7.3 Hz, 3 H), 1.58 - 1.52 (m, 1 H), 1.44 - 1.36 (m, 4 H), 1.29 - 1.24 (m, 1 H). ^{13}C NMR (100 MHz, $CDCl_3$): δ 167.8, 166.0, 145.5, 138.5, 125.6, 117.5, 72.9, 31.7(2C), 25.4, 23.8(2C), 20.4, 12.2. HRMS (ESI) m/z calcd. for $C_{14}H_{20}O_4$ $[M+H]^+$ 253.1440; found 253.1443. IR (neat): v_{max}/cm^{-1} 2936, 2859, 1764, 1713, 1653, 1626, 1450, 1371, 1259, 1197, 1173.

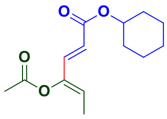

Compound 5gh Following general procedure, **5gh** was obtained as a pale yellow oil (8:2::pet ether:EtOAc) (29 mg, 0.144 mmol, 72%). ^{1}H NMR (500 MHz, $CDCl_3$): δ 7.16 (d, *J* = 15.5 Hz, 1 H), 5.85 - 5.80 (m, 1 H), 5.75 (d, *J* = 15.6 Hz, 1 H), 4.20 (q, *J* = 7.2 Hz, 2 H), 2.28 (s, 3 H), 1.70 (d, *J* = 7.2 Hz, 3 H), 1.29 (t, *J* = 7.1 Hz, 3 H). ^{13}C NMR (125 MHz, $CDCl_3$): δ 168.1, 166.8, 145.7, 139.2, 126.1, 117.2, 60.9, 20.7, 14.6, 12.5. HRMS (ESI) m/z calcd. for $C_{10}H_{14}O_4$ $[M+NH_4]^+$ 216.1236; found 216.1233. IR (neat): v_{max}/cm^{-1} 2982, 1764, 1714, 1652, 1628, 1369, 1305, 1197, 1175.

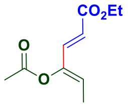

Compound 5gi Following general procedure, **5gi** was obtained as a white solid (8:2::pet ether:EtOAc) (40 mg, 0.148 mmol, 74%). ^{1}H NMR (500 MHz, $CDCl_3$): δ 7.16 (d, *J* = 15.5 Hz, 1 H), 5.83 (q, *J* = 7.3 Hz, 1 H), 5.76 (d, *J* = 15.6 Hz, 1 H), 4.14 (t, *J* = 6.8 Hz, 2 H), 2.29 (s, 3 H), 1.70 (d, *J* = 7.3 Hz, 3 H), 1.67 - 1.64 (m, 2 H), 1.32 (m, 8 H), 0.89 (t, *J* = 7.0 Hz, 3 H). ^{13}C NMR (100 MHz, $CDCl_3$): δ 168.1, 167.0, 145.7, 139.1, 126.1, 117.2, 65.1, 32.1, 29.3, 29.0, 26.2, 22.9, 20.7, 14.4, 12.5. HRMS (ESI) m/z calcd. for $C_{15}H_{24}O_4$ $[M+H]^+$ 269.1753; found 269.1757. IR (neat): v_{max}/cm^{-1}2925, 2855, 1727, 1651, 1627, 1453, 1435, 1307, 1261, 1180, 1167.

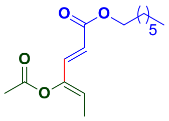

Compound 5ge Following general procedure, **5ge** was obtained as a white solid (8:2::pet ether:EtOAc) (40 mg, 0.108 mmol, 54%). ^{1}H NMR (400 MHz, $CDCl_3$): δ7.16 (d, *J* = 15.5 Hz, 1 H), 6.40 (dd, *J* = 1.4, 17.3 Hz, 1 H), 6.12 (dd, *J* = 10.5, 17.3 Hz, 1 H), 5.84 - 5.80 (m, 2 H), 5.75 (d, *J* = 15.5 Hz, 1 H), 4.17 - 4.12 (m, 4 H), 2.28 (s, 3 H), 1.70 (d, *J* = 7.3 Hz, 3 H), 1.68 - 1.63 (m, 4 H), 1.37 - 1.31 (m, 10 H). ^{13}C NMR (100 MHz, $CDCl_3$): δ 167.8, 166.6, 166.4, 145.4, 138.8, 130.5, 128.7, 125.8, 116.9, 64.7, 64.7, 29.4, 29.2(2C), 28.7, 28.6, 25.9(2C), 20.4, 12.2. HRMS (ESI) m/z calcd. for $C_{20}H_{30}O_6$ $[M+H]^+$ 367.2121; found 367.2121. IR (neat): v_{max}/cm^{-1} 2930,2856, 1725 (br), 1636, 1465, 1408, 1371, 1370, 1302, 1288, 1297, 1272, 1196.

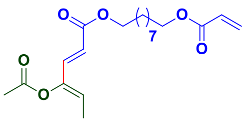

Compound 5gj Following general procedure, **5gj** was obtained as a white solid (8:2::pet ether:EtOAc) (48 mg, 0.156 mmol, 78%). ^{1}H NMR (400 MHz, $CDCl_3$): δ 7.15 (d, *J* = 15.3 Hz, 1 H), 5.84 (q, *J* = 7.3 Hz, 1 H), 5.78 (d, *J* = 15.9 Hz, 1 H), 4.97 - 4.92 (m, 1 H), 2.42 - 2.34 (m, 1 H), 2.31 (s, 3 H), 2.01 - 1.93 (m, 1 H), 1.77 (ddd, *J* = 4.0, 7.6, 11.6 Hz, 1 H), 1.70 (d, *J* = 7.3 Hz, 3 H), 1.37 - 1.22 (m, 3 H), 1.01 (dd, *J* = 3.4, 13.7 Hz, 1 H), 0.92 (s, 3 H), 0.88 (s, 3 H), 0.85 (s, 3 H). ^{13}C NMR (100 MHz, $CDCl_3$): δ 167.9, 166.9, 145.4, 138.5, 125.7, 117.4, 80.2, 48.9, 47.9, 44.9, 36.8, 28.0, 27.2, 20.4, 19.7, 18.9, 13.5, 12.2. HRMS (ESI) m/z calcd. for $C_{18}H_{26}O_4$ $[M+NH_4]^+$ 324.2175; found 324.2177. IR (neat): v_{max}/cm^{-1}2924, 2864, 1750, 1722, 1651, 1627, 1453, 1435, 1307, 1201, 1180, 1167. $[\alpha]_D^{30}$ = -20.68 (c = 0.81 in $CHCl_3$).

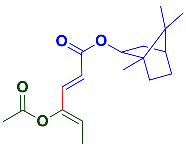

Compound 5gk Following general procedure, **5gk** was obtained as a white solid (8:2::pet ether:EtOAc) (42 mg, 0.160 mmol, 80%). ^{1}H NMR (400 MHz, $CDCl_3$): δ 7.16 (d, *J* = 15.9 Hz, 1 H), 5.83 (q, *J* = 7.1 Hz, 1 H), 5.76 (d, *J* = 15.3 Hz, 1 H), 3.96 (d, *J* = 6.7 Hz, 2 H), 2.29 (s, 3 H), 1.76 - 1.66 (m, 10 H), 1.28 - 1.21 (m, 3 H), 1.02 - 0.98 (m, 1 H). ^{13}C NMR (100 MHz, $CDCl_3$): δ 168.1, 167.0, 145.8, 139.1, 126.0, 117.3, 70.1, 37.5, 30.1(2C), 26.7, 26.0(2C), 20.7, 12.5. HRMS (ESI) m/z calcd. for $C_{15}H_{22}O_4$ $[M+Na]^+$ 289.1416; found 289.1418. IR (neat): ν_{max}/cm^{-1} 2928, 2854, 1766, 1715, 1651, 1627, 1450, 1370, 1305, 1288, 1196, 1168, 1023.

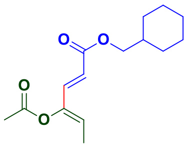

Compound 5gl Following general procedure, **5gl** was obtained as a white solid (8:2::pet ether:EtOAc) (43 mg, 0.14 mmol, 70%). ^{1}H NMR (400 MHz, $CDCl_3$): δ 7.14 (d, *J* = 15.9 Hz, 1 H), 5.82 (q, *J* = 7.1 Hz, 1 H), 5.75 (d, *J* = 15.3 Hz, 1 H), 4.76 (dt, *J* = 4.3, 10.7 Hz, 1 H), 2.29 (s, 3 H), 2.04 - 1.98 (m, 1 H), 1.91 - 1.84 (m, 1 H), 1.69 [m, 4 H (3H +1H)], 1.55 - 1.45 (m, 1 H), 1.43 - 1.36 (m, 1 H), 1.10 - 0.97 (m, 2 H), 0.92 - 0.88 (m, 8 H), 0.75 (d, *J* = 6.7 Hz, 3 H). ^{13}C NMR (100 MHz, $CDCl_3$): δ 168.2, 166.4, 145.8, 138.9, 126.0, 117.6, 74.6, 47.5, 41.3, 34.6, 31.7, 26.5, 23.7, 22.4, 21.1, 20.7, 16.6, 12.5. HRMS (ESI) m/z calcd. for $C_{18}H_{28}O_4$ $[M+Na]^+$ 331.1885; found 331.1889. IR (neat): ν_{max}/cm^{-1} 2956, 2929, 2870, 1766, 1711, 1651, 1626, 1455, 1370, 1302, 1288, 1196, 1171. $[\alpha]_D^{30}$ = -15.78 (c = 0.58 in $CHCl_3$).

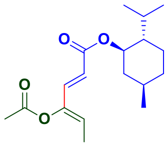

Compound 5gm Following general procedure, **5gm** was obtained as a brownish oil (8:2::pet ether:EtOAc) (29 mg, 0.116 mmol, 58%). ^{1}H NMR (500 MHz, $CDCl_3$): δ 7.41 - 7.38 (m, 2 H), 7.36 (d, *J* = 15.5 Hz, 1 H), 7.26 - 7.22 (m, 1 H), 7.12 (d, *J* = 7.6 Hz, 2 H), 5.98 - 5.90 (m, 2 H), 2.33 (s, 3 H), 1.75 (d, *J* = 7.2 Hz, 3 H). ^{13}C NMR (125 MHz, $CDCl_3$): δ 168.1, 165.3, 151.1, 145.8, 141.0, 129.8(2C), 127.4, 126.2, 121.9(2C), 116.4, 20.7, 12.7. HRMS (ESI) m/z calcd. for $C_{14}H_{14}O_4$ $[M+H]^+$ 247.0970; found 247.0973. IR (neat): ν_{max}/cm^{-1} 2924, 2854, 1762, 1731, 1649, 1626, 1493, 1371, 1290, 1192, 1141.

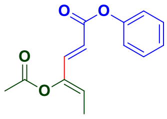

Compound 5gn Following general procedure, **5gn** was obtained as a white solid (8:2::pet ether:EtOAc) (36 mg, 0.138 mmol, 69%). ^{1}H NMR (500 MHz, $CDCl_3$): δ 7.39 - 7.35 (m, 5 H), 7.21 (d, *J* = 15.5 Hz, 1 H), 5.86 - 5.80 (m, 2 H), 5.20 (s, 2 H), 2.27 (s, 3 H), 1.70 (d, *J* = 7.2 Hz, 3 H). ^{13}C NMR (125 MHz, $CDCl_3$): δ 167.7, 166.4, 145.5, 139.5, 136.0, 128.7(2C), 128.4(2C), 128.4, 126.2, 116.6, 66.5, 20.4, 12.3. HRMS (ESI) m/z calcd. for $C_{15}H_{16}O_4$ $[M+NH_4]^+$ 278.1392; found 278.1394. IR (neat): ν_{max}/cm^{-1} 3034, 2938, 1815, 1760, 1723, 1650, 1626, 1455, 1372, 1264, 1198, 1165.

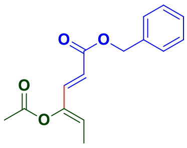

Compound 5ag Following general procedure, **5ag** was obtained as a white solid (8:2::pet ether:EtOAc) (43mg, 0.088 mmol, 44%). ^{1}H NMR (400 MHz, $CDCl_3$): δ 7.88 (d, *J* = 7.3 Hz, 2 H), 7.62 (t, *J* = 7.3 Hz, 1 H), 7.53 (t, *J* = 7.6 Hz, 2 H), 7.19 (d, *J* = 14.6 Hz, 1 H), 6.20 (d, *J* = 14.6 Hz, 1 H), 5.95 (q, *J* = 7.3 Hz, 1 H), 2.47 (t, *J* = 7.6 Hz, 2 H), 1.69 (d, *J* = 7.1 Hz 3 H), 1.68 - 1.63 (m, 2 H), 1.31 - 1.25 (m, 28 H), 0.88 (t, *J* = 6.7 Hz, 3 H). ^{13}C NMR (100 MHz, $CDCl_3$): δ 170.2, 143.6, 140.5, 137.8, 133.4, 129.3(2C), 128.8, 127.6(2C), 125.7, 33.8, 31.9, 29.7(6C), 29.6, 29.6, 29.4, 29.4, 29.2, 29.1, 24.9, 22.7, 14.2, 12.6. HRMS (ESI) m/z calcd. for $C_{29}H_{46}O_4S$ $[M+NH_4]^+$ 508.3461; found 508.3463. IR (neat): ν_{max}/cm^{-1} 2923, 2853, 1763, 1652, 1601, 1447, 1308, 1192, 1147, 1112, 1085.

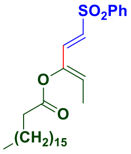

Compound 5cg Following general procedure, **5cg** was obtained as a white solid (8:2::pet ether:EtOAc) (34 mg, 0.10 mmol, 50%). ^{1}H NMR (500 MHz, $CDCl_3$): δ 7.88 (d, *J* = 8.2 Hz, 2 H), 7.62 (t, *J* = 7.4 Hz, 1 H), 7.54 (t, *J* = 7.9 Hz, 2 H), 7.19 (d, *J* = 14.9 Hz, 1 H), 6.20 (d, *J* = 14.9 Hz, 1 H), 5.95 (q, *J* = 7.3 Hz, 1 H), 2.48 (t, *J* = 7.6 Hz, 2 H), 1.70 (d, *J* = 7.2 Hz, 3 H), 1.68 - 1.64 (m, 2 H), 1.34 - 1.27 (m, 6 H), 0.90 - 0.86 (m, 3 H). ^{13}C NMR (100 MHz, $CDCl_3$): δ 170.2, 143.6, 140.5, 137.8, 133.5, 129.3(2C), 128.8, 127.6 (2C), 125.7, 33.8, 31.3, 28.8, 24.9, 22.4, 14.0, 12.6. HRMS (ESI) m/z calcd. for $C_{18}H_{24}O_4S$ $[M+NH_4]^+$ 354.1739; found 354.1737. IR (neat): ν_{max}/cm^{-1} 2923, 2853, 1721, 1650, 1661, 1447, 1308, 1147, 1084, 1025.

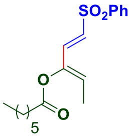

Compound 5gg Following general procedure, **5gg** was obtained as a white solid (8:2::pet ether:EtOAc) (28.19 mg, 0.106 mmol, 53%). ^{1}H NMR (500 MHz, $CDCl_3$): δ 7.88 (d, *J* = 8.2 Hz, 2 H), 7.62 (t, *J* = 7.4 Hz, 1 H), 7.54 (t, *J* = 7.9 Hz, 2 H), 7.19 (d, *J* = 14.9 Hz, 1 H), 6.23 (d, *J* = 14.9 Hz, 1 H), 5.96 (q, *J* = 7.2 Hz, 1 H), 2.23 (s, 3 H), 1.71 (d, *J* = 7.3 Hz, 3 H). ^{13}C NMR (125 MHz, $CDCl_3$): δ 167.6, 144.0, 140.8, 137.9, 133.8, 129.7(2C), 129.2, 128.0 (2C), 126.2, 20.6, 12.9. HRMS (ESI) m/z calcd. for $C_{13}H_{14}O_4S$ $[M+NH_4]^+$ 284.0957; found 284.0954. IR (neat): ν_{max}/cm^{-1} 2923, 2852, 1762, 1652, 1600, 1447, 1371, 1306, 1194, 1145, 1085, 1023.

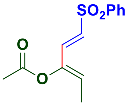

Procedure for Isomerization of Allylsilanes:

A 8 mL screw-cap vial was charged with $[RuCl_2(p\text{-cymene})]_2$ (6.1 mg, 0.01 mmol, 5.0 mol%), $Cu(OAc)_2{\cdot}H_2O$ (80 mg, 0.4 mmol, 2.0 equiv), $AgSbF_6$ (14 mg, 0.04 mmol, 20 mol%) and 1,2-dichloroethane (1.5 mL). Then allylsilane (0.2 mmol, 1.0 equiv) was added into the solution. The vial was sealed under N_2 and heated to 80°C with stirring for 12 h. After cooling down, the mixture was diluted with dichloromethane, filtered and concentrated to give the crude compound which was directly purified by column chromatography using only pentane as a eluent.

Compound 6b Following general procedure, **6b** was obtained as a colourless oil (using pentane as a eluent) (34 mg, 0.174 mmol, 87%). ^{1}H NMR (500 MHz, $CDCl_3$): δ 7.21 (t, *J* = 7.2 Hz, 2 H), 7.07 (t, *J* = 6.9 Hz, 1 H), 7.00 (d, *J* = 7.4 Hz, 2 H), 6.12 - 5.99 (m, 1 H), 5.63 (d, *J* = 20 Hz, 1 H), 2.11 (s, 2 H), 1.81 (d, *J* = 6.3 Hz, 3 H), 0.02 (s, 6 H). ^{13}C NMR (125 MHz, $CDCl_3$): δ 143.7, 140.6, 129.8, 128.6, 128.4, 124.2, 26.5, 23.0, -3.0. HRMS (ESI) m/z calcd. for $C_{12}H_{18}Si$ $[M+H]^+$ 191.1256; found 191.1258. IR (neat): ν_{max}/cm^{-1} 3078, 3022, 2988, 2953, 2849, 1799, 1618, 1599, 1492, 1247, 1055, 831, 697.

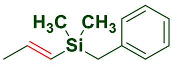

Compound 6c Following general procedure, **6c** was obtained as a colourless oil (using pentane as a eluent) (26 mg, 0.166 mmol, 83%). ^{1}H NMR (500 MHz, $CDCl_3$): δ 6.05 (qd, *J* = 6.0, 18.5 Hz, 1 H), 5.67 - 5.60 (m, 1 H), 1.85 - 1.79 (m, 3 H), 0.85 (s, 9 H), -0.01 (s, 7 H). ^{13}C NMR (125 MHz, $CDCl_3$): δ 143.3, 128.6, 26.5, 22.8, 16.5, -6.0. HRMS (ESI) m/z calcd. for $C_9H_{20}Si$ $[M+H]^+$ 157.1413; found 157.1416. IR (neat): ν_{max}/cm^{-1} 2923, 2852, 1600, 1371, 1234, 1050, 827, 720.

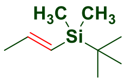

Compound 6d Following general procedure, **6d** was obtained as a colourless oil (using pentane as a eluent) (50 mg, 0.18 mmol, 90%). ^{1}H NMR (500 MHz, $CDCl_3$): 7.67 - 7.59 (m, 4 H), 7.43 - 7.31 (m, 6 H), 6.17 - 6.00 (m, 2 H), 1.93 (d, *J* = 4.6 Hz, 3 H), 1.09 (s, 9 H). ^{13}C NMR (100 MHz, $CDCl_3$): δ 147.8, 136.4, 135.2, 129.0, 127.6, 124.7, 27.8, 23.1, 18.2. HRMS (ESI) m/z calcd. for $C_{19}H_{24}Si$ $[M+H]^+$ 281.1726; found 281.1723. IR (neat): ν_{max}/cm^{-1} 3082, 3035, 2992, 2949, 2901, 1601, 1592, 1567, 1492, 1282, 1012 845, 693.

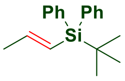

Compound 6e Following general procedure, **6e** was obtained as a colourless oil (using pentane as a eluent) (34 mg, 0.17 mmol, 90%). ^{1}H NMR (500 MHz, $CDCl_3$): 7.67 - 7.59 (m, 4 H), 7.43 - 7.31 (m, 6 H), 6.17 - 6.00 (m, 2 H), 1.93 (d, *J* = 4.6 Hz, 3 H), 1.09 (s, 9 H). ^{13}C NMR (100 MHz, $CDCl_3$): δ 147.8, 136.4, 135.2, 129.0, 127.6, 124.7, 27.8, 23.1, 18.2. HRMS (ESI) m/z calcd. for $C_{12}H_{26}Si$ $[M+H]^+$ 199.1882; found 199.1886. IR (neat): ν_{max}/cm^{-1} 2946, 2893, 1593, 1299, 1203, 1001, 819, 725.

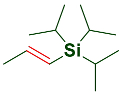

Procedure for Deuterium study:

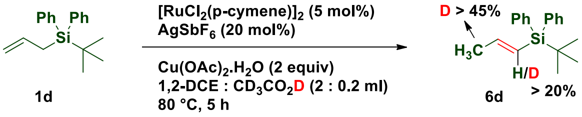

A 8 mL screw-cap vial was charged with $[RuCl_2(p\text{-cymene})]_2$ (11 mg, 0.017 mmol, 5.0 mol%), $Cu(OAc)_2{\cdot}H_2O$ (143 mg, 0.71 mmol, 2.0 equiv), $AgSbF_6$ (24.5 mg, 0.07 mmol, 20 mol%), 1,2-dichloroethane (2 mL) and CD_3CO_2D (0.2 ml). Then Diphenyltertiarybutyl allylsilane **1d** (0.2 mmol, 1.0 equiv) was added into the solution. The vial was sealed under N_2 and heated to 80°C with stirring for 5 h. After cooling down, the mixture was diluted with dichloromethane, filtered and concentrated to give the crude compound which was directly purified by column chromatography using only pentane as a eluent to afford compound **6d**. It was found that deuterium scrambling at the α-position (> 20%) as well as at the methyl group (> 45%) in vinylsilane.

Coupling reaction between vinylsilane 6b and methyl acrylate 2a:

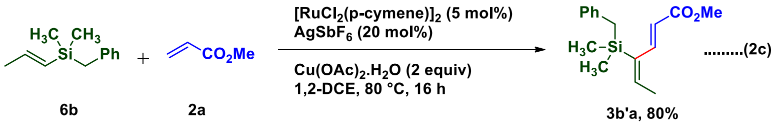

A 8 mL screw-cap vial was charged with $[RuCl_2(p\text{-cymene})]_2$ (11 mg, 0.017 mmol, 5.0 mol%), $Cu(OAc)_2{\cdot}H_2O$ (143 mg, 0.71 mmol, 2.0 equiv), $AgSbF_6$ (24.5 mg, 0.07 mmol, 20 mol%) and 1,2-dichloroethane (2 mL) Then vinylsilane **6b** (70 mg,

0.4 mmol, 1.2 equiv) and methyl acrylate **2a** (30 mg, 0.35 mmol, 1.0 equiv) were added into the solution. The vial was sealed under N_2 and heated to 80°C with stirring for 16 h. After cooling down, the mixture was diluted with dichloromethane, filtered and concentrated to give the crude compound which was directly purified by column chromatography using 30:1 pet::ether:EtOAc as a eluent to afford compound **3b'a** (77 mg, 0.28 mmol, 80%).

^{1}H NMR (400 MHz, $CDCl_3$) δ 7.36 (d, *J* = 15.9 Hz, 1 H), 7.18 (t, *J* = 7.6 Hz, 2 H), 7.06 (t, *J* = 7.3 Hz, 1 H), 6.96 (d, *J* = 6.7 Hz, 2 H), 6.68 - 6.47 (m, 1 H), 5.75 (d, *J* = 15.9 Hz, 1 H), 3.72 (s, 3 H), 2.25 (s, 2 H), 1.75 (d, *J* = 7.3 Hz, 3 H), 0.19 (s, 6 H). ^{13}C NMR (125 MHz, $CDCl_3$) δ 167.8, 151.9, 146.8, 139.3, 136.8, 128.3, 128.3, 124.4, 117.1, 51.5, 26.1, 18.4, -1.3.

Procedure for Diels-Alder reaction:

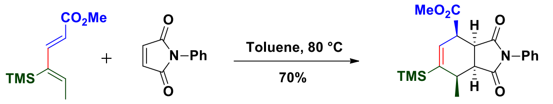

3aa 7 8
Single diastereomer

Compound **3aa** (100 mg, 0.5 mmol, 1.0 equiv.,) and N-phenyl maleimide **7** (88 mg, 0.5 mmol, 1.0 equiv.,) were taken in 8 mL screw-cap vial and Toluene (1.5 ml) was added. Then reaction mixture was heated at 80 °C for 15 h. After cooling down, Toluene was removed under reduced pressure and crude compound was directly purified by column chromatography using 0 - 20% EtOAc in Pet ether as a eluent to afford single diastereomer **8** (130 mg, 0.35 mmol) in 70% yield.

Compound 8:
^{1}H NMR (500 MHz, $CDCl_3$): δ 7.44 - 7.38 (m, 2 H), 7.36 - 7.31 (m, 1 H), 7.19 - 7.13 (m, 2 H), 6.67 (dd, *J* = 2.6, 3.7 Hz, 1 H), 3.80 (s, 3 H), 3.77 - 3.69 (m, 1 H), 3.33 - 3.25 (m, 1 H), 3.23 - 3.15 (m, 1 H), 2.67 (t, *J* = 6.9 Hz, 1 H), 1.56 (d, *J* = 7.4 Hz, 3 H), 0.13 (s, 9 H). ^{13}C NMR (125 MHz, $CDCl_3$): δ 176.5, 176.4, 171.4, 148.0, 135.3, 131.8, 129.1, 128.6, 126.5, 52.4, 44.6, 42.9, 41.1, 35.6, 16.7, -0.1. HRMS (ESI) m/z calcd. for $C_{20}H_{25}NO_4Si$ $[M+H]^+$ 372.1631; found 372.1634. IR (neat): ν_{max}/cm^{-1} 2951, 2922, 2849, 1739, 1709, 1499, 1455, 1382, 1276, 1247, 1201, 1036, 923, 834, 690.

2.2. Gram scale synthesis and its applications

2.2a. Synthesis of compound 3gb

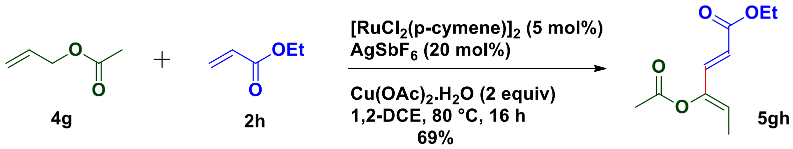

By following the general procedure, A 100 mL seal tube was charged with $[RuCl_2(p\text{-cymene})]_2$ (307 mg, 0.5 mmol, 5.0 mol%), $Cu(OAc)_2{\cdot}H_2O$ (4.0 g, 20 mmol, 2.0 equiv), $AgSbF_6$ (685 mg, 2.0 mmol, 20 mol%) and 1,2-dichloroethane (30 mL). Then ethyl acrylate **2h** (1.0g, 1.06ml, 10 mmol, 1.0 equiv) and allyl acetate **4g** (1.2g, 1.3ml, 12mmol, 1.2equiv) were added into the solution in sequence. Then vial was sealed under N_2 and heated to 80 °C with stirring for 16 h. After cooling down, the mixture was diluted with ethyl acetate, filtrated and concentrated to give the crude compound which was directly purified by column chromatography (8:2 petroleum ether/ethyl acetate) to give **5gh** as a colourless oil (1.36 g, 6.9 mmol, 69%).

2.2b. Synthesis of compound 9

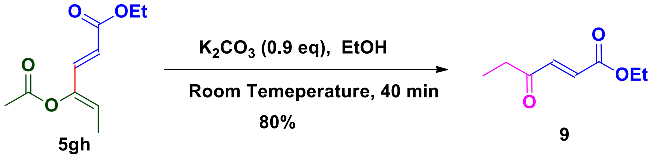

To a solution of **5gh** (1.3 g, 6.5 mmol, 1.0 equiv) in ethanol (15 ml), added potassium carbonate (815 mg, 5.9 mmol, 0.9 equiv) in four portions over 5 min and reaction mixture was allowed to stirred for 40 minutes at room temperature. Then reaction mixture was quenched with water and extracted with ethyl acetate (15 ml X 3), washed with brine, dried over anhydrous Na_2SO_4, concentrated *in vacuo* and purified by column chromatography (7:3 petroleum ether/ethyl acetate) furnishing the enone **9** (816 mg, 5.2 mmol, 80%) as a yellow liquid.

(E)-ethyl 4-oxohex-2-enoate (9)

^{1}H NMR (400 MHz, $CDCl_3$): δ 7.06 (d, *J* = 16.5 Hz, 1 H), 6.67 (d, *J* = 15.9 Hz, 1 H), 4.26 (q, *J* = 7.3 Hz, 2 H), 2.67 (q, *J* = 7.3 Hz, 2 H), 1.32 (t, *J* = 7.1 Hz, 3 H), 1.13 (t, *J* = 7.3 Hz, 3 H). ^{13}C NMR (101 MHz, $CDCl_3$): δ 200.2, 165.6, 139.2, 130.6, 61.4, 34.8, 14.1, 7.6. HRMS (ESI) m/z calcd. for $C_8H_{12}O_3$ $[M+NH_4]^+$ 174.1130; found 174.1139. IR (neat): ν_{max}/cm^{-1} 2982, 2939, 1727, 1704, 1688, 1641, 1304, 1185.

2.2c.Synthesis of compound 13[1]

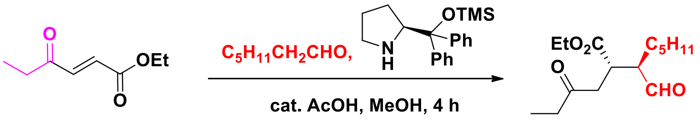

9 72% 13

To a suspension of catalyst (52 mg, 0.16 mmol, 0.1 equiv), enone **9** (250 mg, 1.60 mmol, 1 equiv) and acetic acid (10 µl, 0.16 mmol, 0.1 equiv) in methanol (7 ml) was added heptaldehyde (0.45 ml, 365 mg, 3.2 mmol, 2 equiv) at 0 °C. The reaction mixture was stirred for 1 h at the same temperature and then warmed to room temperature for 3 h. The solution was partitioned between ethyl acetate and saturated $NaHCO_3$. The organic phase was separated and aqueous phase was extracted with ethyl acetate. The combined organic layers were dried over Na_2SO_4 and concentrated. Purification of the residue by flash column chromatography (silica gel) eluting with 10:1 petroleum ether/ethyl acetate afforded the Michael adduct **13** (310 mg, 1.152 mmol, 72%) as a pale-yellow liquid.

(2R,3S)-ethyl 3-formyl-2-(2-oxobutyl)octanoate (13)

^{1}H NMR (400 MHz, $CDCl_3$): δ 9.61 (s, 1 H), 4.13 (q, *J* = 7.3 Hz, 2 H), 3.37 - 3.28 (m, 1 H), 2.97 (dd, *J* = 9.8, 17.7 Hz, 1 H), 2.65 - 2.57 (m, 1 H), 2.51 - 2.44 (m, 2 H), 2.42 - 2.36 (m, 1 H), 1.33 - 1.26 (m, 8 H), 1.23 (s, 3 H), 1.05 (t, *J* = 7.3 Hz, 3 H), 0.87 (s, 3 H). ^{13}C NMR (101 MHz, $CDCl_3$): δ 209.0, 202.7, 173.1, 61.1, 52.7, 40.7, 39.6, 36.1, 31.7, 27.1, 26.2, 22.4, 14.1, 13.9, 7.7. HRMS (ESI) m/z calcd. for $C_{15}H_{26}O_4$ $[M-H]^-$ 269.1757; found 269.1753. IR (neat): ν_{max}/cm^{-1} 2957, 2932, 2860, 1725 (br), 1461, 1376, 1212, 1179, 1115. $[\alpha]_D^{30}$ = +12.68 (c = 1.6 in $CHCl_3$).

2.2d. Synthesis of compound 11[1]

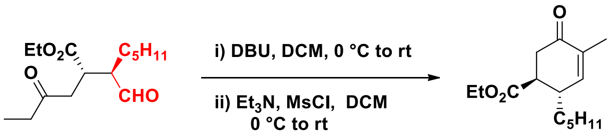

13 60% 11

To a solution of Michael adduct **13** (100 mg, 0.37 mmol) in 4 mL of DCM, was added DBU (113 ml, 0.74 mmol, 2.0 equiv) at 0 °C. After it was stirred for 1 h at 0 °C, the solution was warmed to room temperature and stirred for 2h. The reaction mixture was partitioned between ethyl acetate and brine. The organic phase was separated and aqueous phase was extracted with ethyl acetate. The combined organic layers were dried over Na_2SO_4 and concentrated. The residue was purified by flash column chromatography (silicagel, 10:1 petroleum ether/ethyl acetate as eluent) to give the corresponding cyclohexanone.

To a solution of aldol reaction product (70 mg, 0.25 mmol) in DCM (3 mL) were added MsCl (60 µl, 0.77 mmol, 3.0 equiv) and Et_3N (0.19 ml, 1.5 mmol, 6.0 equiv) at 0 °C. After the reaction mixture was stirred for 1h at 0 °C, and 6 h at 25 °C, it was partitioned between ethyl acetate and brine. The organic phase was separated and aqueous phase was extracted with ethyl acetate. The combined organic layers were dried over Na_2SO_4 and concentrated. The residual oil was purified viaflash column chromatography (silica gel, 20:1 petroleum ether/ethyl acetate as eluent) to afford the corresponding cyclohexenone **11** (38mg, 0.15mmol, 60%).

(1S, 2S)-ethyl 4-methyl-5-oxo-2-pentylcyclohex-3-enecarboxylate (11)

^{1}H NMR (400 MHz, $CDCl_3$): δ 6.57 (br. s., 1 H), 4.15 (q, *J* = 6.9 Hz, 2 H), 2.86 - 2.75 (m, 2 H), 2.64 - 2.59 (m, 2 H), 1.77 (s, 3 H), 1.53 - 1.35 (m, 4 H), 1.30 (br. s., 4 H), 1.25 (d, *J* = 7.3 Hz, 3 H), 0.89 (t, *J* = 6.7 Hz, 3 H). ^{13}C NMR (101 MHz, $CDCl_3$): δ 197.4, 173.8, 147.8, 135.0, 61.2, 46.1, 39.3, 38.5, 33.5, 32.1, 26.5, 22.8, 16.1, 14.5, 14.3. HRMS (ESI) m/z calcd. for $C_{15}H_{24}O_3$ $[M+H]^+$ 253.1804; found 253.1806. IR (neat): ν_{max}/cm^{-1} 2957, 2928, 2859, 1733, 1682, 1454, 1377, 1276, 1175, 1032. $[\alpha]_D^{30}$ = +68.6 (c = 0.70 in $CHCl_3$).

2.2e. Synthesis of compound 12[1]

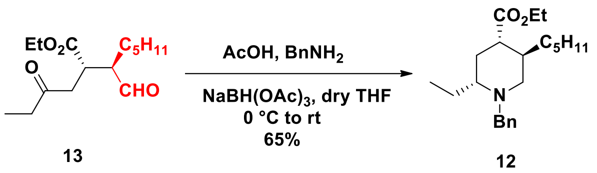

The Michael adduct **13** (100 mg, 0.37 mmol) was dissolved in 3 mL THF. To this solution NaBH(OAc)3 (235 mg, 1.11 mmol, 3.0 equiv), AcOH (23 µl, 0.37 mmol, 1.0 equiv) and $BnNH_2$ (60 mg, 61 µl, 0.55 mmol, 1.5 equiv) were added at 0 °C. After it was stirred for 1 h at 0 °C, the solution was warmed to rt, and stirred for 2.5hours. Then mixture was partitioned between ethyl acetate and saturated $NaHCO_3$. The organic phase was separated and aqueous phase was extracted with ethyl acetate. The combined organic layers were dried over Na_2SO_4 and concentrated. Purification of the residue by flash column chromatography (silica gel, 20:1 petroleum ether/ethyl acetate as eluent) afforded the corresponding piperidine **12** (82.5 mg, 0.24 mmol, 65%).

(2R,4S,5R)-ethyl 1-benzyl-2-ethyl-5-pentylpiperidine-4-carboxylate (12)

^{1}H NMR (400 MHz, $CDCl_3$): δ 7.32 - 7.28 (m, 3 H), 7.25 (br..s, 2 H), 4.17 - 4.11 (m, 2 H), 2.84 (d, *J* = 13.6 Hz, 1 H), 2.79 (dd, *J* = 2.7, 11.8 Hz, 1 H), 2.51 (td, *J* = 4.1, 13.1 Hz, 1 H), 2.12 - 2.07 (m, 1 H), 1.86 - 1.75 (m, 3 H), 1.73 - 1.61 (m, 4 H), 1.25 - 1.22 (m, 5 H), 1.20 - 1.15 (m, 3 H), 0.98 - 0.94 (m, 3 H), 0.92 - 0.86 (m, 3 H), 0.81 (t, *J* = 6.8 Hz, 3 H). ^{13}C NMR (100 MHz, $CDCl_3$): δ175.1, 140.7, 128.8(2C), 128.4(2C), 126.9, 62.0, 60.4, 57.5, 55.6, 46.2, 36.9, 32.3, 28.0, 27.6, 26.9, 26.3, 22.9, 14.6, 14.4, 8.6. HRMS (ESI) m/z calcd. for $C_{22}H_{35}O_2N$ $[M+H]^+$ 346.2746; found 346.2735. IR (neat): ν_{max}/cm^{-1} 2957, 2929, 2873, 2856, 2791, 2754, 1732, 1453, 1377, 1338, 1254, 1237, 1185, 1169. $[\alpha]_D^{30}$ = -29.2 (c = 1.1 in $CHCl_3$).

2.2f. Synthesis of compound 7[2]

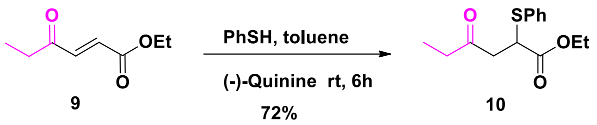

To a solution of thiophenol (39 mg, 36 µl, 0.35 mmol, 1.1 equiv) and Quinine (10.5 mg, 0.032 mmol, 0.1 equiv) in toluene, was added enone **9** (50 mg, 0.32 mmol, 1 equiv) at rt and reaction mixture was allowed stirred for 6 h. Then reaction mixture was quenched with water and extracted with ethyl acetate (15ml X 3), washed with brine, dried over anhydrous Na_2SO_4, concentrated *in vacuo* and purified by column chromatography furnishing the product **10**[2](61.2 mg, 0.23 mmol, 72%).

Ethyl 4-oxo-2-(phenylthio)hexanoate (10)

^{1}H NMR (400 MHz, $CDCl_3$): δ 7.54 - 7.45 (m, 2 H), 7.37 - 7.29 (m, 3 H), 4.11 (q, *J* = 7.1 Hz, 2 H), 4.06 (dd, *J* = 4.6, 10.1 Hz, 1 H), 3.10 (dd, *J* = 10.4, 17.7 Hz, 1 H), 2.79 (dd, *J* = 4.6, 18.0 Hz, 1 H), 2.48 - 2.39 (m, 2 H), 1.17 (t, *J* = 7.3 Hz, 3 H), 1.05 (t, *J* = 7.3 Hz, 3 H). ^{13}C NMR (100 MHz, $CDCl_3$): δ 208.4, 171.9, 134.0 (2C), 132.7, 129.3 (2C), 128.8, 61.7, 45.1, 44.3, 36.3, 14.3, 7.9. HRMS (ESI) m/z calcd. for $C_{14}H_{18}O_3S$ $[M+Na]^+$ 289.0874; found 289.0878. IR (neat): ν_{max}/cm^{-1} 2979, 2938, 2906, 1731, 1582, 1439, 1369, 1223, 1173, 1153.

2.3. Synthesis of norpyrenophorin (14)

2.3a. Synthesis of compound 19

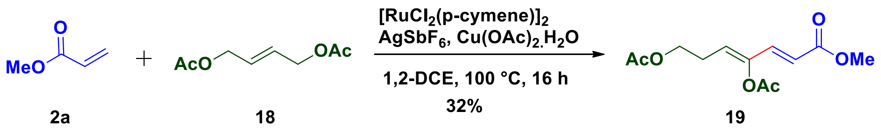

By following the general procedure, A 50 mL seal tube was charged with $[RuCl_2(p\text{-cymene})]_2$ (179 mg, 5.83 mmol, 5.0 mol%), $Cu(OAc)_2 \cdot H_2O$ (2.32 g, 11.6 mmol, 2.0 equiv), $AgSbF_6$ (400 mg, 2.0 mmol, 20 mol%) and 1,2-dichloroethane (20 mL). Then methyl acrylate **2a** (250mg, 0.255ml, 2.90 mmol, 0.5equiv) and diacetate **18** (1.0 g,5.83 mmol, 1.0 equiv) were added into the solution in sequence. Then vial was sealed under N_2 and heated to 100 °C with stirring for 16 h. After cooling down, the mixture was diluted with ethyl acetate, filtrated and concentrated to give the crude compound which was directly purified by column chromatography (3:1:: Pet ether : EtOAc) to give **19** (477 mg, 1.86 mmol, 32%).

(3Z,5E)-7-methoxy-7-oxohepta-3,5-diene-1,4-diyl diacetate (19)

^{1}H NMR (400 MHz, $CDCl_3$): δ 7.17 (d, *J* = 15.9 Hz, 1 H), 5.83 - 5.73 (m, 2 H), 4.13 (t, *J* = 6.7 Hz, 2 H), 3.75 (s, 3 H), 2.42 (q, *J* = 6.9 Hz, 2 H), 2.28 (s, 3 H), 2.05 (s, 3 H). ^{13}C NMR (101 MHz, $CDCl_3$): δ 171.0, 167.8, 166.7, 146.0, 138.7, 125.9, 117.7, 62.3, 51.8, 26.4, 20.9, 20.3. HRMS (ESI) m/z calcd. for $C_{12}H_{16}O_6$ $[M+NH_4]^+$ 274.1291; found 274.1296. IR (neat): ν_{max}/cm^{-1} 2954, 2849, 1765, 1738, 1652, 1627, 1435, 1368, 1309, 1238, 1198, 1172, 1037.

2.3b. Synthesis of compound 20

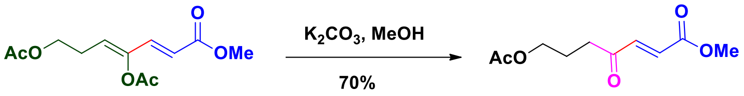

19 20

To a solution of **19** (450 mg, 1.75 mmol, 1.0 equiv) in methanol, added potassium carbonate (218 mg, 1.58 mmol, 0.9 equiv) in four portions over 5 min and reaction mixture was allowed to stirred for 40 minutes at room temperature. Then reaction mixture was quenched with water and extracted with ethyl acetate (15ml X 3), washed with brine, dried over anhydrous Na_2SO_4, concentrated *in vacuo* and purified by column chromatography (3 : 1.5 :: Pet ether : EtOAc) furnishing the compound **20** (262mg, 1.22mmol, 70%).

(E)-methyl 7-acetoxy-4-oxohept-2-enoate (20)
^{1}H NMR (400 MHz, $CDCl_3$): δ 7.08 (d, *J* = 16.0 Hz, 1 H), 6.70 (d, *J* = 16.0 Hz, 1 H), 4.10 (t, *J* = 6.3 Hz, 2 H), 3.82 (s, 3 H), 2.74 (t, *J* = 6.9 Hz, 2 H), 2.04 (s, 3 H), 2.02 - 1.97 (m, 2 H). ^{13}C NMR (101 MHz, $CDCl_3$): δ 198.5, 171.1, 166.0, 139.3, 130.6, 63.4, 52.5, 38.0, 22.7, 21.0. HRMS (ESI) m/z calcd. for $C_{10}H_{14}O_5$ $[M+Na]^+$ 237.0739; found 237.0730. IR (neat): v_{max}/cm^{-1} 2956, 2853, 1737, 1731, 1703, 1643, 1437, 1366, 1305, 1241, 1176, 1040.

2.3c. Synthesis of compound 21

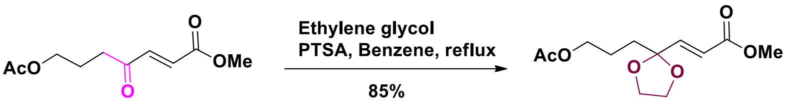

20 21

Using a Dean-Stark trap, ethylene glycol (1.3 ml, 23.36 mmol, 30 equiv.) was refluxed in benzene (5 mL) for 1 hour. Upon cooling, compound **20** (250 mg, 1.16 mmol) in benzene (2 mL) and p-toluene sulfonic acid (10 mg, 0.046 mmol, 0.04 equiv.) were added to the solution and the resulting mixture was heated to reflux under a condenser for 1.5 hours (heating for more than 1.5 hours resulted in decomposition of product). The solution was then cooled and quenched with $NaHCO_3$ (10 mL). The resulting mixture was extracted with EtOAc (3 x 10 mL), washed with water (80 mL) and brine (80 mL), dried over Na_2SO_4 and concentrated. The crude product was purified by column chromatography (3 : 1.5:: Pet ether : EtOAc) to obtain of the pure ketal **21** (254 mg, 0.986 mmol, 85% yield) as a yellow liquid.

(E)-methyl 3-(2-(3-acetoxypropyl)-1,3-dioxolan-2-yl)acrylate (21)
^{1}H NMR (400 MHz, $CDCl_3$): δ 6.73 (d, *J* = 15.6 Hz, 1 H), 6.09 (d, *J* = 15.8 Hz, 1 H), 4.07 (t, *J* = 6.4 Hz, 2 H), 4.00 - 3.95 (m, 2 H), 3.90 - 3.85 (m, 2 H), 3.76 (s, 3 H), 2.04 (s, 3 H), 1.85 - 1.78 (m, 2 H), 1.78 - 1.70 (m, 2 H). ^{13}C NMR (101 MHz, $CDCl_3$): δ 171.4, 166.9, 146.6, 121.9, 108.2, 65.3 (2C), 64.5, 52.1, 34.4, 22.8, 21.3. HRMS (ESI) m/z calcd. for $C_{12}H_{18}O_6$ $[M+Na]^+$ 281.1001; found 281.1000. IR (neat): v_{max}/cm^{-1}2955, 2895, 1731 (br.), 1663, 1436, 1387, 1365, 1305, 1243, 1197, 1169, 1036.

2.3d. Synthesis of compound 22

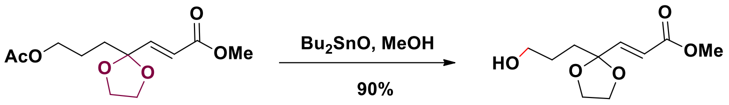

21 22

To a solution of **21** (240 mg, 0.930 mmol, 1.0 equiv) in methanol (8 ml), added dibutyl tin oxide (2.31 g, 5.9 mmol, 0.9 equiv) in one portion and reaction mixture was refluxed for 2 hours. Upon cooling the reaction mixture, concentrated *in vacuo,* solid was removed by filtration, residue washed with ethyl acetate and filterarte was concentrated, purified by column chromatography (3 : 2 :: Pet ether : EtOAc) furnishing theproduct **22** (216 mg, 0.93 mmol, 90%) as a pale brownish liquid.

(E)-methyl 3-(2-(3-hydroxypropyl)-1,3-dioxolan-2-yl)acrylate (22)
^{1}H NMR (400 MHz, $CDCl_3$): δ 6.73 (d, *J* = 15.4 Hz, 1 H), 6.07 (d, *J* = 15.4 Hz, 1 H), 4.00 - 3.96 (m, 2 H), 3.89 - 3.85 (m, 2 H), 3.74 (s, 3 H), 3.63 (t, *J* = 5.9 Hz, 2 H), 2.03 (br. s., 1 H), 1.87 - 1.82 (m, 2 H), 1.69 - 1.64 (m, 2 H). ^{13}C NMR (101 MHz, $CDCl_3$): δ 166.6, 146.3, 121.5, 108.1, 64.9 (2C), 62.5, 51.8, 34.1, 26.2. HRMS (ESI) m/z calcd. for $C_{10}H_{16}O_5$ $[M+Na]^+$ 239.0895; found 239.0894. IR (neat): v_{max}/cm^{-1} 3400 (br.), 2954, 2849, 1765, 1738, 1652, 1627, 1435, 1368, 1309, 1238, 1198, 1172, 1037.

2.3e. Synthesis of norpyrenophorin 14[3]

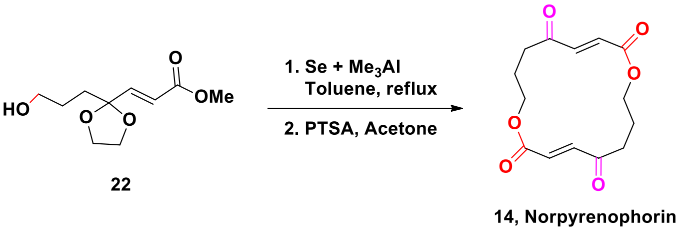

Trimethylaluminium (0.925 mL of a 2 M solution, 1.85 mmol, 10 equiv) in toluene was added to a 5 mL side-arm flask containing selenium powder (150 mg, 1.924 mmol, 10.4 equiv) at 0 °C and refluxed till the selenium powder had reacted completely, the resulting mixture was cooled to room temperature. An aliquot of $Me_2SeAlMe$ (10 µl, 1.1 mmol) was transferred by syringe to a solution containing compound **22** (40 mg, 0.185 mmol) in toluene (1.2 mL) at 0 °C. After 28 h of heating at reflux, the yellow solution was warmed to room temperature over a period of 30 min and treated with sodium sulfate. The solution was then diluted with dichloromethane, washed with water six times and treated with sodium hydrogen carbonate. The solution was then dried with brine and anhydrous magnesium sulfate. The solvent was removed under vacuum from the yellow solution and the residue wasdissolved in acetone (0.75 ml), added p-toluene sulfonic acid (3 mg, 0.0081 mmol, 0.1 equiv) at 0 °C and stirred for 4 hours at room temperature. then reaction mixture was quenched with $NaHCO_3$ solution and extracted with EtOAc, dried over Na_2SO_4 and concentrated. The crude product was purified by column chromatography(1:1 :: Pet ether : EtOAc) furnishing the **norpyrenophorin 14**[3](6 mg, 0.0214 mmol, 23% in two steps).

Norpyrenophorin (14)

^{1}H NMR (500 MHz, $CDCl_3$): δ 7.03 (d, J = 16.3 Hz, 1 H), 6.68 (d, J = 16.2 Hz, 1 H), 4.31 (t, J = 5.9 Hz, 2 H), 2.85 (t, J = 7.0 Hz, 2 H), 2.13 - 2.07 (m, 2 H). ^{13}C NMR (101 MHz, $CDCl_3$): δ 198.5, 165.4, 140.2, 131.2, 64.6, 37.2, 22.4. HRMS (ESI) m/z calcd. for $C_{14}H_{17}O_6$ $[M+H]^+$ 281.1025; found 281.1025. IR (neat): v_{max}/cm^{-1} 2923, 2852, 1728, 1428, 1258.

3. NMR spectra

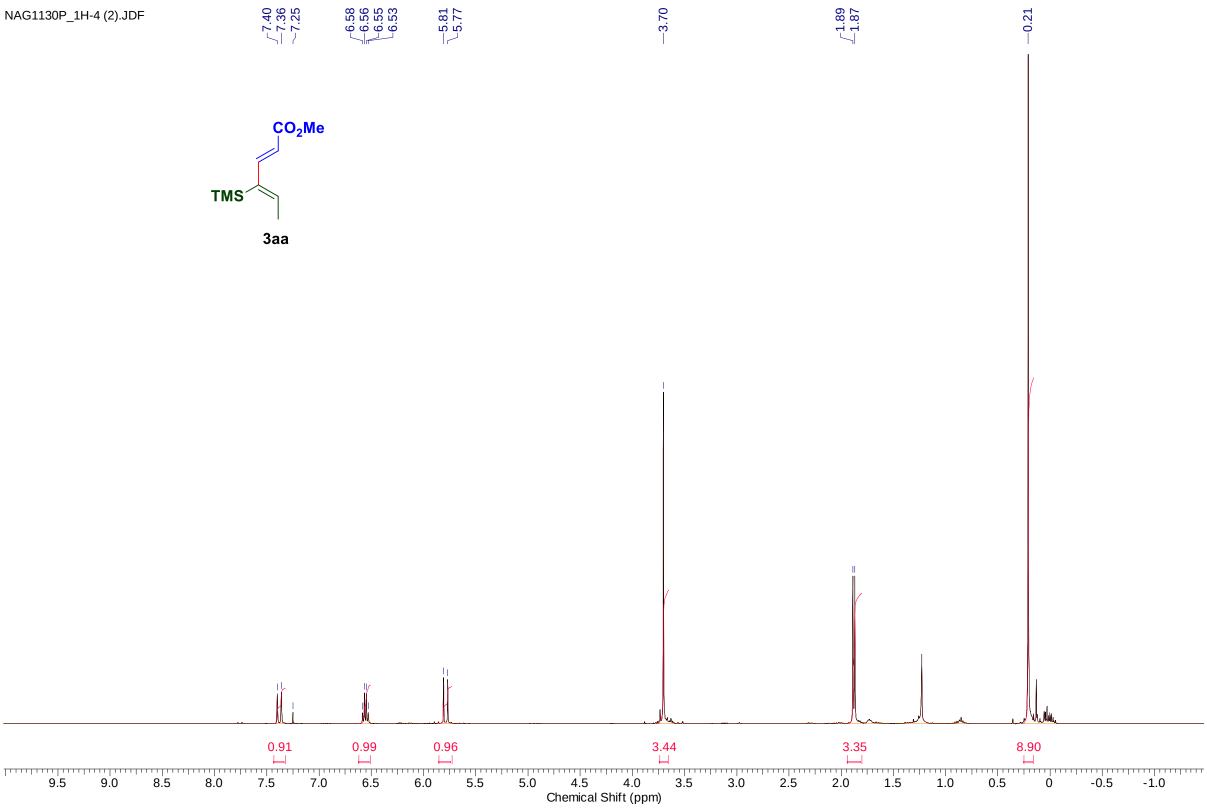

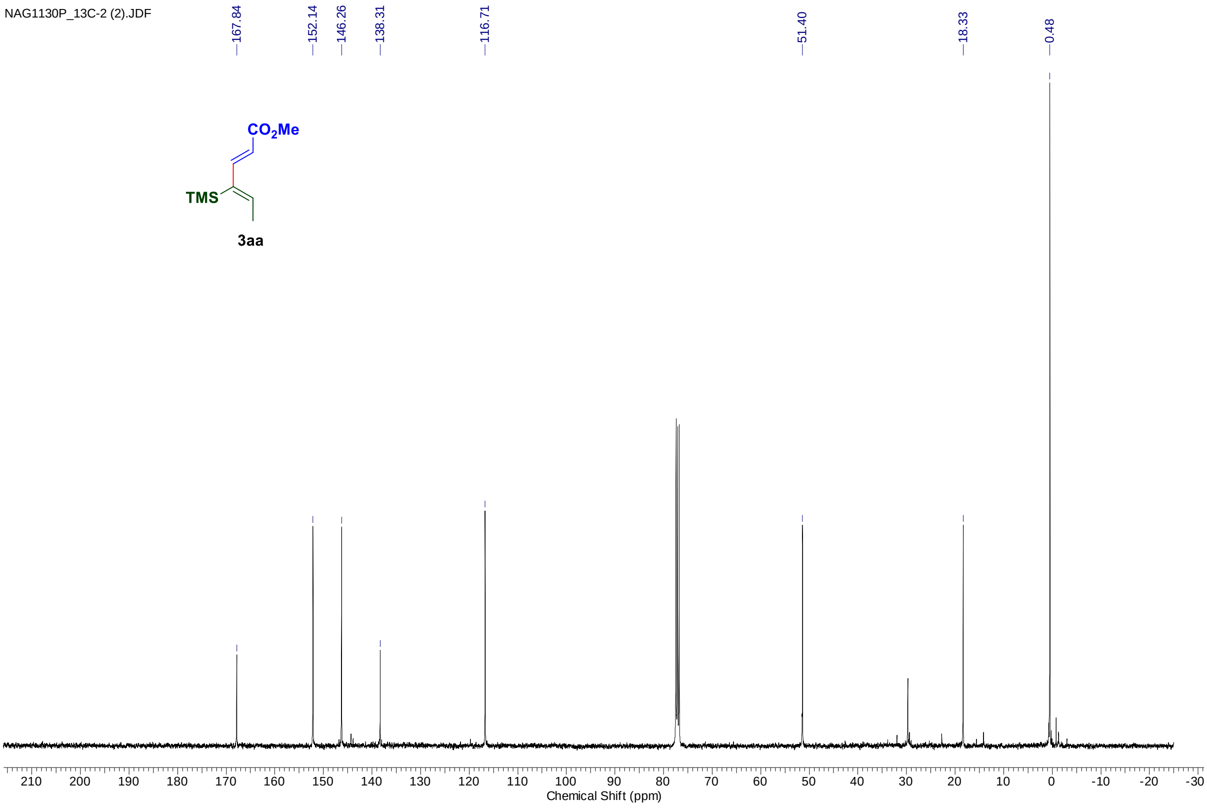

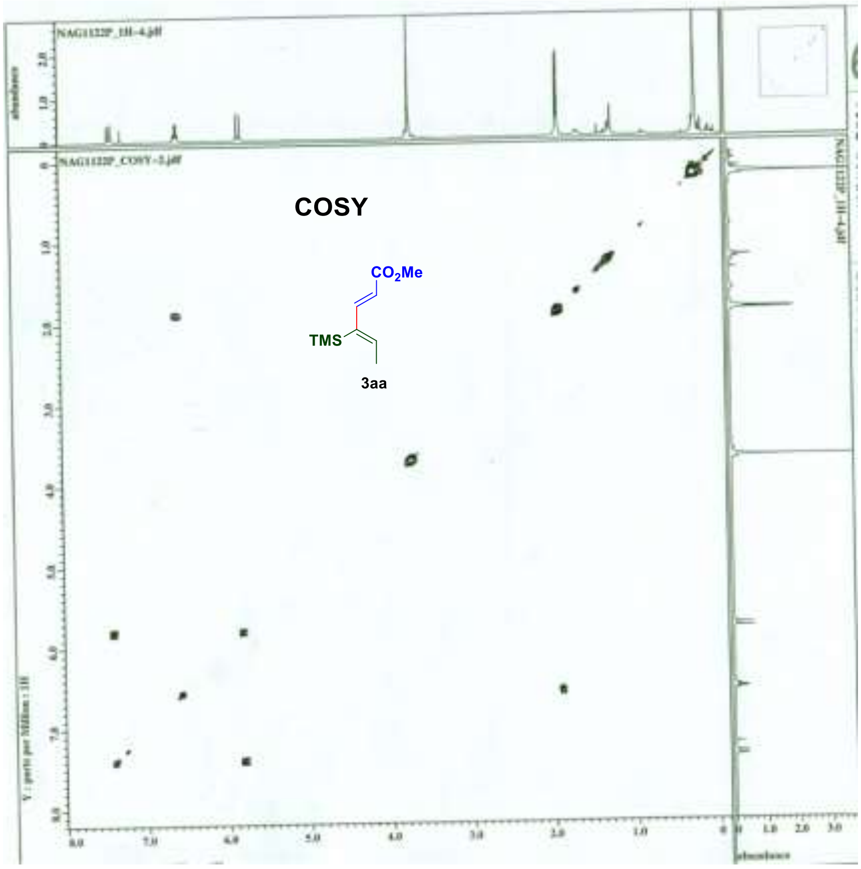
COSY
CO2Me
TMS
3aa

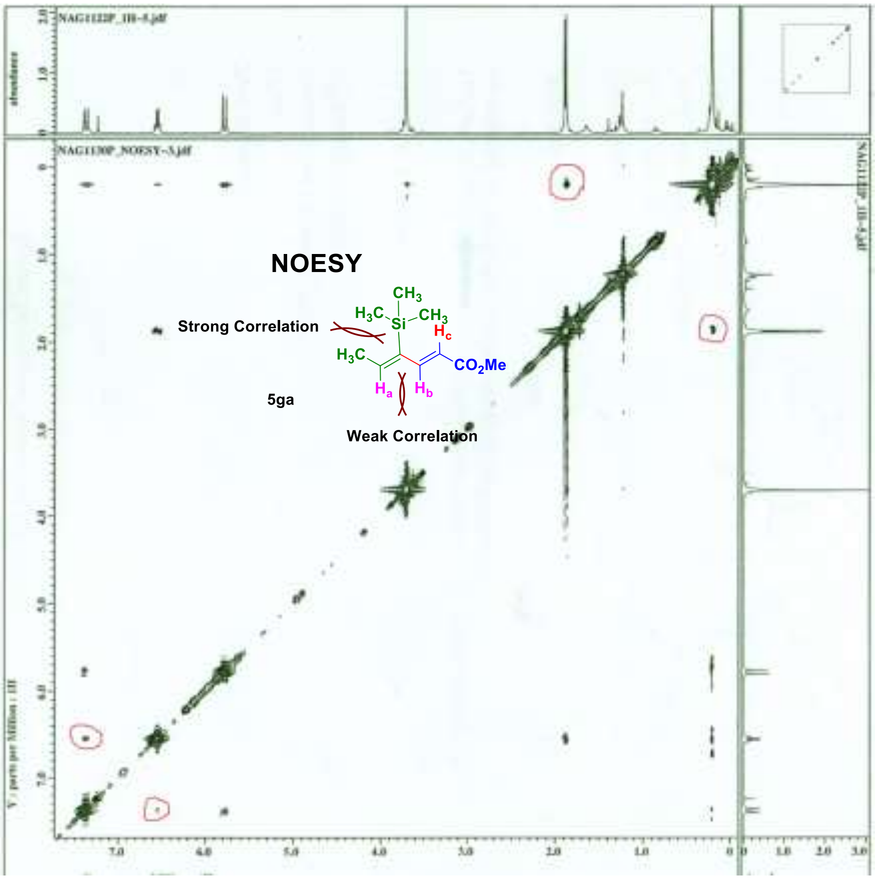
NOESY
Strong Correlation
CH3
H3C
Si
CH3
Hc
H3C
CO2Me
Ha
Hb
5ga
Weak Correlation

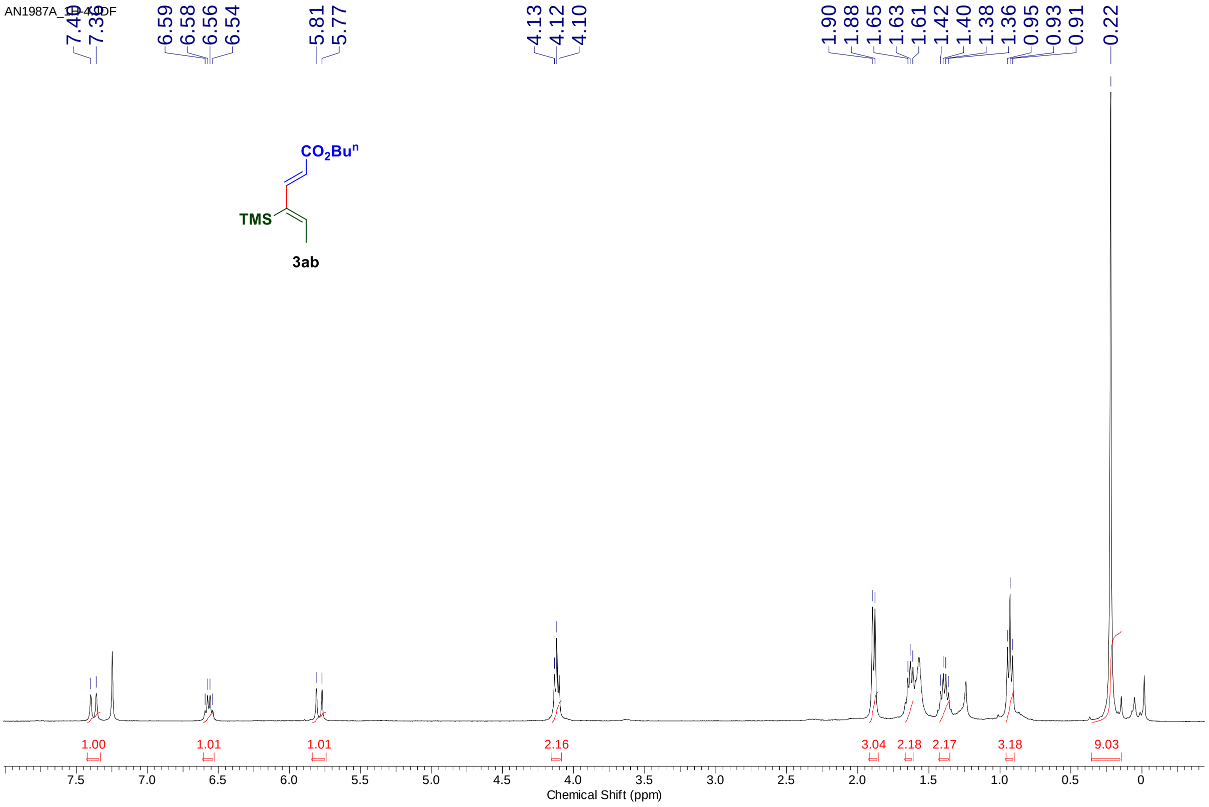
AN1987A_1H-4.JDF
7.40
7.39
6.59
6.58
6.56
6.54
5.81
5.77
4.13
4.12
4.10
1.90
1.88
1.65
1.63
1.61
1.42
1.40
1.38
1.36
0.95
0.93
0.91
0.22
CO2Bun
TMS
3ab
1.00
1.01
1.01
2.16
3.04
2.18
2.17
3.18
9.03
Chemical Shift (ppm)

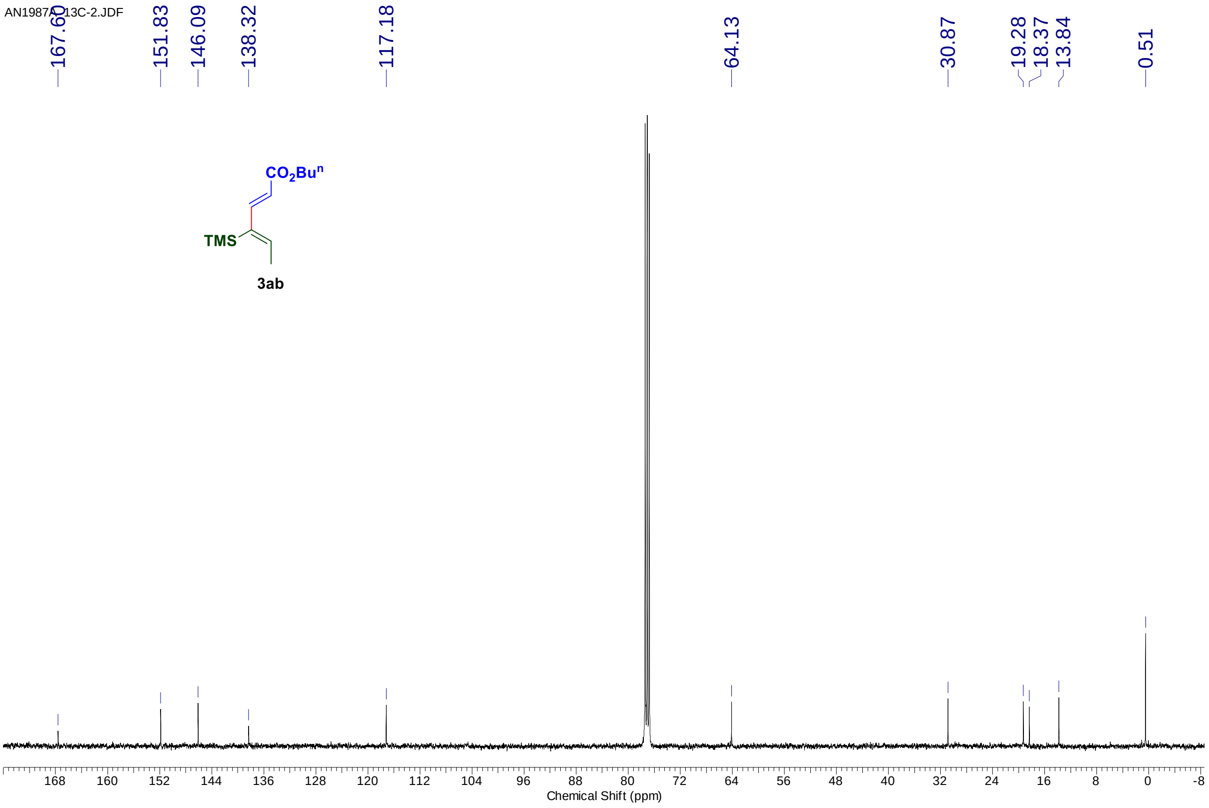
AN1987A_13C-2.JDF
167.60
151.83
146.09
138.32
117.18
64.13
30.87
19.28
18.37
13.84
0.51
CO2Bun
TMS
3ab
Chemical Shift (ppm)

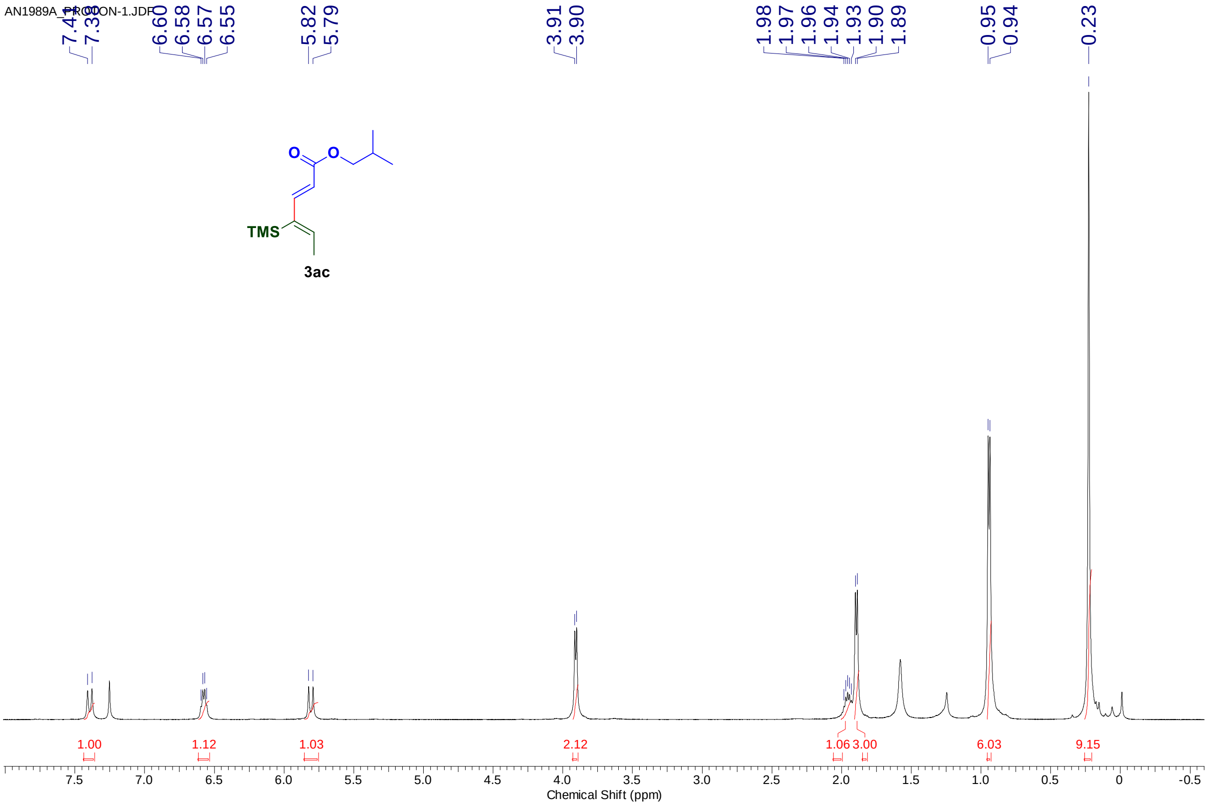
AN1989A_PROTON-1.JDF
7.41
7.39
6.60
6.58
6.57
6.55
5.82
5.79
3.91
3.90
1.98
1.97
1.96
1.94
1.93
1.90
1.89
0.95
0.94
0.23
O
O
TMS
3ac
1.00
1.12
1.03
2.12
1.06
3.00
6.03
9.15
Chemical Shift (ppm)

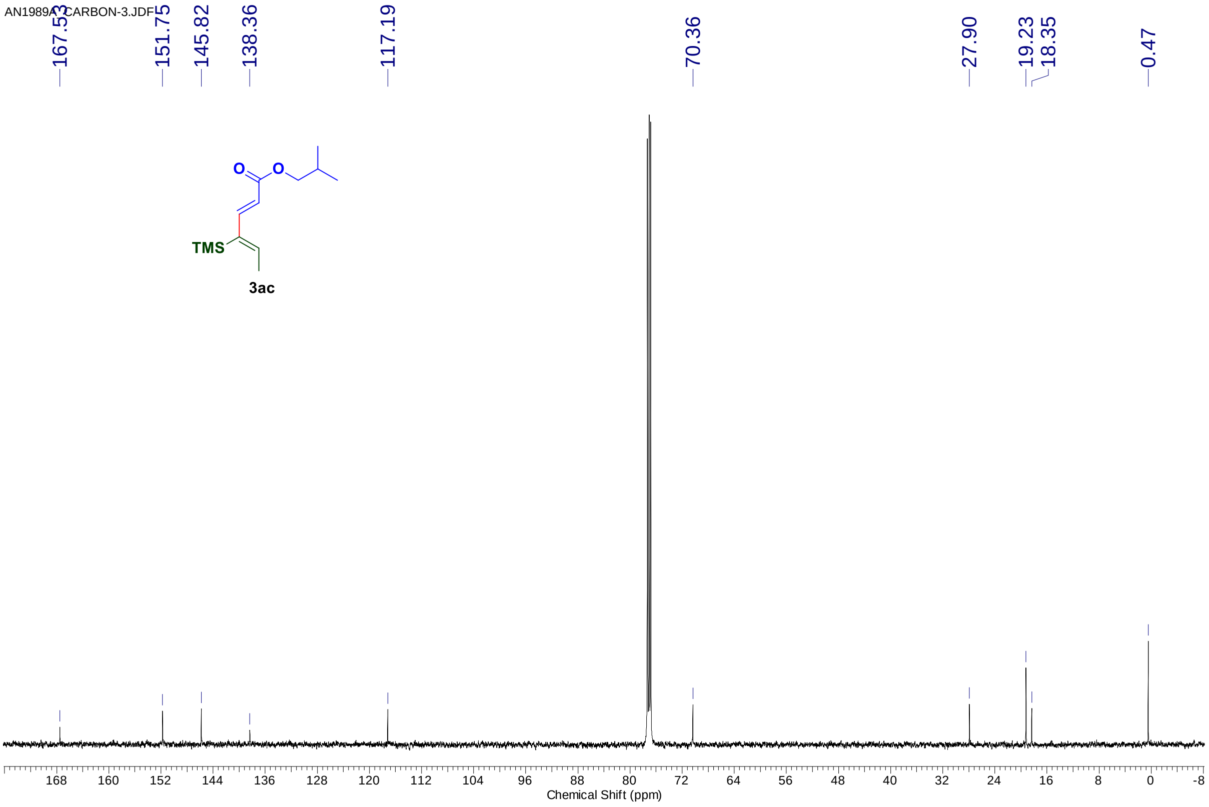
AN1989A_CARBON-3.JDF
167.53
151.75
145.82
138.36
117.19
70.36
27.90
19.23
18.35
0.47
O
O
TMS
3ac
Chemical Shift (ppm)

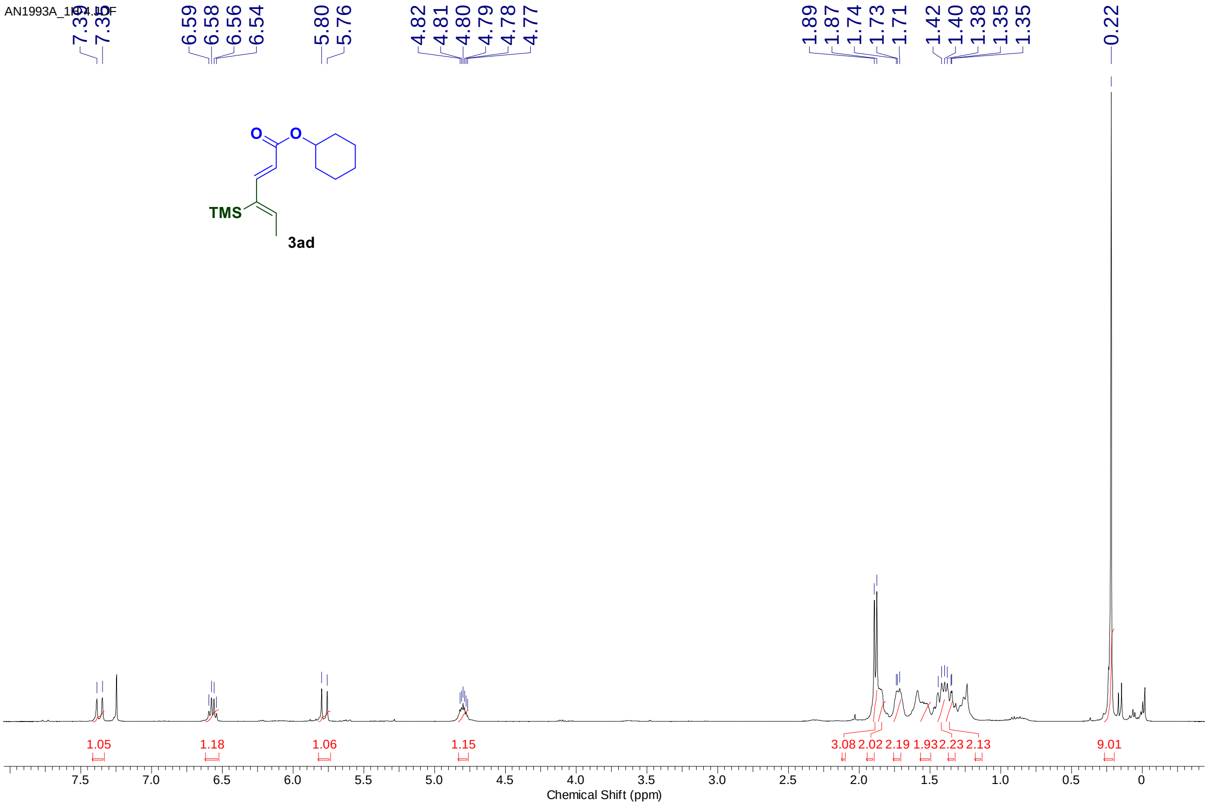
7.39
7.35
6.59
6.58
6.56
6.54
5.80
5.76
4.82
4.81
4.80
4.79
4.78
4.77
1.89
1.87
1.74
1.73
1.71
1.42
1.40
1.38
1.35
1.35
0.22
O
O
TMS
3ad
1.05
1.18
1.06
1.15
3.08
2.02
2.19
1.93
2.23
2.13
9.01
Chemical Shift (ppm)

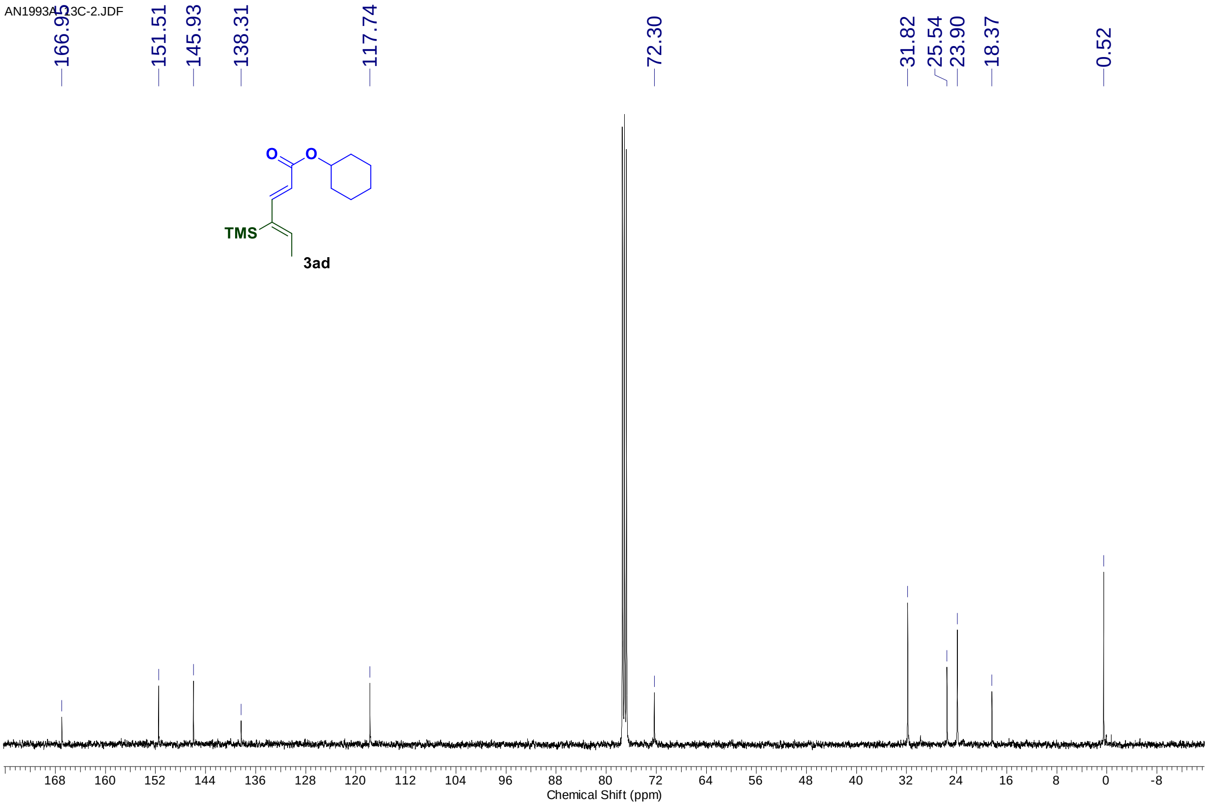
166.95
151.51
145.93
138.31
117.74
72.30
31.82
25.54
23.90
18.37
0.52
O
O
TMS
3ad
Chemical Shift (ppm)

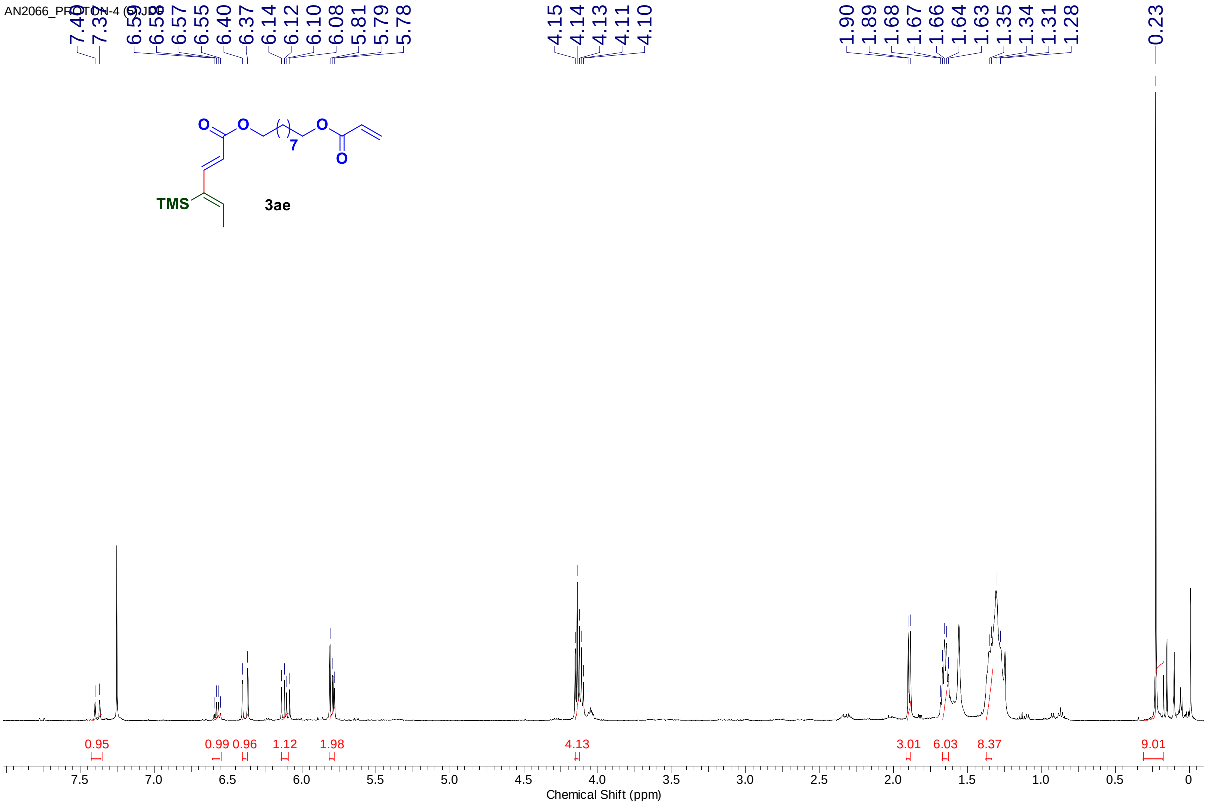
7.40
6.59
6.57
6.55
6.40
6.37
6.14
6.12
6.10
6.08
5.81
5.79
5.78
4.15
4.14
4.13
4.11
4.10
1.90
1.89
1.68
1.67
1.66
1.64
1.63
1.35
1.34
1.31
1.28
0.23
TMS
3ae
0.95
0.99
0.96
1.12
1.98
4.13
3.01
6.03
8.37
9.01
Chemical Shift (ppm)

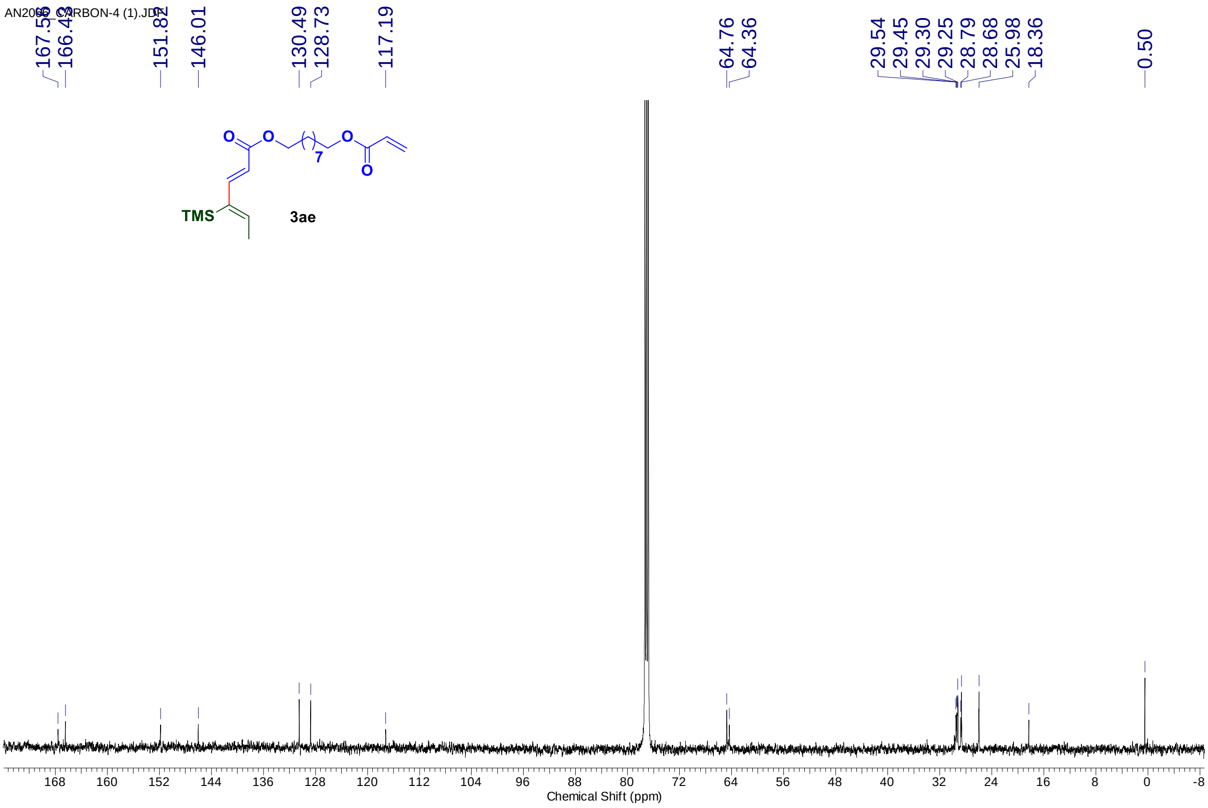
146.01
130.49
128.73
117.19
64.76
64.36
29.54
29.45
29.30
29.25
28.79
28.68
25.98
18.36
0.50
TMS
3ae
Chemical Shift (ppm)

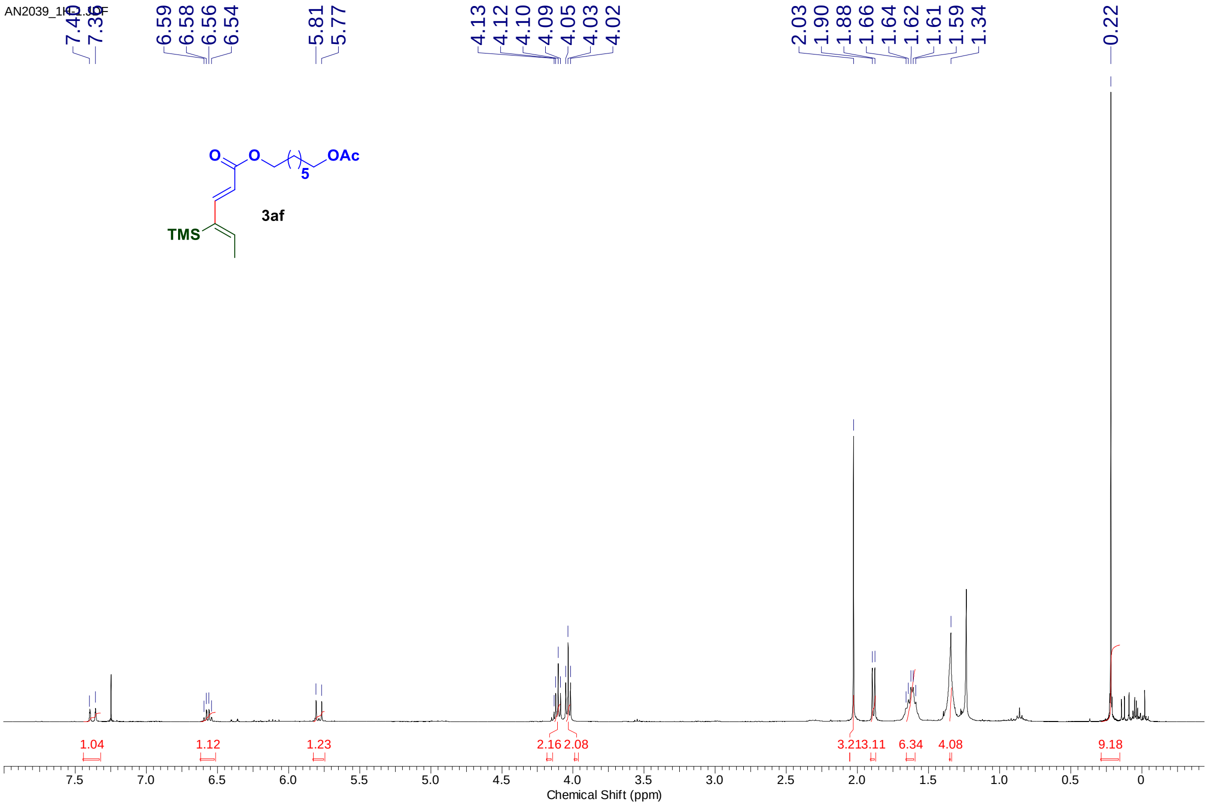
AN2039_1H-1.JDF
7.40
7.36
6.59
6.58
6.56
6.54
5.81
5.77
4.13
4.12
4.10
4.09
4.05
4.03
4.02
2.03
1.90
1.88
1.66
1.64
1.62
1.61
1.59
1.34
0.22
O
O
5
OAc
TMS
3af
1.04
1.12
1.23
2.16
2.08
3.21
3.11
6.34
4.08
9.18
Chemical Shift (ppm)

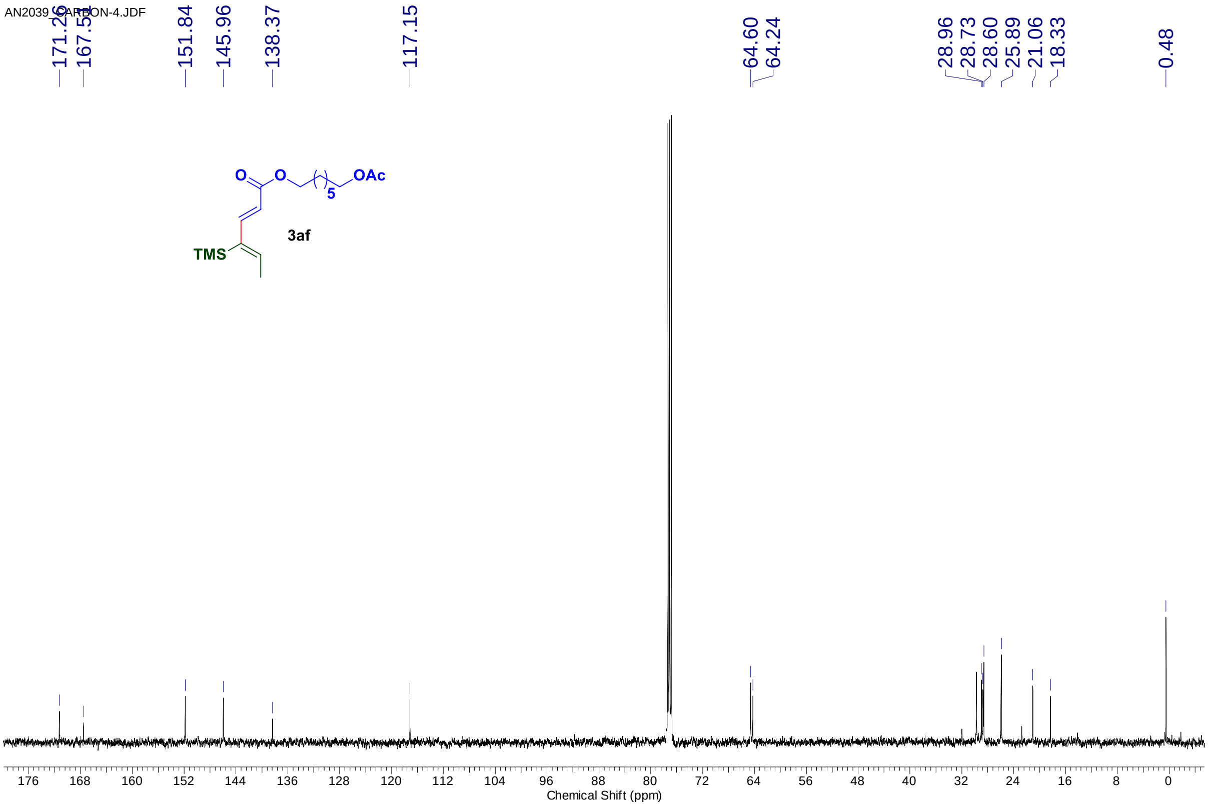
AN2039_CARBON-4.JDF
171.26
167.53
151.84
145.96
138.37
117.15
64.60
64.24
28.96
28.73
28.60
25.89
21.06
18.33
0.48
O
O
5
OAc
TMS
3af
Chemical Shift (ppm)

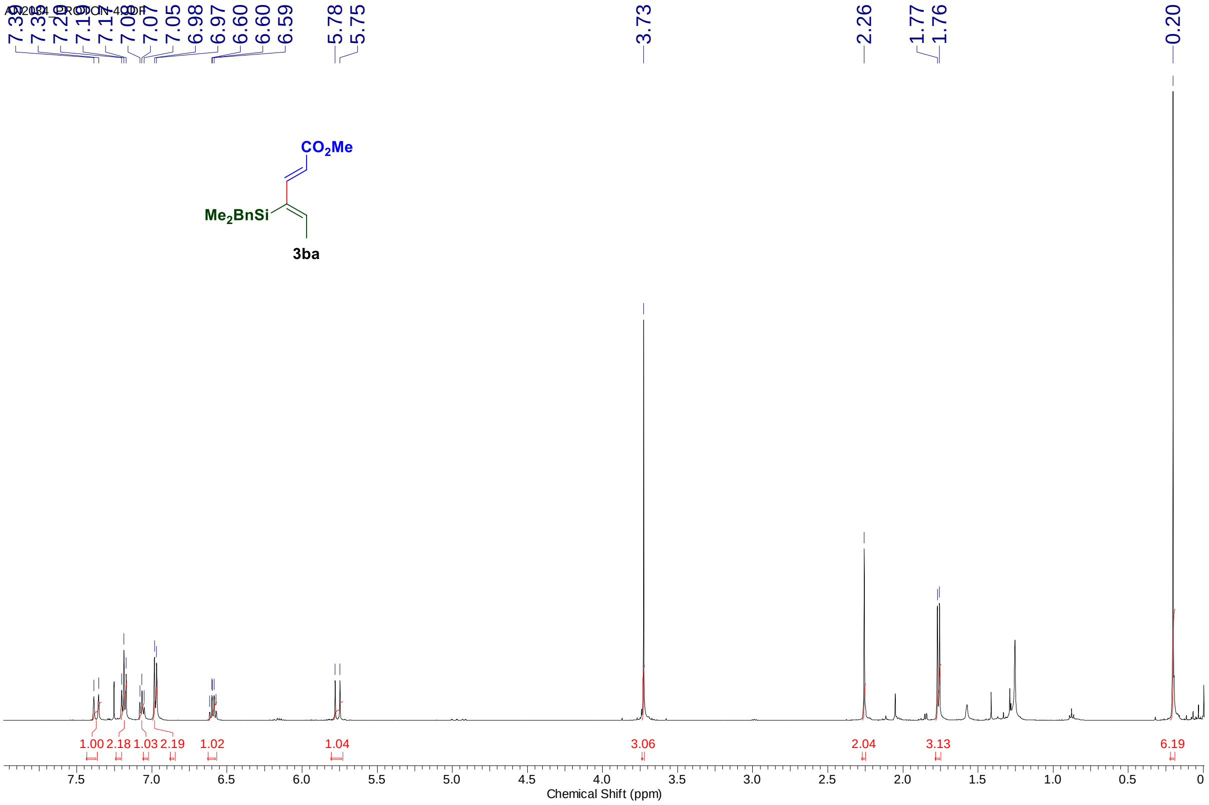
7.05
6.98
6.97
6.60
6.60
6.59
5.78
5.75
3.73
2.26
1.77
1.76
0.20
CO2Me
Me2BnSi
3ba
1.00
2.18
1.03
2.19
1.02
1.04
3.06
2.04
3.13
6.19
Chemical Shift (ppm)

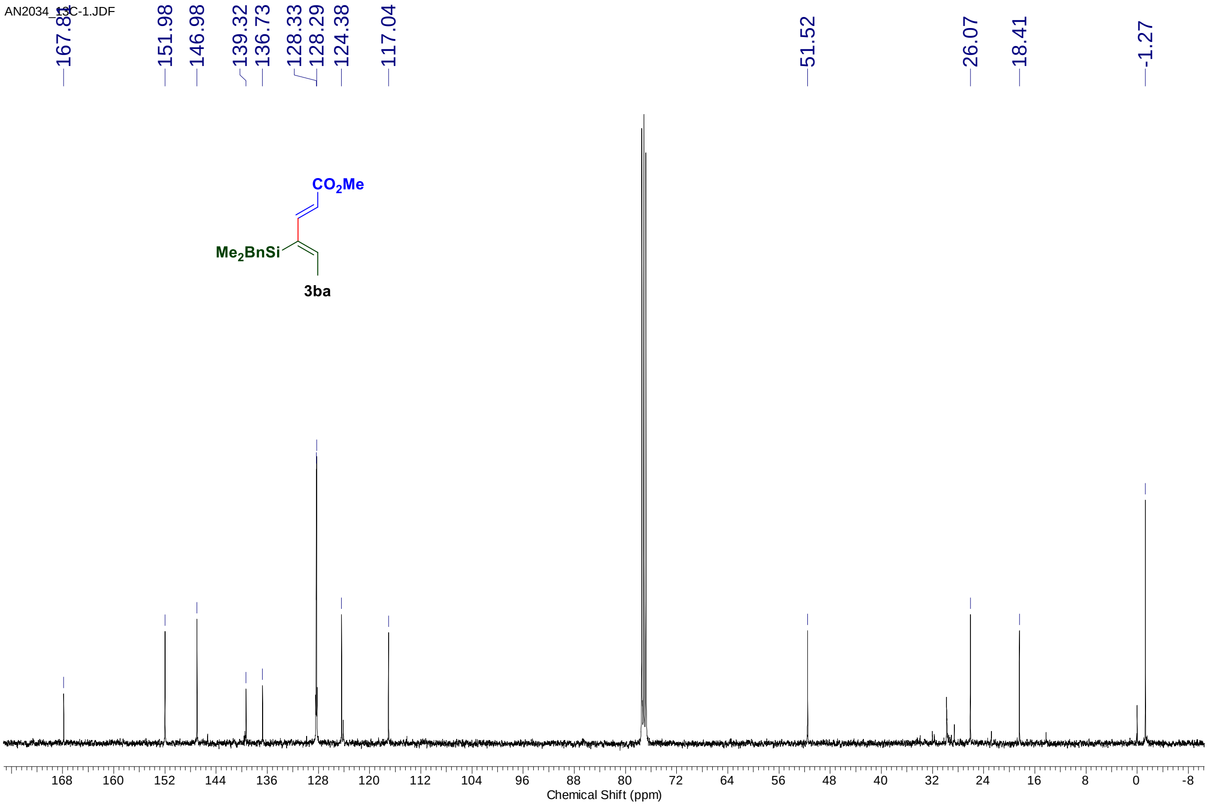
151.98
146.98
139.32
136.73
128.33
128.29
124.38
117.04
51.52
26.07
18.41
-1.27
CO2Me
Me2BnSi
3ba
Chemical Shift (ppm)

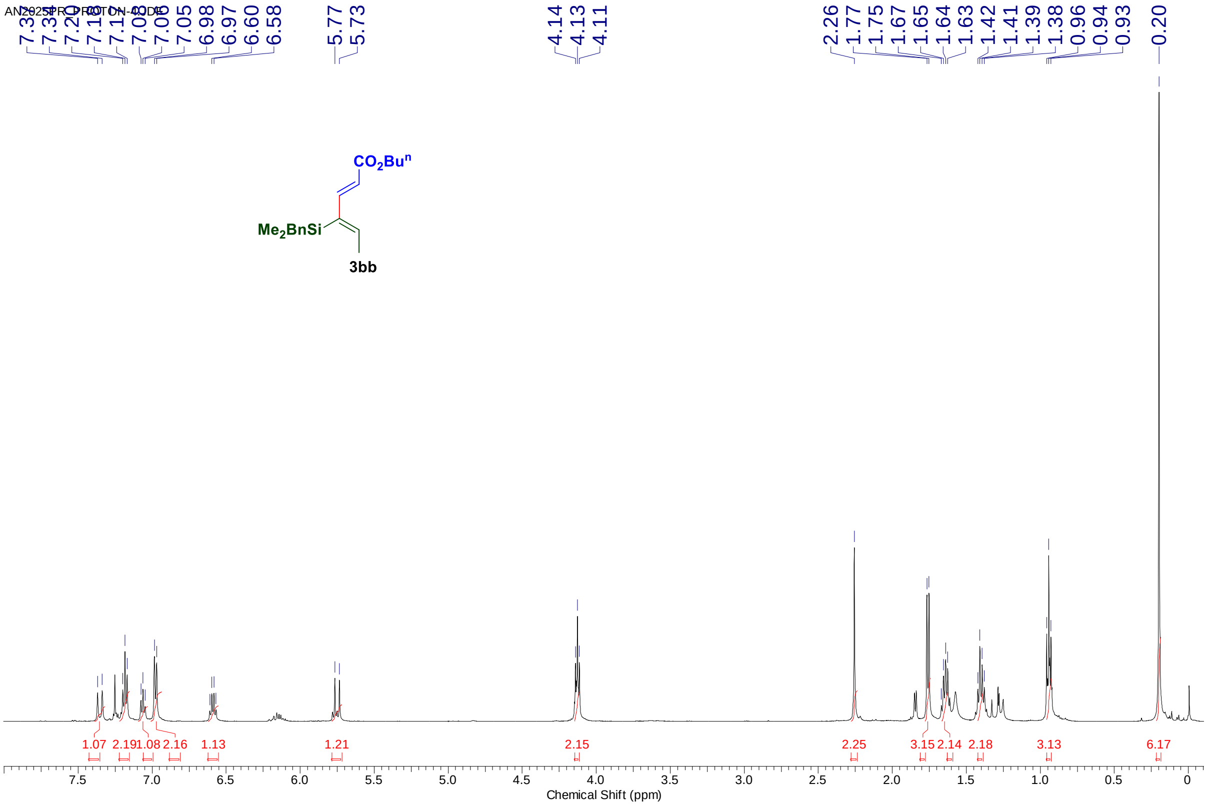
4.14
4.13
4.11
2.26
1.77
1.75
1.67
1.65
1.64
1.63
1.42
1.41
1.39
1.38
0.96
0.94
0.93
0.20
7.05
6.98
6.97
6.60
6.58
5.77
5.73
CO2Bun
Me2BnSi
3bb
1.07
2.19
1.08
2.16
1.13
1.21
2.15
2.25
3.15
2.14
2.18
3.13
6.17
Chemical Shift (ppm)

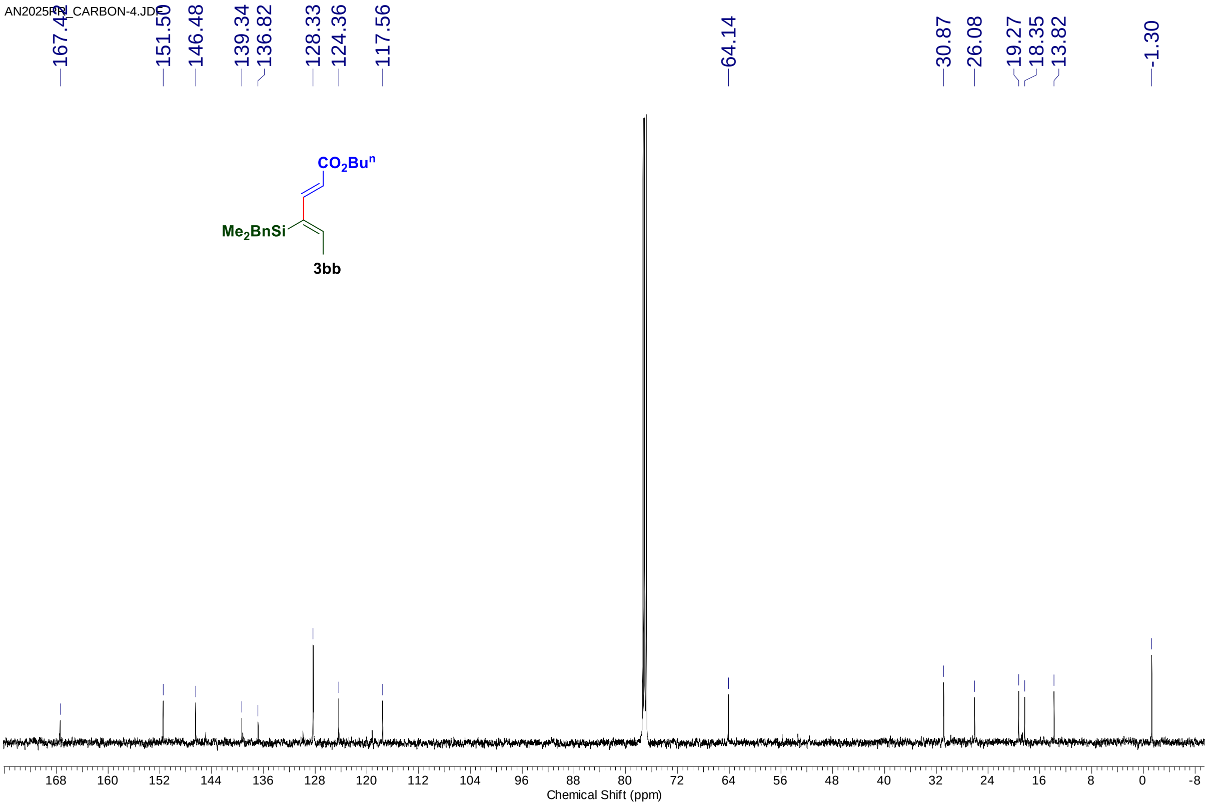
AN2025RN_CARBON-4.JDF
151.50
146.48
139.34
136.82
128.33
124.36
117.56
64.14
30.87
26.08
19.27
18.35
13.82
-1.30
CO2Bun
Me2BnSi
3bb
Chemical Shift (ppm)

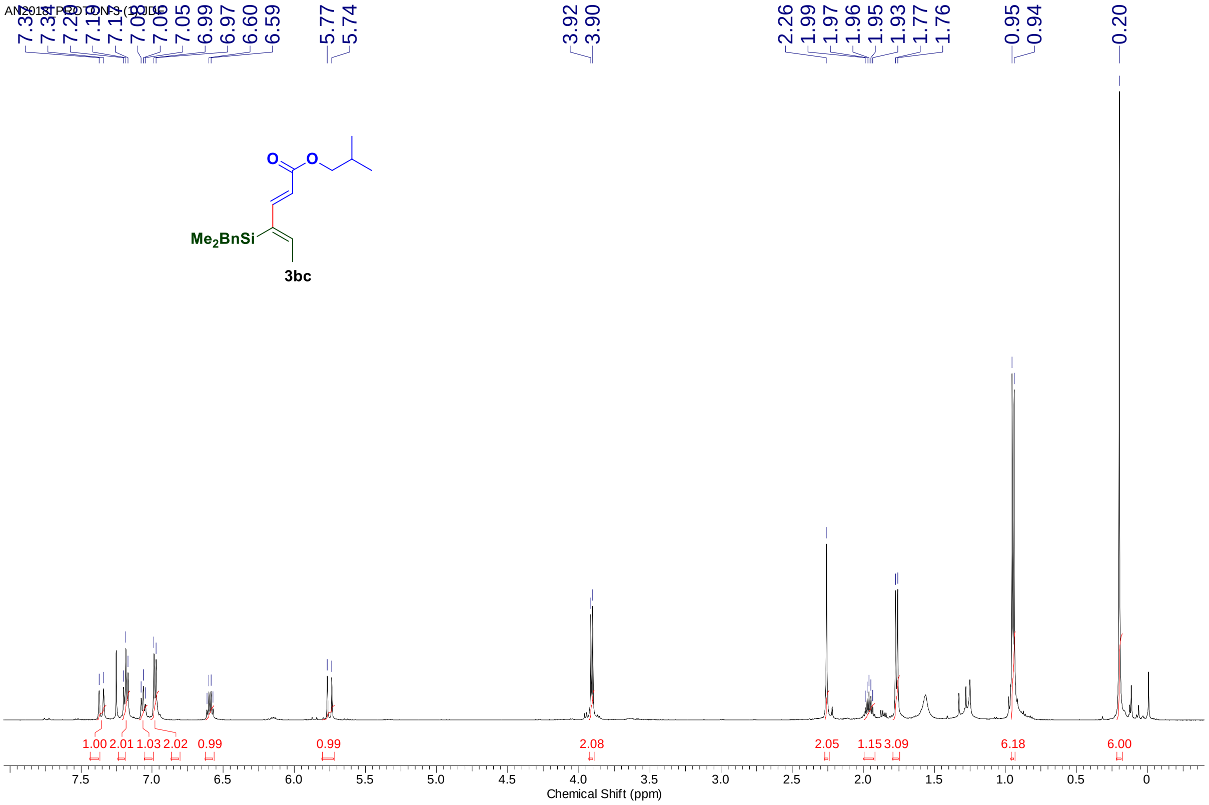
Me2BnSi
3bc
7.05
6.99
6.97
6.60
6.59
5.77
5.74
3.92
3.90
2.26
1.99
1.97
1.96
1.95
1.93
1.77
1.76
0.95
0.94
0.20
1.00 2.01 1.03 2.02 0.99
0.99
2.08
2.05
1.15 3.09
6.18
6.00
Chemical Shift (ppm)

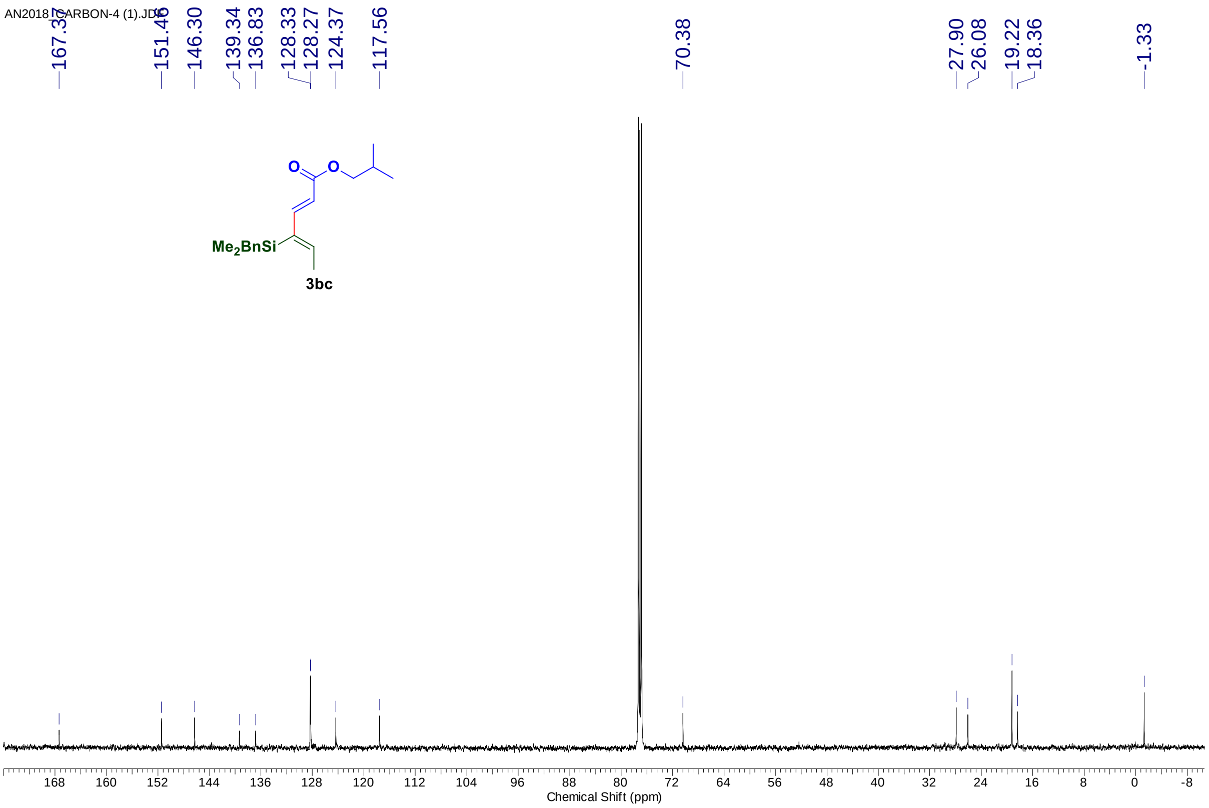
AN2018 CARBON-4 (1).JDF
Me2BnSi
3bc
151.40
146.30
139.34
136.83
128.33
128.27
124.37
117.56
70.38
27.90
26.08
19.22
18.36
-1.33
Chemical Shift (ppm)

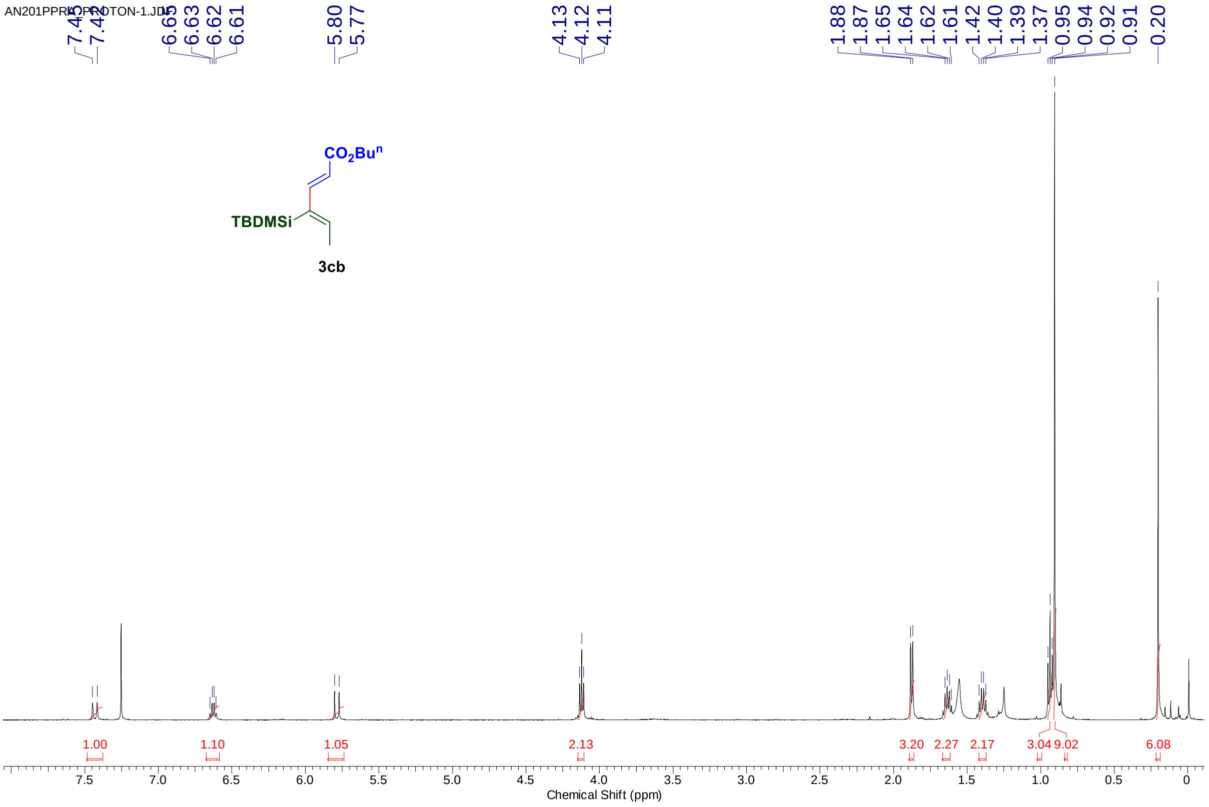
AN201PPRA_PROTON-1.JDF
7.43
7.41
6.65
6.63
6.62
6.61
5.80
5.77
4.13
4.12
4.11
1.88
1.87
1.65
1.64
1.62
1.61
1.42
1.40
1.39
1.37
0.95
0.94
0.92
0.91
0.20
CO_2Bu^n
TBDMSi
3cb
1.00
1.10
1.05
2.13
3.20
2.27
2.17
3.04
9.02
6.08
Chemical Shift (ppm)

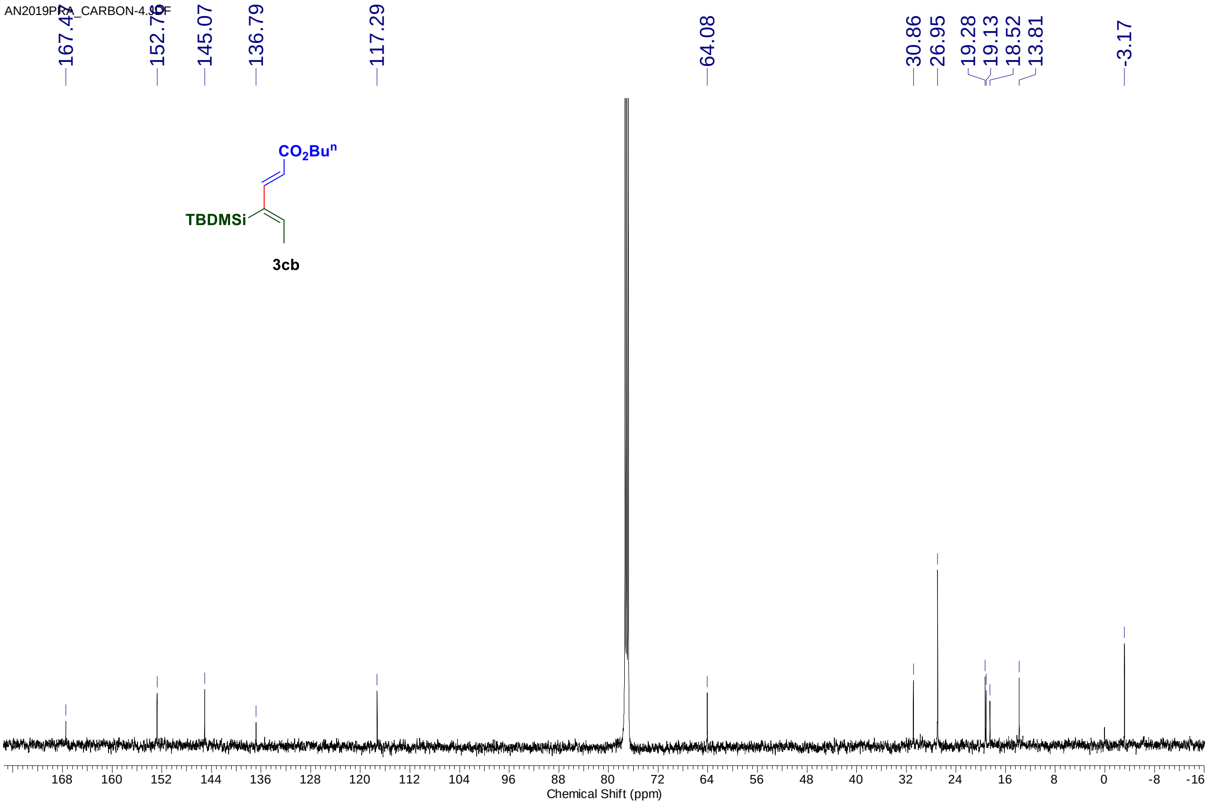
AN2019PRA_CARBON-4.JDF
167.47
152.76
145.07
136.79
117.29
64.08
30.86
26.95
19.28
19.13
18.52
13.81
-3.17
CO_2Bu^n
TBDMSi
3cb
Chemical Shift (ppm)

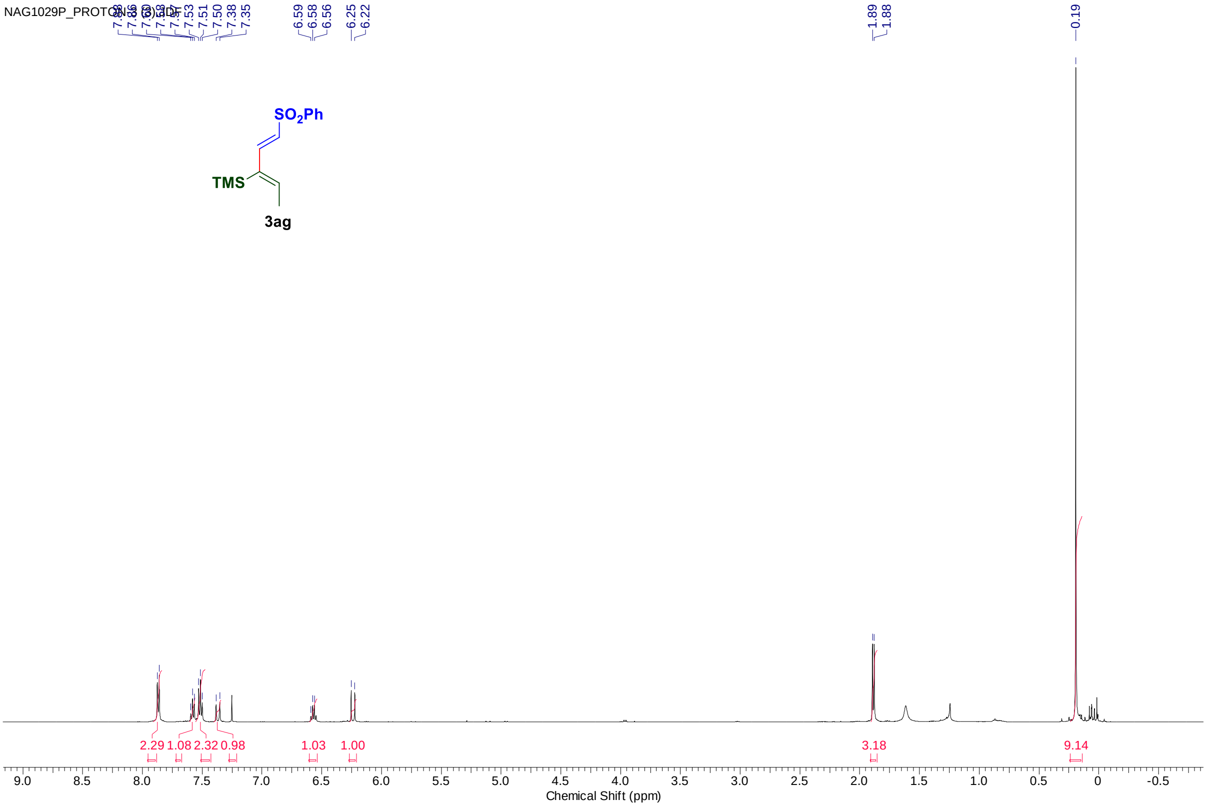
NAG1029P_PROTON-3 (3).JDF
7.53
7.51
7.50
7.38
7.35
6.59
6.58
6.56
6.25
6.22
1.89
1.88
0.19
SO2Ph
TMS
3ag
2.29 1.08 2.32 0.98
1.03
1.00
3.18
9.14
9.0 8.5 8.0 7.5 7.0 6.5 6.0 5.5 5.0 4.5 4.0 3.5 3.0 2.5 2.0 1.5 1.0 0.5 0 -0.5
Chemical Shift (ppm)

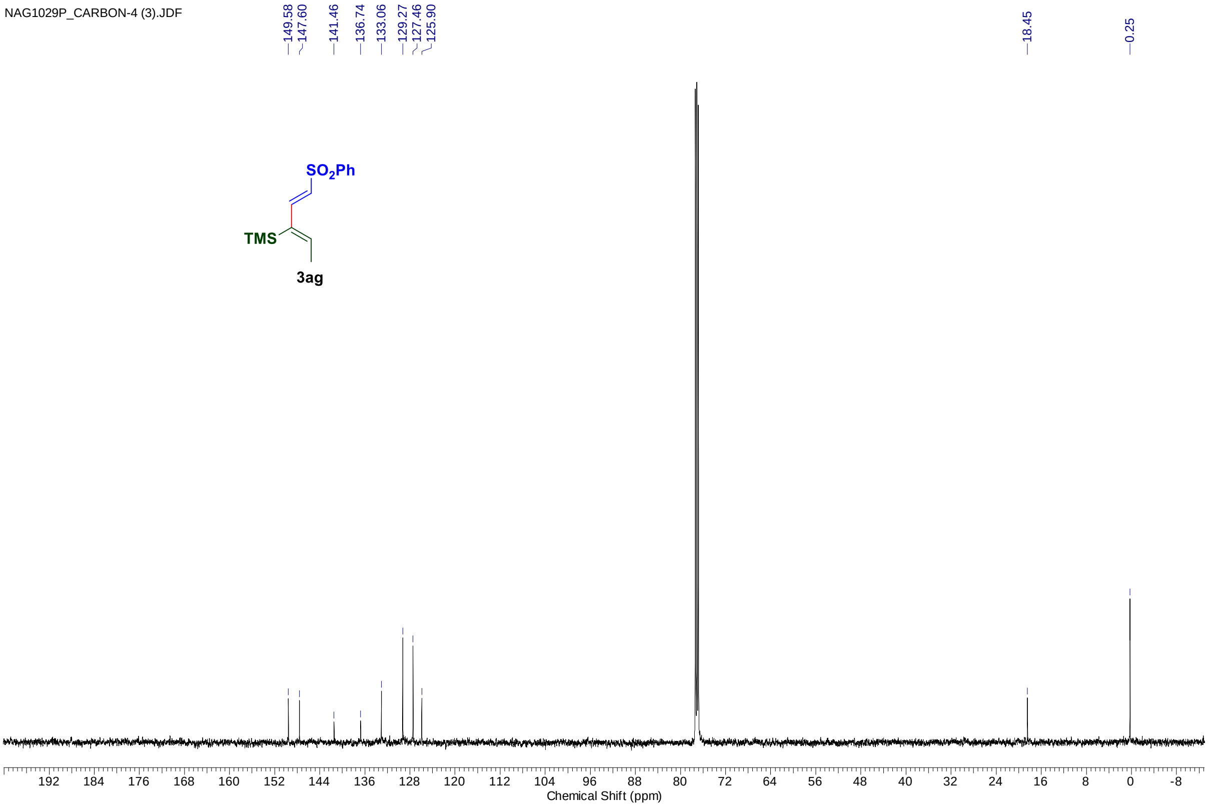
NAG1029P_CARBON-4 (3).JDF
149.58
147.60
141.46
136.74
133.06
129.27
127.46
125.90
18.45
0.25
SO2Ph
TMS
3ag
192 184 176 168 160 152 144 136 128 120 112 104 96 88 80 72 64 56 48 40 32 24 16 8 0 -8
Chemical Shift (ppm)

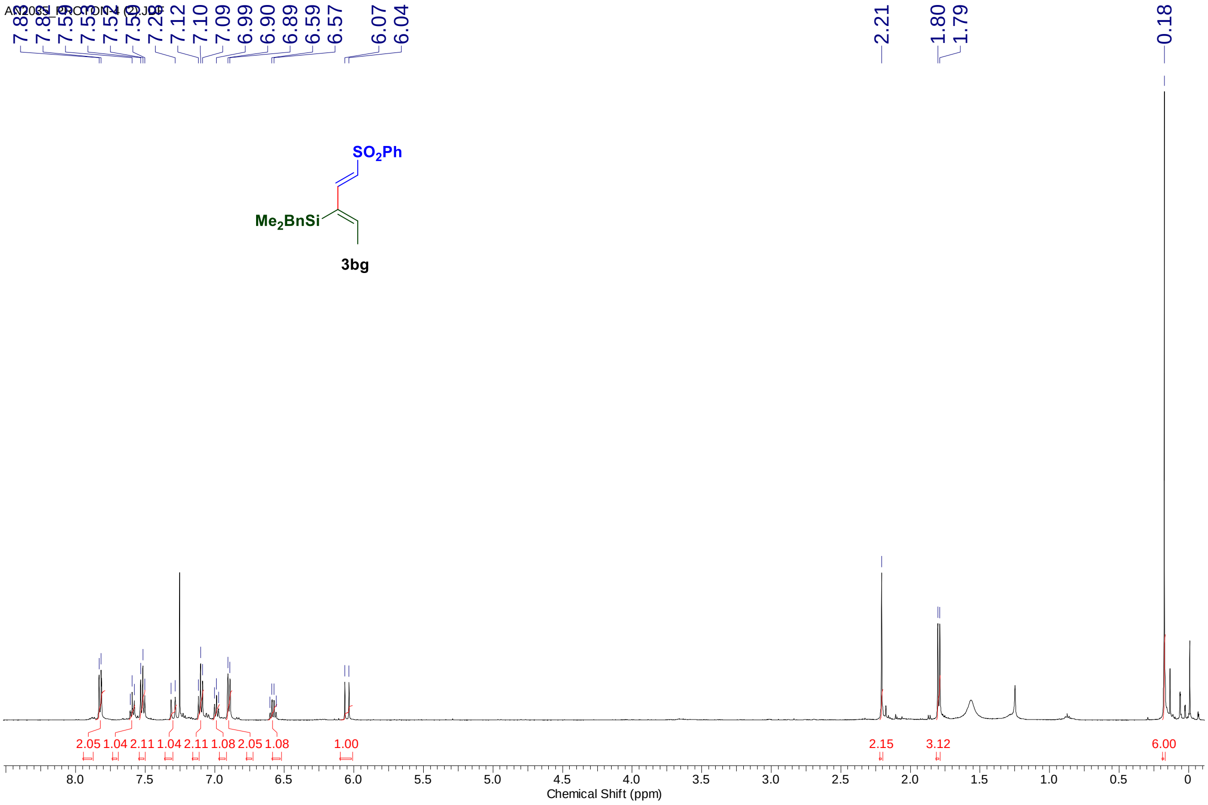
7.29
7.12
7.10
7.09
6.99
6.90
6.89
6.59
6.57
6.07
6.04
2.21
1.80
1.79
0.18
SO2Ph
Me2BnSi
3bg
2.05 1.04 2.11 1.04 2.11 1.08 2.05 1.08
1.00
2.15
3.12
6.00
Chemical Shift (ppm)

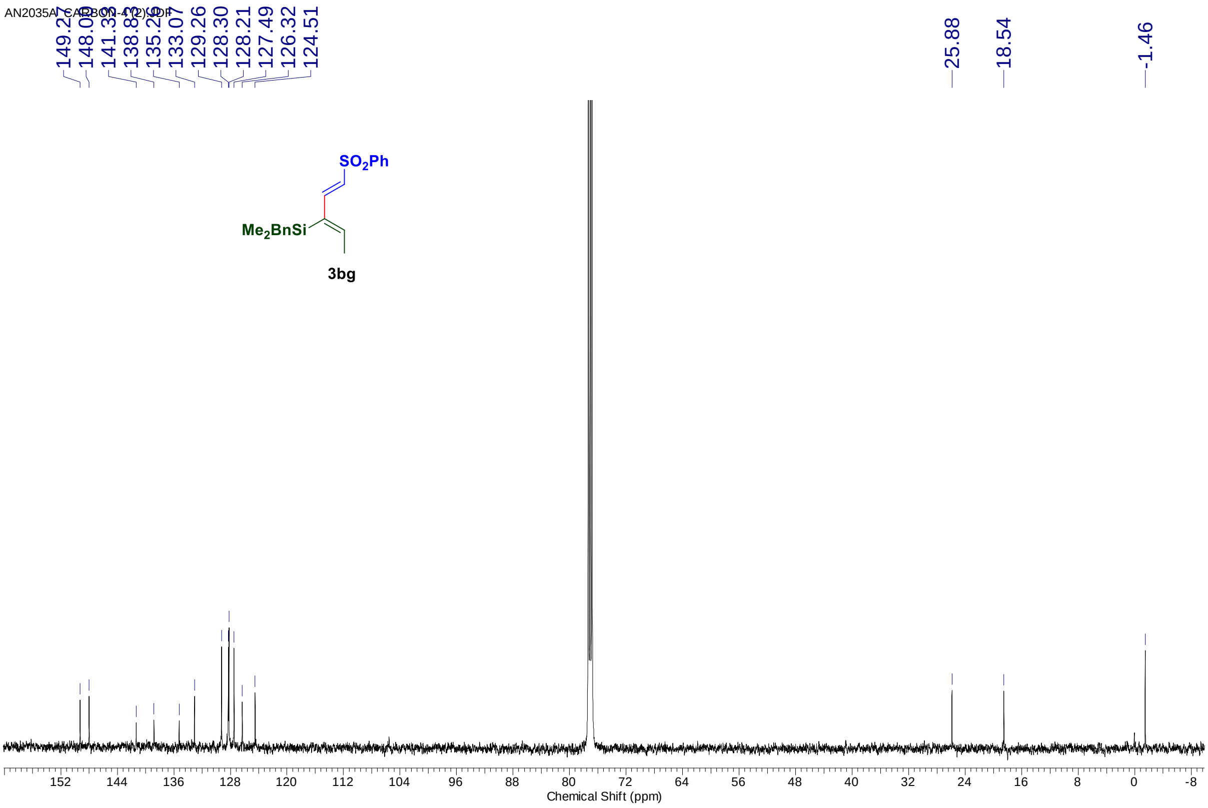
133.07
129.26
128.30
128.21
127.49
126.32
124.51
25.88
18.54
-1.46
SO2Ph
Me2BnSi
3bg
Chemical Shift (ppm)

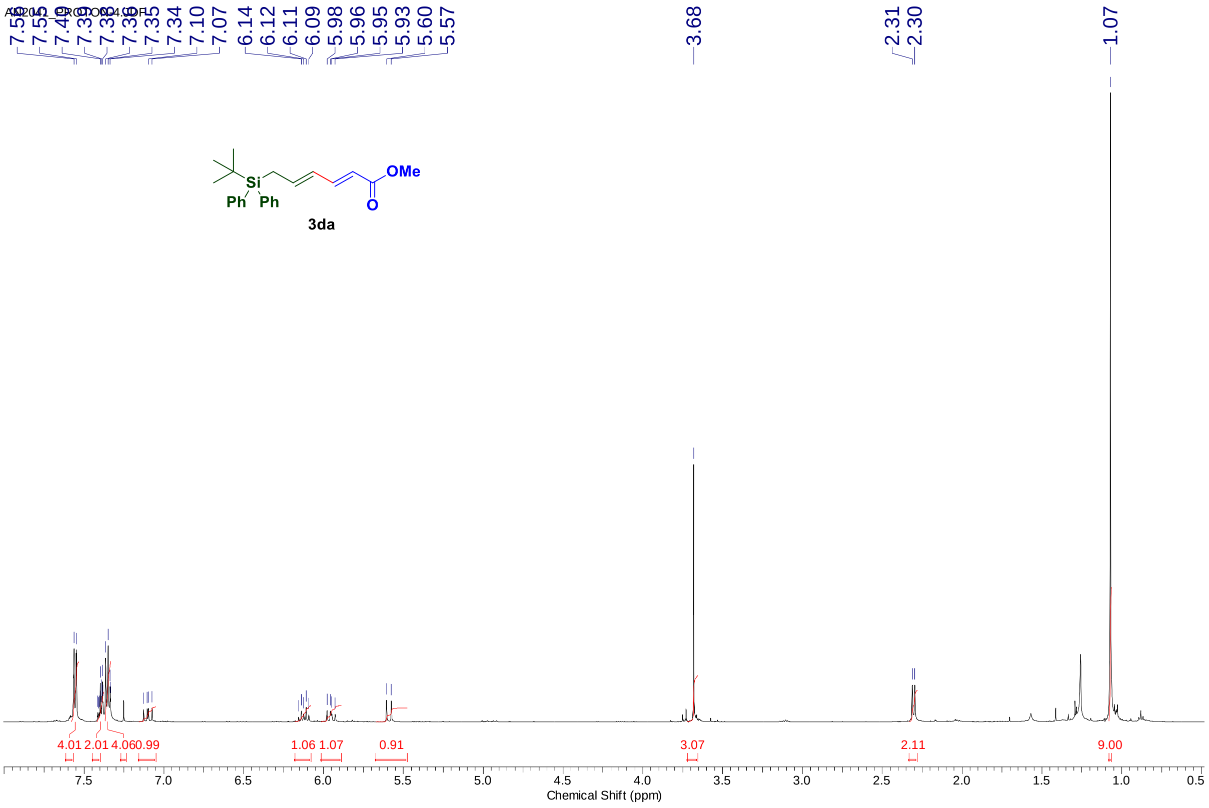
7.49
7.36
7.35
7.34
7.10
7.07
6.14
6.12
6.11
6.09
5.98
5.96
5.95
5.93
5.60
5.57
3.68
2.31
2.30
1.07
Si
Ph
Ph
OMe
O
3da
4.01
2.01
4.06
0.99
1.06
1.07
0.91
3.07
2.11
9.00
Chemical Shift (ppm)

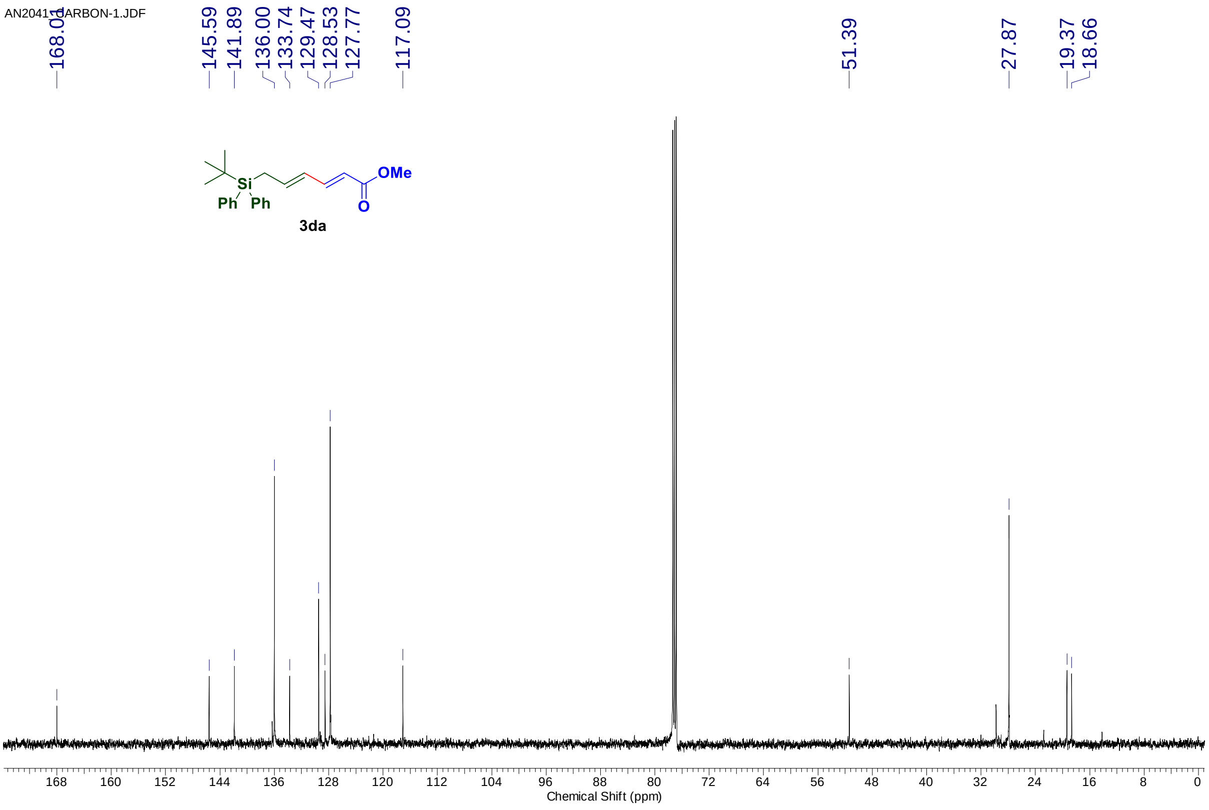
145.59
141.89
136.00
133.74
129.47
128.53
127.77
117.09
51.39
27.87
19.37
18.66
Si
Ph
Ph
OMe
O
3da
Chemical Shift (ppm)

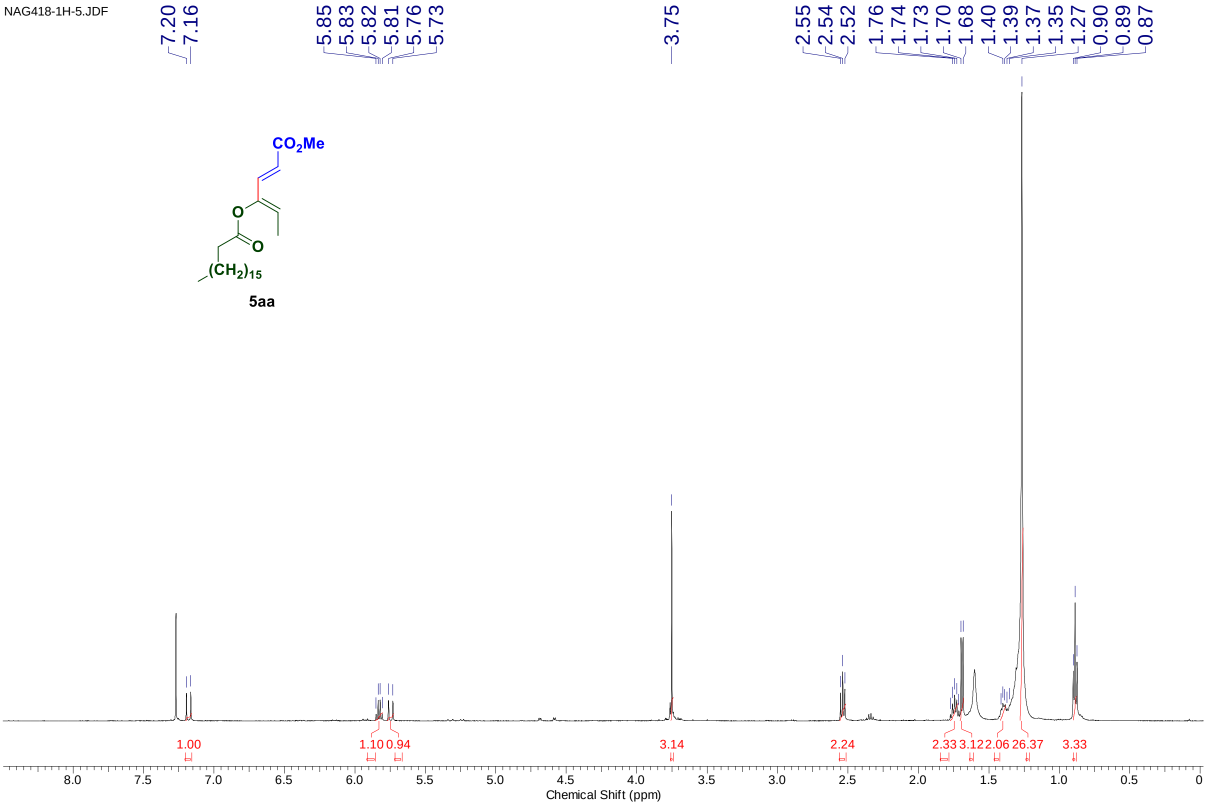
NAG418-1H-5.JDF
7.20
7.16
5.85
5.83
5.82
5.81
5.76
5.73
3.75
2.55
2.54
2.52
1.76
1.74
1.73
1.70
1.68
1.40
1.39
1.37
1.35
1.27
0.90
0.89
0.87
CO2Me
O
O
(CH2)15
5aa
1.00
1.10
0.94
3.14
2.24
2.33
3.12
2.06
26.37
3.33
Chemical Shift (ppm)

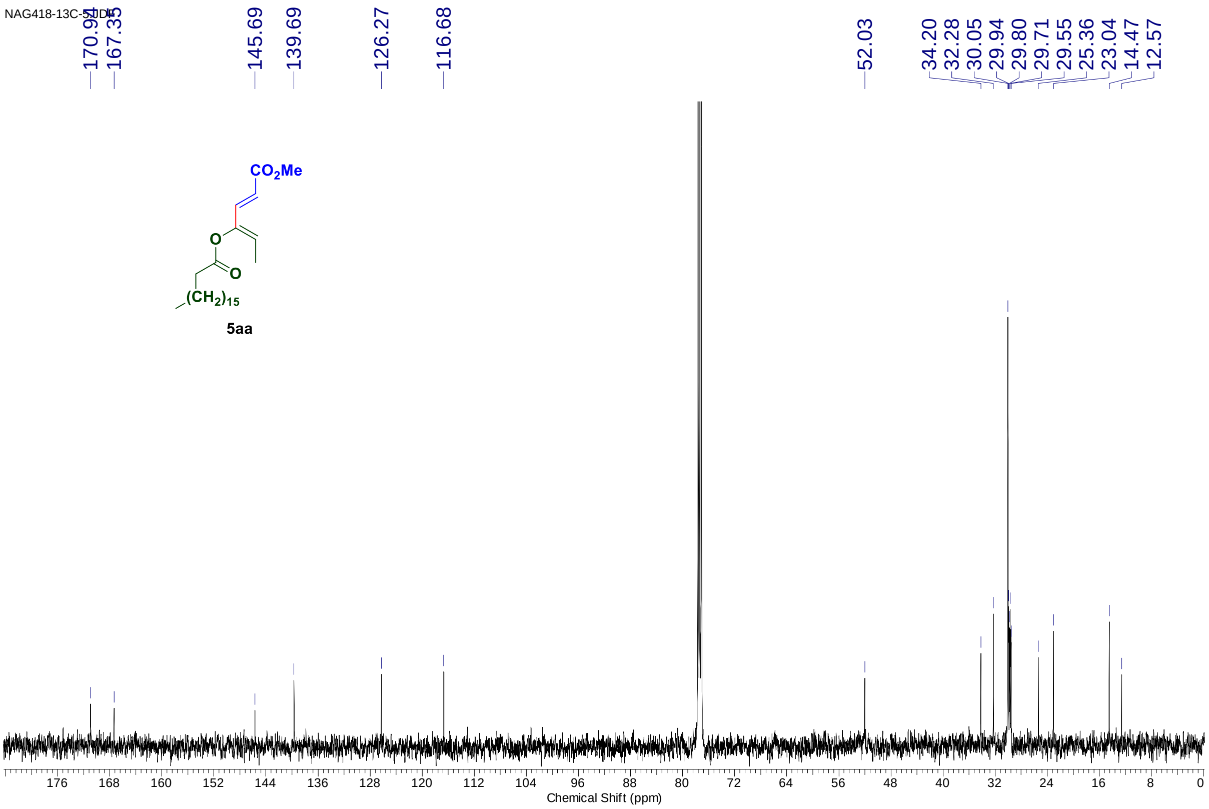
NAG418-13C-5.JDF
170.91
167.35
145.69
139.69
126.27
116.68
52.03
34.20
32.28
30.05
29.94
29.80
29.71
29.55
25.36
23.04
14.47
12.57
CO2Me
O
O
(CH2)15
5aa
Chemical Shift (ppm)

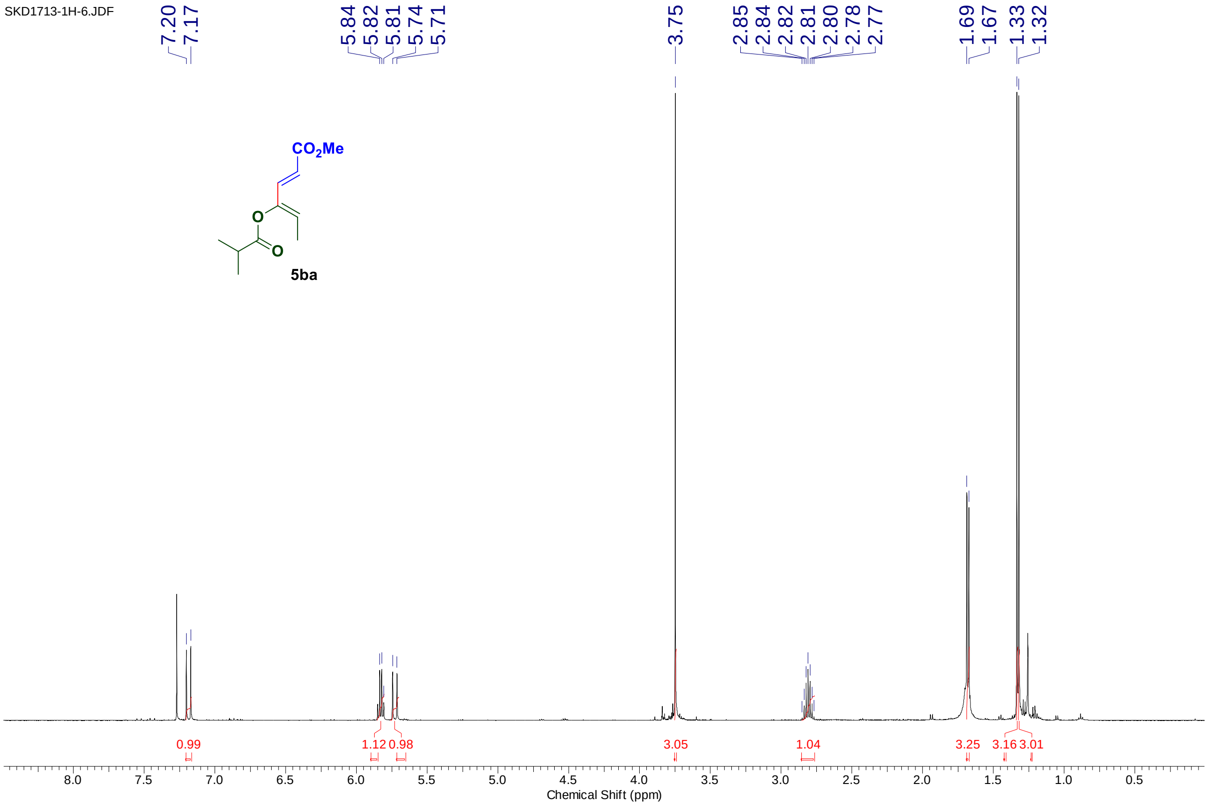
SKD1713-1H-6.JDF
7.20
7.17
5.84
5.82
5.81
5.74
5.71
3.75
2.85
2.84
2.82
2.81
2.80
2.78
2.77
1.69
1.67
1.33
1.32
CO2Me
O
O
5ba
0.99
1.12
0.98
3.05
1.04
3.25
3.16
3.01
8.0
7.5
7.0
6.5
6.0
5.5
5.0
4.5
4.0
3.5
3.0
2.5
2.0
1.5
1.0
0.5
Chemical Shift (ppm)

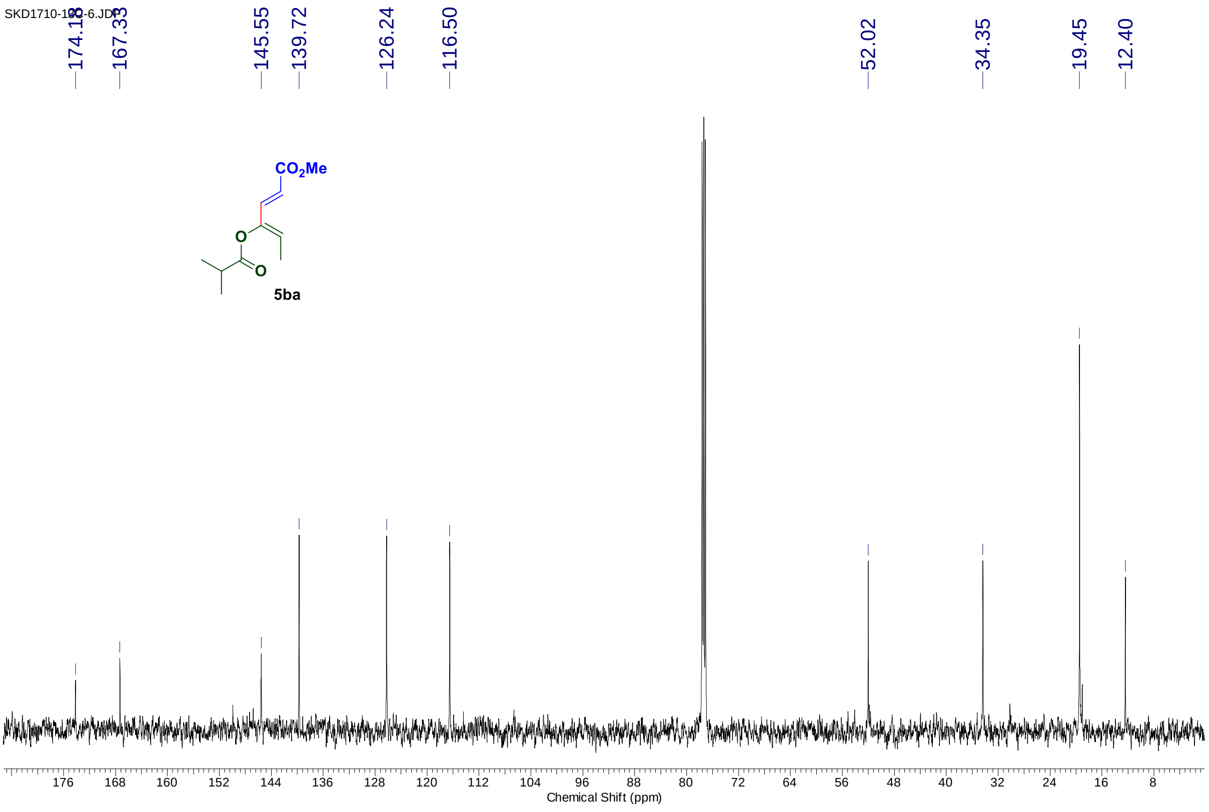
145.55
139.72
126.24
116.50
52.02
34.35
19.45
12.40
CO2Me
O
O
5ba
176
168
160
152
144
136
128
120
112
104
96
88
80
72
64
56
48
40
32
24
16
8
Chemical Shift (ppm)

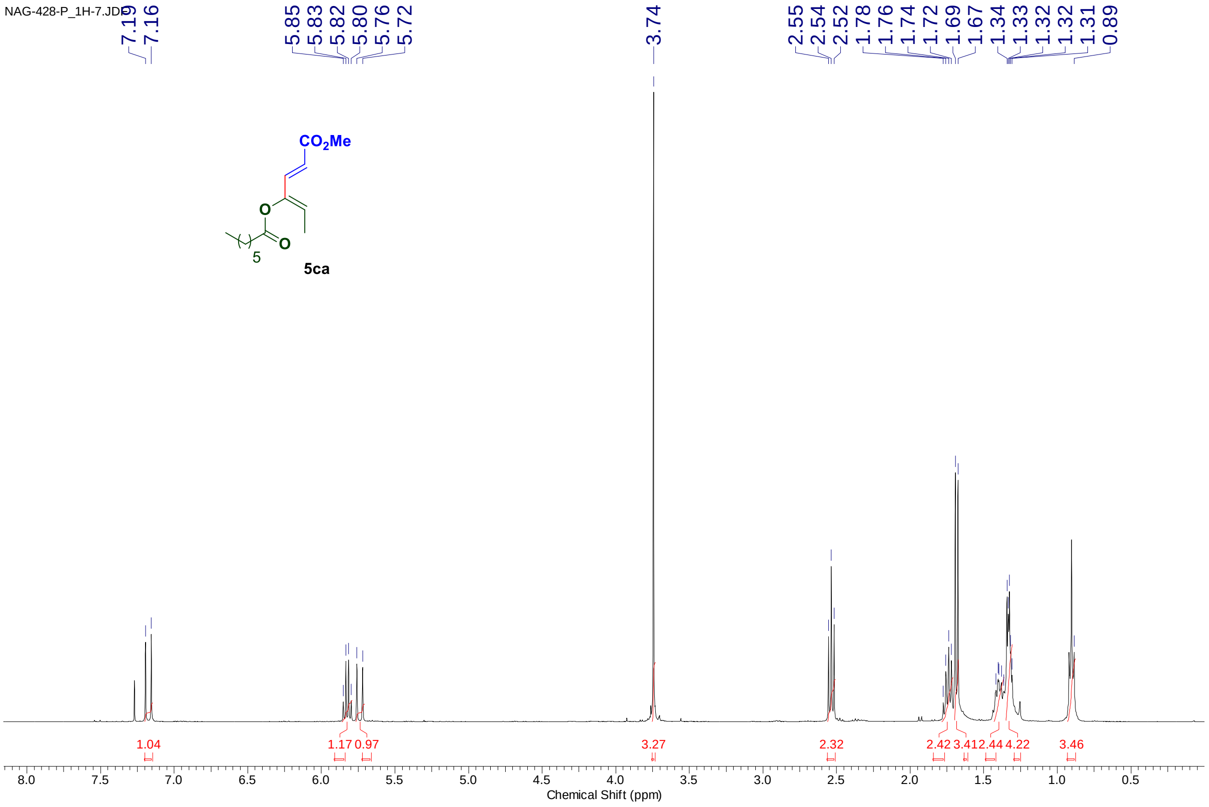
NAG-428-P_1H-7.JDF
7.19
7.16
5.85
5.83
5.82
5.80
5.76
5.72
3.74
2.55
2.54
2.52
1.78
1.76
1.74
1.72
1.69
1.67
1.34
1.33
1.32
1.32
1.31
0.89
CO2Me
O
O
5
5ca
1.04
1.17
0.97
3.27
2.32
2.42
3.41
2.44
4.22
3.46
Chemical Shift (ppm)

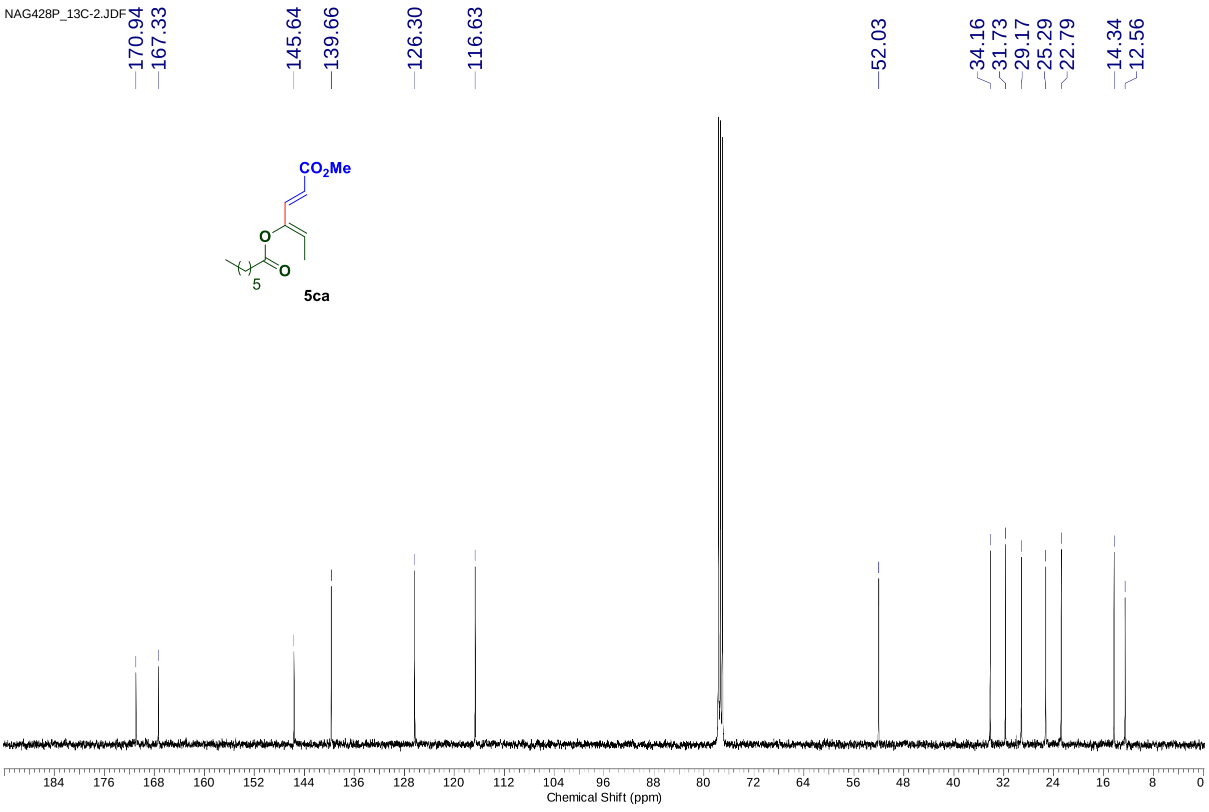
NAG428P_13C-2.JDF
170.94
167.33
145.64
139.66
126.30
116.63
52.03
34.16
31.73
29.17
25.29
22.79
14.34
12.56
CO2Me
O
O
5
5ca
Chemical Shift (ppm)

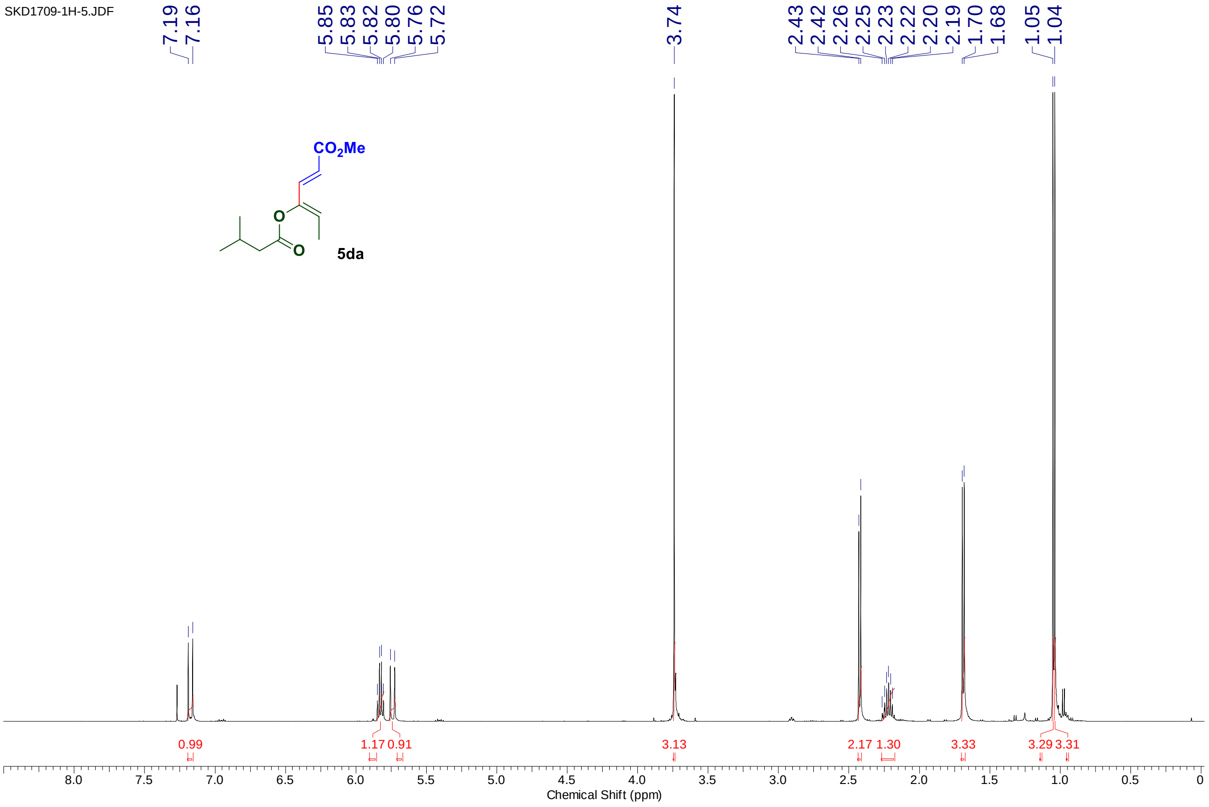
SKD1709-1H-5.JDF
7.19
7.16
5.85
5.83
5.82
5.80
5.76
5.72
3.74
2.43
2.42
2.26
2.25
2.23
2.22
2.20
2.19
1.70
1.68
1.05
1.04
CO2Me
O
O
5da
0.99
1.17
0.91
3.13
2.17
1.30
3.33
3.29
3.31
8.0 7.5 7.0 6.5 6.0 5.5 5.0 4.5 4.0 3.5 3.0 2.5 2.0 1.5 1.0 0.5 0
Chemical Shift (ppm)

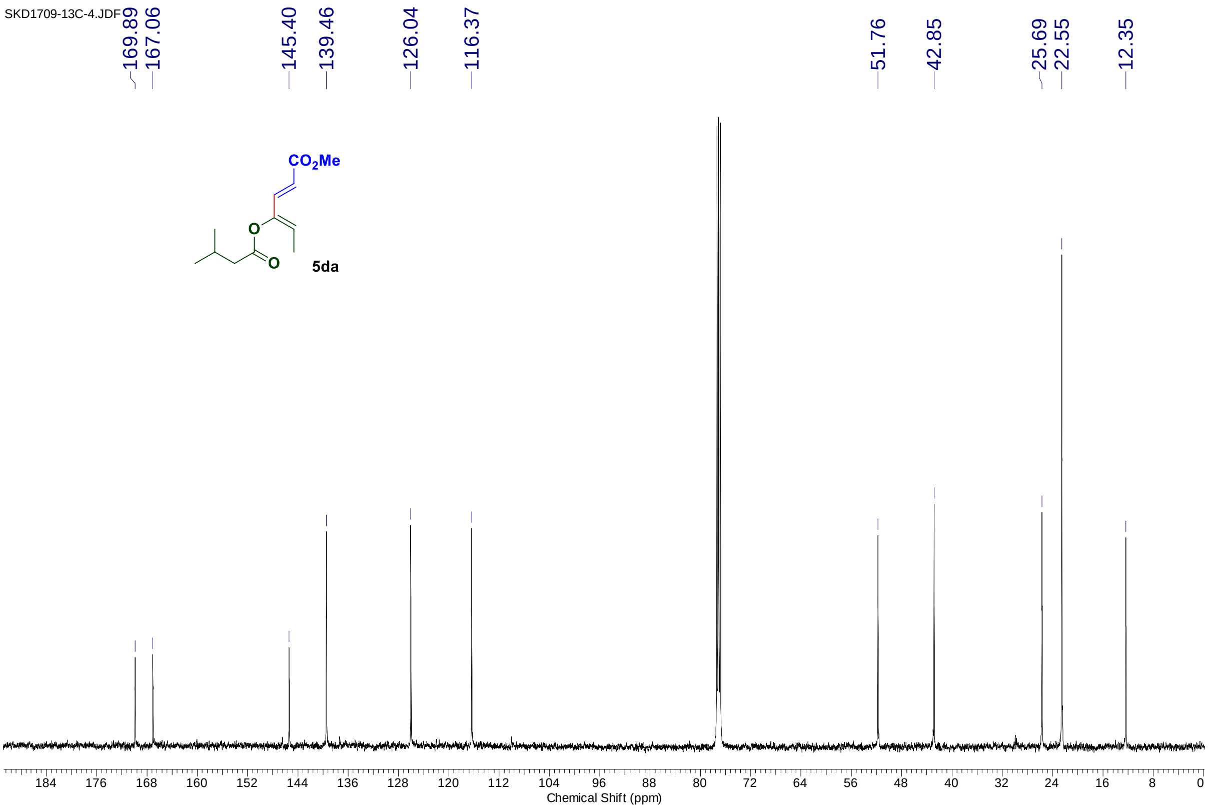
SKD1709-13C-4.JDF
169.89
167.06
145.40
139.46
126.04
116.37
51.76
42.85
25.69
22.55
12.35
CO2Me
O
O
5da
184 176 168 160 152 144 136 128 120 112 104 96 88 80 72 64 56 48 40 32 24 16 8 0
Chemical Shift (ppm)

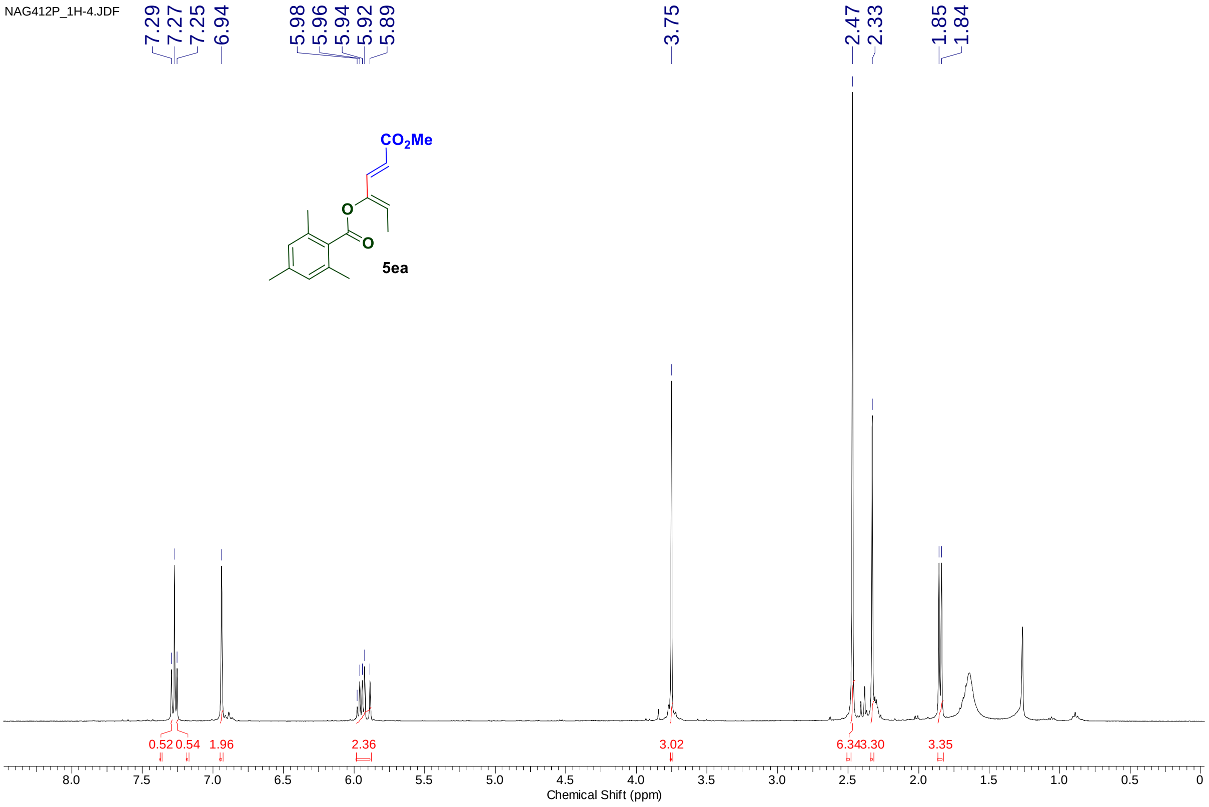
NAG412P_1H-4.JDF
7.29
7.27
7.25
6.94
5.98
5.96
5.94
5.92
5.89
3.75
2.47
2.33
1.85
1.84
CO2Me
O
O
5ea
0.52
0.54
1.96
2.36
3.02
6.34
3.30
3.35
Chemical Shift (ppm)

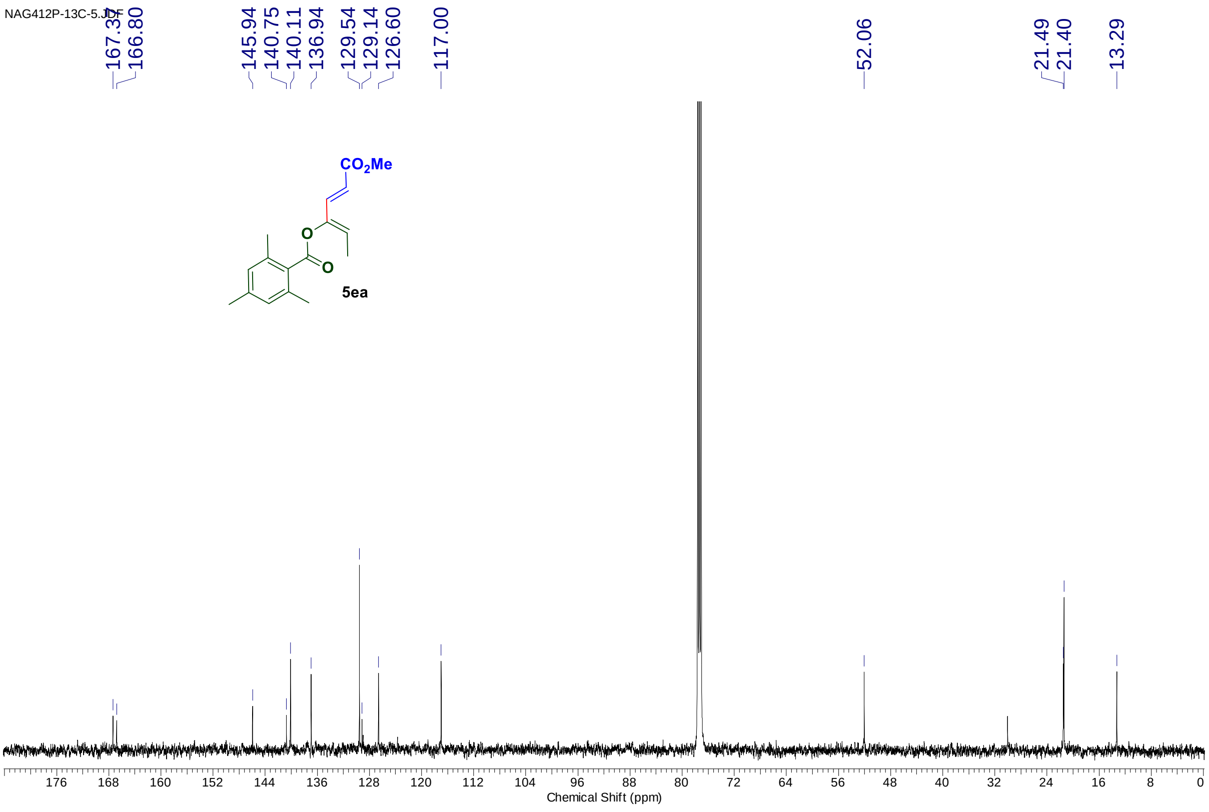
NAG412P-13C-5.JDF
167.37
166.80
145.94
140.75
140.11
136.94
129.54
129.14
126.60
117.00
52.06
21.49
21.40
13.29
CO2Me
O
O
5ea
Chemical Shift (ppm)

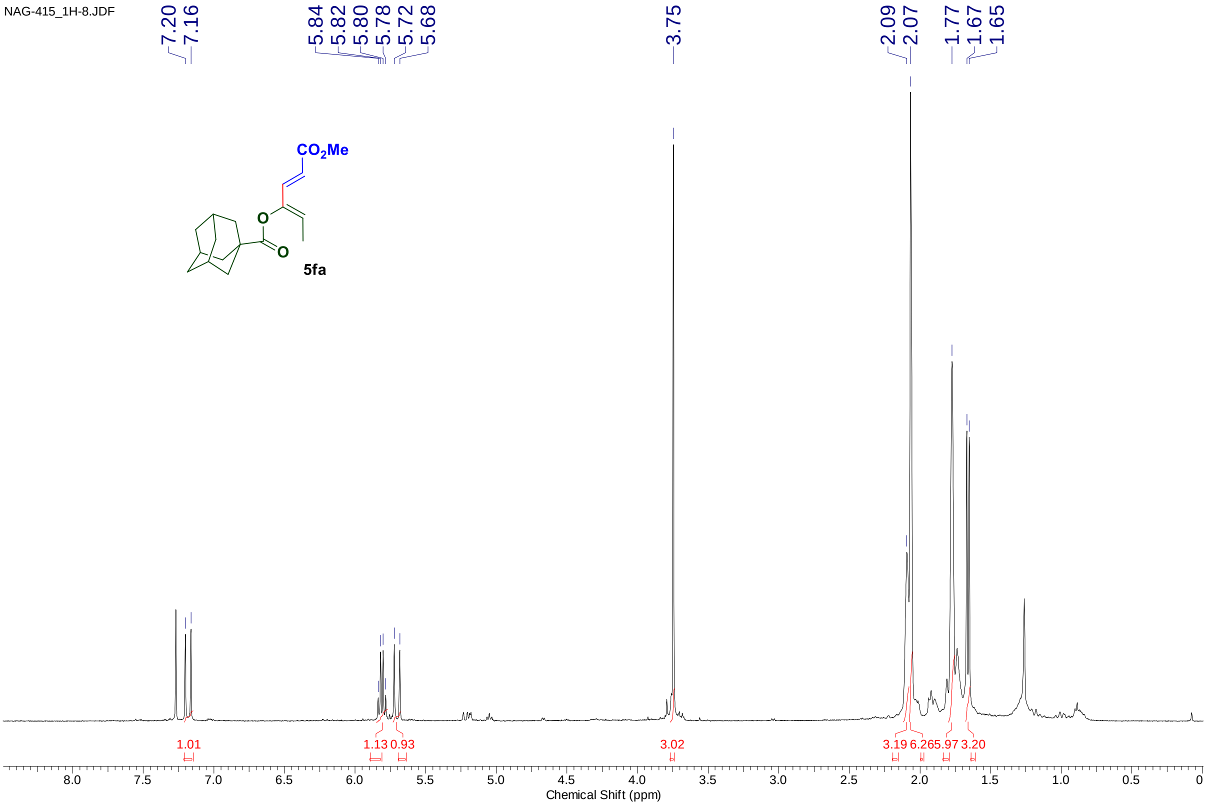

NAG-415_1H-8.JDF
7.20
7.16
5.84
5.82
5.80
5.78
5.72
5.68
3.75
2.09
2.07
1.77
1.67
1.65
CO2Me
O
O
5fa
1.01
1.13
0.93
3.02
3.19
6.26
5.97
3.20
8.0 7.5 7.0 6.5 6.0 5.5 5.0 4.5 4.0 3.5 3.0 2.5 2.0 1.5 1.0 0.5 0
Chemical Shift (ppm)

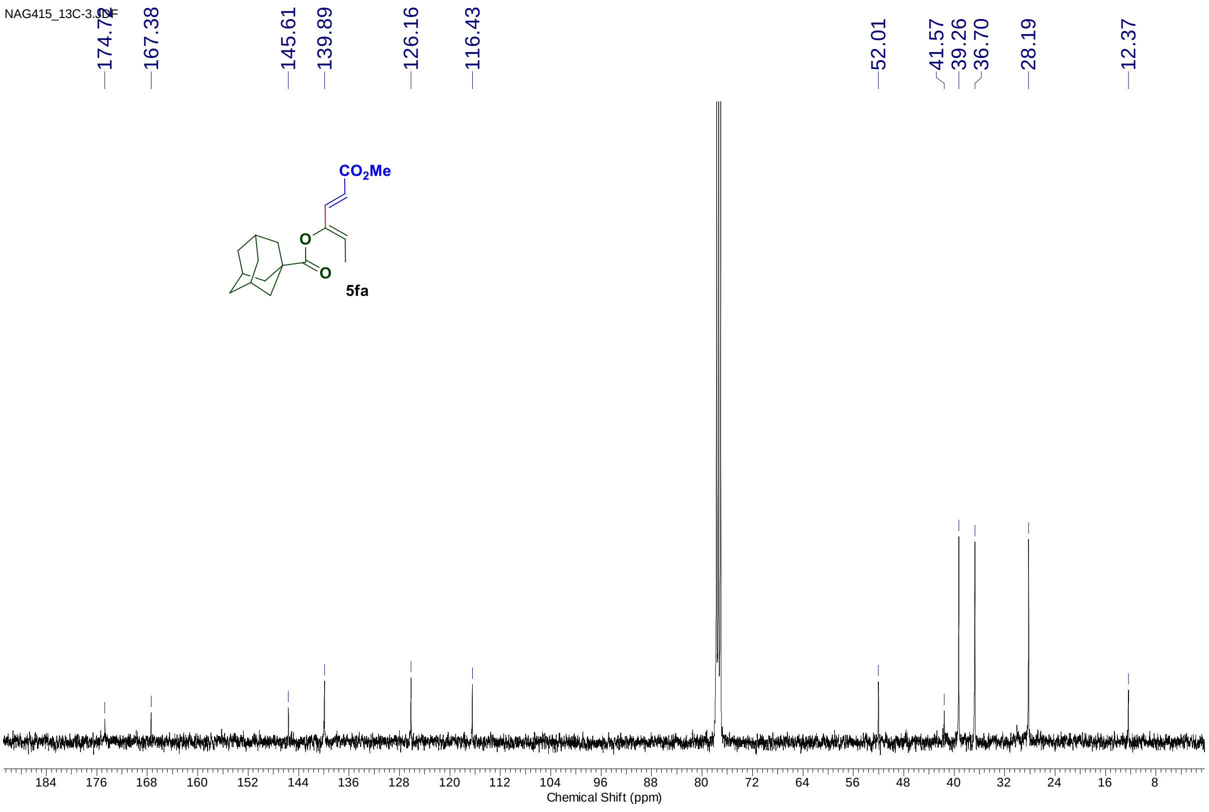

NAG415_13C-3.JDF
174.72
167.38
145.61
139.89
126.16
116.43
52.01
41.57
39.26
36.70
28.19
12.37
CO2Me
O
O
5fa
184 176 168 160 152 144 136 128 120 112 104 96 88 80 72 64 56 48 40 32 24 16 8
Chemical Shift (ppm)

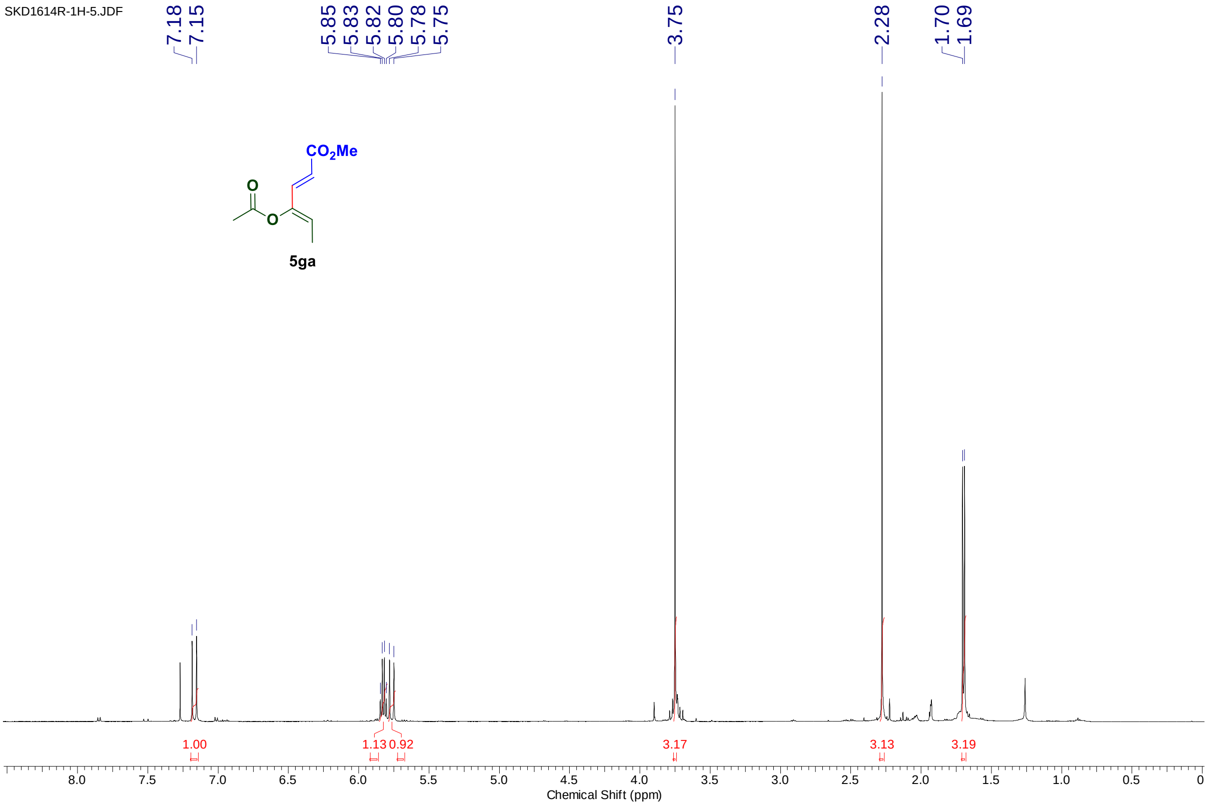
SKD1614R-1H-5.JDF
7.18
7.15
5.85
5.83
5.82
5.80
5.78
5.75
3.75
2.28
1.70
1.69
CO2Me
O
O
5ga
1.00
1.13
0.92
3.17
3.13
3.19
8.0
7.5
7.0
6.5
6.0
5.5
5.0
4.5
4.0
3.5
3.0
2.5
2.0
1.5
1.0
0.5
0
Chemical Shift (ppm)

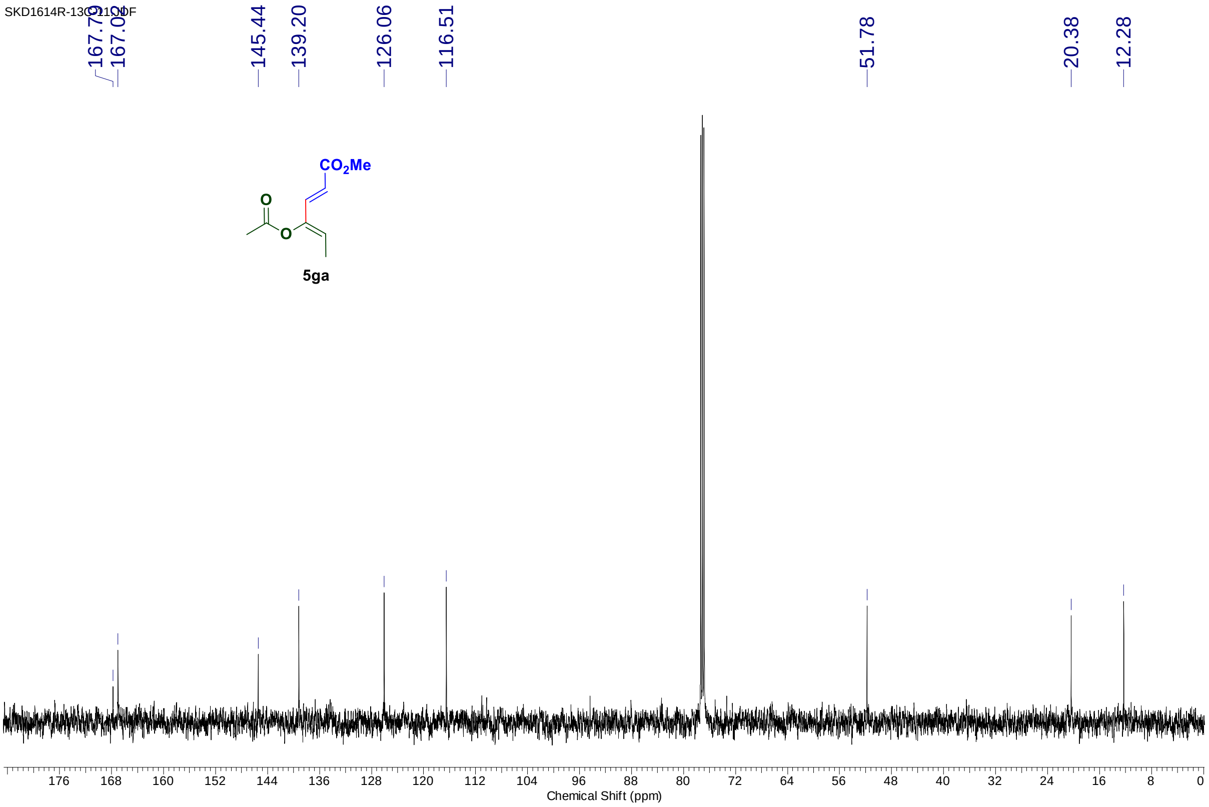
SKD1614R-13C-1.JDF
167.79
167.02
145.44
139.20
126.06
116.51
51.78
20.38
12.28
CO2Me
O
O
5ga
176
168
160
152
144
136
128
120
112
104
96
88
80
72
64
56
48
40
32
24
16
8
0
Chemical Shift (ppm)

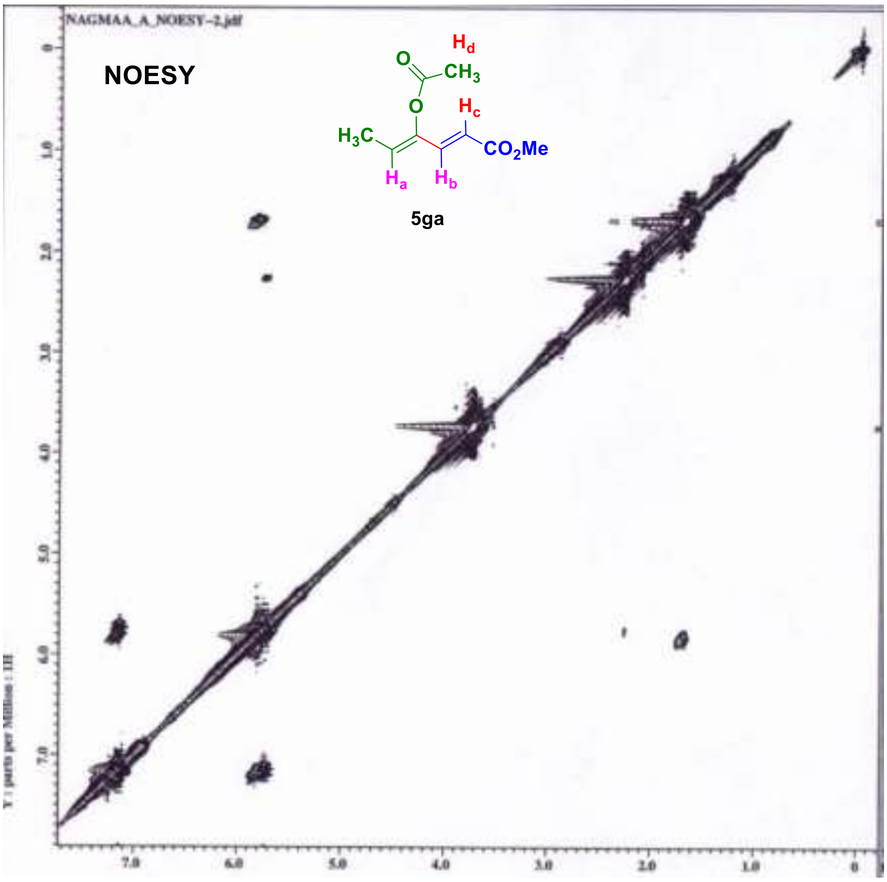
NAGMAA_A_NOESY-2.jdf
NOESY
5ga
H3C
CO2Me
Ha
Hb
Hc
Hd
CH3
O
7.0
6.0
5.0
4.0
3.0
2.0
1.0
0

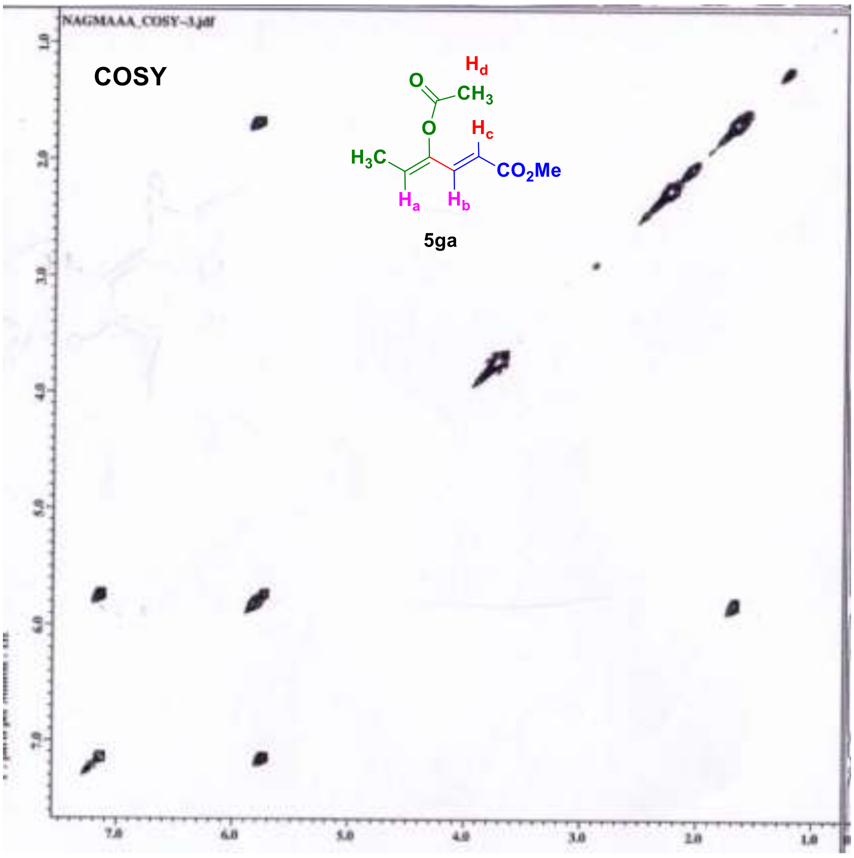
NAGMAA_COSY-3.jdf
COSY
5ga
H3C
CO2Me
Ha
Hb
Hc
Hd
CH3
O
7.0
6.0
5.0
4.0
3.0
2.0
1.0

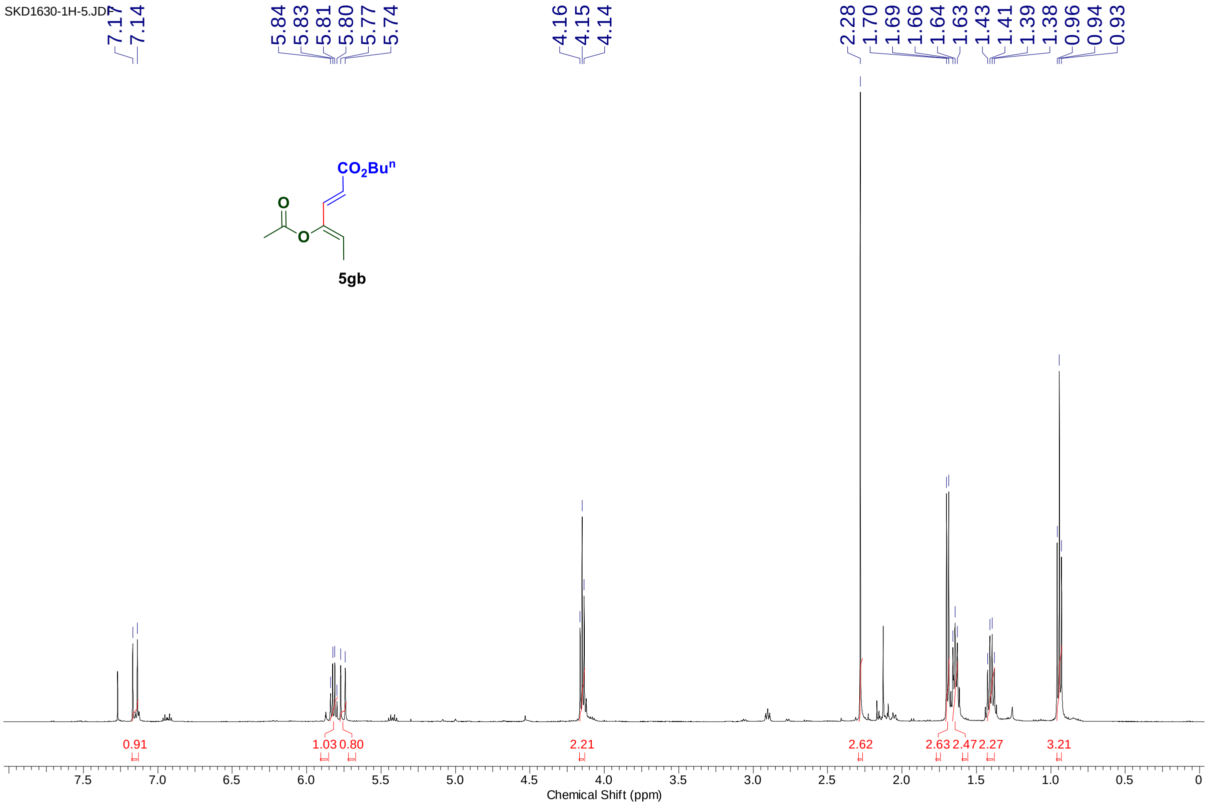
SKD1630-1H-5.JDF
7.17
7.14
5.84
5.83
5.81
5.80
5.77
5.74
4.16
4.15
4.14
2.28
1.70
1.69
1.66
1.64
1.63
1.43
1.41
1.39
1.38
0.96
0.94
0.93
CO_2Bu^n
O
O
5gb
0.91
1.03
0.80
2.21
2.62
2.63
2.47
2.27
3.21
Chemical Shift (ppm)

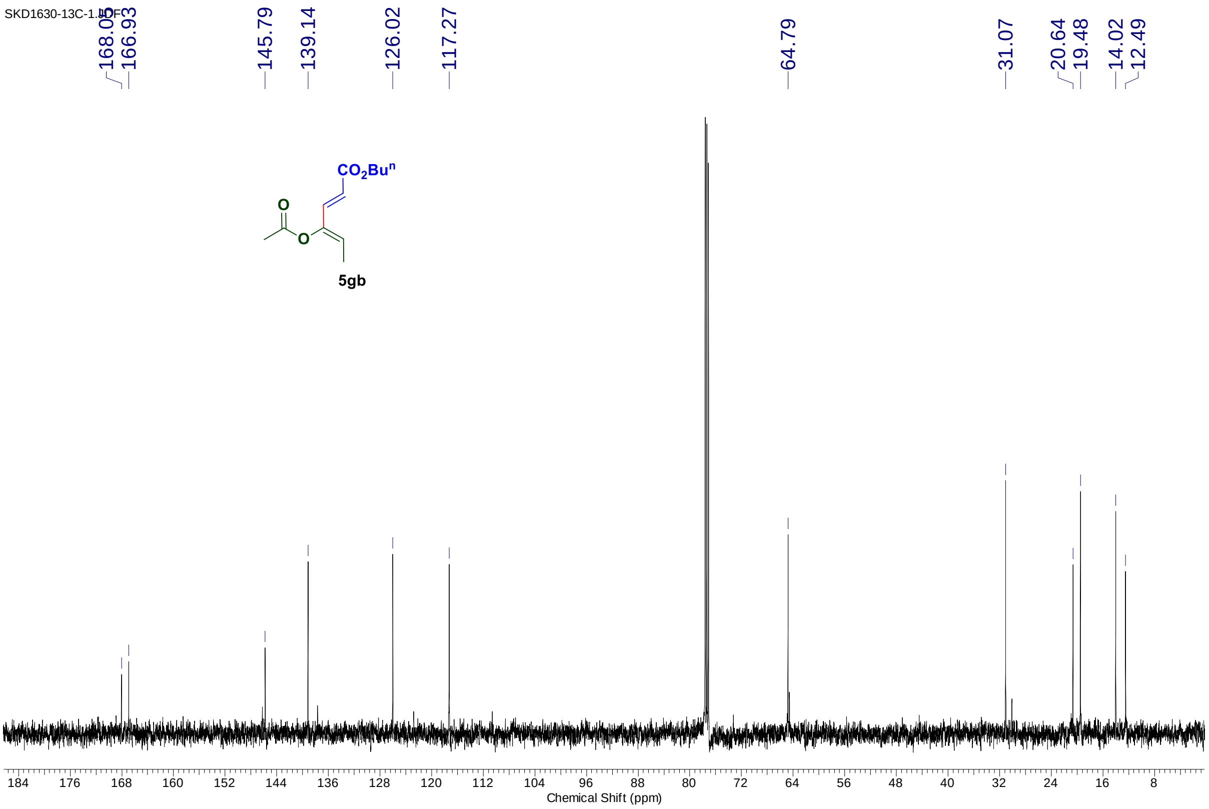
SKD1630-13C-1.JDF
168.05
166.93
145.79
139.14
126.02
117.27
64.79
31.07
20.64
19.48
14.02
12.49
CO_2Bu^n
O
O
5gb
Chemical Shift (ppm)

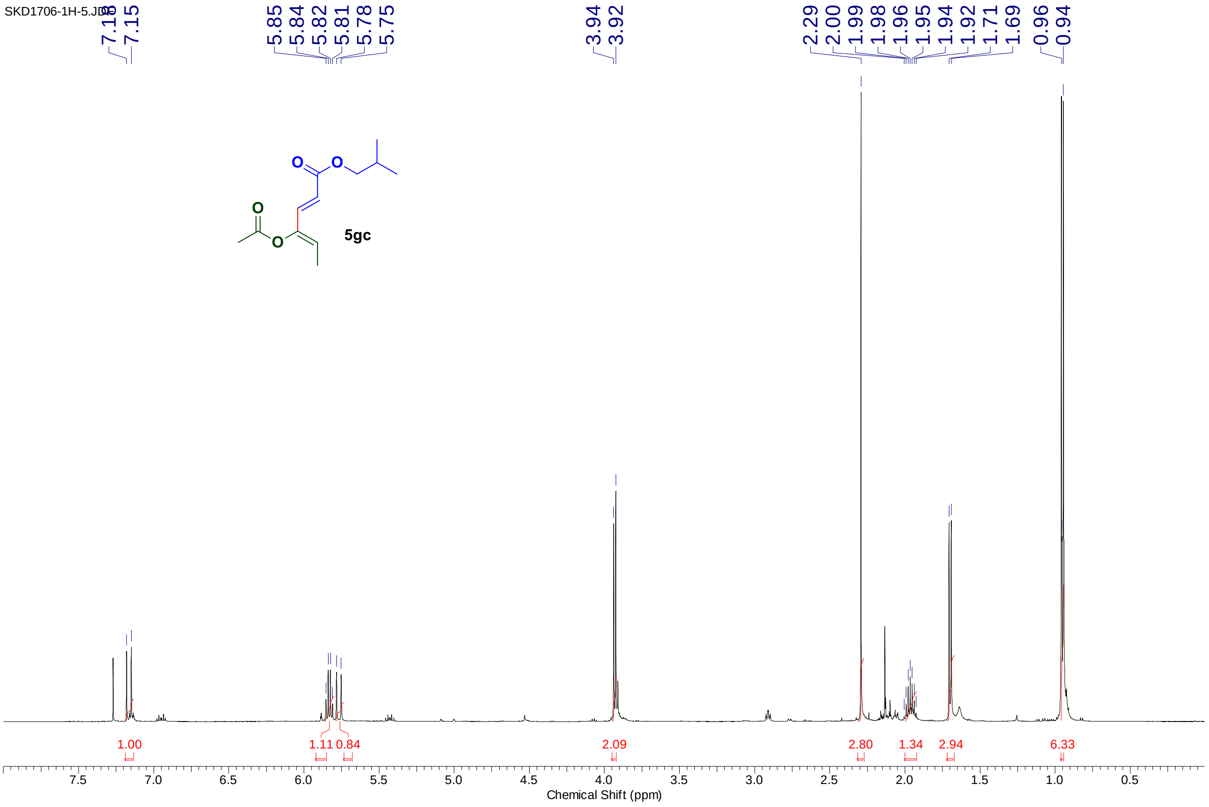
SKD1706-1H-5.JDF
7.18
7.15
5.85
5.84
5.82
5.81
5.78
5.75
3.94
3.92
2.29
2.00
1.99
1.98
1.96
1.95
1.94
1.92
1.71
1.69
0.96
0.94
5gc
1.00
1.11
0.84
2.09
2.80
1.34
2.94
6.33
Chemical Shift (ppm)

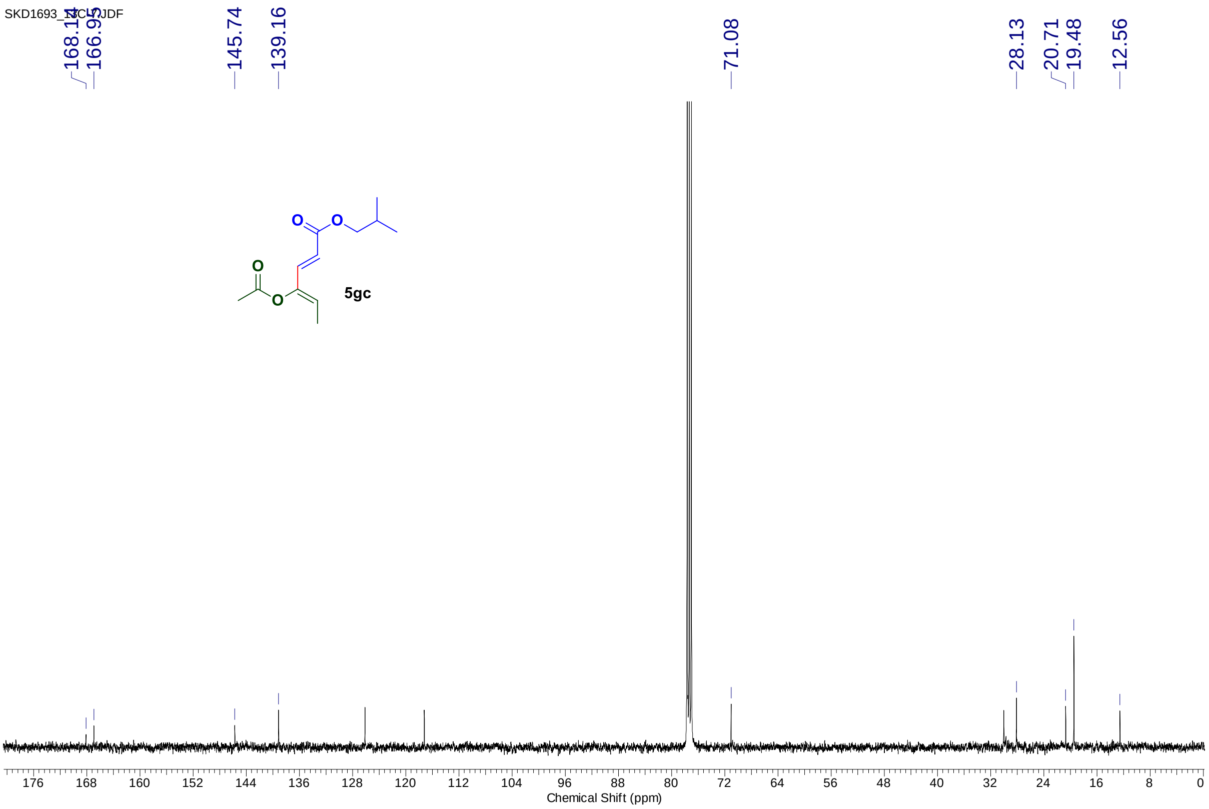
SKD1693_13C-JDF
168.14
166.95
145.74
139.16
71.08
28.13
20.71
19.48
12.56
5gc
Chemical Shift (ppm)

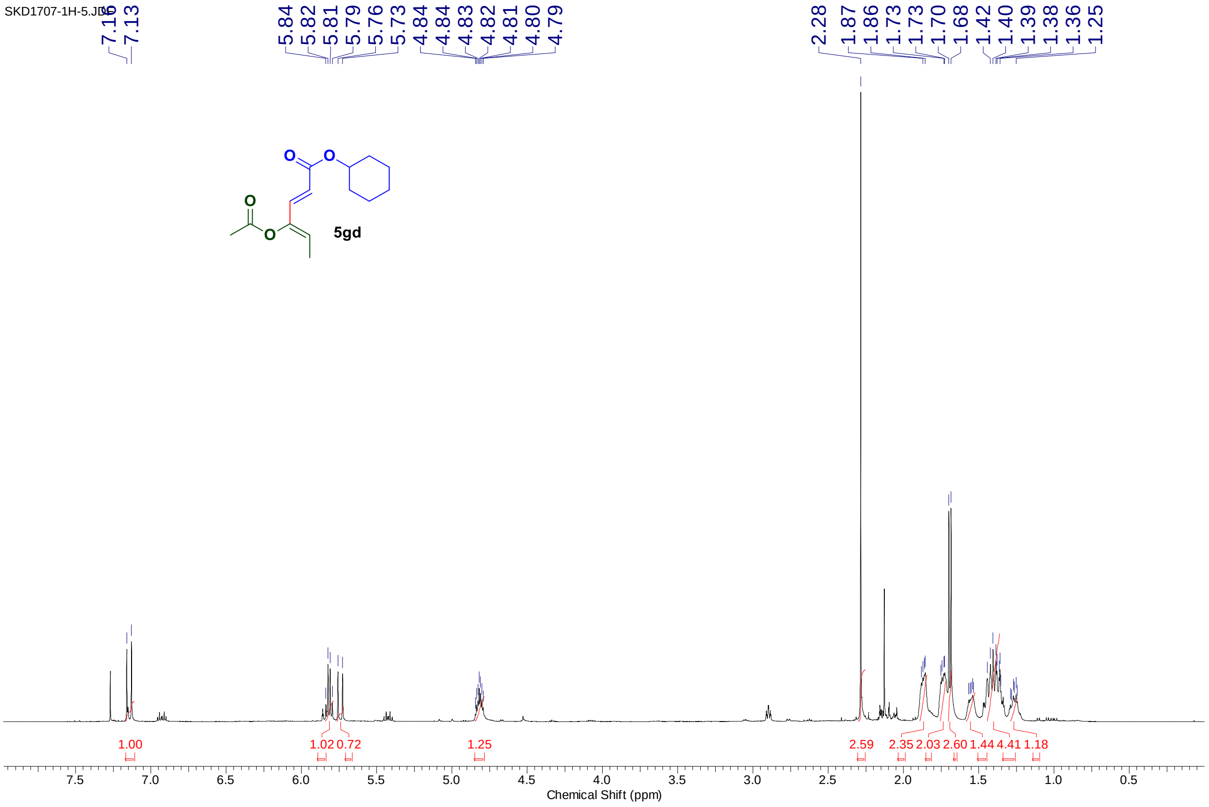
5gd
7.13
5.84 5.82 5.81 5.79 5.76 5.73 4.84 4.84 4.83 4.82 4.81 4.80 4.79
2.28 1.87 1.86 1.73 1.73 1.70 1.68 1.42 1.40 1.39 1.38 1.36 1.25
1.00 1.02 0.72 1.25 2.59 2.35 2.03 2.60 1.44 4.41 1.18
7.5 7.0 6.5 6.0 5.5 5.0 4.5 4.0 3.5 3.0 2.5 2.0 1.5 1.0 0.5
Chemical Shift (ppm)

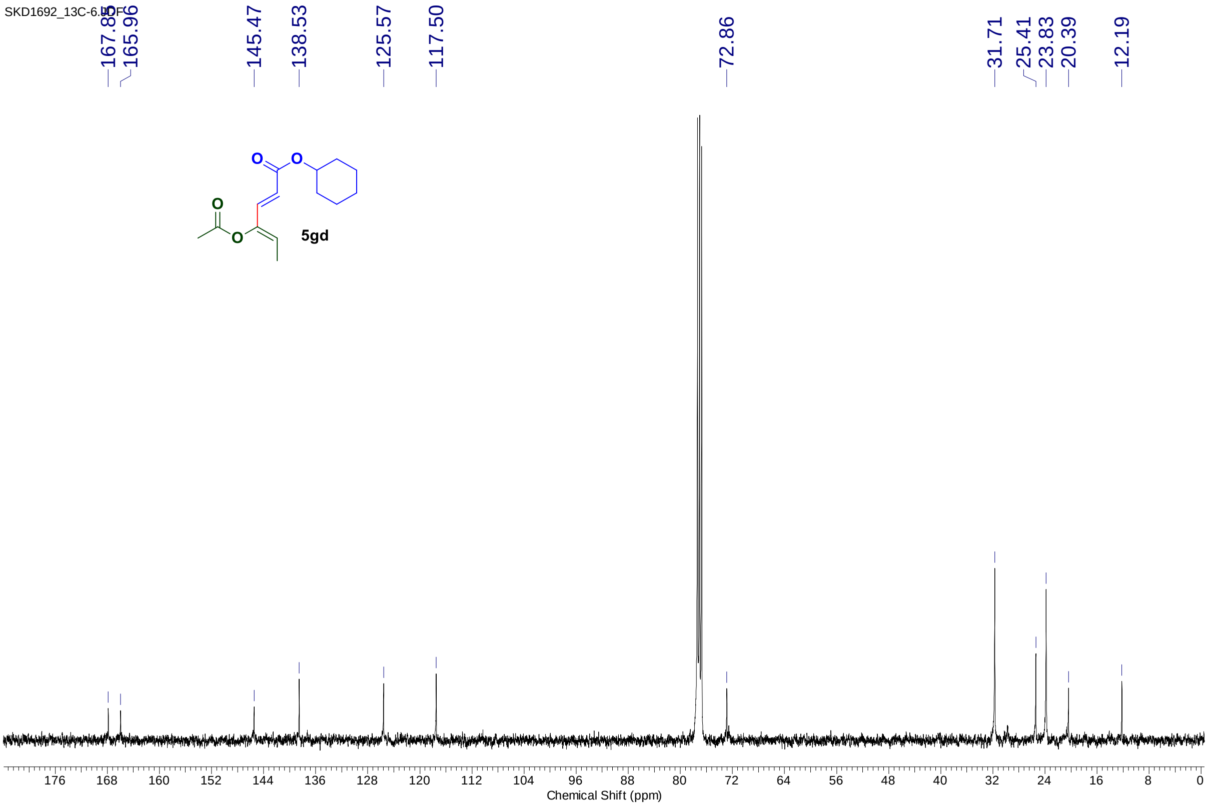
5gd
165.96 145.47 138.53 125.57 117.50 72.86 31.71 25.41 23.83 20.39 12.19
176 168 160 152 144 136 128 120 112 104 96 88 80 72 64 56 48 40 32 24 16 8 0
Chemical Shift (ppm)

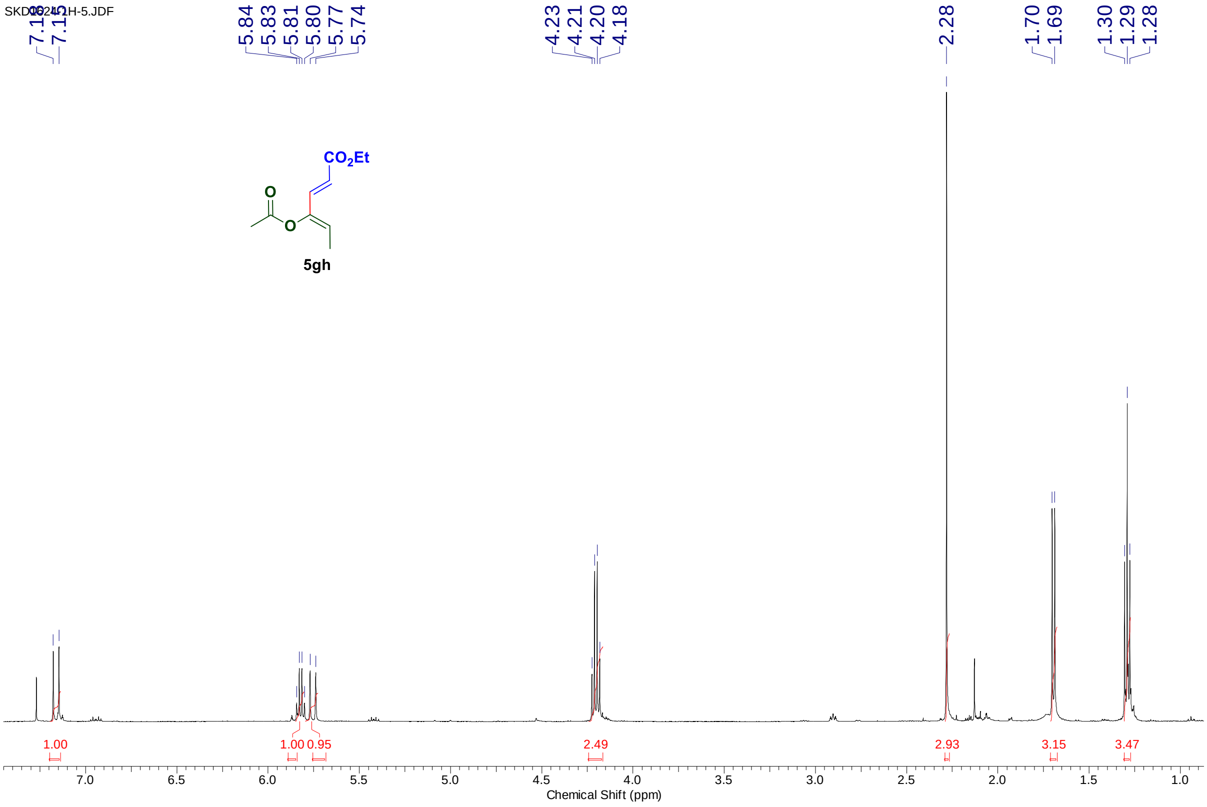
7.15
5.84
5.83
5.81
5.80
5.77
5.74
4.23
4.21
4.20
4.18
2.28
1.70
1.69
1.30
1.29
1.28
CO2Et
O
O
5gh
1.00
1.00
0.95
2.49
2.93
3.15
3.47
7.0 6.5 6.0 5.5 5.0 4.5 4.0 3.5 3.0 2.5 2.0 1.5 1.0
Chemical Shift (ppm)

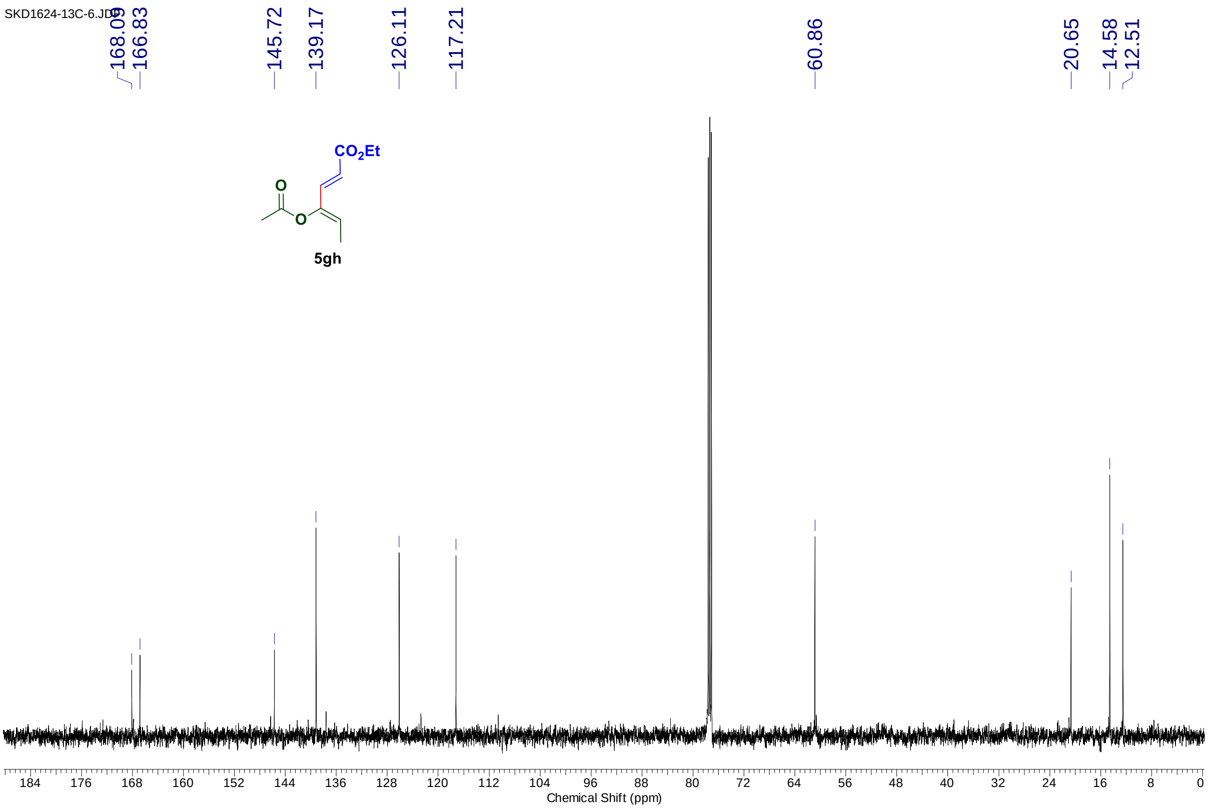
SKD1624-13C-6.JDF
166.83
145.72
139.17
126.11
117.21
60.86
20.65
14.58
12.51
CO2Et
O
O
5gh
184 176 168 160 152 144 136 128 120 112 104 96 88 80 72 64 56 48 40 32 24 16 8 0
Chemical Shift (ppm)

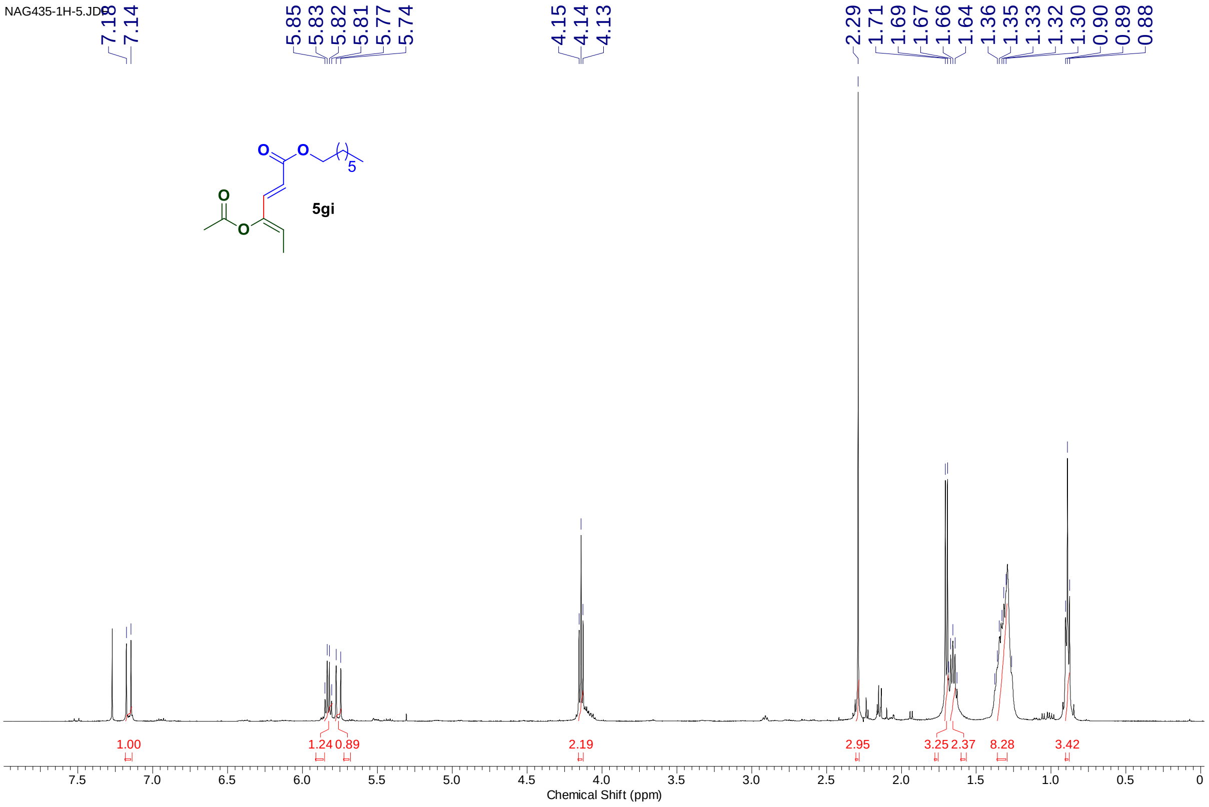
NAG435-1H-5.JDF
7.18
7.14
5.85
5.83
5.82
5.81
5.77
5.74
4.15
4.14
4.13
2.29
1.71
1.69
1.67
1.66
1.64
1.36
1.35
1.33
1.32
1.30
0.90
0.89
0.88
5gi
1.00
1.24
0.89
2.19
2.95
3.25
2.37
8.28
3.42
Chemical Shift (ppm)

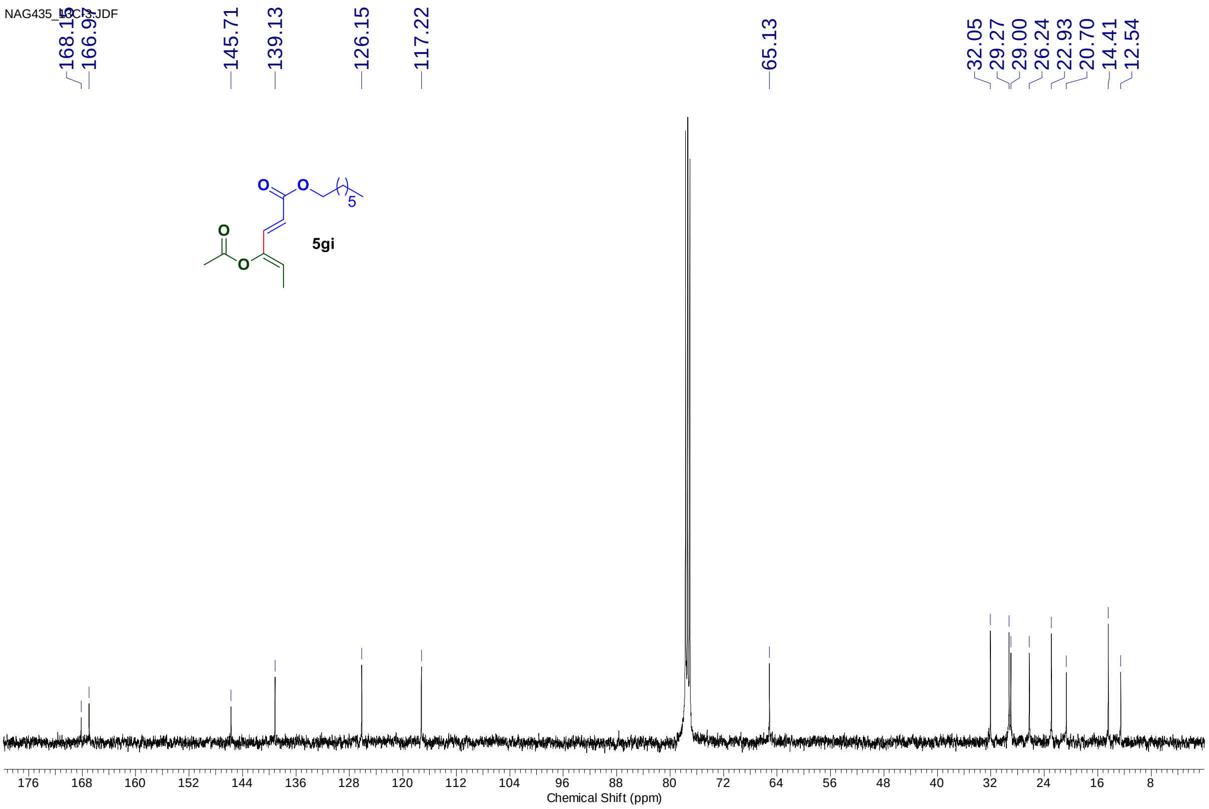
NAG435_13C13.JDF
168.13
166.97
145.71
139.13
126.15
117.22
65.13
32.05
29.27
29.00
26.24
22.93
20.70
14.41
12.54
5gi
Chemical Shift (ppm)

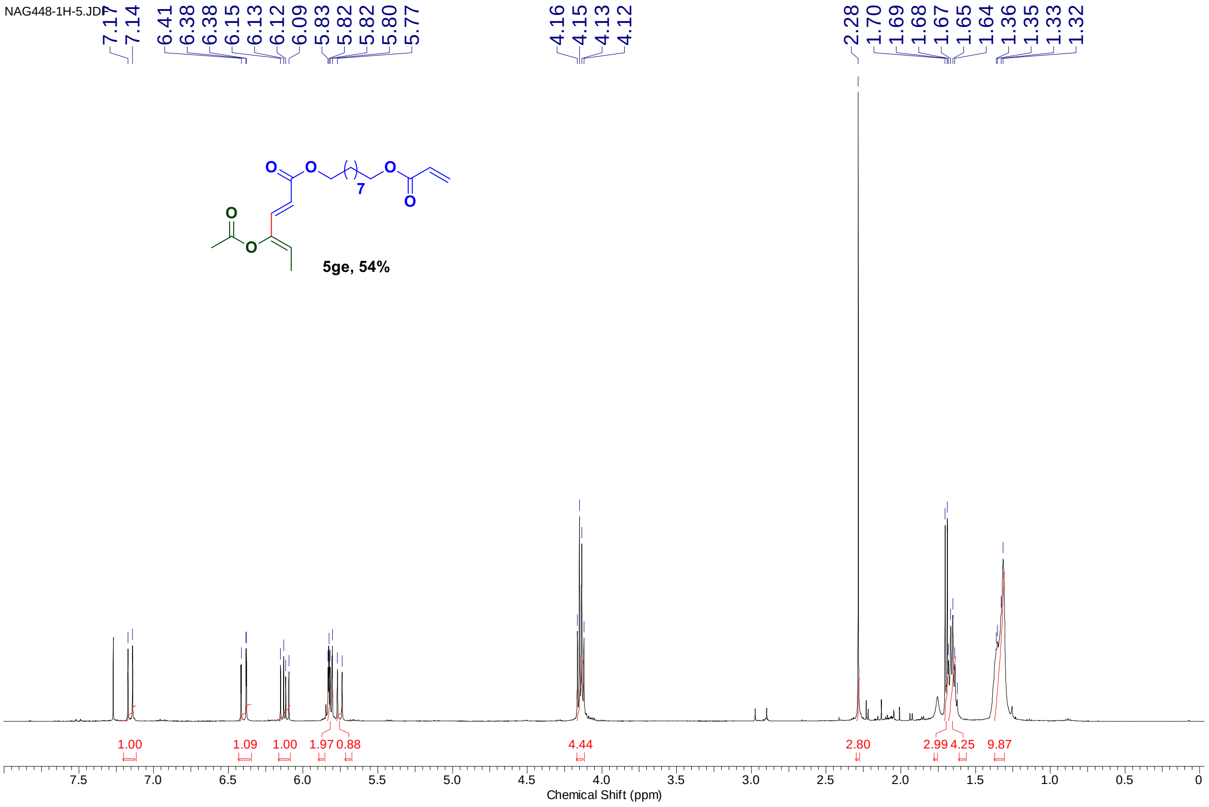
NAG448-1H-5.JDF
7.17
7.14
6.41
6.38
6.38
6.15
6.13
6.12
6.09
5.83
5.82
5.82
5.80
5.77
4.16
4.15
4.13
4.12
2.28
1.70
1.69
1.68
1.67
1.65
1.64
1.36
1.35
1.33
1.32
5ge, 54%
1.00
1.09
1.00
1.97
0.88
4.44
2.80
2.99
4.25
9.87
Chemical Shift (ppm)

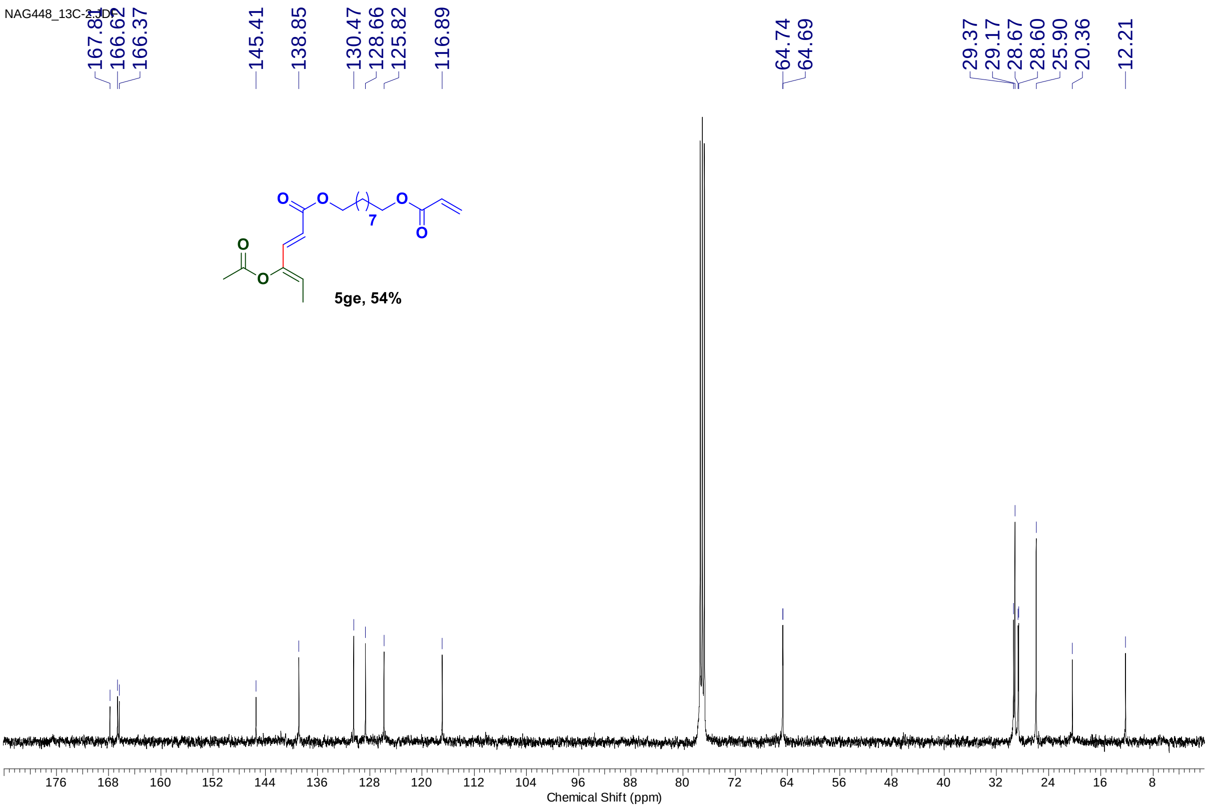
NAG448_13C-2.JDF
167.81
166.62
166.37
145.41
138.85
130.47
128.66
125.82
116.89
64.74
64.69
29.37
29.17
28.67
28.60
25.90
20.36
12.21
5ge, 54%
Chemical Shift (ppm)

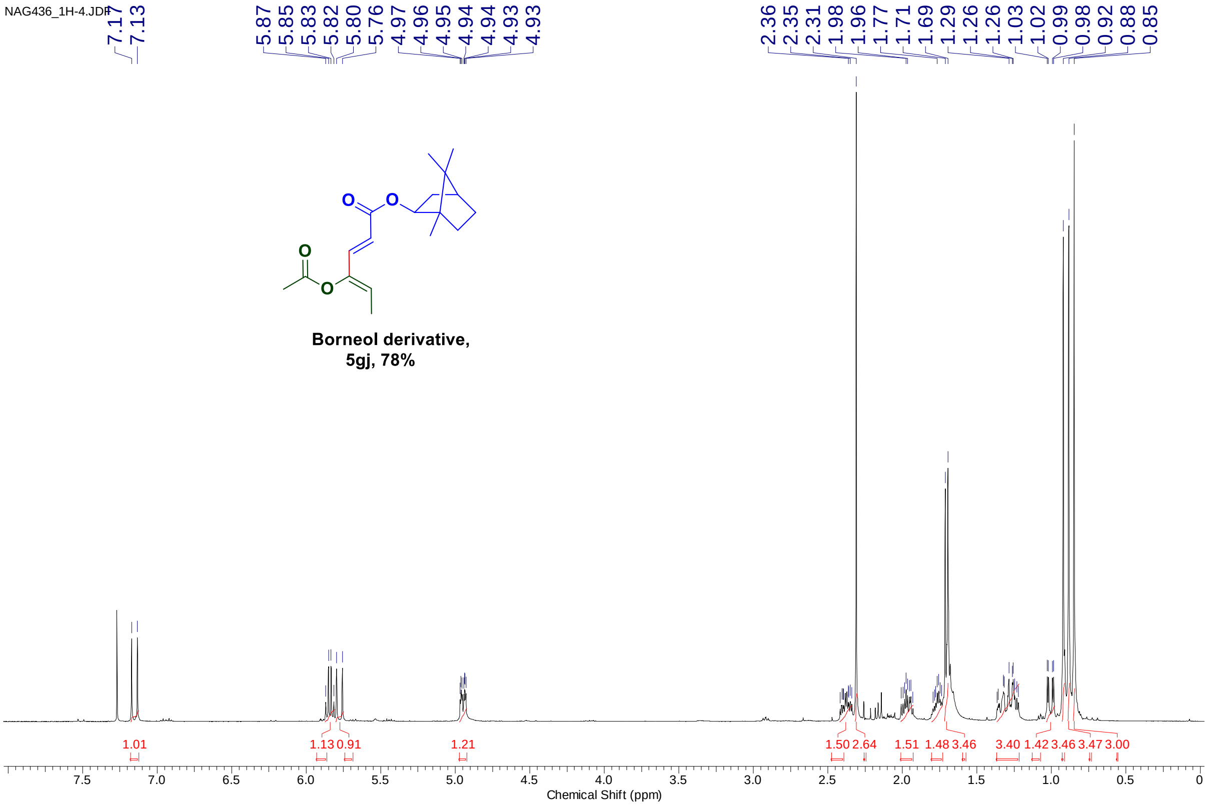
NAG436_1H-4.JDF
7.17
7.13
5.87
5.85
5.83
5.82
5.80
5.76
4.97
4.96
4.95
4.94
4.94
4.93
4.93
2.36
2.35
2.31
1.98
1.96
1.77
1.71
1.69
1.29
1.26
1.26
1.03
1.02
0.99
0.98
0.92
0.88
0.85
O
O
O
O
Borneol derivative,
5gj, 78%
1.01
1.13
0.91
1.21
1.50
2.64
1.51
1.48
3.46
3.40
1.42
3.46
3.47
3.00
7.5 7.0 6.5 6.0 5.5 5.0 4.5 4.0 3.5 3.0 2.5 2.0 1.5 1.0 0.5 0
Chemical Shift (ppm)

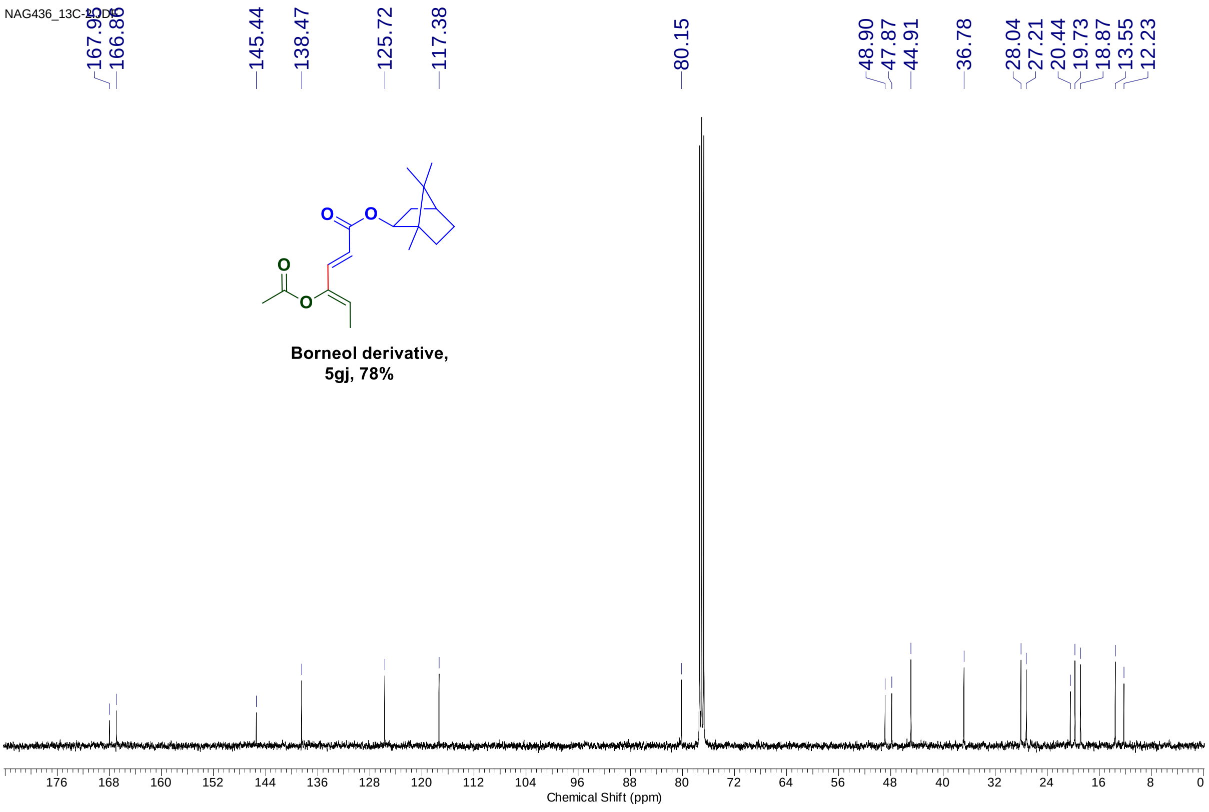
145.44
138.47
125.72
117.38
80.15
48.90
47.87
44.91
36.78
28.04
27.21
20.44
19.73
18.87
13.55
12.23
O
O
O
O
Borneol derivative,
5gj, 78%
176 168 160 152 144 136 128 120 112 104 96 88 80 72 64 56 48 40 32 24 16 8 0
Chemical Shift (ppm)

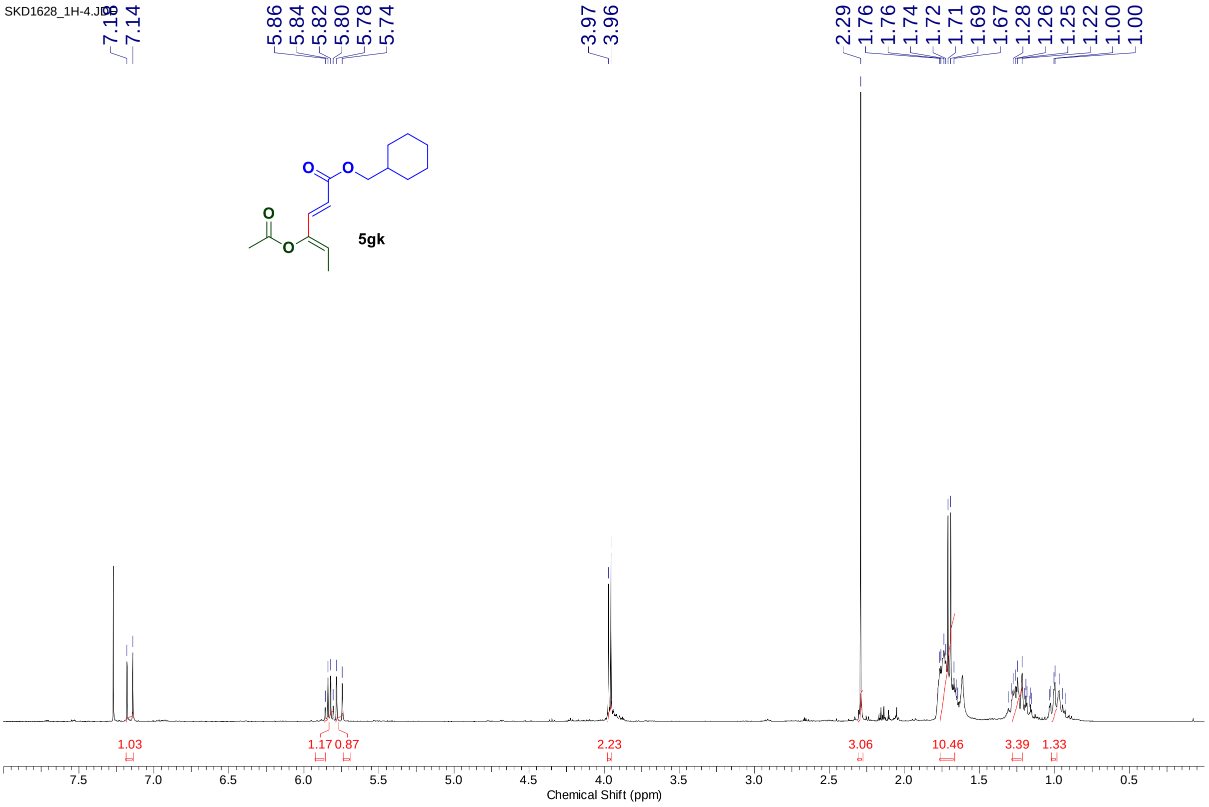
SKD1628_1H-4.JDF
7.19
7.14
5.86
5.84
5.82
5.80
5.78
5.74
3.97
3.96
2.29
1.76
1.76
1.74
1.72
1.71
1.69
1.67
1.28
1.26
1.25
1.22
1.00
1.00
5gk
1.03
1.17
0.87
2.23
3.06
10.46
3.39
1.33
Chemical Shift (ppm)

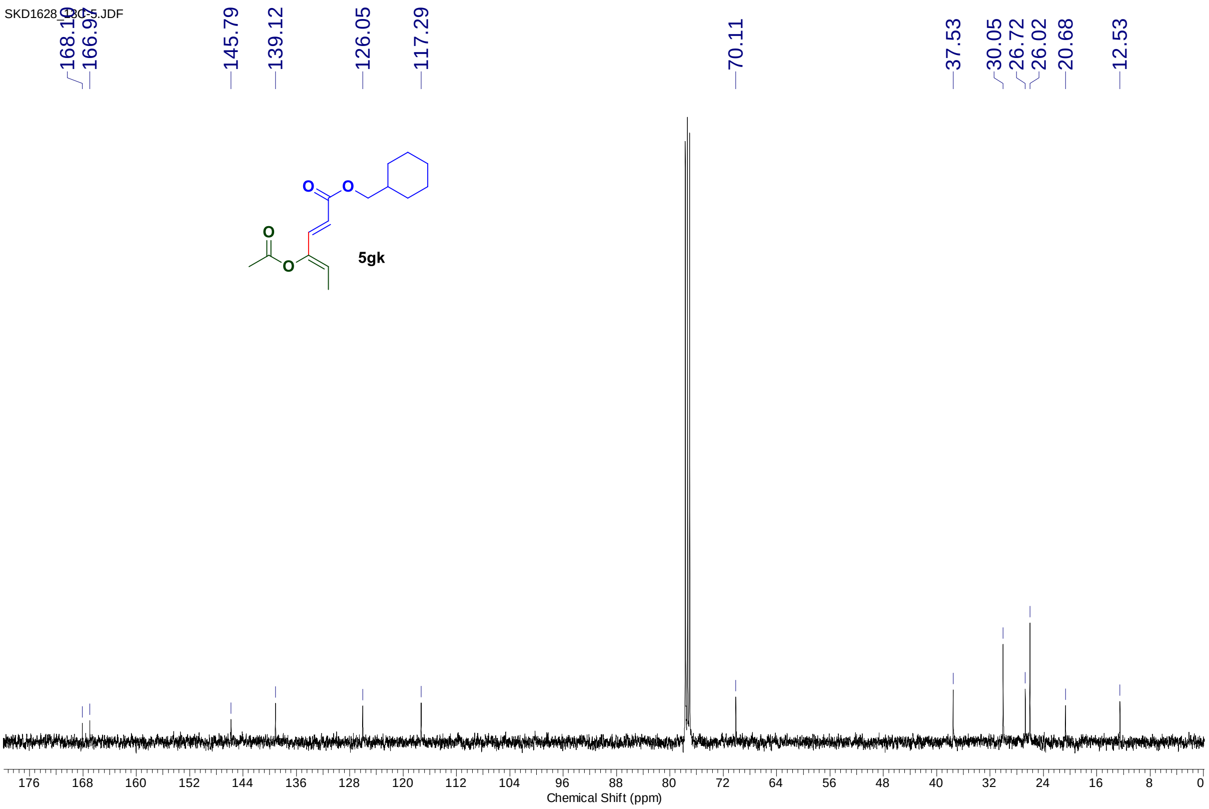
SKD1628_13C-5.JDF
168.10
166.97
145.79
139.12
126.05
117.29
70.11
37.53
30.05
26.72
26.02
20.68
12.53
5gk
Chemical Shift (ppm)

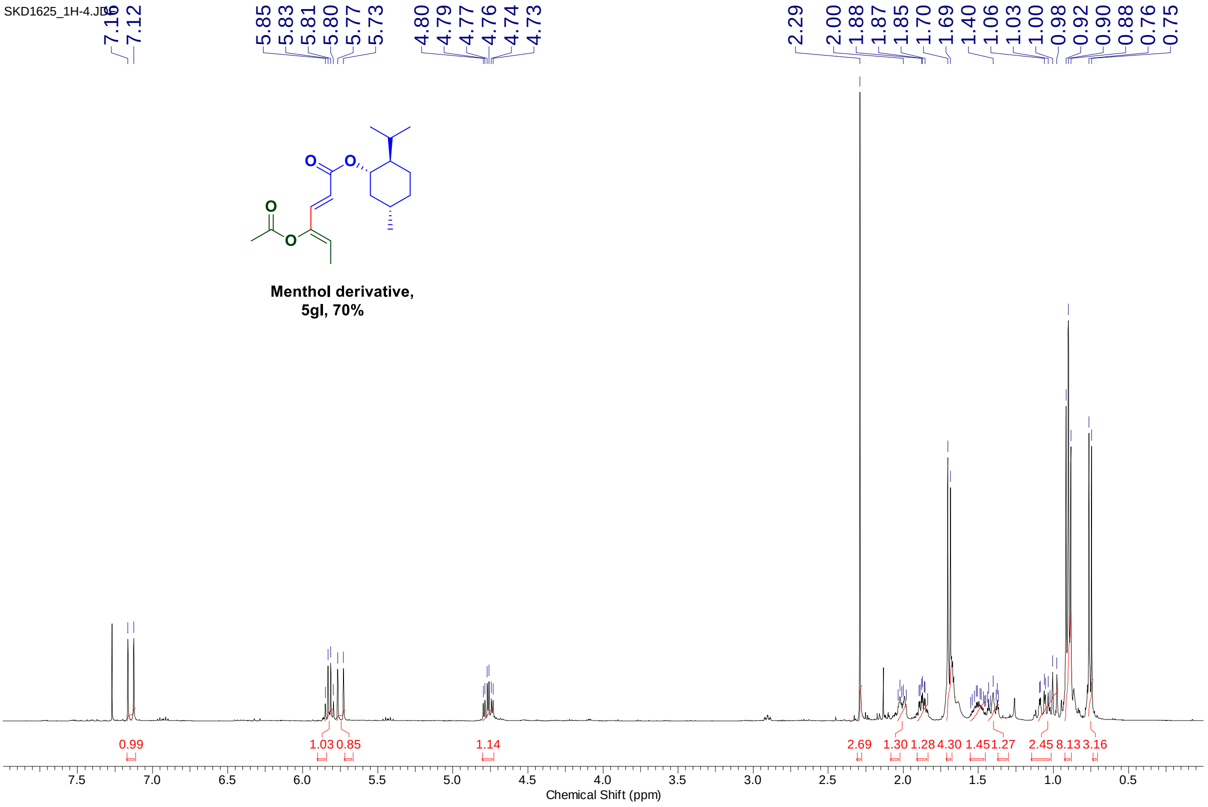
SKD1625_1H-4.JDF
Menthol derivative,
5gl, 70%
Chemical Shift (ppm)

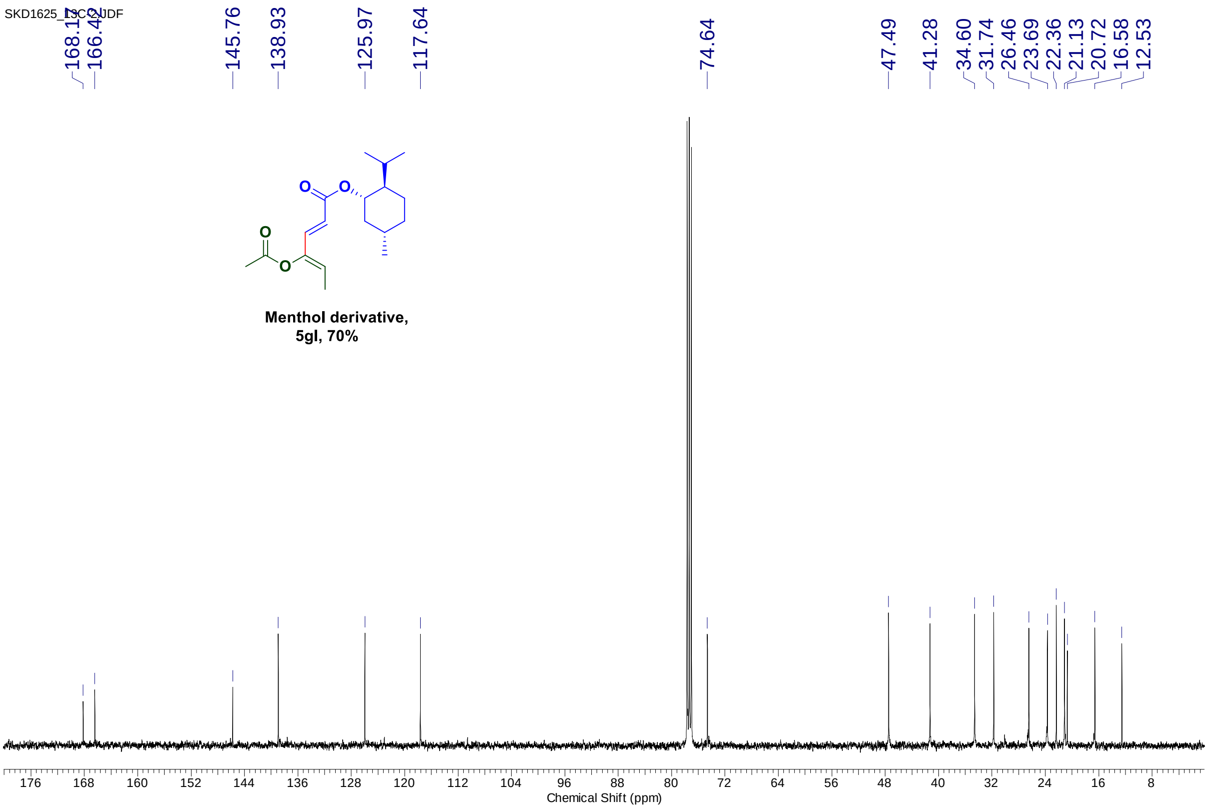
SKD1625_13C-2.JDF
Menthol derivative,
5gl, 70%
Chemical Shift (ppm)

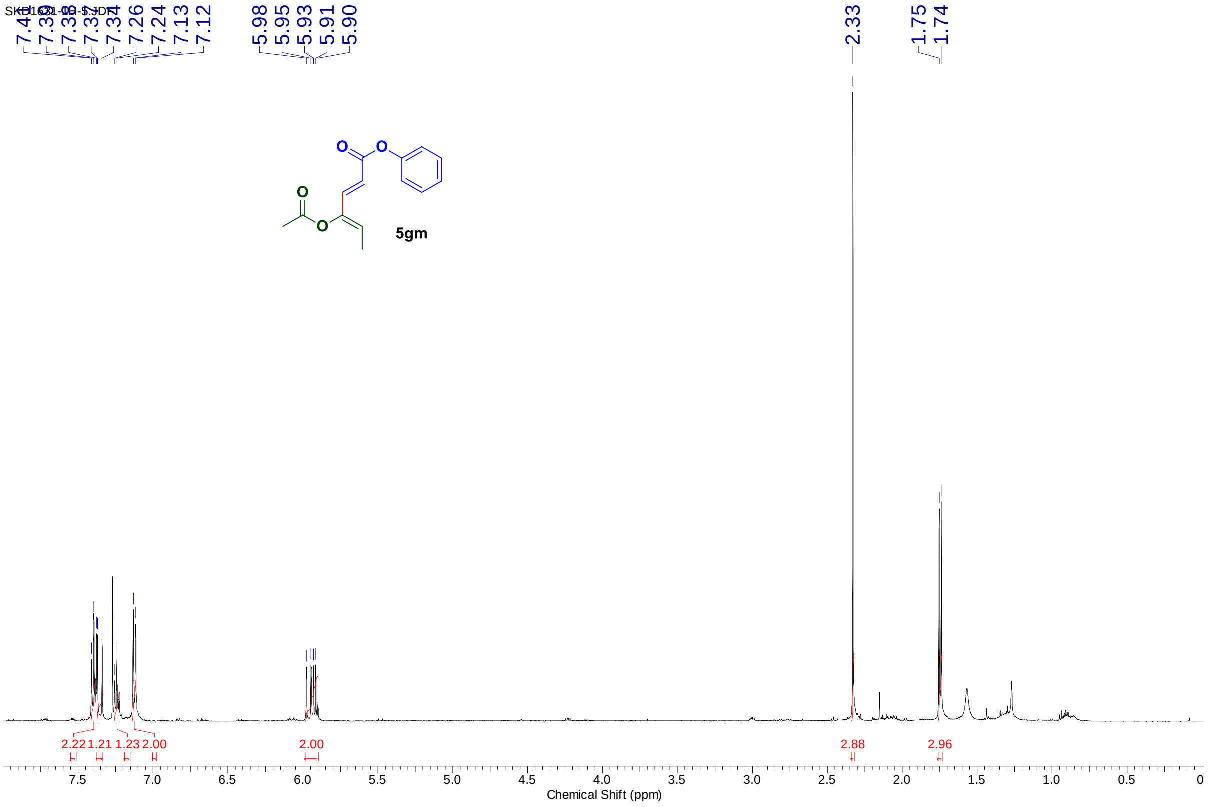
7.26
7.24
7.13
7.12
5.98
5.95
5.93
5.91
5.90
2.33
1.75
1.74
5gm
2.22
1.21
1.23
2.00
2.00
2.88
2.96
Chemical Shift (ppm)

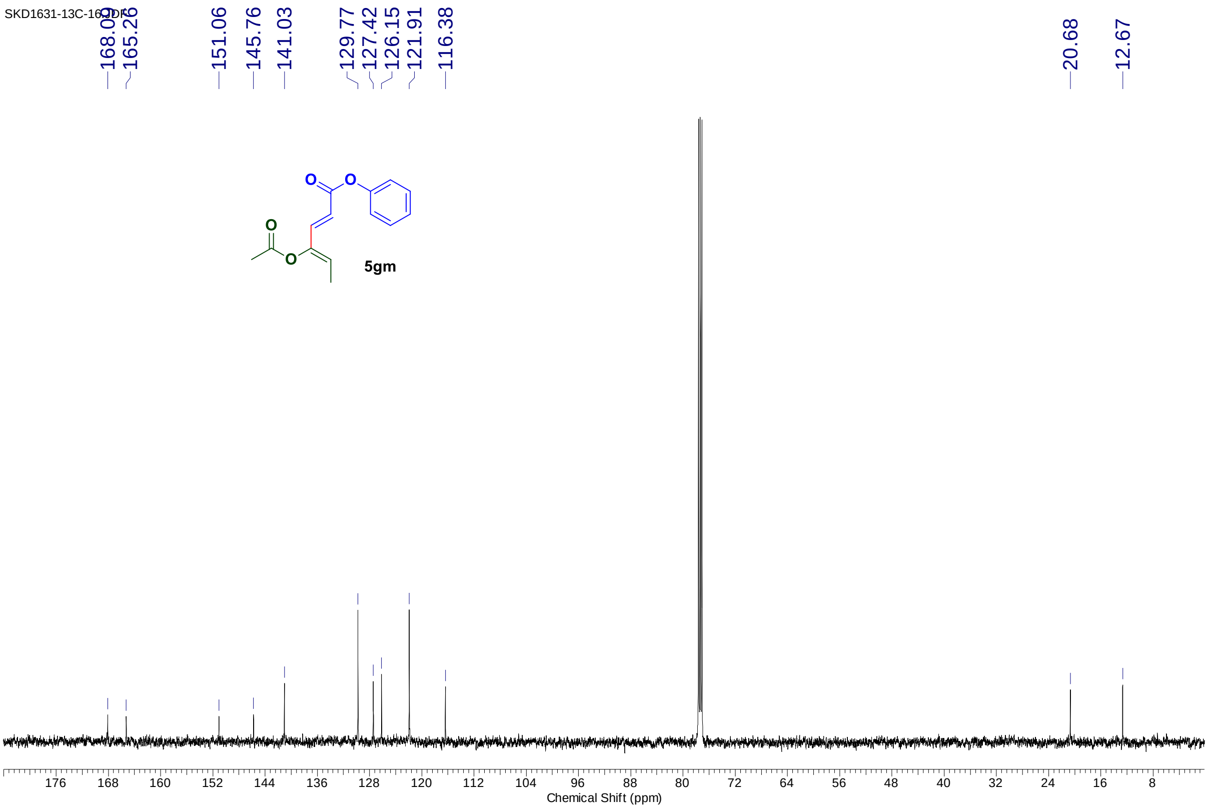
165.26
151.06
145.76
141.03
129.77
127.42
126.15
121.91
116.38
20.68
12.67
5gm
Chemical Shift (ppm)

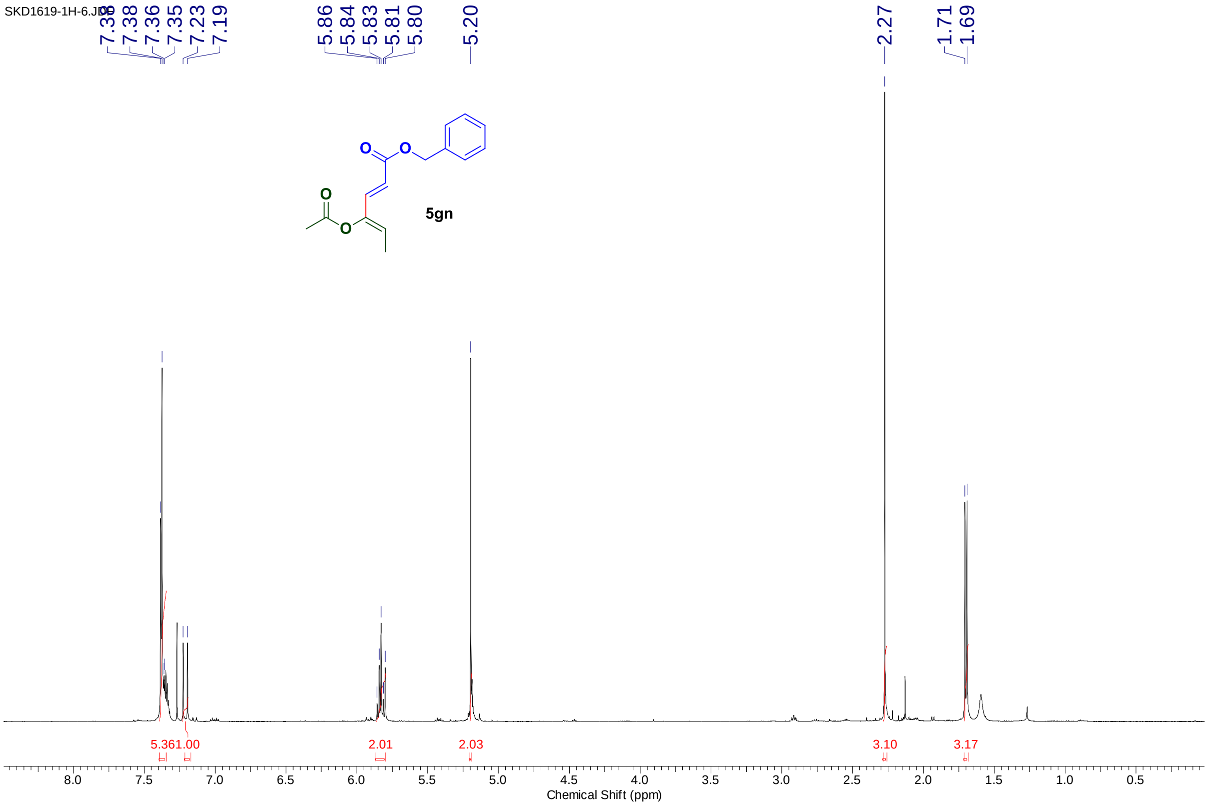
SKD1619-1H-6.JDF
7.38
7.36
7.35
7.23
7.19
5.86
5.84
5.83
5.81
5.80
5.20
2.27
1.71
1.69
O
O
O
O
5gn
5.36
1.00
2.01
2.03
3.10
3.17
8.0 7.5 7.0 6.5 6.0 5.5 5.0 4.5 4.0 3.5 3.0 2.5 2.0 1.5 1.0 0.5
Chemical Shift (ppm)

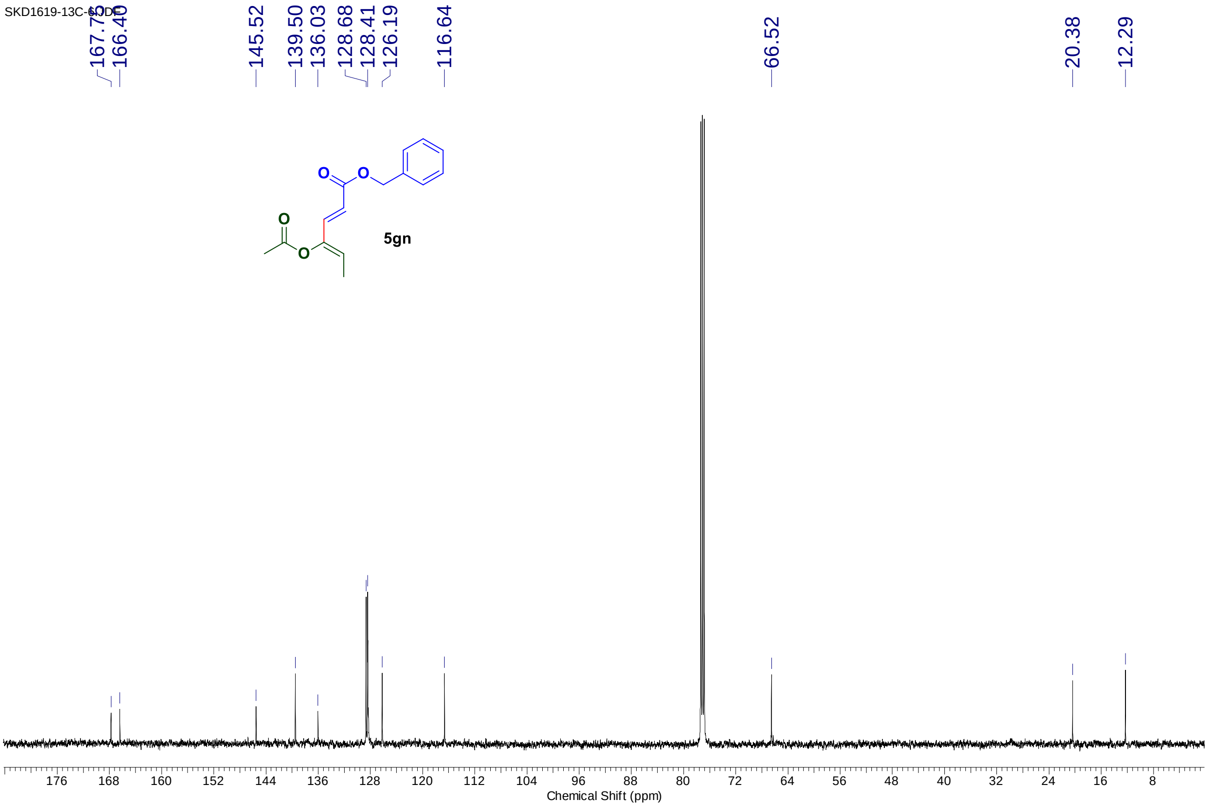
SKD1619-13C-6.JDF
166.40
145.52
139.50
136.03
128.68
128.41
126.19
116.64
66.52
20.38
12.29
O
O
O
O
5gn
176 168 160 152 144 136 128 120 112 104 96 88 80 72 64 56 48 40 32 24 16 8
Chemical Shift (ppm)

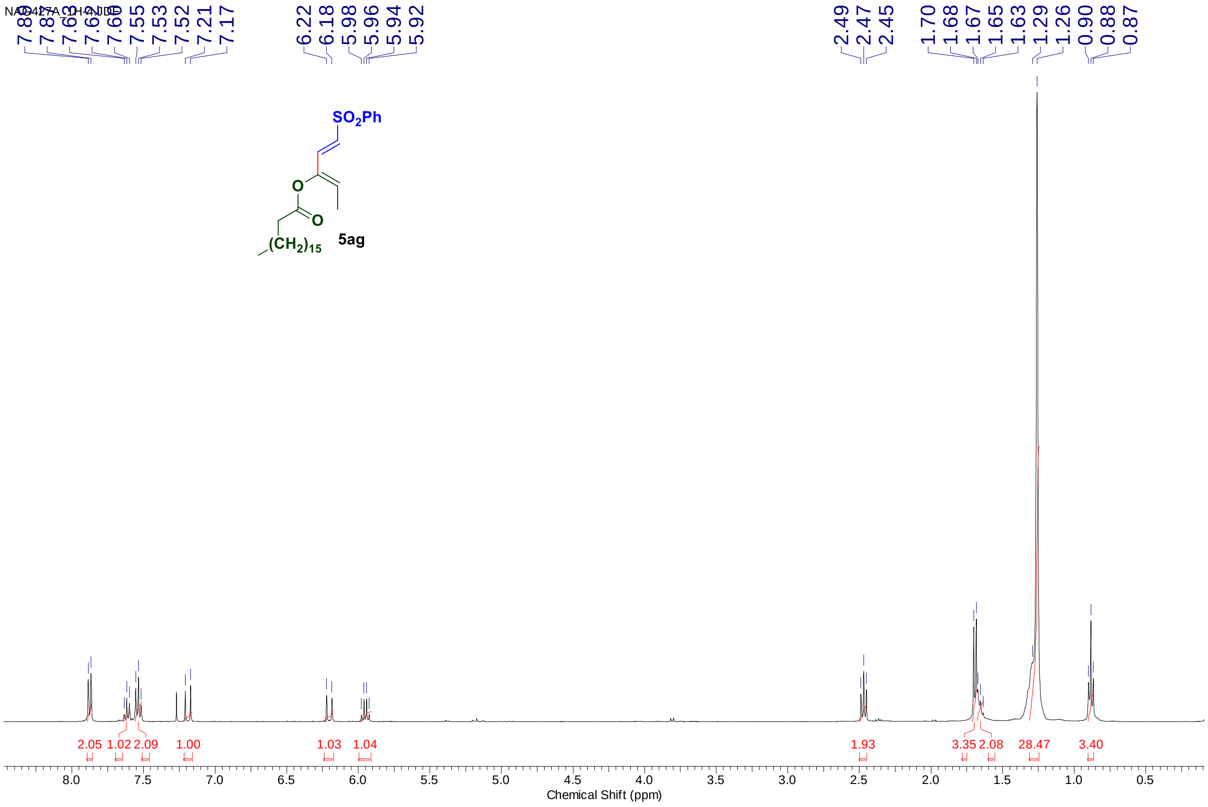
7.87
7.63
7.62
7.60
7.55
7.53
7.52
7.21
7.17
6.22
6.18
5.98
5.96
5.94
5.92
2.49
2.47
2.45
1.70
1.68
1.67
1.65
1.63
1.29
1.26
0.90
0.88
0.87
SO2Ph
O
O
(CH2)15
5ag
2.05 1.02 2.09 1.00 1.03 1.04 1.93 3.35 2.08 28.47 3.40
Chemical Shift (ppm)

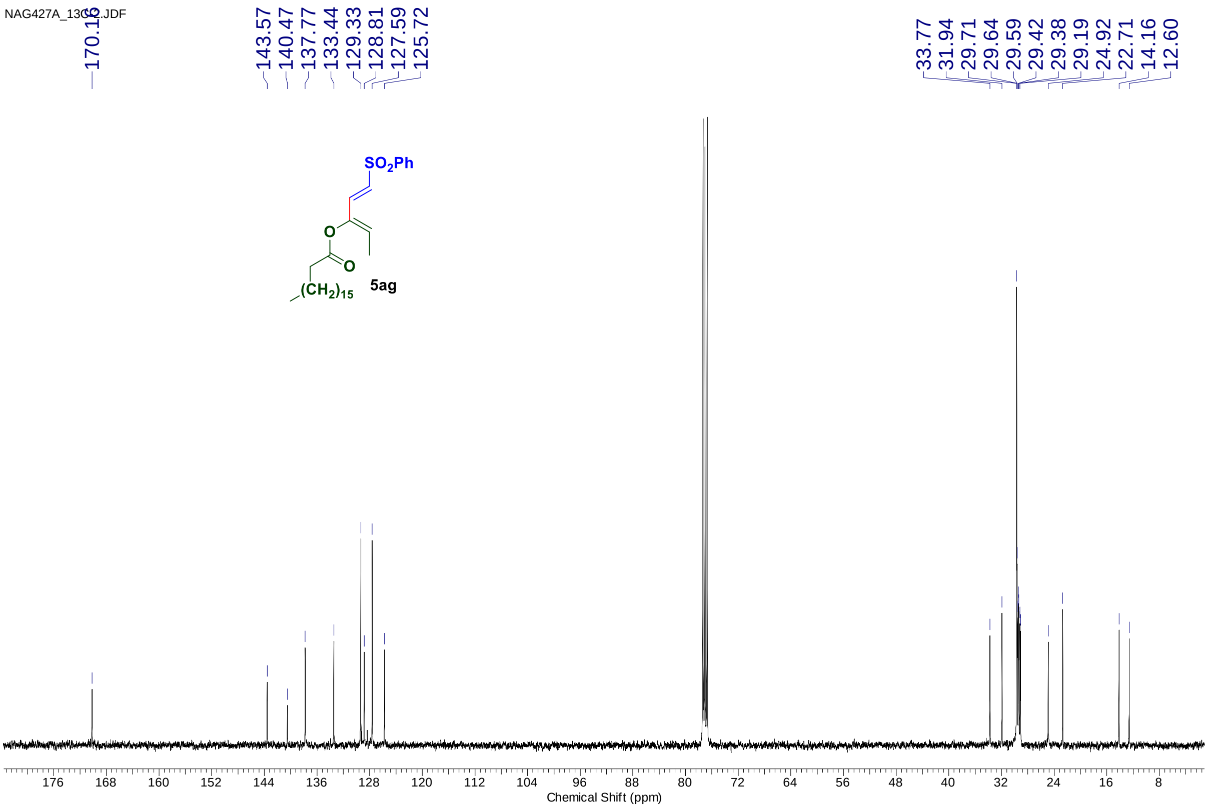
NAG427A_13C
170.10
143.57
140.47
137.77
133.44
129.33
128.81
127.59
125.72
33.77
31.94
29.71
29.64
29.59
29.42
29.38
29.19
24.92
22.71
14.16
12.60
SO2Ph
O
O
(CH2)15
5ag
Chemical Shift (ppm)

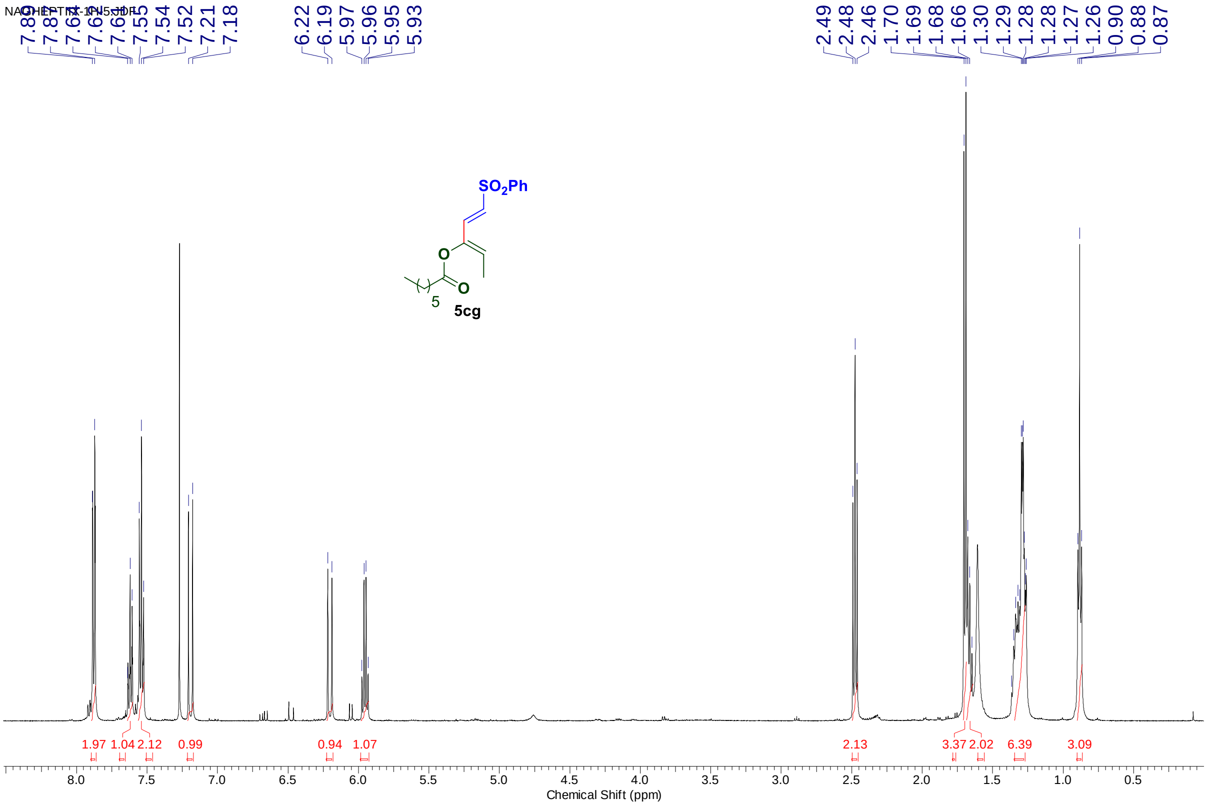
7.89 7.87 7.64 7.62 7.60 7.55 7.54 7.52 7.21 7.18
6.22 6.19 5.97 5.96 5.95 5.93
2.49 2.48 2.46 1.70 1.69 1.68 1.66 1.30 1.29 1.28 1.28 1.27 1.26 0.90 0.88 0.87
SO2Ph
O
O
5
5cg
1.97 1.04 2.12 0.99 0.94 1.07 2.13 3.37 2.02 6.39 3.09
Chemical Shift (ppm)

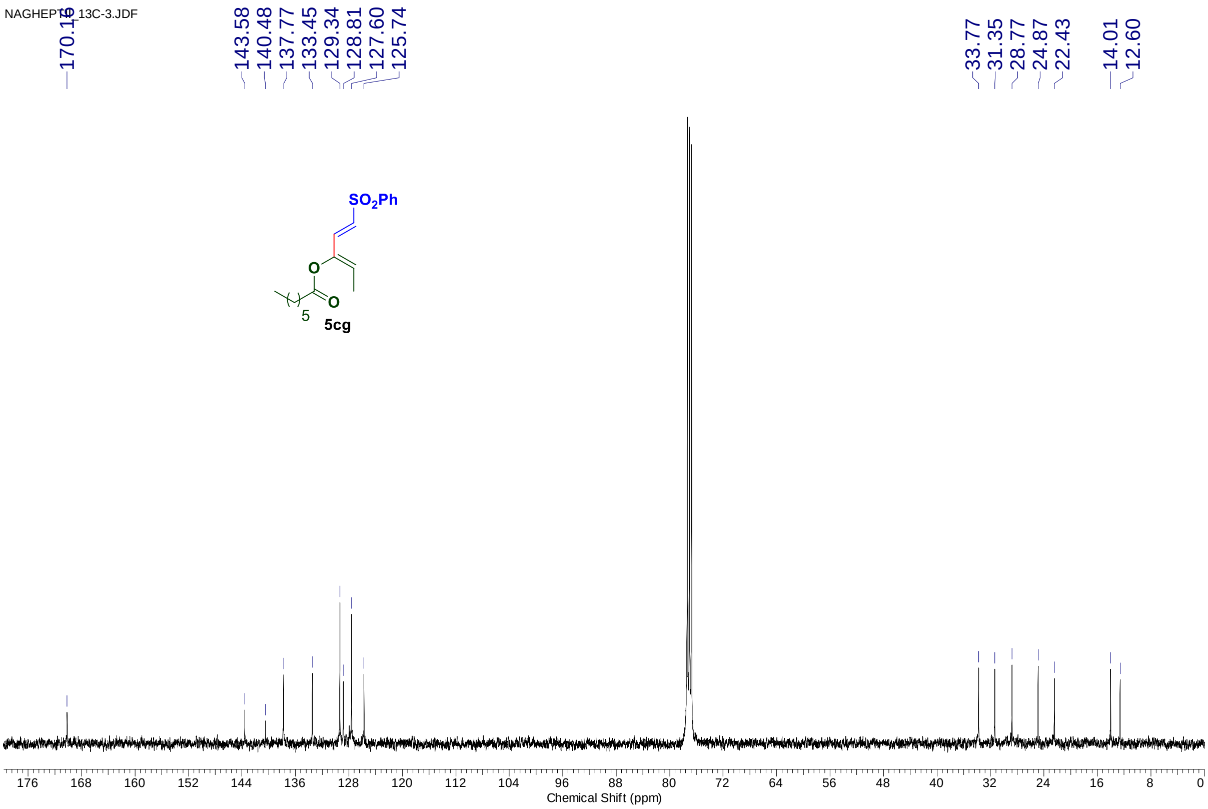
143.58 140.48 137.77 133.45 129.34 128.81 127.60 125.74
33.77 31.35 28.77 24.87 22.43 14.01 12.60
SO2Ph
O
O
5
5cg
Chemical Shift (ppm)

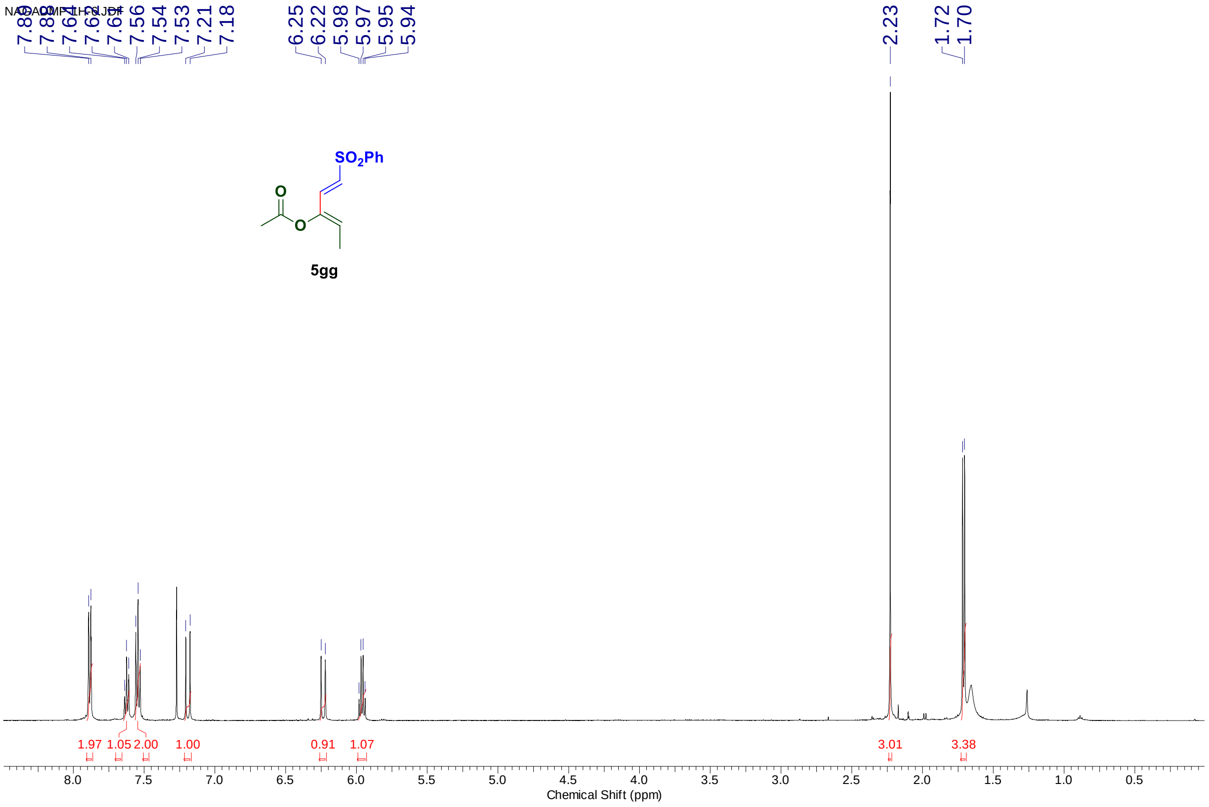
7.61
7.56
7.54
7.53
7.21
7.18
6.25
6.22
5.98
5.97
5.95
5.94
2.23
1.72
1.70
SO2Ph
O
O
5gg
1.97 1.05 2.00 1.00 0.91 1.07 3.01 3.38
8.0 7.5 7.0 6.5 6.0 5.5 5.0 4.5 4.0 3.5 3.0 2.5 2.0 1.5 1.0 0.5
Chemical Shift (ppm)

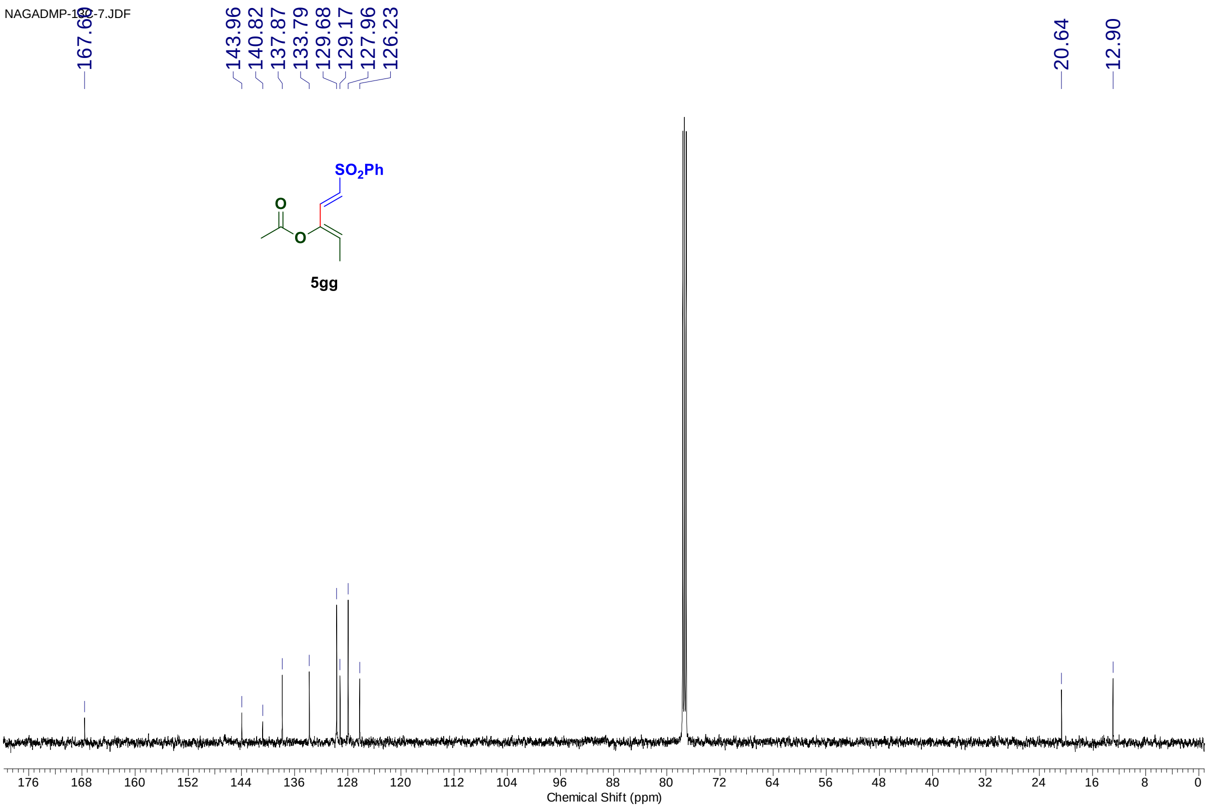
NAGADMP-13C-7.JDF
167.60
143.96
140.82
137.87
133.79
129.68
129.17
127.96
126.23
20.64
12.90
SO2Ph
O
O
5gg
176 168 160 152 144 136 128 120 112 104 96 88 80 72 64 56 48 40 32 24 16 8 0
Chemical Shift (ppm)

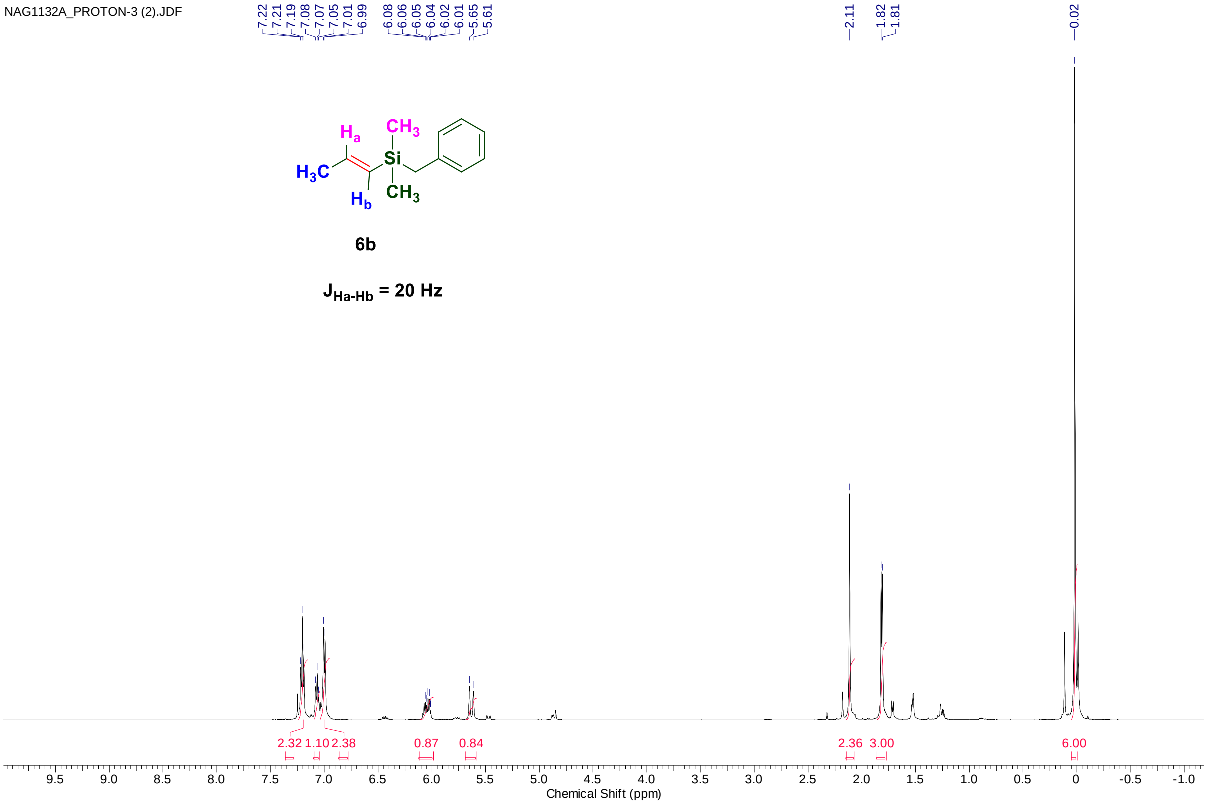
NAG1132A_PROTON-3 (2).JDF
7.22 7.21 7.19 7.08 7.07 7.05 7.01 6.99 6.08 6.06 6.05 6.04 6.02 6.01 5.65 5.61
2.11 1.82 1.81
0.02
H$_a$
CH$_3$
H$_3$C
Si
H$_b$
CH$_3$
6b
J$_{Ha-Hb}$ = 20 Hz
2.32 1.10 2.38 0.87 0.84 2.36 3.00 6.00
9.5 9.0 8.5 8.0 7.5 7.0 6.5 6.0 5.5 5.0 4.5 4.0 3.5 3.0 2.5 2.0 1.5 1.0 0.5 0 -0.5 -1.0
Chemical Shift (ppm)

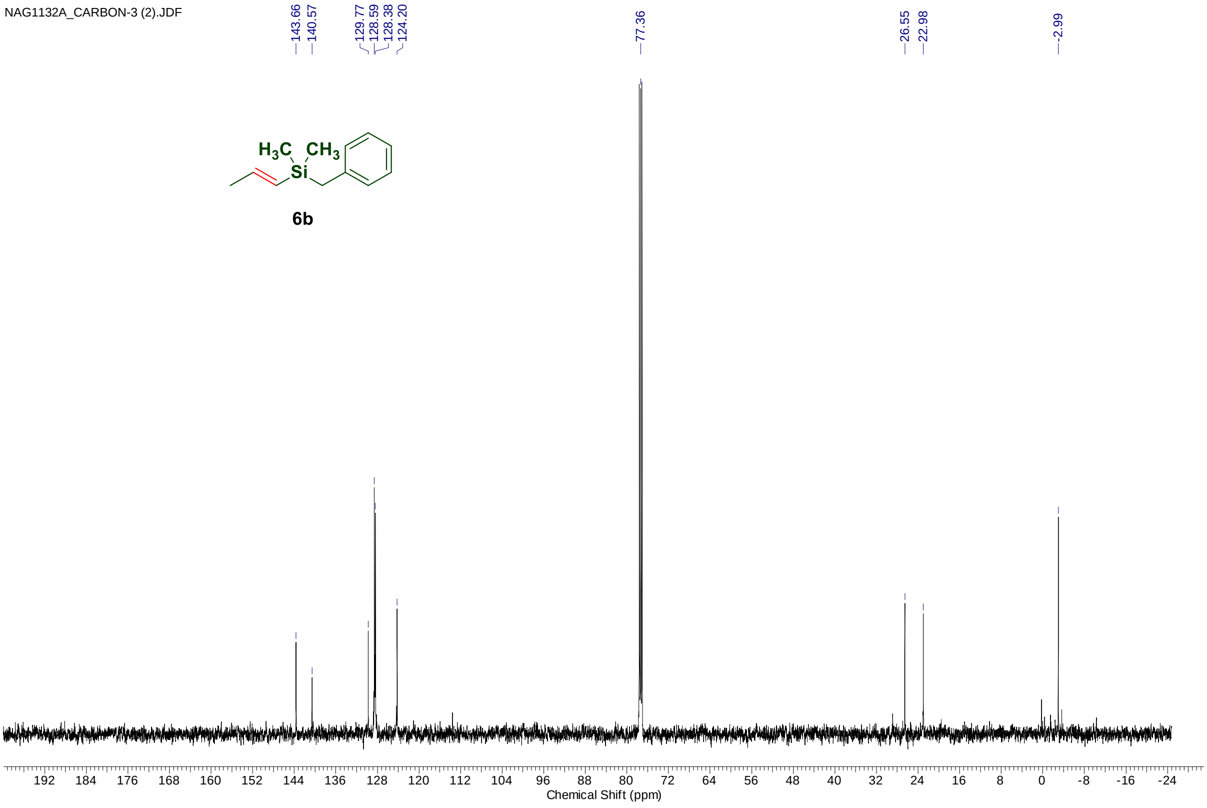
NAG1132A_CARBON-3 (2).JDF
143.66 140.57 129.77 128.59 128.38 124.20
77.36
26.55 22.98
-2.99
H$_3$C CH$_3$
Si
6b
192 184 176 168 160 152 144 136 128 120 112 104 96 88 80 72 64 56 48 40 32 24 16 8 0 -8 -16 -24
Chemical Shift (ppm)

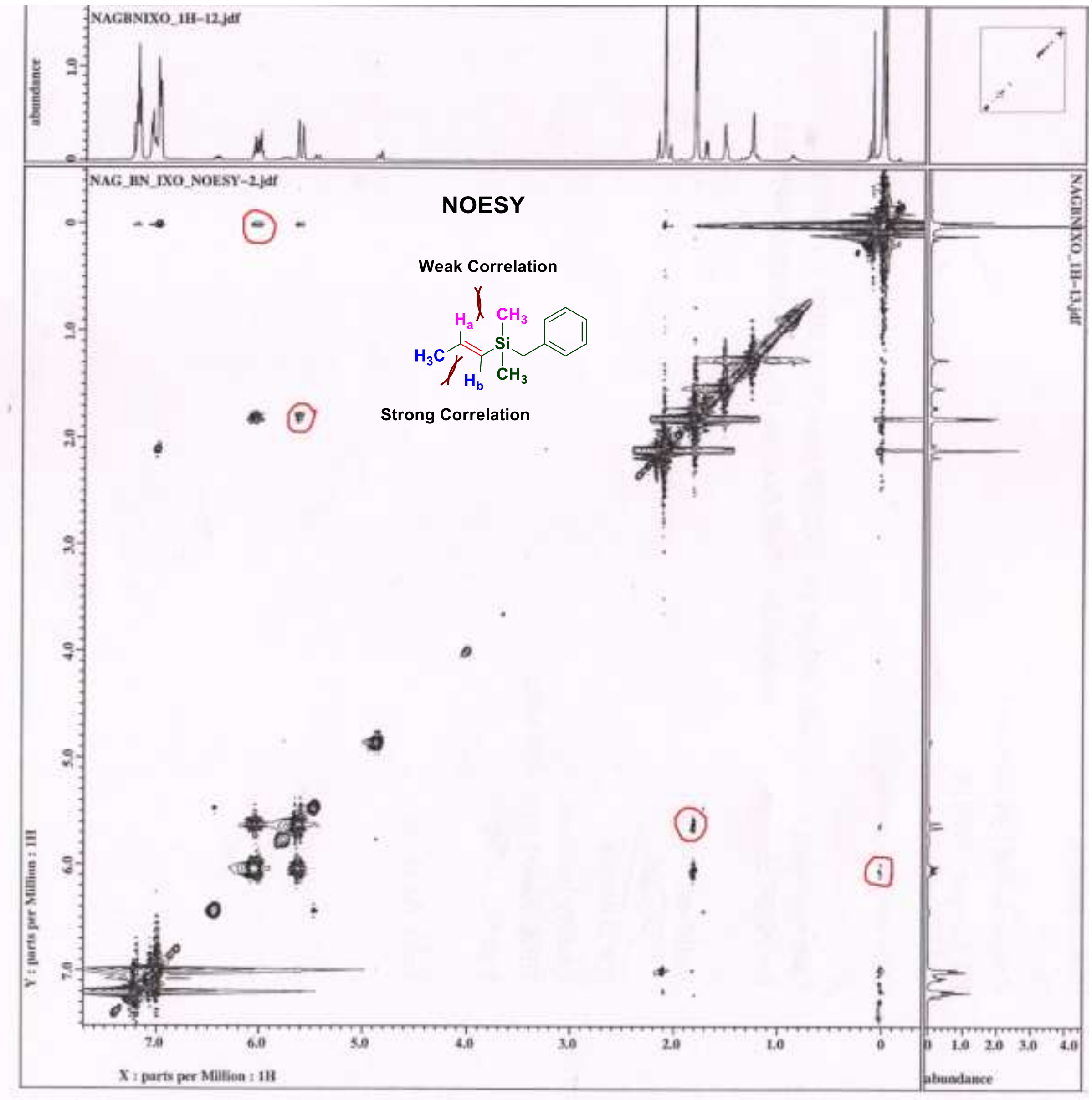
NAGBNIXO_1H-12.jdf
abundance
NAG_BN_IXO_NOESY-2.jdf
NOESY
Weak Correlation
Strong Correlation
X : parts per Million : 1H
Y : parts per Million : 1H
NAGBNIXO_1H-13.jdf
abundance

NAG1140DP_PROTON-4 (3).JDF

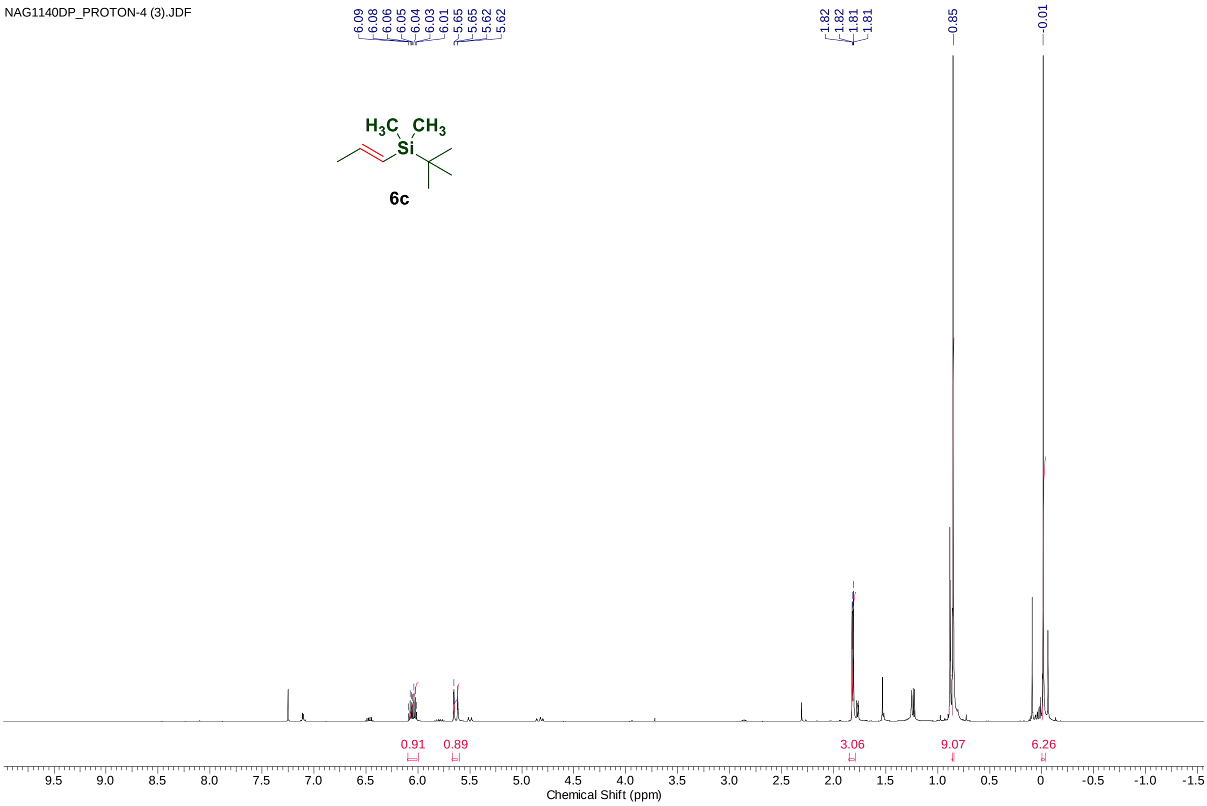

NAG1140DP_CARBON-3 (1).JDF

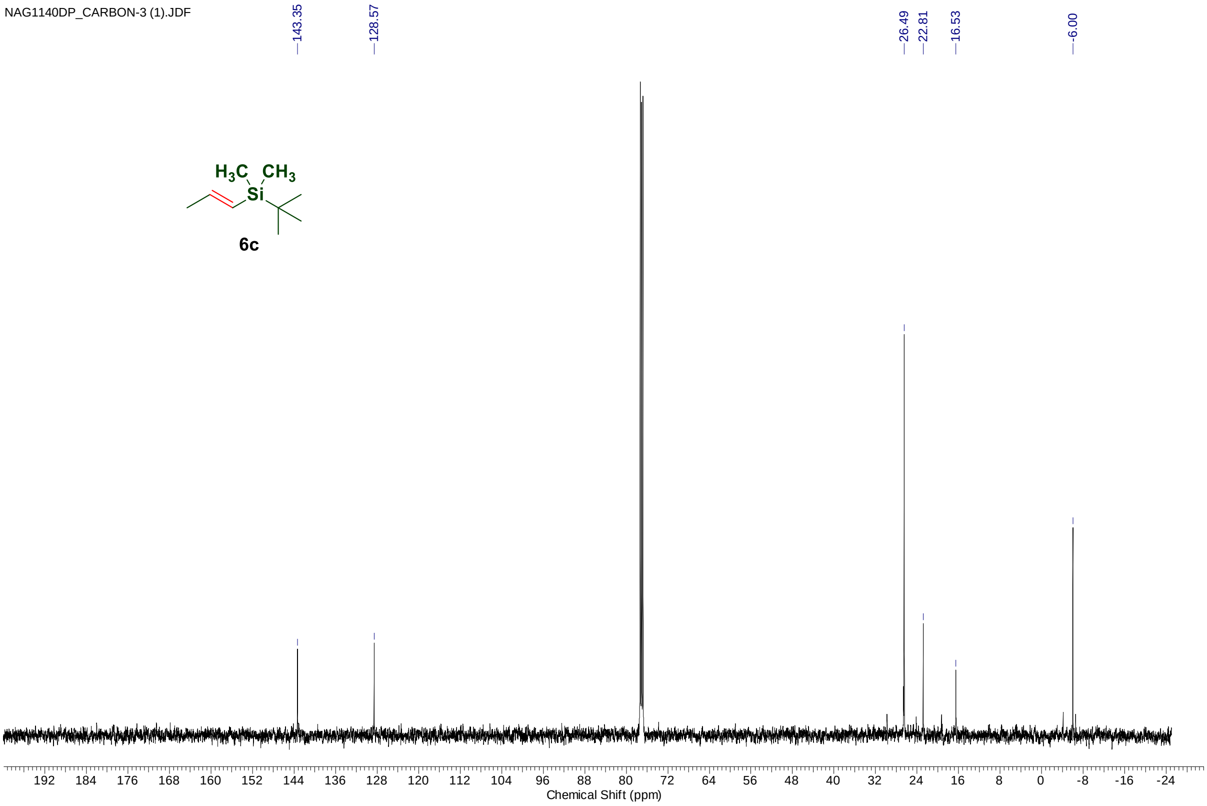

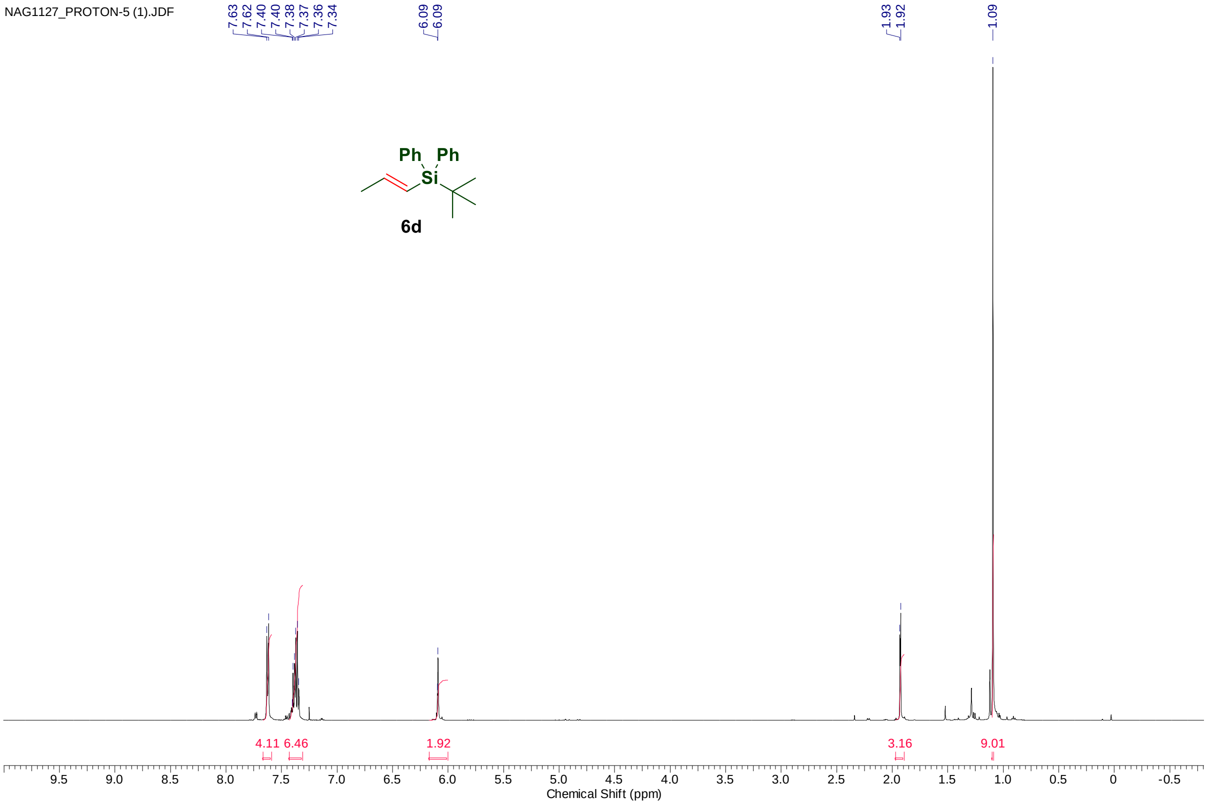
NAG1127_PROTON-5 (1).JDF
7.63
7.62
7.40
7.40
7.38
7.37
7.36
7.34
6.09
6.09
1.93
1.92
1.09
Ph Ph
Si
6d
4.11 6.46
1.92
3.16
9.01
9.5 9.0 8.5 8.0 7.5 7.0 6.5 6.0 5.5 5.0 4.5 4.0 3.5 3.0 2.5 2.0 1.5 1.0 0.5 0 -0.5
Chemical Shift (ppm)

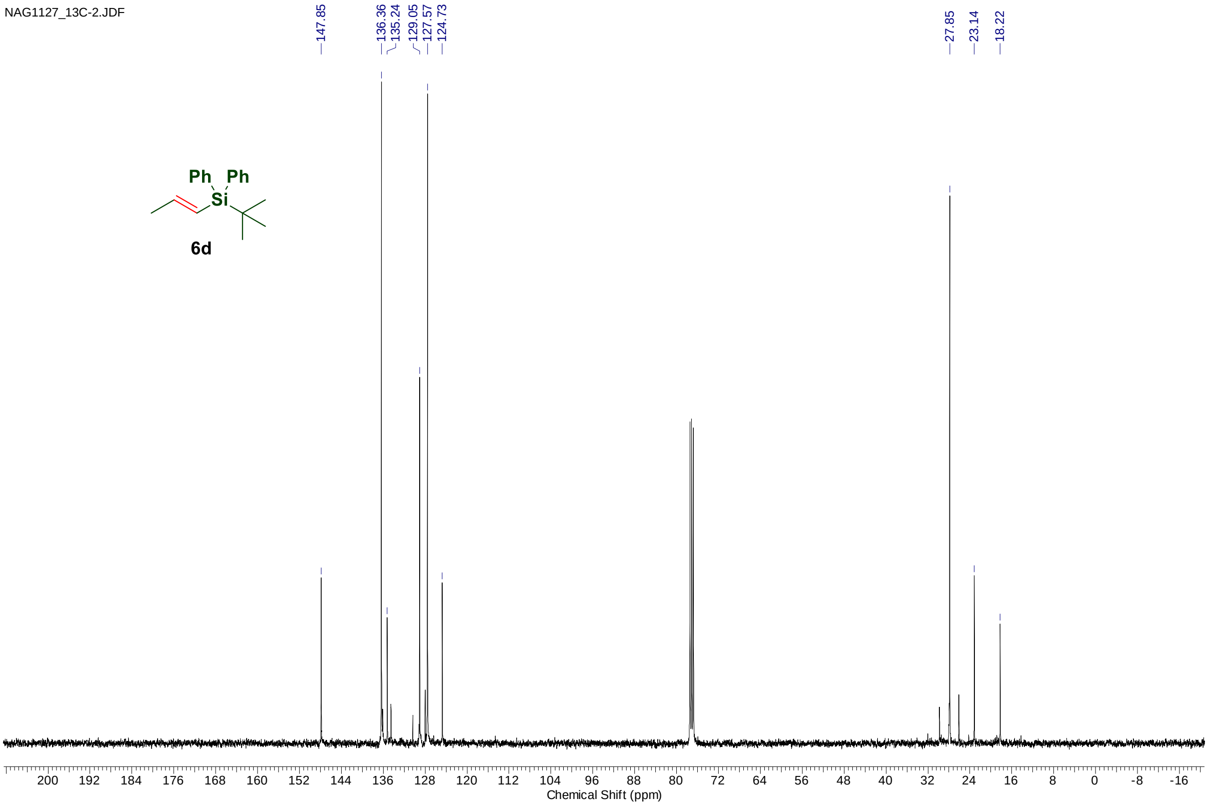
NAG1127_13C-2.JDF
147.85
136.36
135.24
129.05
127.57
124.73
27.85
23.14
18.22
Ph Ph
Si
6d
200 192 184 176 168 160 152 144 136 128 120 112 104 96 88 80 72 64 56 48 40 32 24 16 8 0 -8 -16
Chemical Shift (ppm)

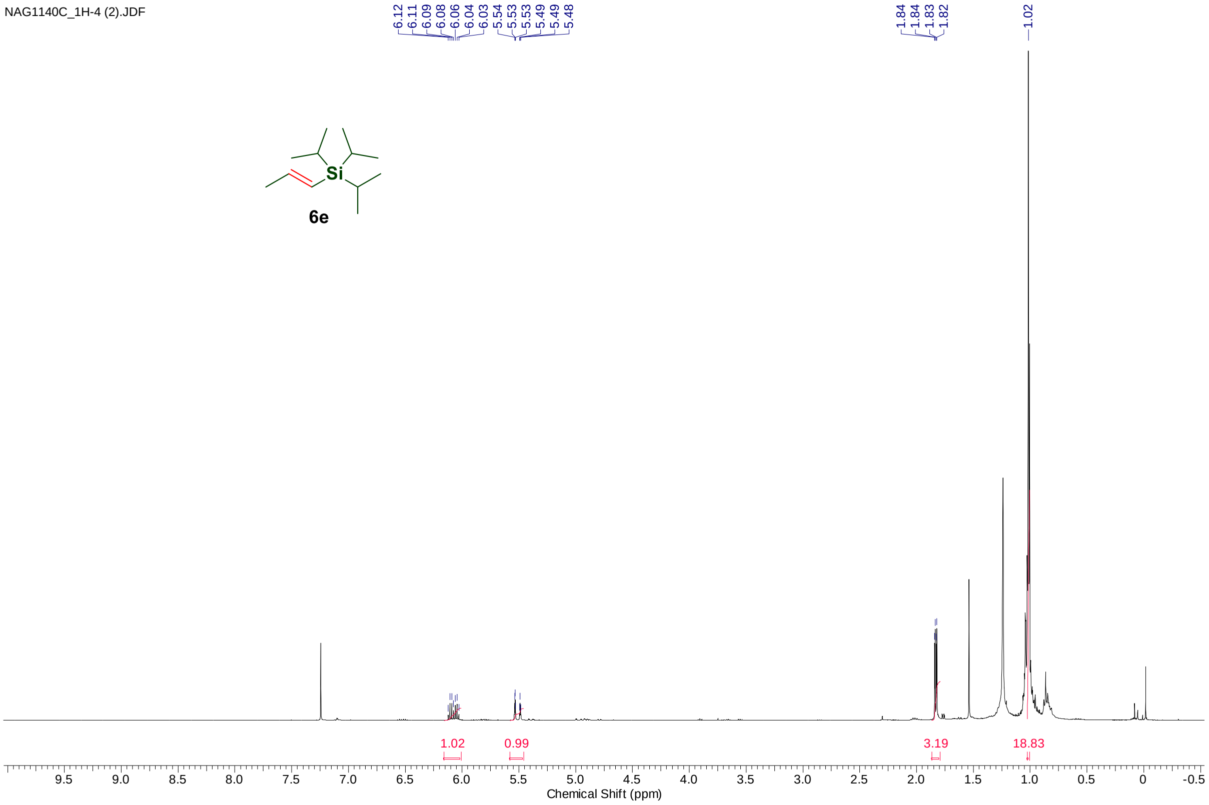
NAG1140C_1H-4 (2).JDF
6.12 6.11 6.09 6.08 6.06 6.04 6.03 5.54 5.53 5.53 5.49 5.49 5.48
1.84 1.84 1.83 1.82
1.02
Si
6e
1.02 0.99 3.19 18.83
9.5 9.0 8.5 8.0 7.5 7.0 6.5 6.0 5.5 5.0 4.5 4.0 3.5 3.0 2.5 2.0 1.5 1.0 0.5 0 -0.5
Chemical Shift (ppm)

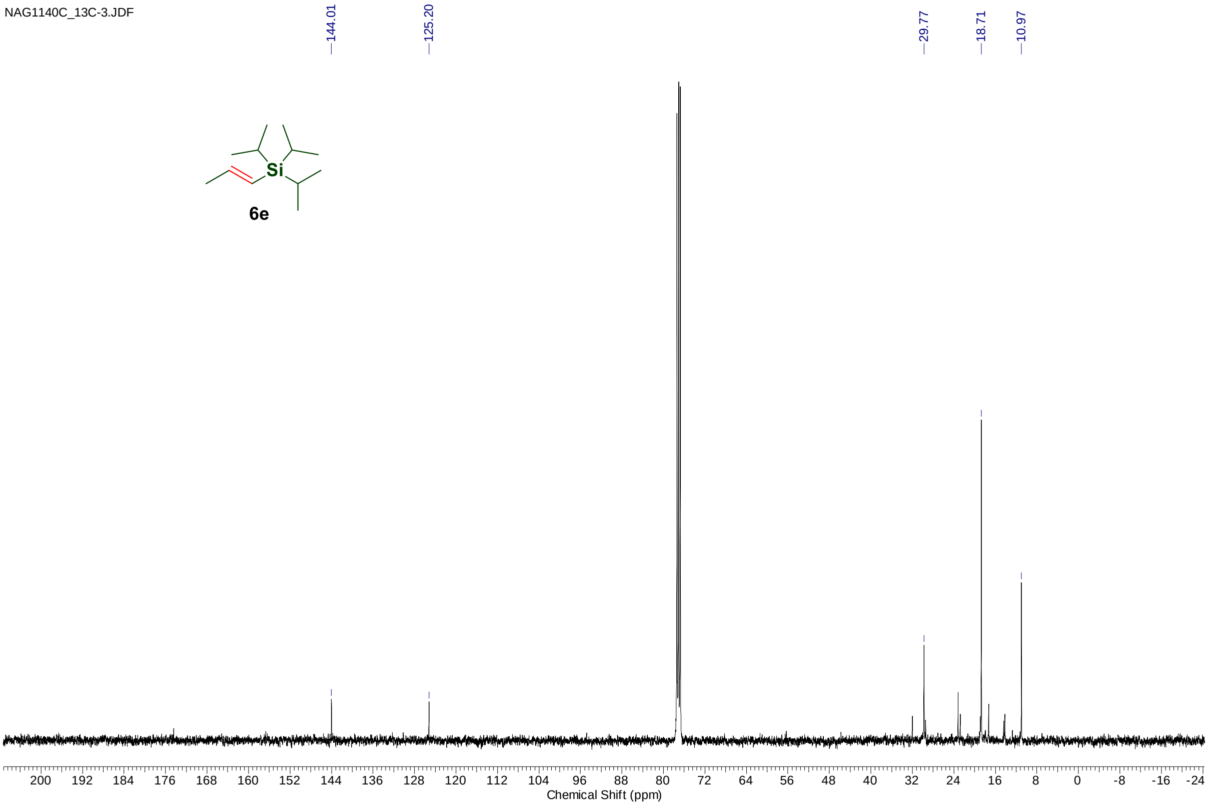
NAG1140C_13C-3.JDF
144.01
125.20
29.77
18.71
10.97
Si
6e
200 192 184 176 168 160 152 144 136 128 120 112 104 96 88 80 72 64 56 48 40 32 24 16 8 0 -8 -16 -24
Chemical Shift (ppm)

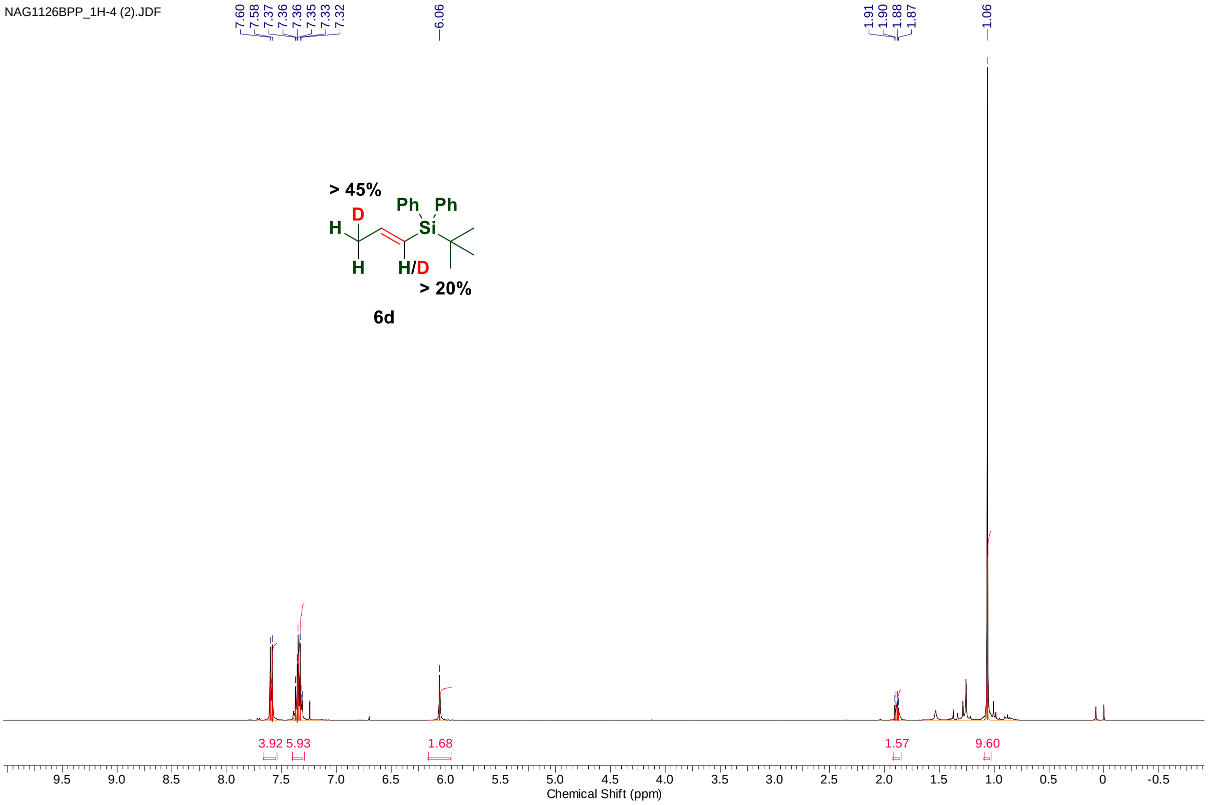
NAG1126BPP_1H-4 (2).JDF
7.60
7.58
7.37
7.36
7.36
7.35
7.33
7.32
6.06
1.91
1.90
1.88
1.87
1.06
> 45%
D
H
H
Ph
Ph
Si
H/D
> 20%
6d
3.92
5.93
1.68
1.57
9.60
Chemical Shift (ppm)

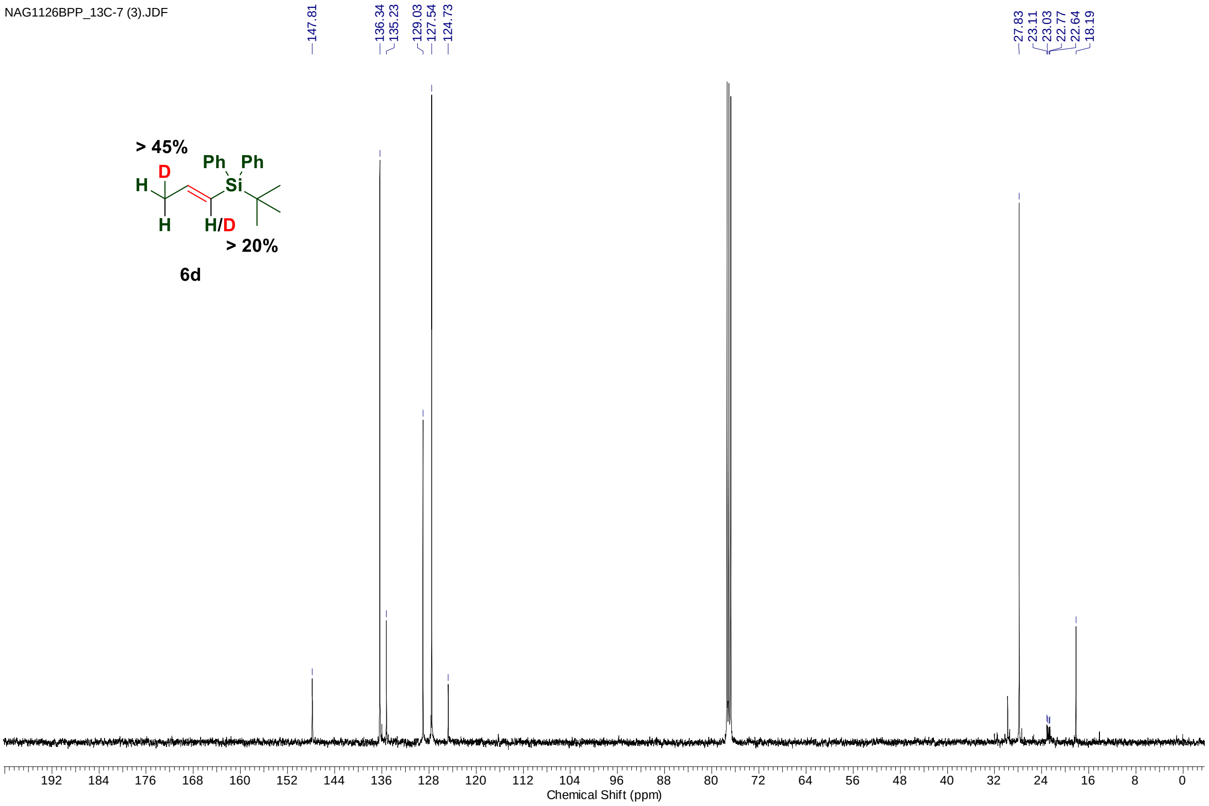
NAG1126BPP_13C-7 (3).JDF
147.81
136.34
135.23
129.03
127.54
124.73
27.83
23.11
23.03
22.77
22.64
18.19
> 45%
D
H
H
Ph
Ph
Si
H/D
> 20%
6d
Chemical Shift (ppm)

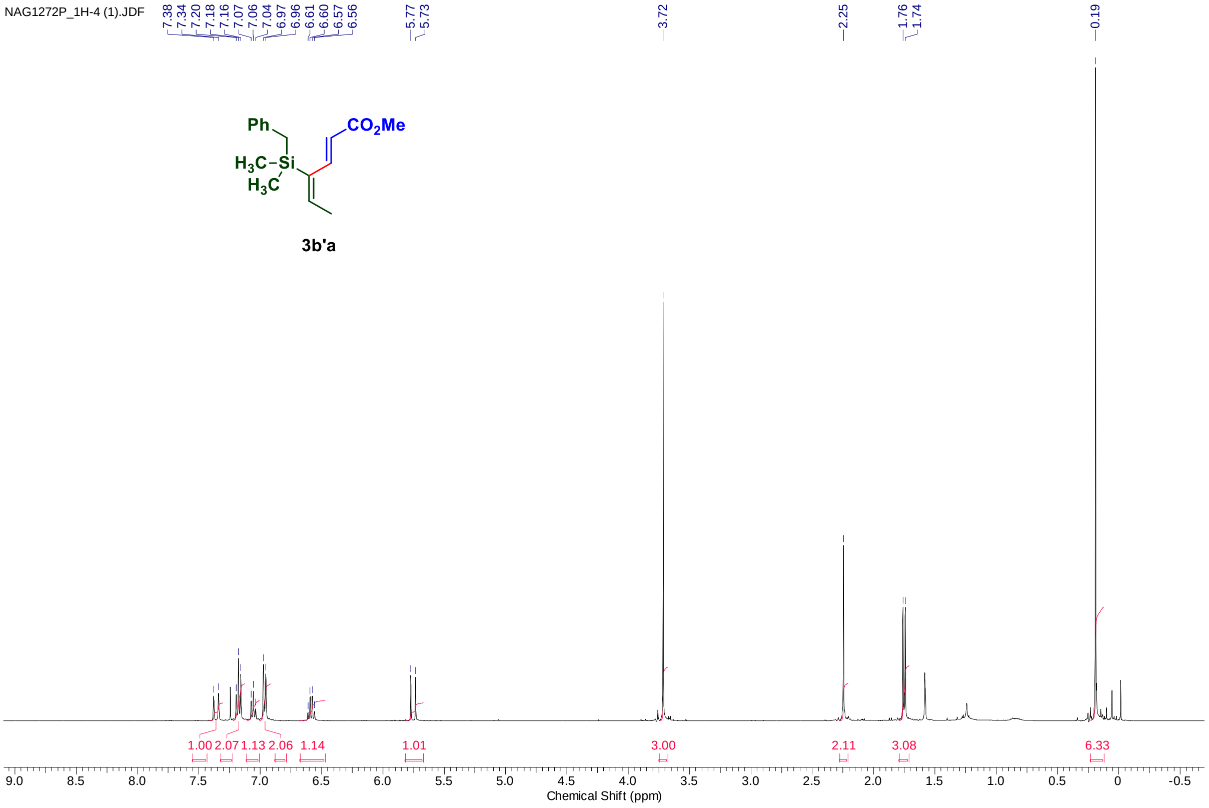
NAG1272P_1H-4 (1).JDF
7.38 7.34 7.20 7.18 7.16 7.07 7.06 7.04 6.97 6.96 6.61 6.60 6.57 6.56
5.77 5.73
3.72
2.25
1.76 1.74
0.19
Ph
CO_2Me
H_3C–Si
H_3C
3b'a
1.00 2.07 1.13 2.06 1.14
1.01
3.00
2.11
3.08
6.33
Chemical Shift (ppm)

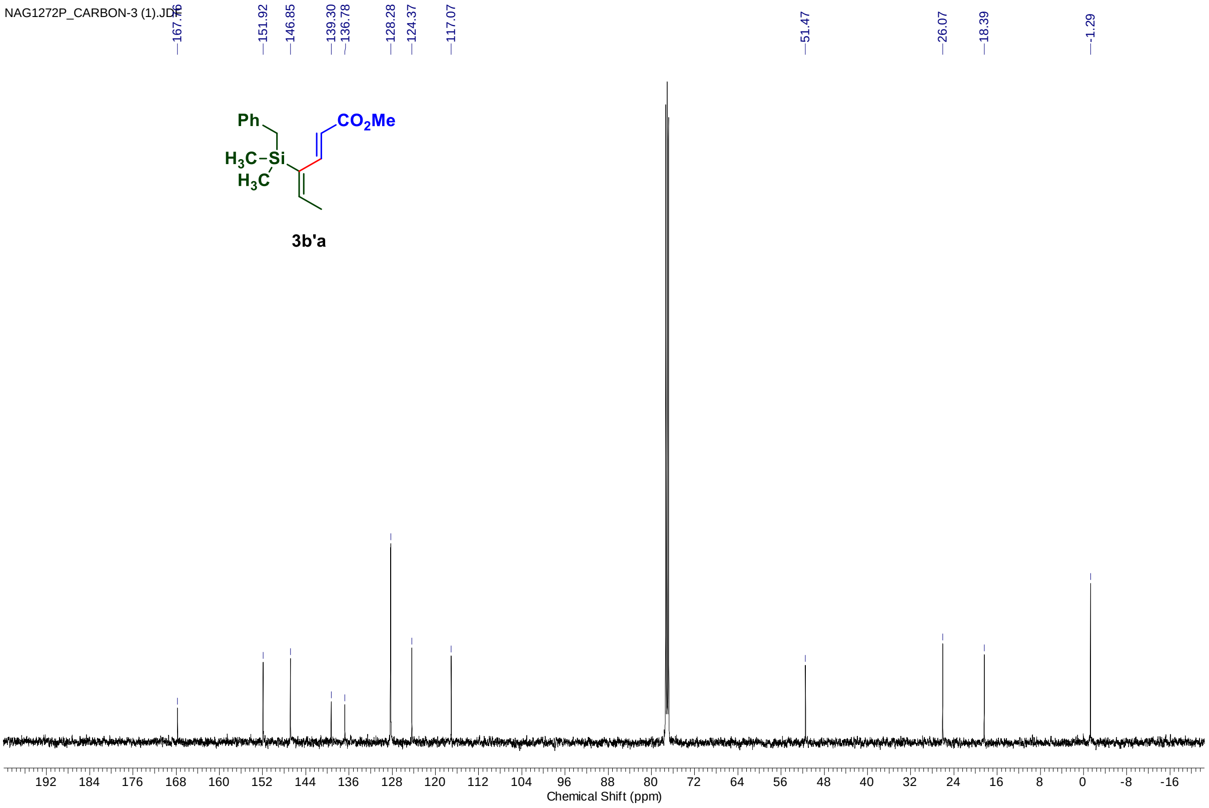
NAG1272P_CARBON-3 (1).JDF
151.92
146.85
139.30
136.78
128.28
124.37
117.07
51.47
26.07
18.39
-1.29
Ph
CO_2Me
H_3C–Si
H_3C
3b'a
Chemical Shift (ppm)

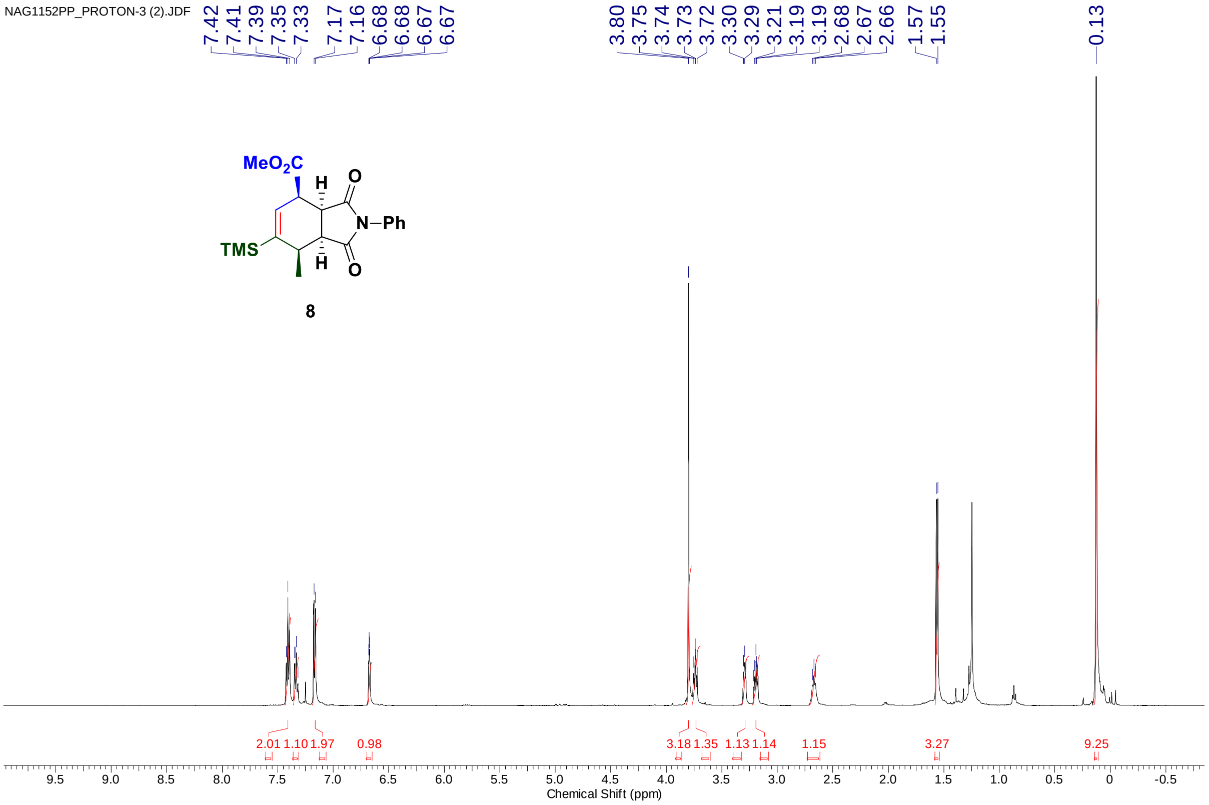
NAG1152PP_PROTON-3 (2).JDF
7.42 7.41 7.39 7.35 7.33 7.17 7.16 6.68 6.68 6.67 6.67
3.80 3.75 3.74 3.73 3.72 3.30 3.29 3.21 3.19 2.68 2.67 2.66 1.57 1.55
0.13
MeO2C
H
O
N−Ph
TMS
H
O
8
2.01 1.10 1.97 0.98 3.18 1.35 1.13 1.14 1.15 3.27 9.25
Chemical Shift (ppm)

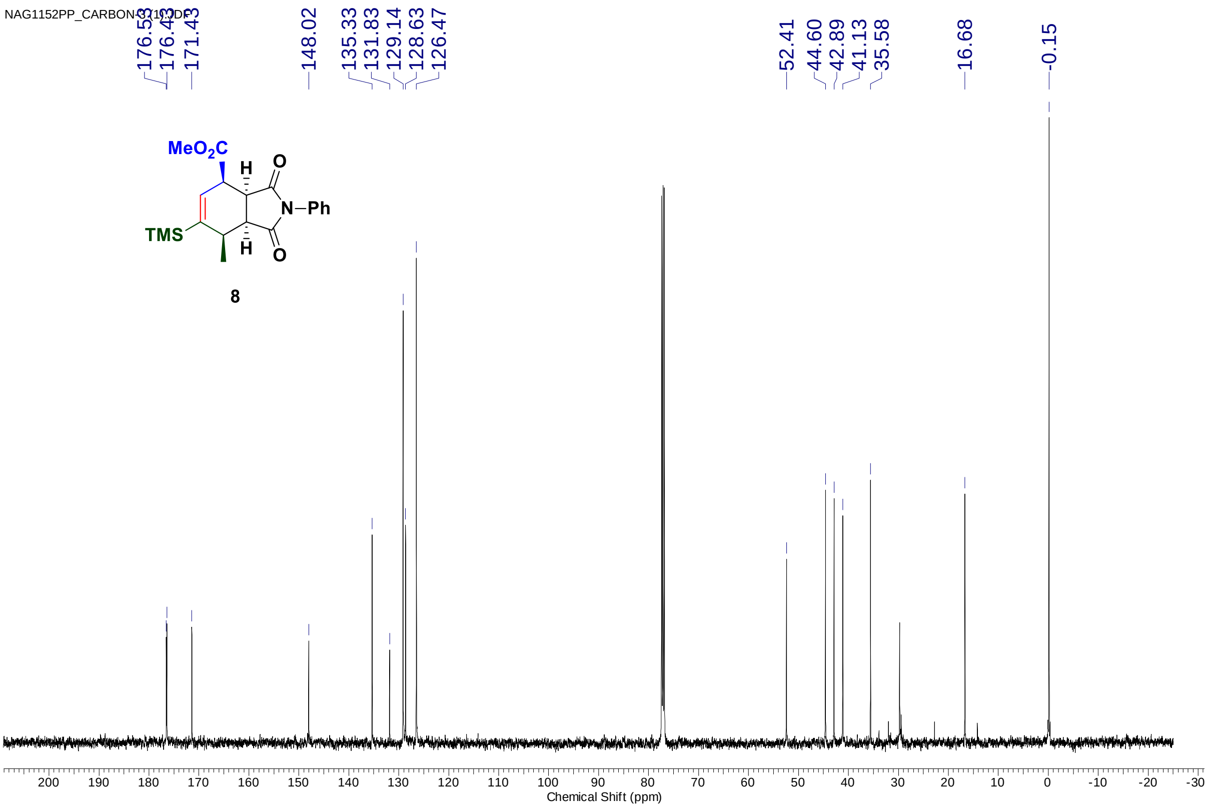
NAG1152PP_CARBON-3 (1).JDF
176.53 176.43 171.43
148.02
135.33 131.83 129.14 128.63 126.47
52.41 44.60 42.89 41.13 35.58
16.68
-0.15
MeO2C
H
O
N−Ph
TMS
H
O
8
Chemical Shift (ppm)

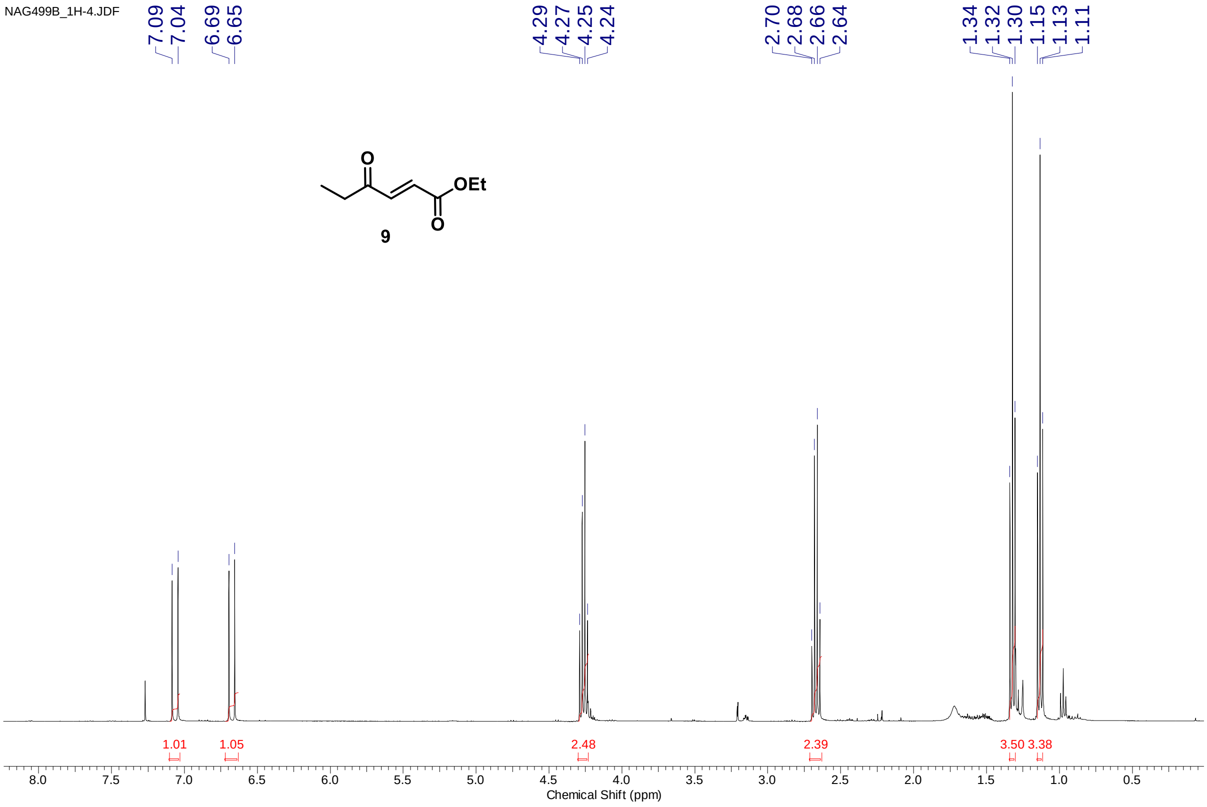
NAG499B_1H-4.JDF
7.09
7.04
6.69
6.65
4.29
4.27
4.25
4.24
2.70
2.68
2.66
2.64
1.34
1.32
1.30
1.15
1.13
1.11
O
OEt
O
9
1.01
1.05
2.48
2.39
3.50
3.38
Chemical Shift (ppm)

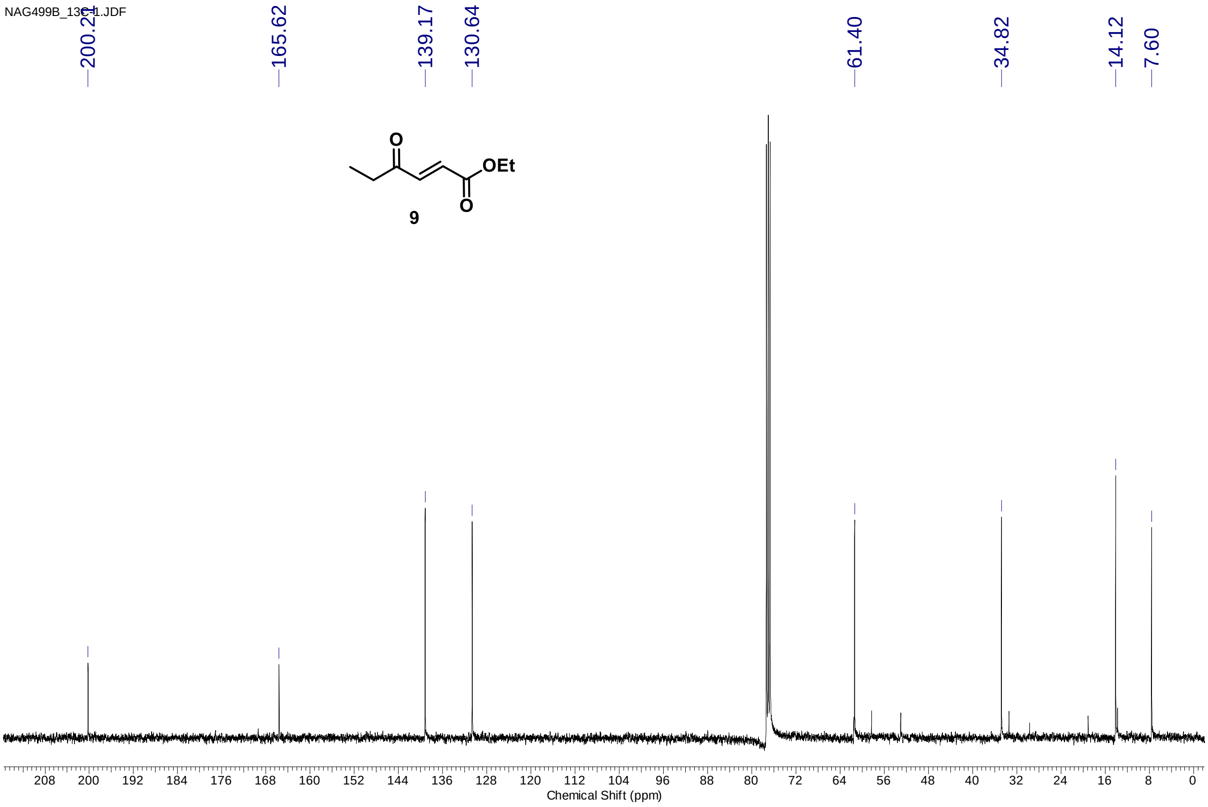
NAG499B_13C-1.JDF
165.62
139.17
130.64
61.40
34.82
14.12
7.60
O
OEt
O
9
Chemical Shift (ppm)

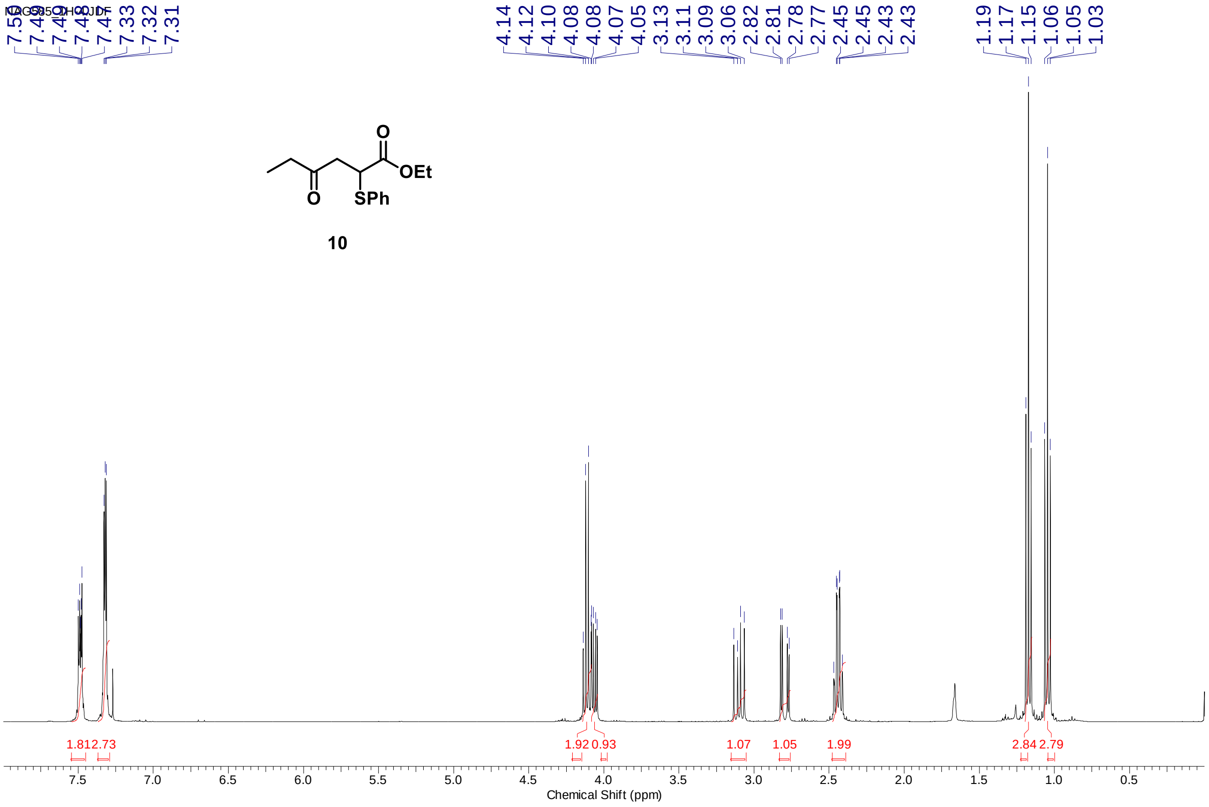
4.14
4.12
4.10
4.08
4.08
4.07
4.05
3.13
3.11
3.09
3.06
2.82
2.81
2.78
2.77
2.45
2.45
2.43
2.43
1.19
1.17
1.15
1.06
1.05
1.03
7.33
7.32
7.31
OEt
O
SPh
10
1.81
2.73
1.92
0.93
1.07
1.05
1.99
2.84
2.79
Chemical Shift (ppm)

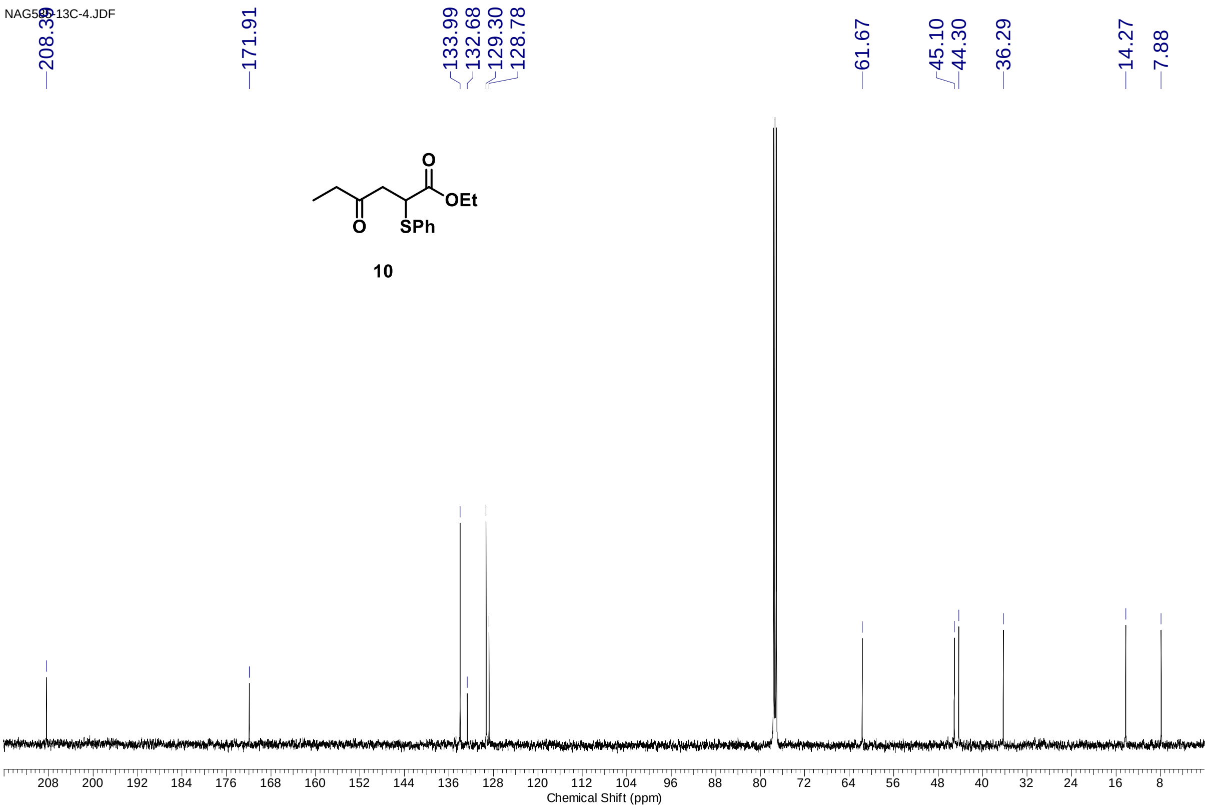
208.39
171.91
133.99
132.68
129.30
128.78
61.67
45.10
44.30
36.29
14.27
7.88
O
OEt
O
SPh
10
Chemical Shift (ppm)

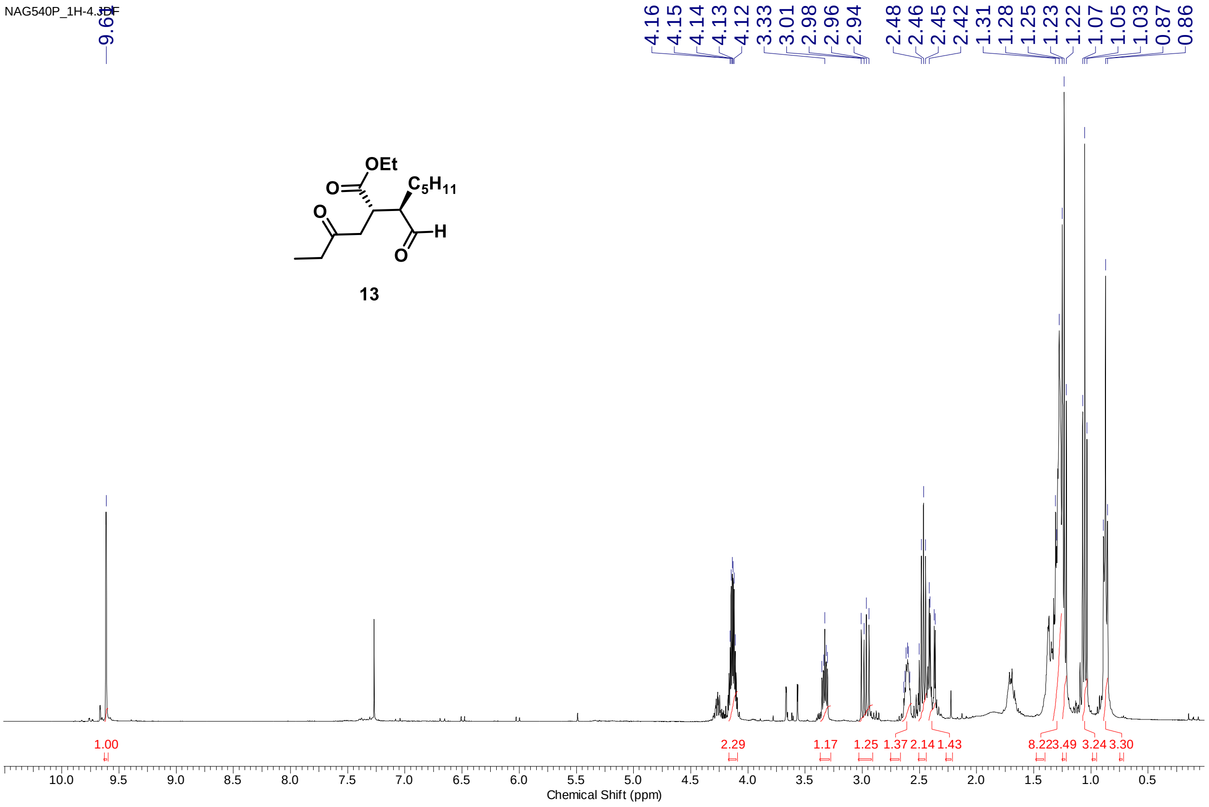
NAG540P_1H-4.JDF
9.61
4.16 4.15 4.14 4.13 4.12 3.33 3.01 2.98 2.96 2.94 2.48 2.46 2.45 2.42 1.31 1.28 1.25 1.23 1.22 1.07 1.05 1.03 0.87 0.86
OEt
O
C5H11
O
H
O
13
1.00 2.29 1.17 1.25 1.37 2.14 1.43 8.22 3.49 3.24 3.30
10.0 9.5 9.0 8.5 8.0 7.5 7.0 6.5 6.0 5.5 5.0 4.5 4.0 3.5 3.0 2.5 2.0 1.5 1.0 0.5
Chemical Shift (ppm)

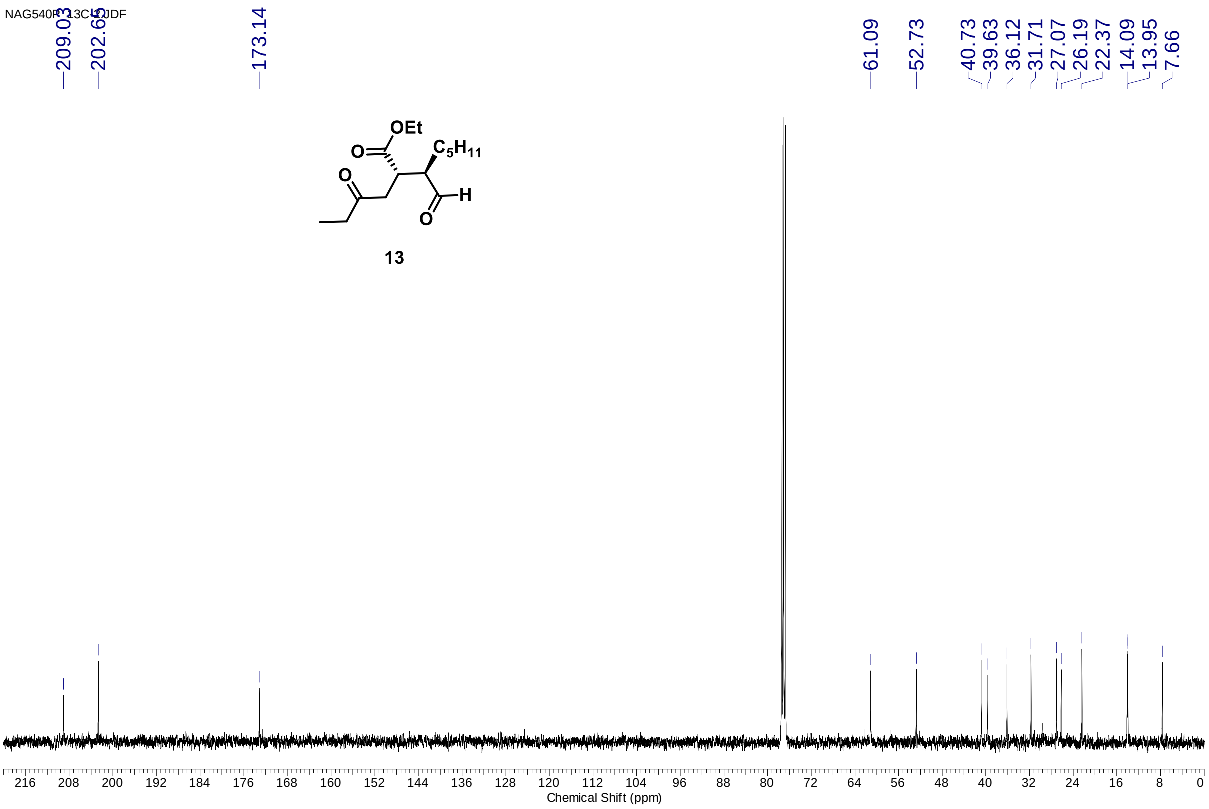
NAG540P_13C-1.JDF
209.03 202.65 173.14 61.09 52.73 40.73 39.63 36.12 31.71 27.07 26.19 22.37 14.09 13.95 7.66
OEt
O
C5H11
O
H
O
13
216 208 200 192 184 176 168 160 152 144 136 128 120 112 104 96 88 80 72 64 56 48 40 32 24 16 8 0
Chemical Shift (ppm)

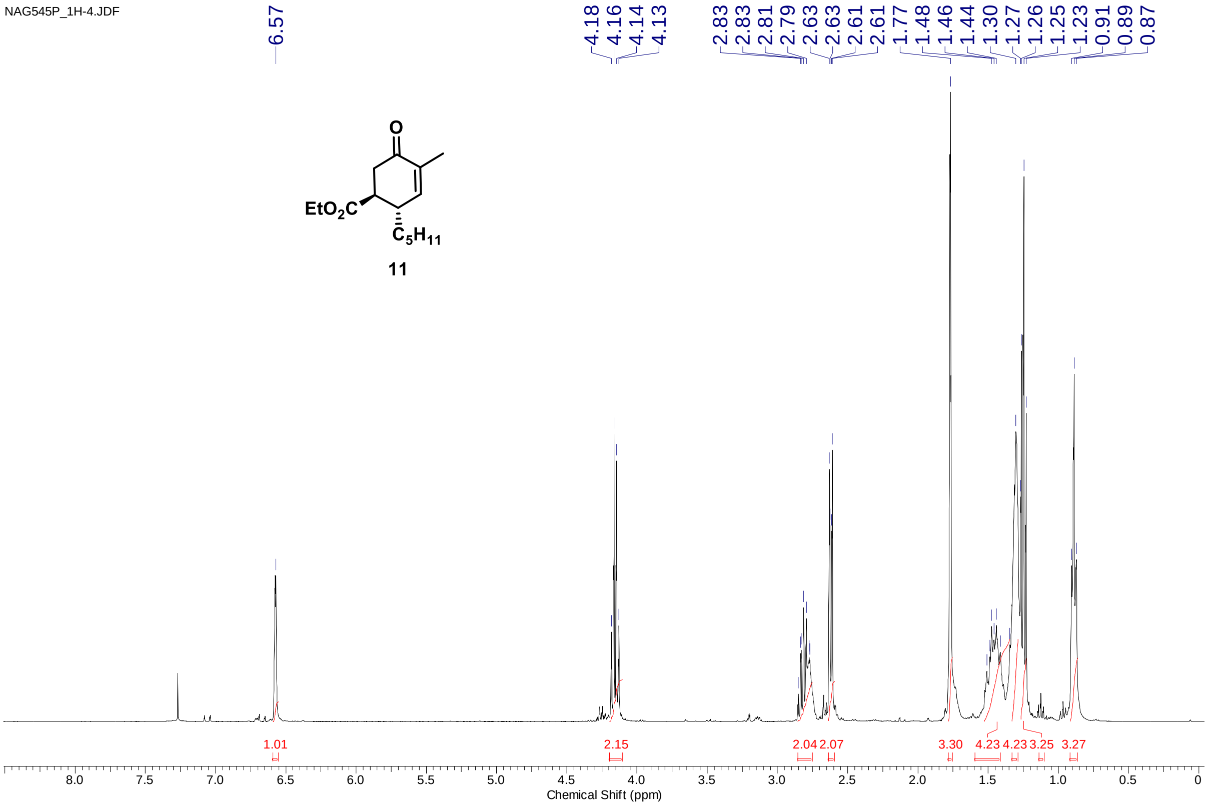
NAG545P_1H-4.JDF
6.57
4.18 4.16 4.14 4.13
2.83 2.83 2.81 2.79 2.63 2.63 2.61 2.61 1.77 1.48 1.46 1.44 1.30 1.27 1.26 1.25 1.23 0.91 0.89 0.87
O
EtO2C
C5H11
11
1.01 2.15 2.04 2.07 3.30 4.23 4.23 3.25 3.27
8.0 7.5 7.0 6.5 6.0 5.5 5.0 4.5 4.0 3.5 3.0 2.5 2.0 1.5 1.0 0.5 0
Chemical Shift (ppm)

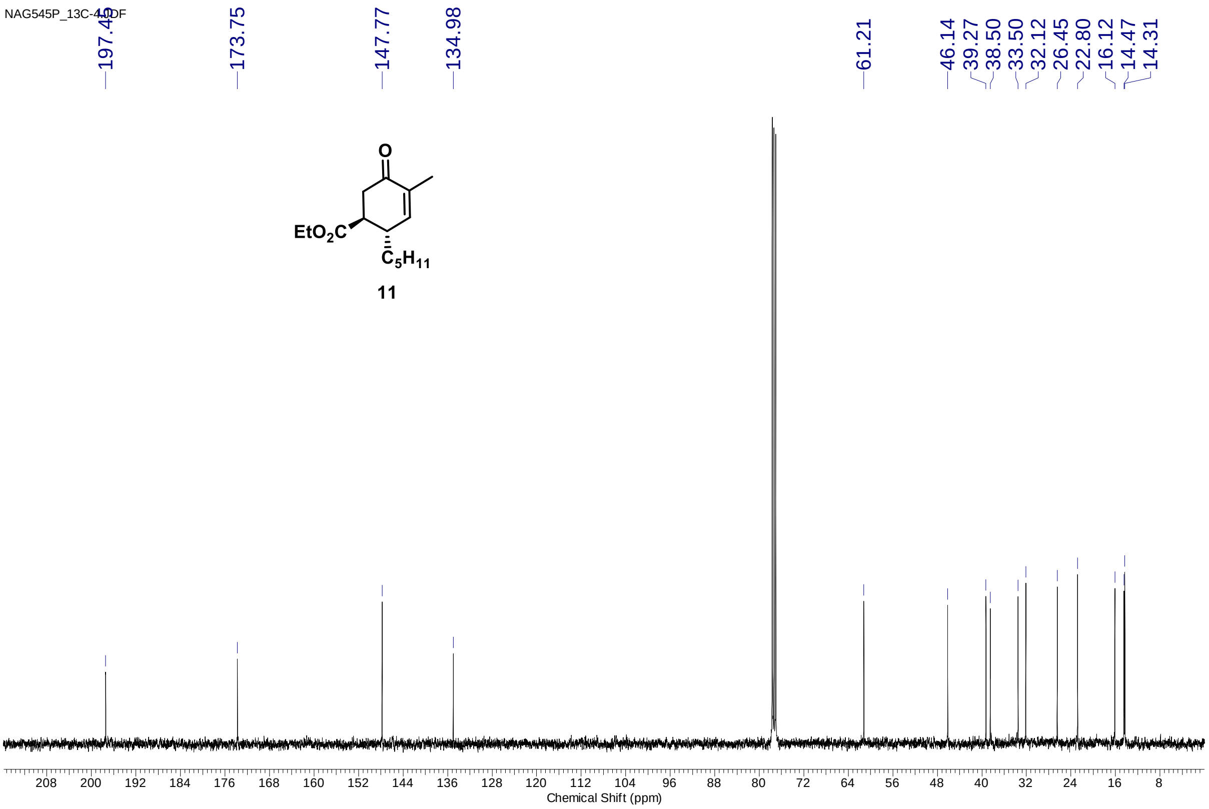
197.4
173.75
147.77
134.98
61.21
46.14 39.27 38.50 33.50 32.12 26.45 22.80 16.12 14.47 14.31
O
EtO2C
C5H11
11
208 200 192 184 176 168 160 152 144 136 128 120 112 104 96 88 80 72 64 56 48 40 32 24 16 8
Chemical Shift (ppm)

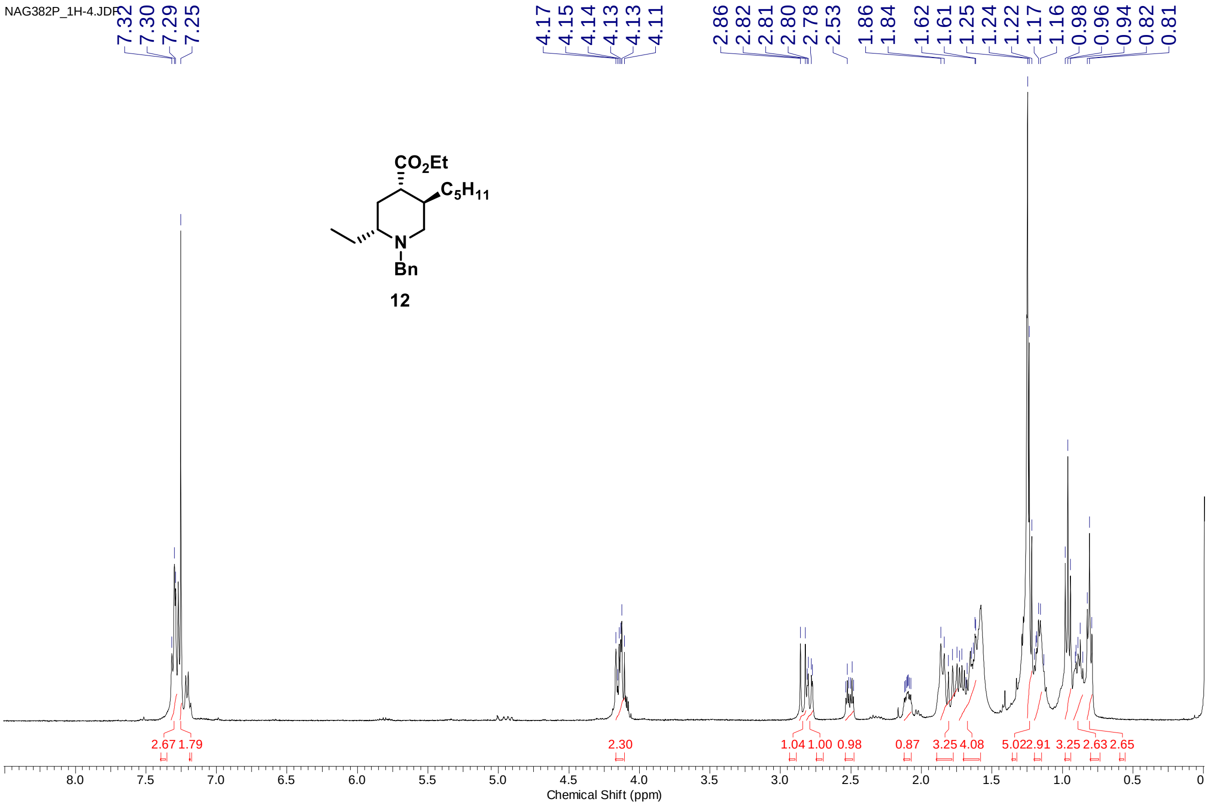
NAG382P_1H-4.JDF
7.32 7.30 7.29 7.25
4.17 4.15 4.14 4.13 4.13 4.11
2.86 2.82 2.81 2.80 2.78 2.53 1.86 1.84 1.62 1.61 1.25 1.24 1.22 1.17 1.16 0.98 0.96 0.94 0.82 0.81
CO2Et
C5H11
N
Bn
12
2.67 1.79 2.30 1.04 1.00 0.98 0.87 3.25 4.08 5.02 2.91 3.25 2.63 2.65
Chemical Shift (ppm)

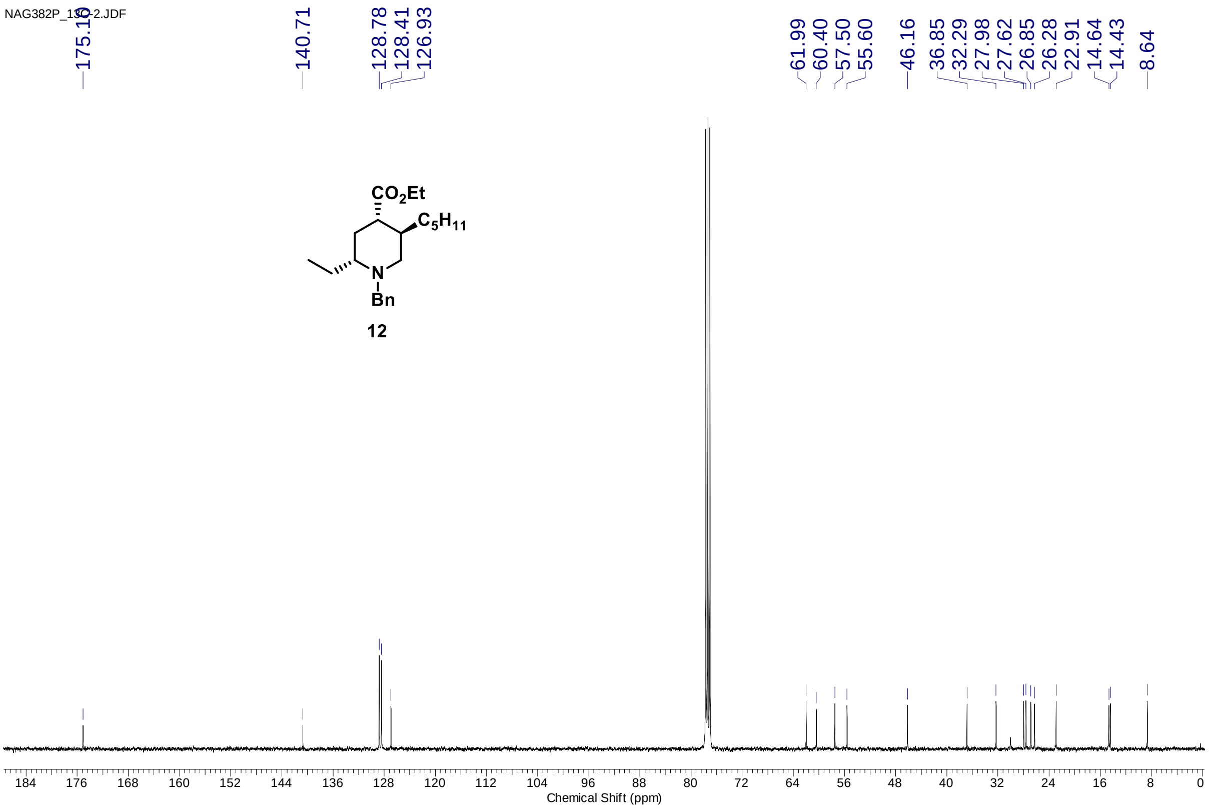
NAG382P_13C-2.JDF
175.10
140.71
128.78 128.41 126.93
61.99 60.40 57.50 55.60 46.16 36.85 32.29 27.98 27.62 26.85 26.28 22.91 14.64 14.43 8.64
CO2Et
C5H11
N
Bn
12
Chemical Shift (ppm)

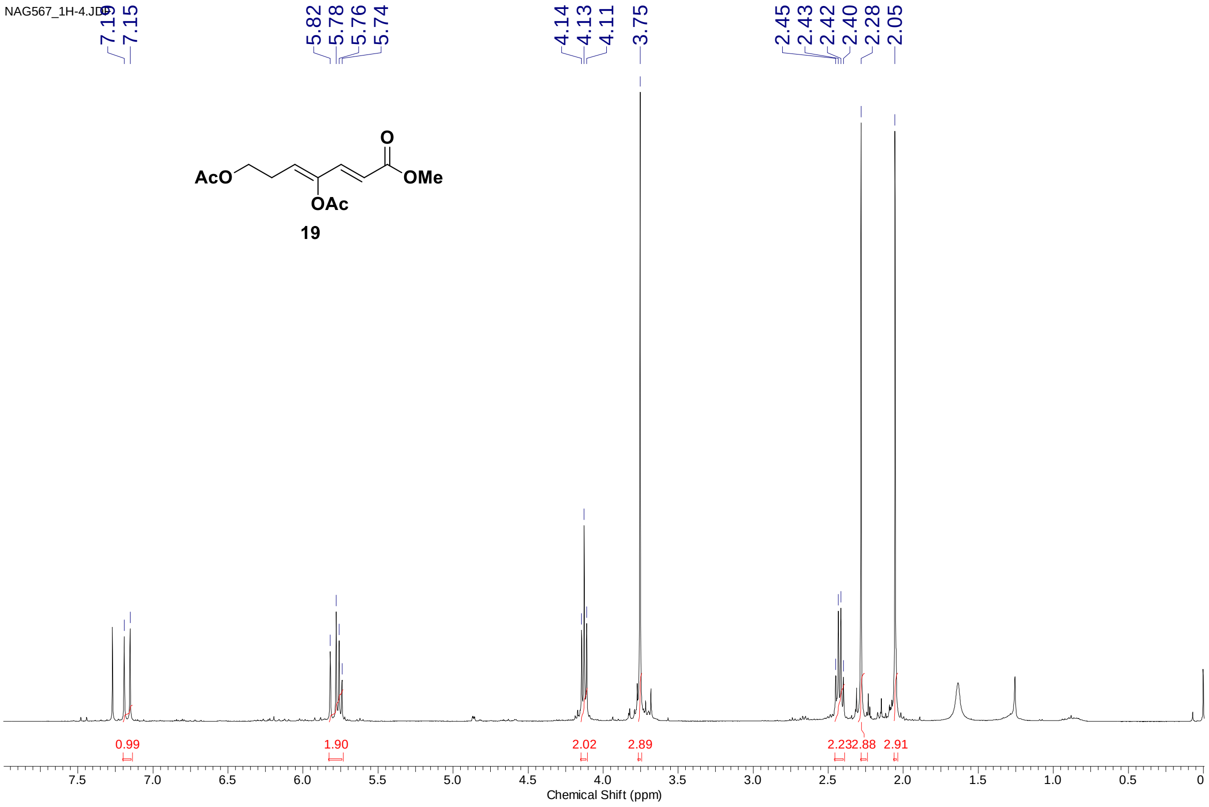
7.15
5.82
5.78
5.76
5.74
4.14
4.13
4.11
3.75
2.45
2.43
2.42
2.40
2.28
2.05
AcO
O
OMe
OAc
19
0.99
1.90
2.02
2.89
2.91
Chemical Shift (ppm)

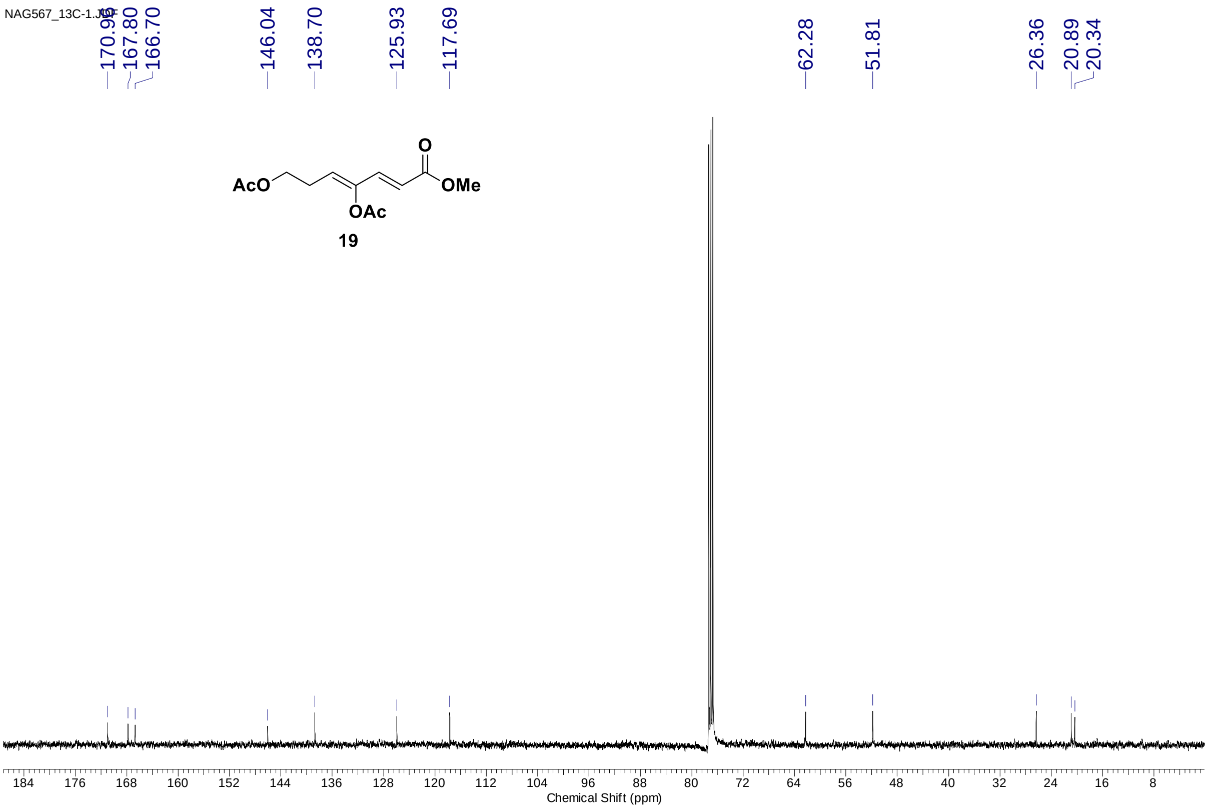
167.80
166.70
146.04
138.70
125.93
117.69
62.28
51.81
26.36
20.89
20.34
AcO
O
OMe
OAc
19
Chemical Shift (ppm)

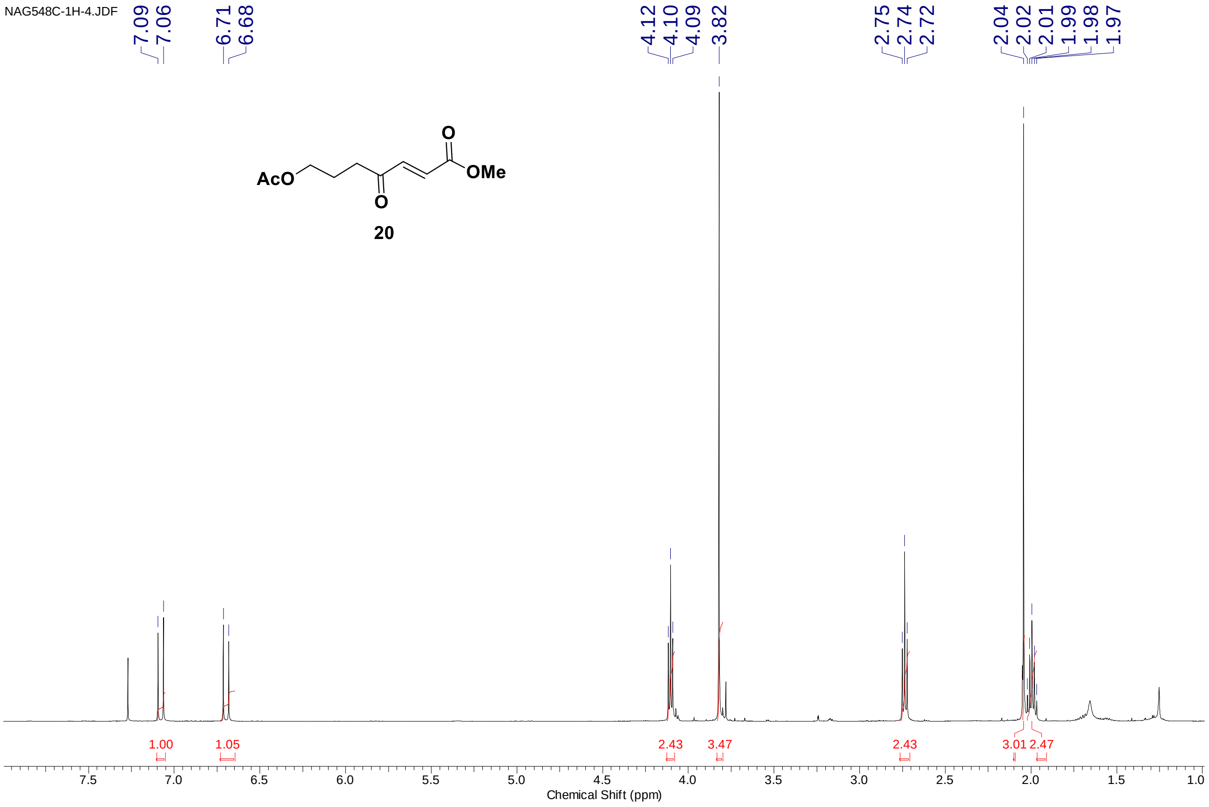
NAG548C-1H-4.JDF
7.09
7.06
6.71
6.68
4.12
4.10
4.09
3.82
2.75
2.74
2.72
2.04
2.02
2.01
1.99
1.98
1.97
AcO
OMe
O
O
20
1.00
1.05
2.43
3.47
2.43
3.01
2.47
7.5
7.0
6.5
6.0
5.5
5.0
4.5
4.0
3.5
3.0
2.5
2.0
1.5
1.0
Chemical Shift (ppm)

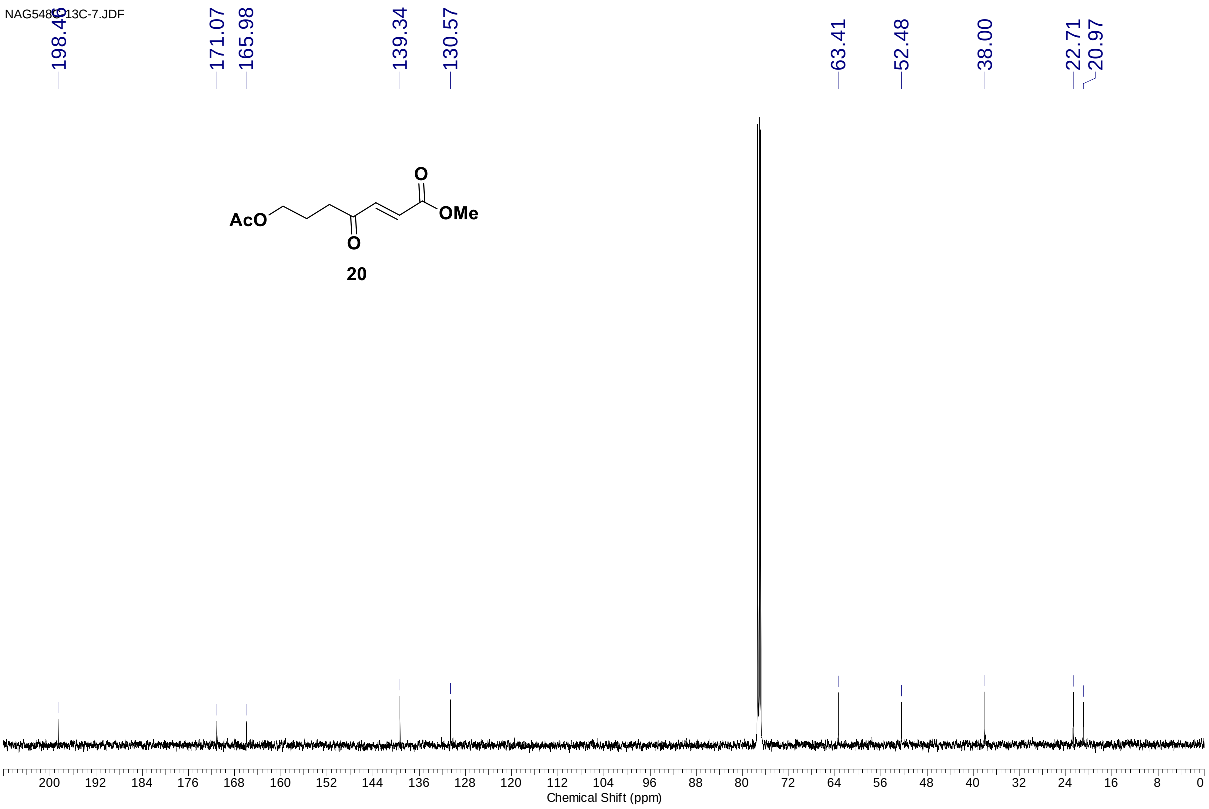
NAG548C-13C-7.JDF
198.40
171.07
165.98
139.34
130.57
63.41
52.48
38.00
22.71
20.97
AcO
OMe
O
O
20
200
192
184
176
168
160
152
144
136
128
120
112
104
96
88
80
72
64
56
48
40
32
24
16
8
0
Chemical Shift (ppm)

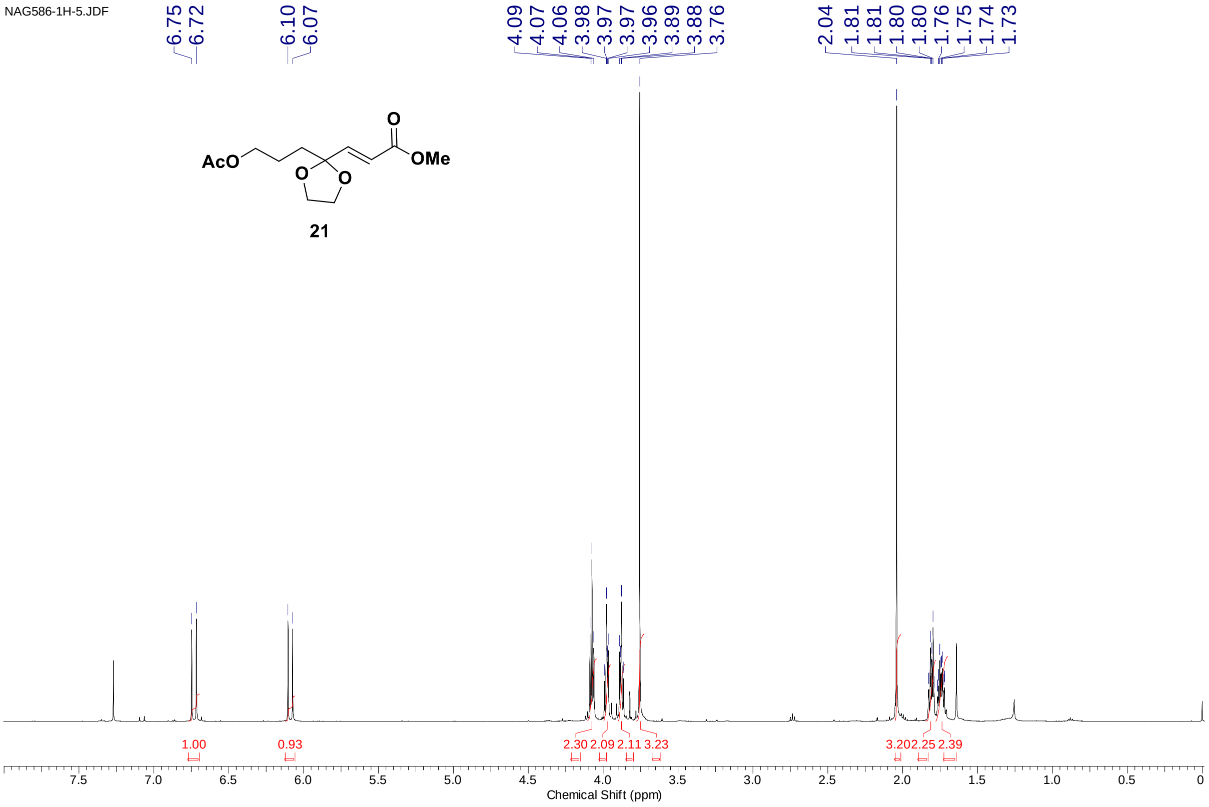
NAG586-1H-5.JDF
6.75
6.72
6.10
6.07
4.09
4.07
4.06
3.98
3.97
3.97
3.96
3.89
3.88
3.76
2.04
1.81
1.81
1.80
1.80
1.76
1.75
1.74
1.73
AcO
O
O
O
OMe
21
1.00
0.93
2.30
2.09
2.11
3.23
3.20
2.25
2.39
7.5
7.0
6.5
6.0
5.5
5.0
4.5
4.0
3.5
3.0
2.5
2.0
1.5
1.0
0.5
0
Chemical Shift (ppm)

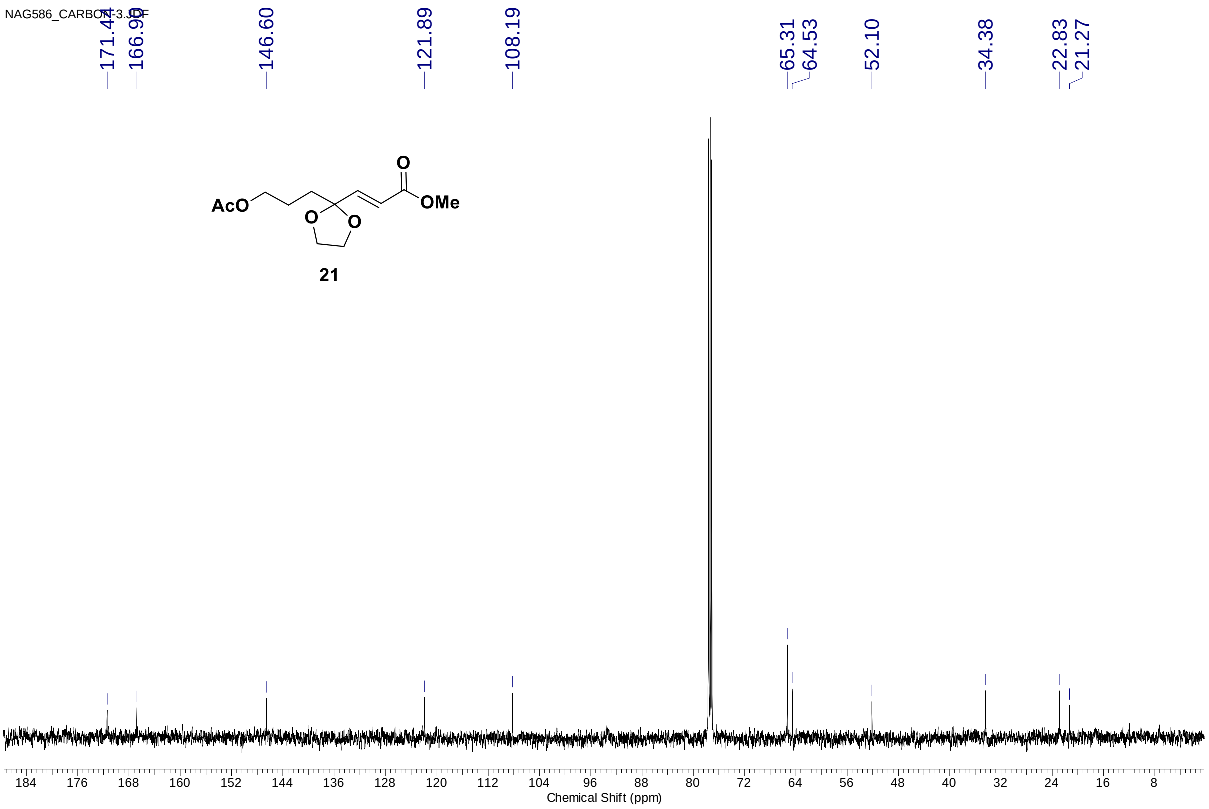
NAG586_CARBON-3.JDF
171.44
166.90
146.60
121.89
108.19
65.31
64.53
52.10
34.38
22.83
21.27
AcO
O
O
O
OMe
21
184
176
168
160
152
144
136
128
120
112
104
96
88
80
72
64
56
48
40
32
24
16
8
Chemical Shift (ppm)

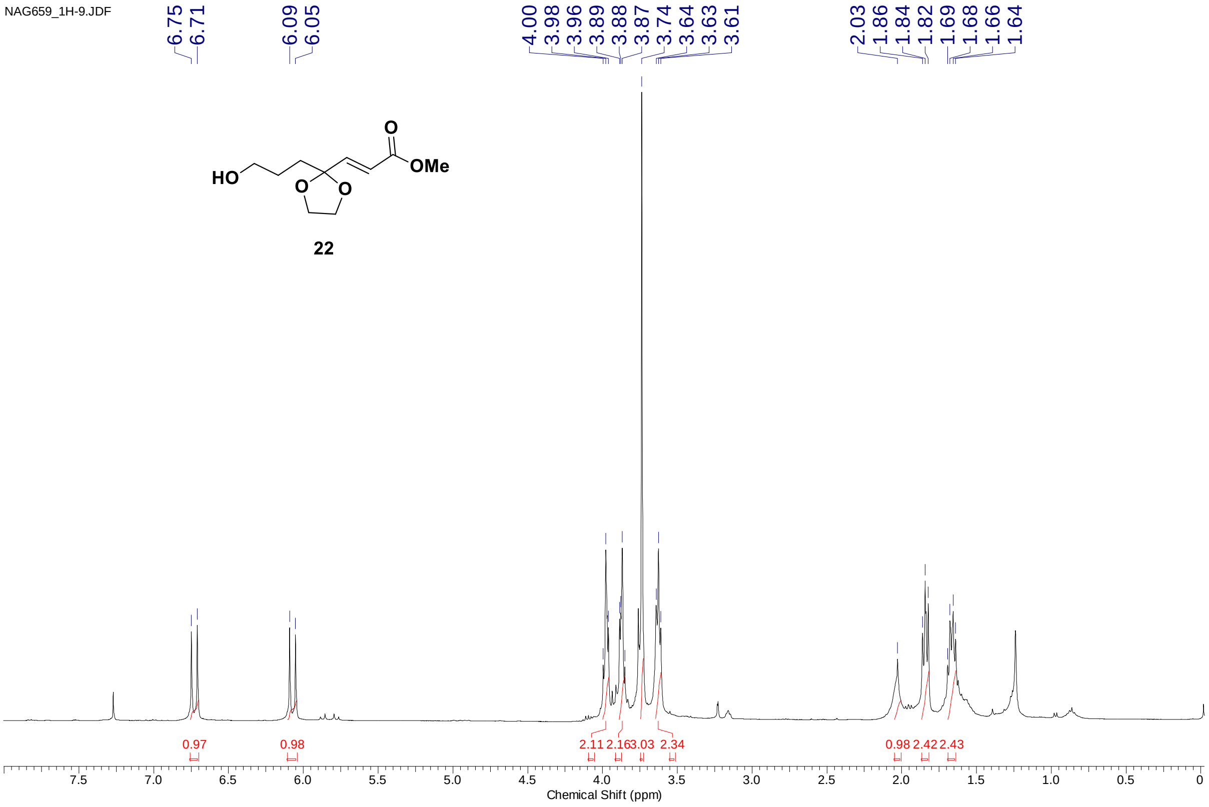
NAG659_1H-9.JDF
6.75
6.71
6.09
6.05
4.00
3.98
3.96
3.89
3.88
3.87
3.74
3.64
3.63
3.61
2.03
1.86
1.84
1.82
1.69
1.68
1.66
1.64
HO
O
O
O
OMe
22
0.97
0.98
2.11
2.16
3.03
2.34
0.98
2.42
2.43
Chemical Shift (ppm)

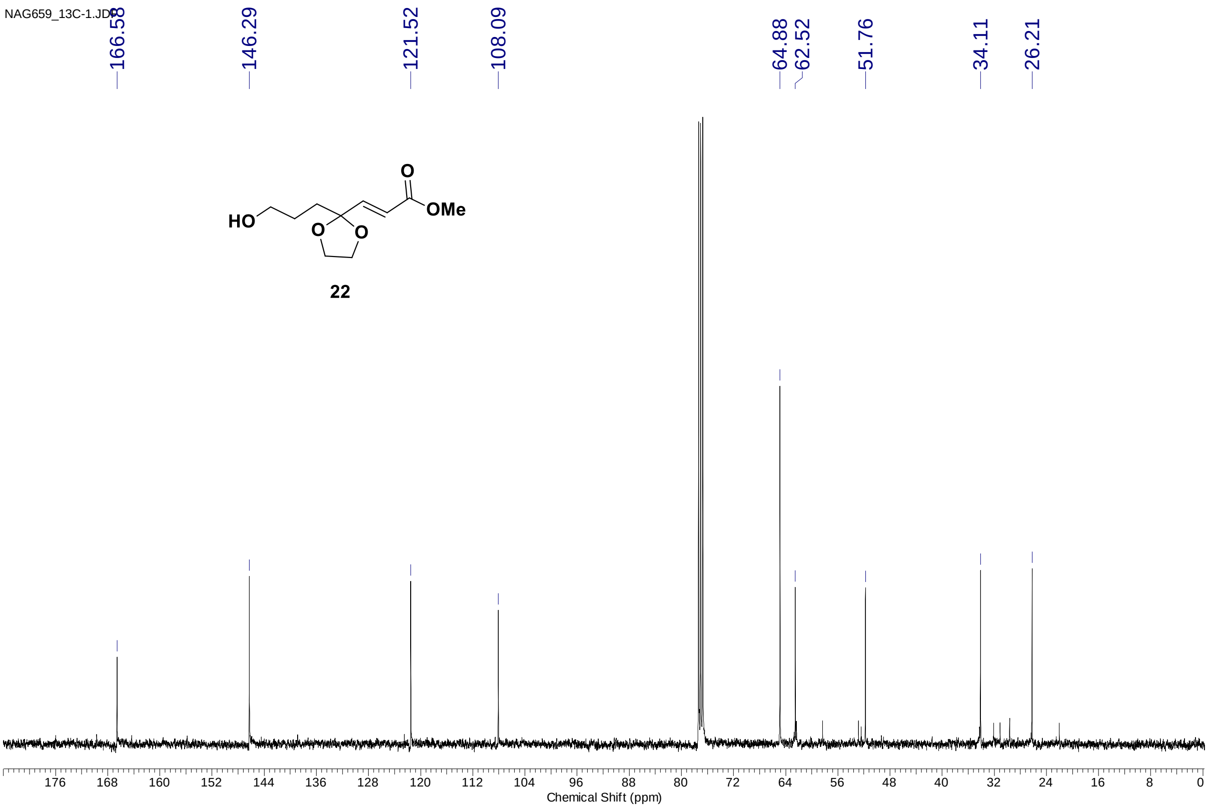
NAG659_13C-1.JDF
166.58
146.29
121.52
108.09
64.88
62.52
51.76
34.11
26.21
HO
O
O
O
OMe
22
Chemical Shift (ppm)

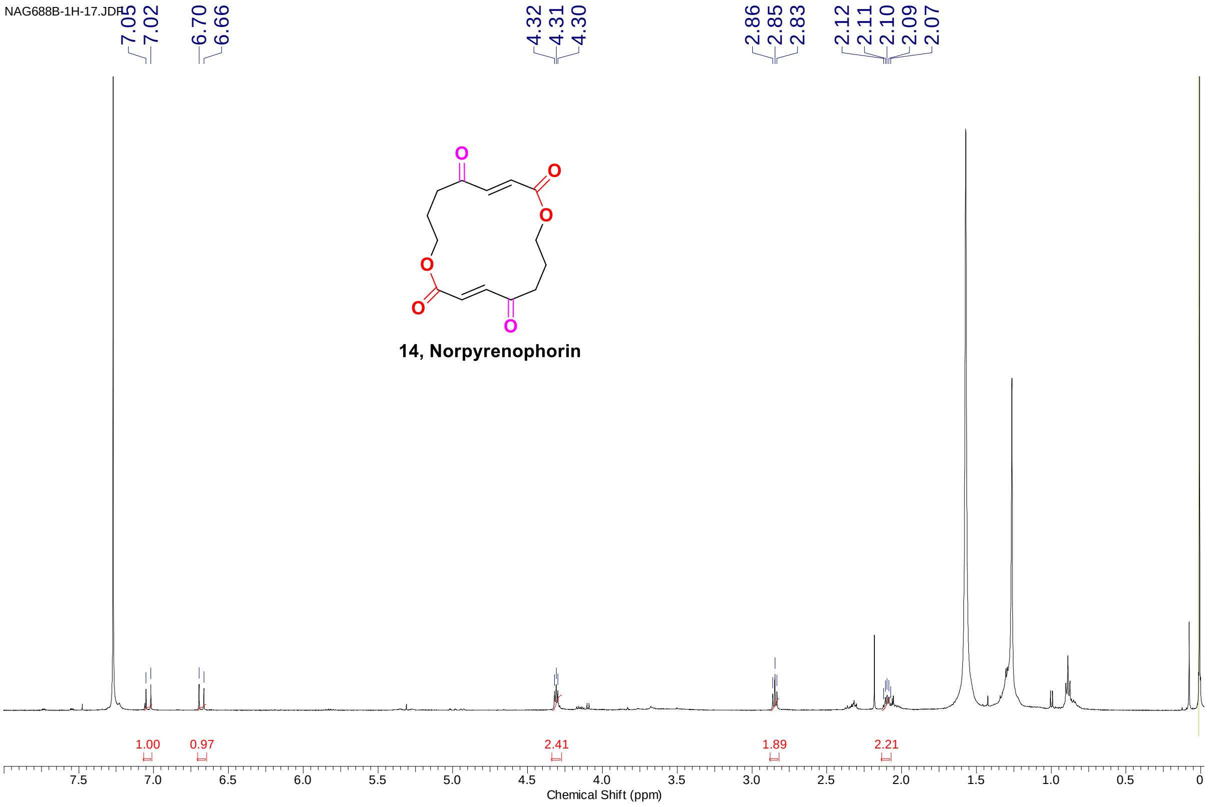
NAG688B-1H-17.JDF
7.05
7.02
6.70
6.66
4.32
4.31
4.30
2.86
2.85
2.83
2.12
2.11
2.10
2.09
2.07
14, Norpyrenophorin
1.00
0.97
2.41
1.89
2.21
7.5
7.0
6.5
6.0
5.5
5.0
4.5
4.0
3.5
3.0
2.5
2.0
1.5
1.0
0.5
0
Chemical Shift (ppm)

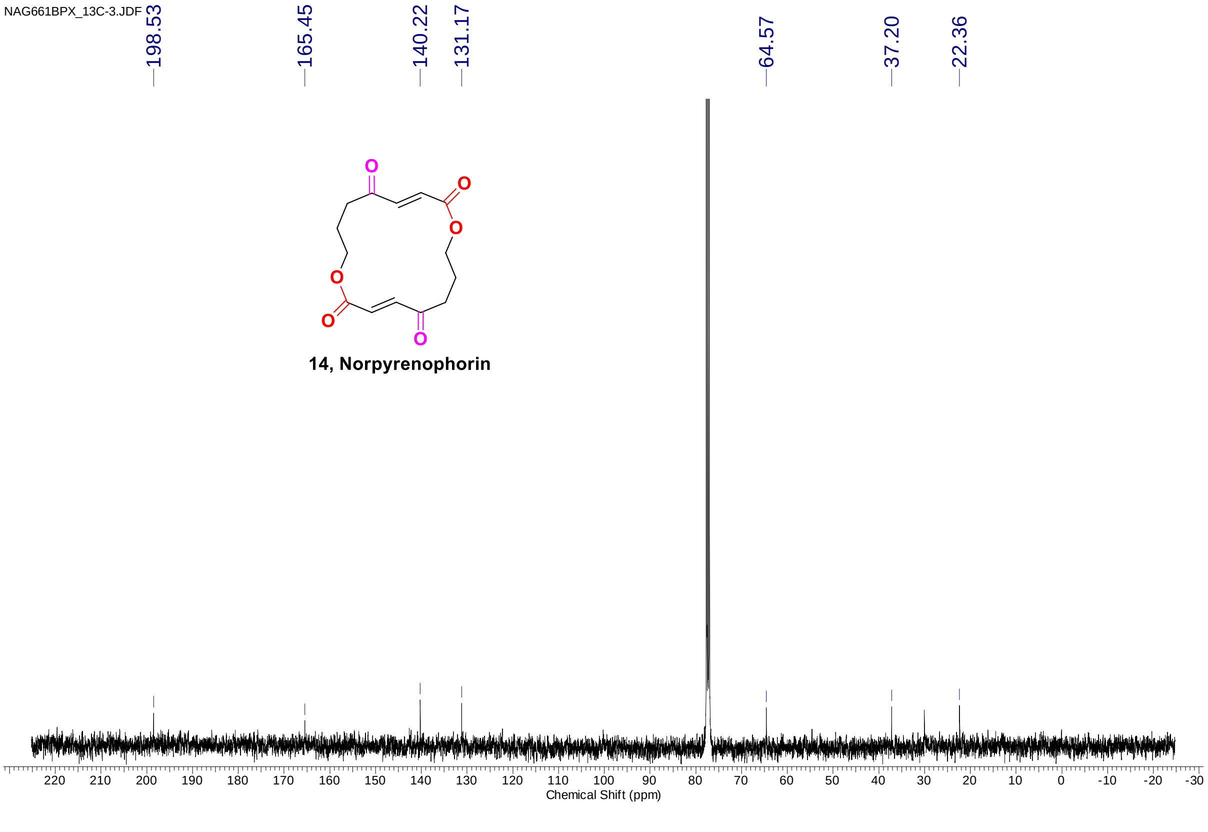
NAG661BPX_13C-3.JDF
198.53
165.45
140.22
131.17
64.57
37.20
22.36
14, Norpyrenophorin
220
210
200
190
180
170
160
150
140
130
120
110
100
90
80
70
60
50
40
30
20
10
0
-10
-20
-30
Chemical Shift (ppm)

4. References

1. Wang, J.; Ma, A.; Ma, D., Organocatalytic Michael Addition of Aldehydes to γ-Keto-α,β-unsaturated Esters. An Efficient Entry to Versatile Chiral Building Blocks. *Organic Letters* **2008,***10* (23), 5425-5428.

2. Kowalczyk, R.; Wierzba, A. J.; Boratynski, P. J.; Bakowicz, J., Enantioselective conjugate addition of aliphatic thiols to divergently activated electron poor alkenes and dienes. *Tetrahedron* **2014,***70* (35), 5834-5842.

3. Shen, L.-L.; Mun, H.-S.; Jeong, J.-H., Macrocyclisation of Macrodiolide with Dimethylaluminium Methaneselenolate. *Eur. J. Org. Chem.* **2010,** (36), 6895-6899, S6895/1-S6895/6.
